# AI‐Driven *De Novo* Design of Ultra Long‐Acting GLP‐1 Receptor Agonists

**DOI:** 10.1002/advs.202507044

**Published:** 2025-08-11

**Authors:** Ting Wei, Jiating Ma, Xiaochen Cui, Jiahui Lin, Zhuoqi Zheng, Liu Cheng, Taiying Cui, Xiaoqian Lin, Junjie Zhu, Xuyang Ran, Xiaokun Hong, Luke Johnston, Zhangsheng Yu, Haifeng Chen

**Affiliations:** ^1^ State Key Laboratory of Microbial Metabolism Department of Bioinformatics and Biostatistics SJTU‐Yale Joint Center for Biostatistics National Experimental Teaching Center for Life Sciences and Biotechnology School of Life Sciences and Biotechnology Shanghai Jiao Tong University Shanghai 200240 China; ^2^ Intelligent Medicine Original Medical Technology (Shanghai) Co., Ltd. Shanghai 200240 China; ^3^ College of Biological Science and Engineering Fuzhou University Fuzhou Fujian 350116 China; ^4^ School of Mathematical Sciences Shanghai Jiao Tong University Shanghai 200240 China; ^5^ Clinical Research Institute Shanghai Jiao Tong University School of Medicine Shanghai 200025 China

**Keywords:** deep learning, GLP‐1RAs, protein design, Semaglutide

## Abstract

Peptide drugs have revolutionized modern therapeutics, offering novel treatment avenues for various diseases. Nevertheless, low design efficacy, time consumption, and high cost still hinder peptide drug design and discovery. Here, an efficient approach that integrates deep learning‐based protein design with functional screening is presented, enabling the rapid design of biotechnologically important peptides with improved stability and efficacy. 10,000 *de novo* glucagon‐like peptide‐1 receptor agonists (GLP‐1RAs) are designed, 60 of these satisfied the stability, efficacy, and diversity criteria in the virtual functional screening. In vitro validations reveal a 52% success rate, and in vivo experiments demonstrate that two lead GLP‐1RAs (D13 and D41) exhibit extended half‐lives, approximately three times longer than that of Semaglutide. In diabetic mouse models, candidate D13 results in significantly lower blood glucose levels than Semaglutide. In the obesity mouse model, D13 induces weight loss efficacy comparable to that of Semaglutide. The AI‐driven peptide design pipeline—which integrates protein design, functional screening, and experimental validation—reduces the number of iterations required to find novel peptide candidates. The entire process, from design to screening, can be completed in a single cycle within two weeks.

## Graphical abstract

*De novo* GLP‐1RAs can be computationally designed to exhibit extended half‐life and superior efficacy compared to Semaglutide.

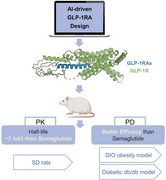

## 1 Introduction

Peptides are emerging as a crucial class of therapeutics due to their high target specificity, strong biological activity, and safety.^[^ ^1^ ^]^ Their clinical applications span oncology, metabolic disorders, infectious diseases, and neurological conditions.^[^ ^1^ ^]^ The global peptide therapeutics market size was estimated at USD 49.13 billion in 2024 and is predicted to reach around USD 83.75 billion by 2034.^[^ ^2^ ^]^ Despite the expanding application of peptides in biomedical fields, peptide drugs face challenges such as poor metabolic stability, short plasma half‐life, and rapid proteolytic degradation.^[^ ^3^ ^]^

Recently, deep learning‐based protein design methods have emerged as powerful tools for generating *de novo* proteins with desirable properties, such as remarkable thermal stability^[^ ^4^ ^]^ and high activity.^[^ ^5^, ^6^, ^7^ ^]^ Similar AI‐based strategies are now accelerating peptide drug discovery.^[^ ^8^ ^]^ For example, a unimolecular GCGR/GLP‐1R dual agonist model has been developed to design molecules with enhanced receptor activation in vitro,^[^ ^9^ ^]^ and the diffusion model HelixDiff^[^ ^10^ ^]^ has been used to design a GLP‐1 analogue demonstrating GLP‐1R activation in vitro. Computational methods have also been applied to design G protein‐coupled receptors (GPCRs) agonists and antagonists with high affinity, potency, and selectivity in vitro.^[^ ^11^ ^]^ EvoBind^[^ ^12^ ^]^ has designed cyclic peptide agonists targeting GCGR/GLP‐1R in vitro. Furthermore, computational approaches have been utilized to design peptide inhibitors targeting β‐catenin,^[^ ^13^ ^]^ NF‐κB,^[^ ^13^ ^]^ and IL‐23R,^[^ ^14^ ^]^ and IL‐17.^[^ ^14^ ^]^ Meanwhile, in antimicrobial peptides (AMPs) research, deep learning methods such as AMP‐Designer^[^ ^15^ ^]^ and TransSAFP^[^ ^16^ ^]^ have been developed to design AMPs with broad‐spectrum activity and self‐assembling capabilities in vivo. However, most reported AI‐generated peptides have only been validated in vitro,^[^ ^9^, ^10^, ^11^, ^12^, ^16^, ^17^, ^18^ ^]^ and rigorous in vivo benchmarks against best‐in‐class drugs are still scarce,^[^ ^14^, ^15^, ^19^ ^]^ creating an urgent need for in vivo studies to demonstrate true clinical potential.

**Figure 1:**
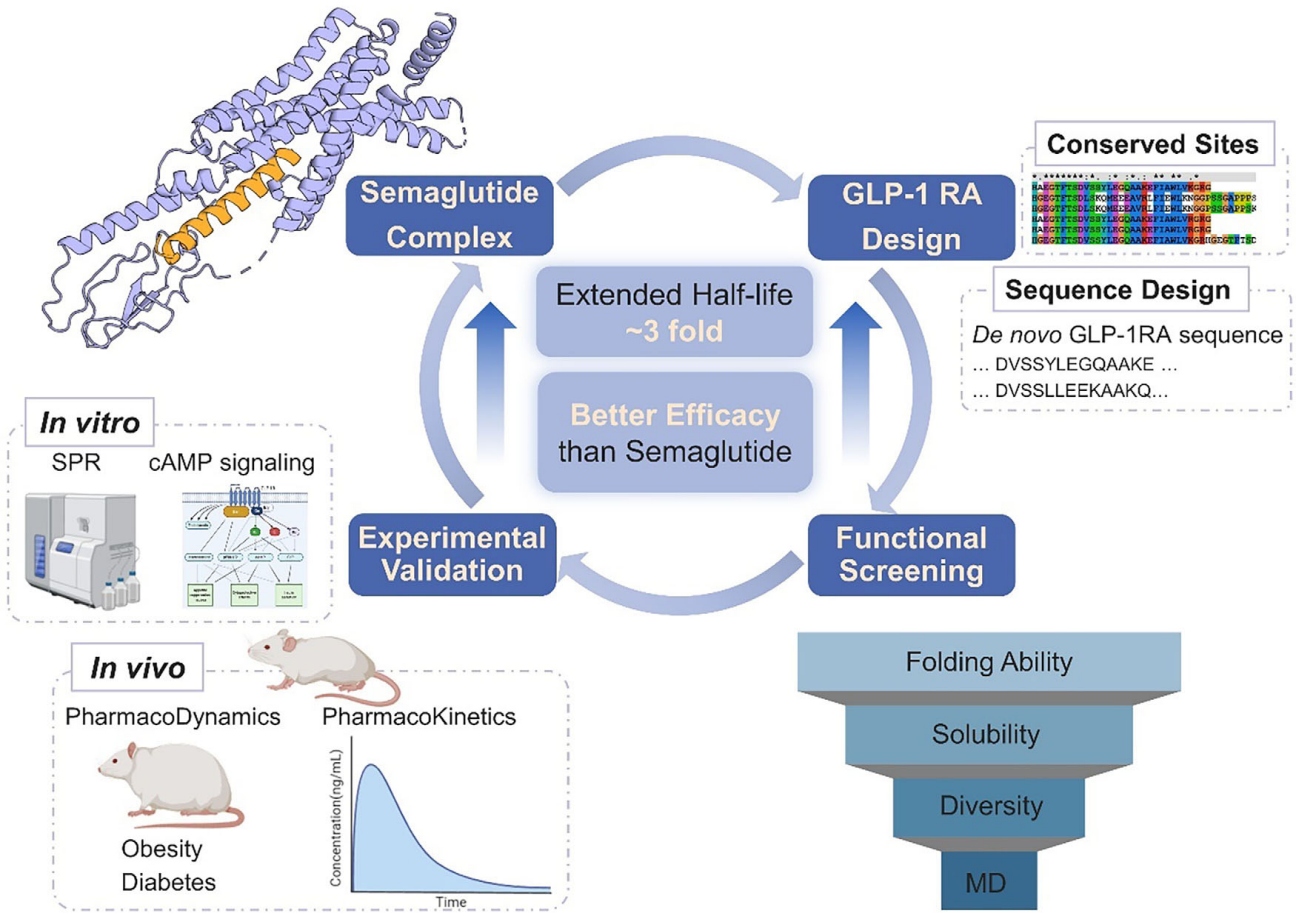
Workflow of the deep learning‐based peptide design, functional screening, and experimental validation pipeline. This workflow includes *de novo* GLP‐1RAs design, computational screening, and experimental validation through in vitro and in vivo studies.

In parallel with design algorithms, efficient functional screening also plays a crucial role in modern drug discovery, narrowing millions of designed proteins to a tractable set for experimental assays. Coupling deep learning‐based protein design method with functional screening has markedly accelerated enzyme engineering, such as serine hydrolases,^[^ ^6^ ^]^ myoglobin,^[^ ^20^ ^]^ and tobacco etch virus (TEV) protease.^[^ ^7^ ^]^ This two‐step design pipeline has significantly improved the accuracy and efficiency of protein design while reducing time and costs associated with traditional methods. Compared to enzyme design, peptide design presents unique challenges, as peptides are susceptible to proteolytic degradation and metabolic instability, necessitating optimization of plasma half‐life and biological activity. Here, we integrate deep learning‐based protein design with efficient computational screening to successfully design promising peptide candidates with high stability, specificity, and efficacy in vivo (**Figure** **1**). We first used ProteinMPNN^[^ ^21^ ^]^ for protein design, which has been successfully applied in previous studies to engineer proteins with enhanced stabilities and target‐binding affinities. We then narrowed down the designed peptides for experimental assay based on stability, efficacy, and diversity screening. Stability is evaluated through enzymatic degradation, helicity, isoelectric point, and hydrophobicity. Efficacy is assessed by folding ability and binding affinity with molecular dynamics (MD) simulation. The diversity reduced sequence similarity to existing market drugs (to avoid potential intellectual property conflicts) while maximizing variability among the designed sequences to increase the likelihood of experimental success.

We demonstrate the effectiveness of our two‐step peptide design method by designing GLP‐1RAs.^[^ ^22^ ^]^ The designed GLP‐1RAs have enhanced biological properties, such as extended half‐life, lower blood glucose, and weight loss effects in the mouse models. GLP‐1RAs have gained significant attention for their efficacy in managing type 2 diabetes mellitus (T2DM),^[^ ^23^, ^24^ ^]^ obesit,^[^ ^23^, ^24^ ^]^ and mental health disorders.^[^ ^25^ ^]^ GLP‐1 RA drugs have been developed from daily formulations to weekly formulations. The currently approved long‐acting GLP‐1RA includes once weekly Exenatide,^[^ ^26^ ^]^ Dulaglutide,^[^ ^27^ ^]^ and Semaglutide.^[^ ^27^ ^]^ Current research focuses on developing ultra‐long‐acting GLP‐1RA with extended half‐life and better efficacy.^[^ ^28^ ^]^ Ultra‐long‐acting GLP‐1RAs have the advantage of reduced dosing frequency, ease of use, and better safety profiles.^[^ ^29^, ^30^, ^31^, ^32^ ^]^

Our two‐step peptide design pipeline combines deep learning‐based protein design with computational screening to efficiently design *de novo* peptides with improved activity, stability, and efficacy. We employed ProteinMPNN for designing 10,000 novel GLP‐1RAs, of which 60 passed the functional screening based on stability, efficacy, and diversity criteria. Notably, we successfully designed a GLP‐1RA with a half‐life approximately three times longer than Semaglutide and demonstrated superior efficacy compared to Semaglutide in diabetic nephropathy and obesity. Engineering new GLP‐1RA with extended half‐life and better efficacy requires a time‐consuming and expensive iterative cycle of design‐make‐test‐analyse (DMTA).^[^ ^33^ ^]^ Our pipeline integrates AI‐powered, high‐throughput protein design with efficient functional screening, enabling the successful design of *de novo* GLP‐1RA candidates in a single cycle lasting approximately two weeks.

## 2 Results

Figure 1 provides an overview of the deep learning based peptide design pipeline, seamlessly integrating efficient functional screening and experimental validation to enable the rapid design of promising peptide candidates with extended half‐life and enhanced efficacy. Using GLP‐1RAs as a case study, we successfully designed 60 *de novo* GLP‐1RAs with improved stability, specificity, and efficacy within two weeks. Among these, three candidates underwent in vivo validation, revealing a half‐life approximately three times longer than Semaglutide and improved efficacy in glucose‐lowering and weight loss. The entire process, from design to validation, was concluded in a single cycle.

### 2.1 *De novo* GLP‐1RAs Design

Our *de novo* GLP‐1 RAs design pipeline consists of two primary stages: conserved sites analysis and ProteinMPNN *de novo* sequences design. Initially, we defined conserved sites or hotspot residues of GLP‐1RAs as critical functions for target recognition, binding, and activation of the GLP‐1R. 13 residue points (7H, 8Aib, 9E, 10G, 11T, 12F, 13T, 14S, 15D, 17S, 26K, 34R, 37G) were selected based on conservation analysis and referenced to Semaglutide. Semaglutide is a modified form of GLP‐1 (7–37), with a total length of 31 amino acids. Residues 7H, 9E, 10G, 11T, 12F, 13T, 14S, 15D, 17S are highly conserved in GLP‐1 analogues (Figure S1, Supporting Information) and play key roles in interacting with the GLP‐1 receptor's transmembrane core.^[^ ^10^ ^]^ 8Aib and 26K are two modification sites specific to Semaglutide. Residue 34K in GLP‐1 is substituted with arginine (34R) in Semaglutide. GLP‐1 exists in two active forms: GLP‐1(7‐36)‐NH2 and GLP‐1(7–37), Gly37 was used as the C‐terminal residue in our design.

We used the experimentally determined crystal structure of the Semaglutide‐GLP‐1R complex (PDB 7KI0) as the template for our design. We fixed 13 conserved sites and then used ProteinMPNN to design the remaining 18 sites, and generated 10,000 *de novo* GLP‐1RAs sequences. ProteinMPNN, a deep learning method for protein complex design, has been successfully deployed in previous studies to engineer proteins,^[^ ^5^, ^18^, ^20^, ^23^, ^34^, ^35^, ^36^ ^]^ such as ubiquitin,^[^ ^5^ ^]^ protein nanomaterials,^[^ ^34^ ^]^ myoglobin,^[^ ^20^ ^]^ TEV protease,^[^ ^23^ ^]^ peptide PROTAC drug,^[^ ^35^ ^]^ binders against Keap1/Nrf2.^[^ ^18^, ^36^ ^]^ In this study, we extend its application to peptide design.

### 2.2 Functional Screening

**Figure 2:**
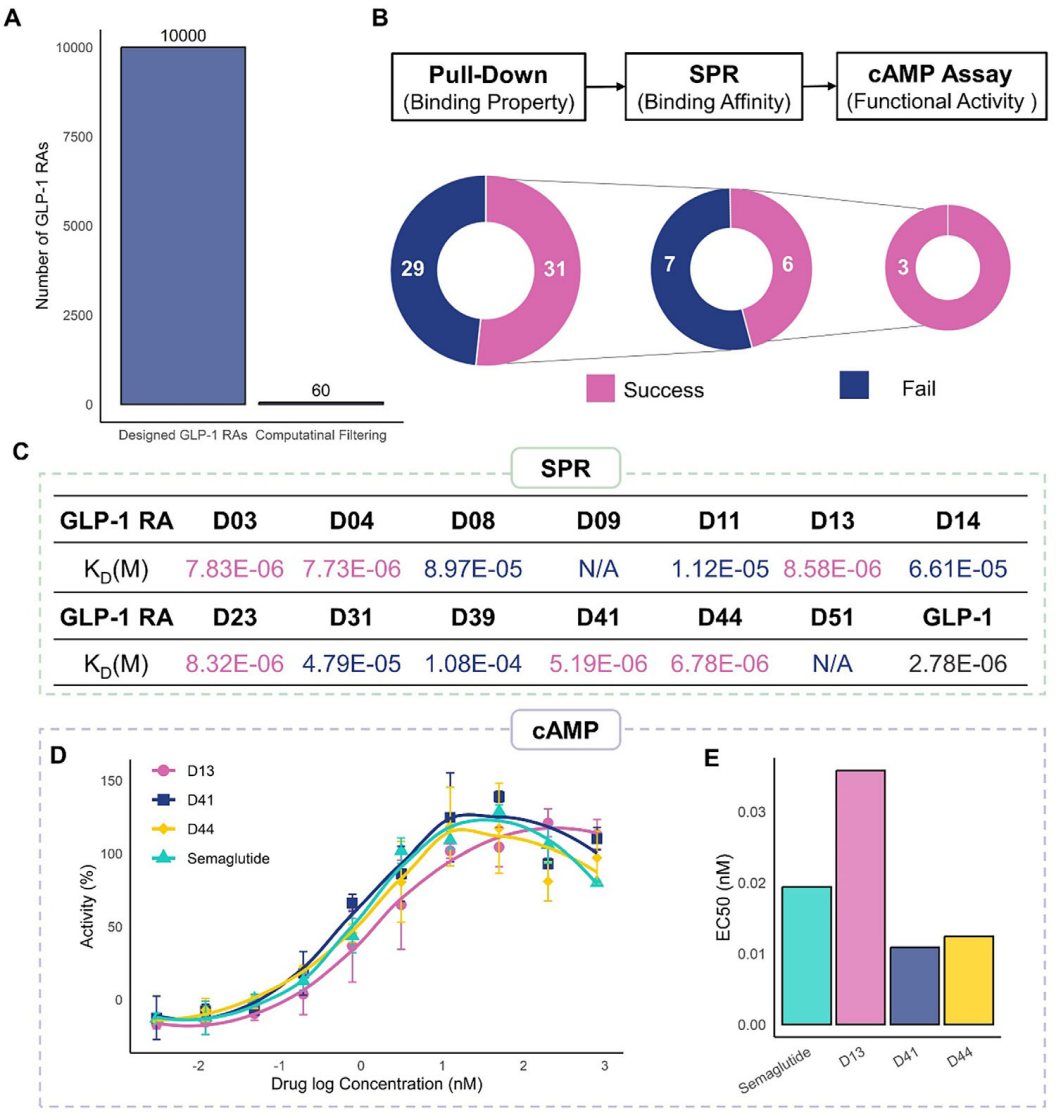
In vitro Functional Screening of GLP‐1RAs. A) the number of GLP‐1RAs after computational screenings. B) the number of successful and failed sequences at each in vitro screening step: GST pulldown assay, SPR, and cAMP accumulation assay. C) binding affinity of GLP‐1RAs assessed by SPR at six or eight concentrations in a single run. D) the cAMP dose‐response curves for GLP‐1RAs were measured in HEK‐293 cells expressing the cloned human GLP‐1R. Data represent means ± SD of two independent experiments performed in ten concentrations. E) the 50% activity concentration (EC_50_).

High‐throughput computational screening is an efficient method for narrowing down designed peptides, identifying promising drug candidates with desirable properties, and accelerating the drug discovery process. The 10,000 designed GLP‐1RAs underwent virtual screening based on stability, efficacy, and diversity criteria. Finally, 60 peptides with desirable properties, including extended biological half‐life, high binding affinity, and greater diversity compared to Semaglutide, were selected for in vitro and in vivo validation (**Figure** **2**A; Figures S2 and S7, Supporting Information).

Peptide drugs are susceptible to degradation by digestive enzymes, resulting in poor stability and a short plasma half‐life. Studies indicated that NEP‐24.11 can cleave GLP‐1RAs at six potential cleavage sites in the central and C‐terminal regions.^[^ ^37^ ^]^ To improve stability and extend the half‐life of the designed GLP‐1RAs, sequences containing these cleavage sites were filtered out. Additionally, factors such as helicity, isoelectric point, and hydrophobicity impact the peptide potency,^[^ ^9^, ^38^ ^]^ we further filter the designed GLP‐1RAs based on the net charge, hydrophobicity, and spatial aggregation propensity (SAP) score (see Methods section).

Binding ability to the receptor is closely associated with efficacy, and was evaluated using the folding ability of the complex structure and MD simulations. AlphaFold2 was used to predict the complex structures of the designed GLP‐1RA sequences and GLP‐1R. The folding capability of the designed GLP‐1RAs were assessed using several key parameters: The predicted Local Distance Difference Test (pLDDT) scores, which evaluate the quality of the predicted protein structure; the TM‐score and Root Mean Square Deviation (RMSD), which assess how well the designed structure aligns with the native structure; and the interface pAE, which evaluates the confidence in the interactions at the interface between GLP‐1RA and GLP‐1R in the complex. MD simulations provided insights into the binding affinity, which measures the binding strength between GLP‐1RA and GLP‐1R.

Sequence diversity was considered from two perspectives. First, diversity relative to the marketed drug was incorporated to avoid potential intellectual property conflicts. The similarity between designed sequences and the approved drug remained below the threshold defined by relevant patents. Patent avoidance is a critical consideration for pharmaceutical companies. Second, diversity among the designed peptides was incorporated to increase the likelihood of experimental success. More variability of designed sequences could cover a broader range of the sequence space landscape. This strategy increases the likelihood of identifying functional candidates. If one sequence fails in experimental validation, a highly similar sequence is likely to encounter the same issue. Phylogenetic trees were generated to categorize sequences, and a single representative sequence was selected in each phylogenetic cluster. Finally, a set of 60 unique GLP‐1RA sequences was selected for subsequent experimental evaluation.

### 2.3 In Vitro Experimental Validation

We performed in vitro functional screening of 60 unique GLP‐1RA sequences using a combination of GST pulldown assay, surface plasmon resonance (SPR), and intracellular cyclic adenosine monophosphate cAMP accumulation assay (Figure 2B; Figure S2, Supporting Information). The GST pulldown assay served as an effective preliminary screening, revealing 31 sequences that could bind to GLP‐1R. Subsequent SPR analysis further evaluated the binding affinity of these sequences, with 6 GLP‐1RAs demonstrating binding affinities comparable to Semaglutide. Finally, the cAMP assay assessed the functional activation of GLP‐1R, revealing that three GLP‐1RAs (D13, D41, and D44) could activate cAMP signaling. The number of successful and failed GLP‐1RAs at each screening step is summarized in Figure 2B.

The GST pulldown assay qualitatively evaluated the binding capability of GST‐peptide fusion proteins to GLP‐1R, providing a qualitative measure of receptor interaction. SDS‐PAGE images were shown in Figures S4 and S5 (Supporting Information), and Semaglutide‐GLP‐1R was used as a positive control. This assay identified 31 GLP‐1RAs capable of binding to GLP‐1R, while the remaining 29 sequences failed to show interaction (Figure 2B and **Table** **1**).

**Table 1:** The GLP‐1 RAs at each in vitro and in vivo step.

ID	Sequence Recovery	Biosynthesis					Chemical Synthesis	
		Pulldown		SPR		cAMP	PK	PD
		Label	Relative Expression	KD [m]	Label	EC50 [nm]	T1/2 [h]	Indication
Semaglutide	1	Success		2.78E‐06	Success	0.019	8.17±0.29	Diabetes, Obesity
D02	0.77	Success	0.95					
D03	0.68	Success	0.86	7.83E‐06	Success			
D04	0.71	Success	0.91	7.73E‐06	Success			
D05	0.68	Fail						
D06	0.71	Fail						
D07	0.71	Fail						
D08	0.74	Success	0.77	8.97E‐05	Fail			
D09	0.71	Success	0.84	NA	Fail			
D10	0.74	Fail						
D11	0.68	Success	0.80	1.12E‐05	Fail			
D12	0.68	Fail						
D13	0.81	Success	0.87	8.58E‐06	Success	0.036	19.86±1.55	Diabetes, Obesity
D14	0.74	Success	0.85	6.61E‐05	Fail			
D15	0.74	Fail						
D16	0.71	Success	0.79					
D17	0.74	Fail						
D18	0.71	Fail						
D19	0.71	Success	0.67					
D20	0.74	Success	0.98					
D21	0.68	Success	0.86					
D22	0.74	Success	0.80					
D23	0.71	Success	1.11	8.32E‐06	Success			
D24	0.71	Success	0.88					
D25	0.74	Success	0.93					
D26	0.74	Fail						
D27	0.71	Fail						
D28	0.77	Fail						
D29	0.65	Fail						
D30	0.65	Fail						
D31	0.71	Success	0.87	4.79E‐05	Fail			
D32	0.74	Fail						
D33	0.71	Success	0.82					
D34	0.68	Fail						
D35	0.71	Success	0.65					
D36	0.68	Fail						
D37	0.74	Success	0.74					
D38	0.71	Fail						
D39	0.71	Success	0.72	1.08E‐04	Fail			
D40	0.65	Fail						
D41	0.74	Success	0.71	5.19E‐06	Success	0.011	23.16±3.69	Diabetes, Obesity
D42	0.68	Success	0.72					
D43	0.74	Fail						
D44	0.68	Success	0.76	6.78E‐06	Success	0.012	4.19±0.37	Diabetes, Obesity
D45	0.68	Fail						
D46	0.65	Fail						
D47	0.74	Fail						
D48	0.74	Fail						
D49	0.74	Success	0.70					
D50	0.68	Success	0.63					
D51	0.68	Success	0.73	NA	Fail			
D52	0.74	Fail						
D53	0.71	Fail						
D54	0.68	Fail						
D55	0.65	Fail						
D56	0.77	Success	0.73					
D57	0.74	Success	0.55					
D58	0.68	Fail						
D59	0.74	Success	0.65					
D60	0.71	Success	0.77					
D61	0.71	Fail						

SPR was utilized as the next screening step to quantitatively assess the binding affinity between the GST‐fusion peptides and GLP‐1R. Of the 31 GLP‐1RAs that passed the GST pulldown assay, 13 underwent SPR analysis (Figure 2B and Table 1). The dissociation constant (K_D_) for GLP‐1 (Semaglutide peptide) binding to GLP‐1R was 2.78 × 10^−6^ m. Among these, 6 GLP‐1RAs (D03, D04, D13, D23, D41, and D44) exhibited binding affinities comparable to GLP‐1, with K_D_ values on the order of 10^−6^ (Figure 2C; Figure S6, Supporting Information).

cAMP accumulation assay evaluated the functional activity of the GLP‐1RAs by measuring their ability to elicit cAMP signaling, a key downstream response of GLP‐1R activation. Among the six GLP‐1RAs that passed the SPR analysis, three (D13, D41, and D44) underwent cAMP accumulation assay (Figure 2D and Table 1). D41 and D44 exhibited superior cAMP signaling, with half‐maximal effective concentrations (EC_50_) of 0.011 and 0.012 nm, respectively, compared to Semaglutide's 0.019 nm (Figure 2E). D13, with an EC_50_ of 0.036 nm, demonstrated similar cAMP signaling. These results indicated that all three GLP‐1RAs could effectively activate intracellular cAMP signaling.

GST pulldown assay, SPR assay, and intracellular cAMP accumulation assay identified three GLP‐1RA candidates (D13, D41, D44) with strong receptor binding and effective functional activity, making them suitable for further in vivo experimental evaluation.

### 2.4 Extended Half‐Life of GLP‐1RAs in Pharmacokinetics (PK)

**Figure 3:**
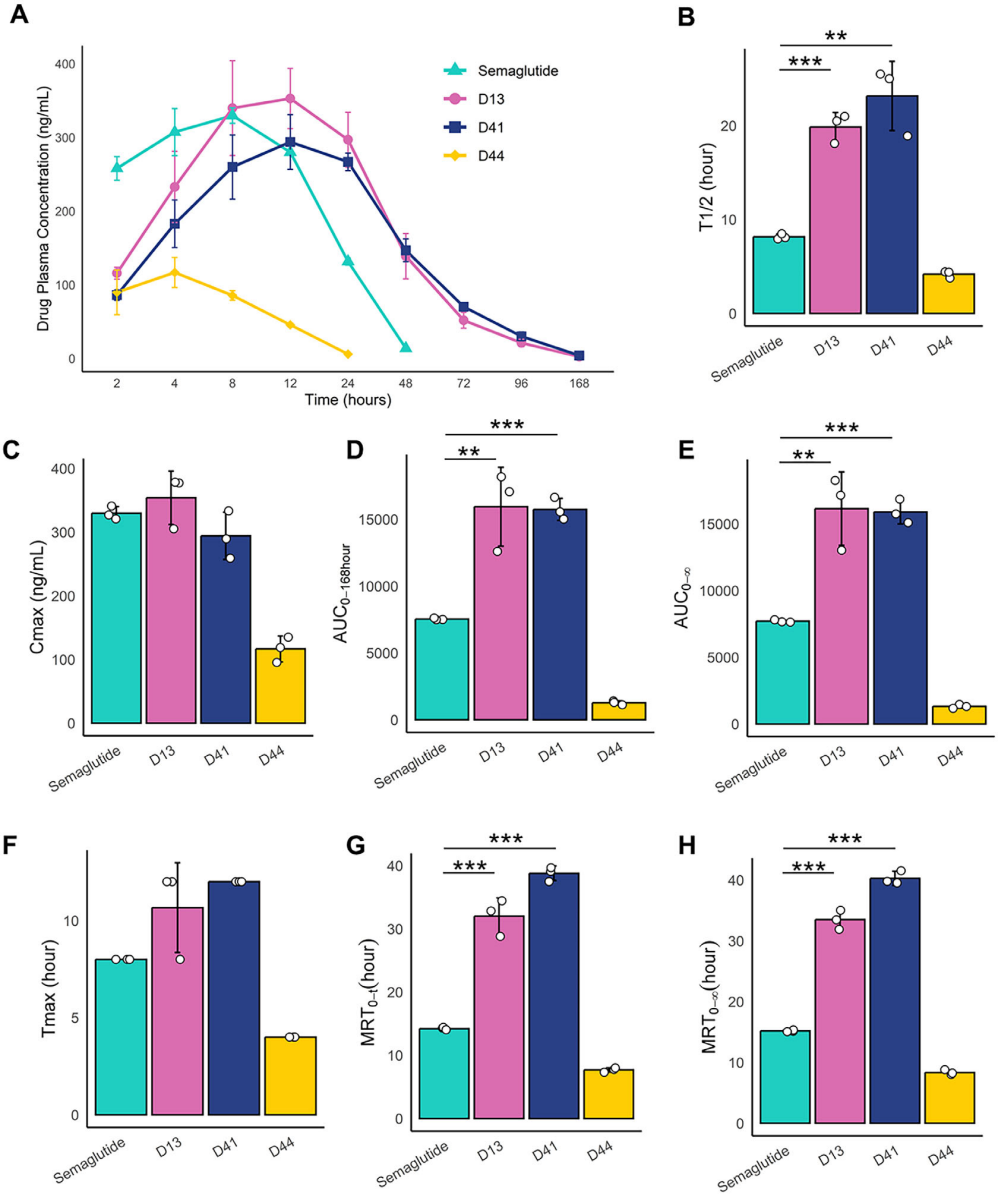
The PK result in rates (Mean ± SD, *n*=3). A) The drug plasma concentration‐time curve for Semaglutide (0.05 mg kg^−1^), D13 (0.05 mg kg^−1^), D41 (0.05 mg kg^−1^), and D44 (0.05 mg kg^−1^). B) half‐life (T_1/2_). C) the maximum plasma concentration (C_max_). D,E) the area under the concentration‐time curve of AUC_0‐168 hours_ and AUC_0‐∞_. F) time to Maximum Concentration (T_max_). G,H) The mean residence time of MRT_0‐168hours_ and MRT_0‐∞_. Data were analyzed with the two‐sided Student's unpaired *t* test. ^*^ *p* < 0.05, ^**^ *p* < 0.01, ^***^ *p* < 0.001.

Pharmacokinetics (PK) refers to the study of a drug's absorption, distribution, metabolism, and excretion (ADME) within the body. Among the three GLP‐1RA candidates, D13 and D41 exhibited significantly longer half‐lives compared to Semaglutide in SD rats. Specifically, D13 exhibited a half‐life (T_1/2_) of 19.86 h, ≈2.43 times longer than Semaglutide's 8.17 h. Similarly, D41 displayed a prolonged T_1/2_ of 23.16 h, 2.83 times that of Semaglutide (**Figure** **3**A,B; Table S4, Supporting Information).

D13 also achieved a maximum plasma concentration (C_max_) of 353.96 ng mL^−1^, which is higher than Semaglutide's C_max_ of 329.82 ng mL^−1^ (Figure 3C). The area under the concentration‐time curve (AUC_0‐t_), representing the total drug exposure over 0–168 h, was ≈2.1 times (D13 is 15939.88 h*ng mL^−1^ and D41 is 15737.19 h*ng mL^−1^) higher than that of Semaglutide's 7538.16 h*ng mL^−1^ (Figure 3D). AUC_0‐∞_, representing the total drug exposure over 0‐infinite time, was also higher for D13 and D41 compared to Semaglutide (Figure 3E).

The time to maximum concentration (T_max_) was 10.67 h for D13 and 12 h for D41, both longer than Semaglutide's 8.17 h (Figure 3F). The mean residence time (MRT_0‐t_), indicating the average duration for the drug to remain in the body before elimination within 0–168 h, was 32.02 h for D13 and 38.79 h for D41, ≈2.3 and 2.7 times that of Semaglutide (14.21 h) (Figure 3G). Similarly, MRT_0‐∞_, representing the average duration for the drug to remain in the body before complete elimination, was higher for both D13 and D41 compared to Semaglutide (Figure 3H).

The D13, D41, and D44 are conjugated with a C20 fatty acid chain, whereas Semaglutide contains a C18 fatty acid chain (Table S1, Supporting Information). According to a study by Novo Nordisk,^[^ ^39^ ^]^ Semaglutide with a C18 fatty acid chain exhibits a half‐life of ≈7–8 h in rats, consistent with our observations. The Semaglutide analogue containing a C20 fatty acid chain demonstrates extended half‐lives of ≈9–10 h. Notably, our designed D13 and D41, both conjugated with C20 fatty acid chains, exhibit significantly longer half‐lives of 19.86 and 23.16 h, respectively. While the fatty acid chain contributes to increased half‐life, our data also suggest that the peptide sequence plays a major role in extended half‐life.

### 2.5 Lower Blood Glucose Levels of GLP‐1RAs in Diabetic Nephropathy

The hypoglycemic effect of the GLP‐1RAs was evaluated in the diabetic db/db mouse model. GLP‐1RAs were administered subcutaneously either as a single dose or as multiple doses for 28 days (10 nmol/kg administered once daily).^[^ ^29^ ^]^ The hypoglycemic effect of a single dose focused on the acute reduction in blood glucose levels. This analysis provides valuable insights into the time‐effect relationship, which is compared with the PK results. The hypoglycemic effect of multiple doses primarily examines the long‐term effects of glucose‐lowering and the cumulative impacts.

**Figure 4:**
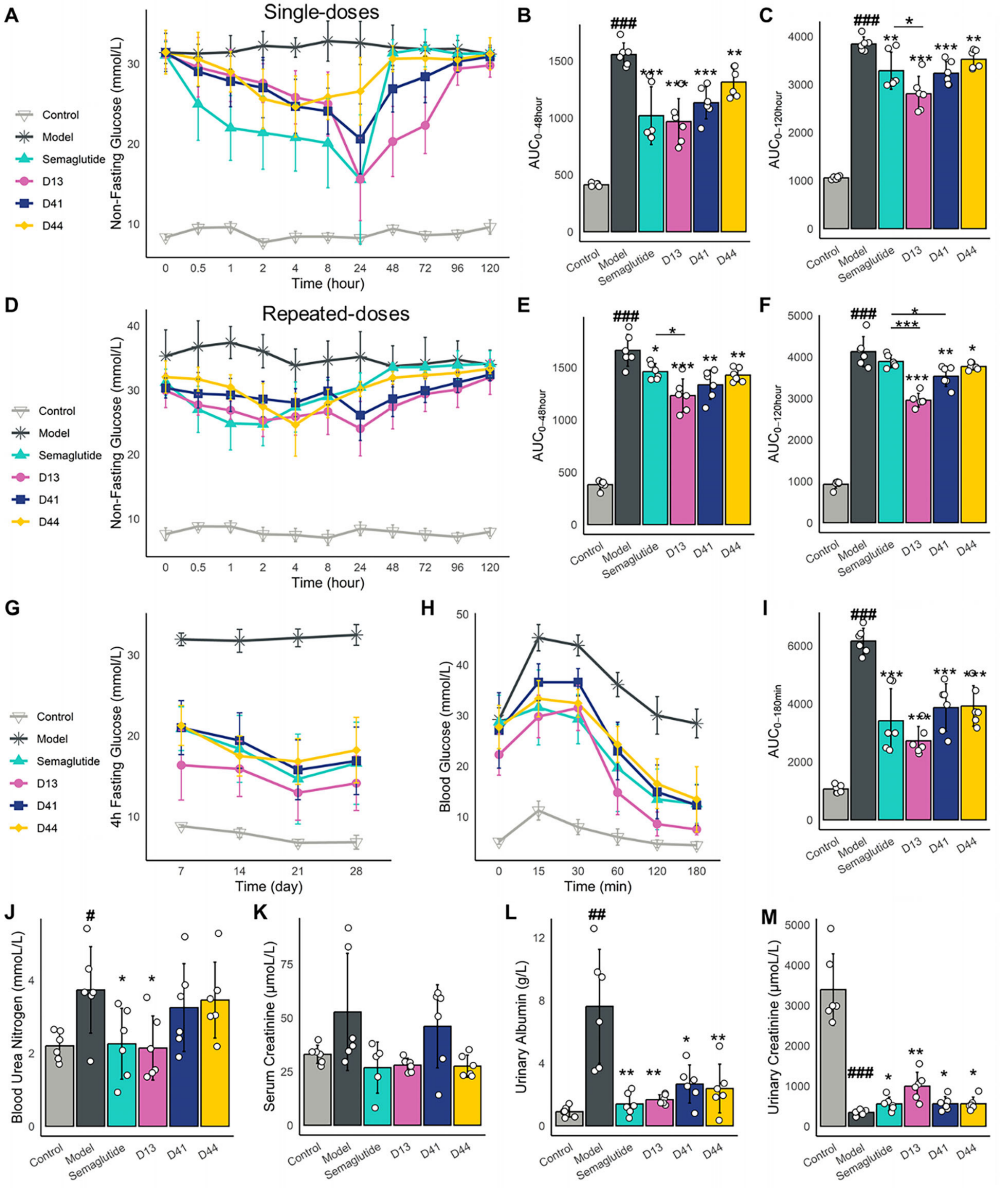
Glucose‐lowering of GLP‐1RAs in the Diabetic model (Mean ± SD, *n*=6). The db/db mice were treated with GLP‐1RAs and Semaglutide at 10 nmol kg^−1^ for a single dose A–C) and multiple doses D–M). (A) the non‐fasting glucose‐time curve after a single dose. (B) the area under the non‐fasting glucose‐time curve (AUC) after a single dose within 0–48 h (AUC_0‐48 hour_) and (C) within 0–120 h (AUC_0‐120 hour_). (D) the non‐fasting glucose‐time curve after multiple doses for 28 days. (E) The area under the non‐fasting glucose‐time curve after multiple doses within 0–48 h (AUC_0‐48 hour_) and (F) within 0–120 h (AUC_0‐120 hour_). (G) 4‐h fasting blood glucose during 28 days continuous administration period. (H) The intraperitoneal glucose tolerance test (iPGTT) after multiple doses. (I) The area under the iPGTT curve (AUC) with 0–180min after multiple doses. (J–M) kidney injury biomarkers of blood urea nitrogen (BUN), serum creatinine (Scr), urinary albumin levels, and urinary creatinine levels after multiple administrations. Data were analyzed with the two‐sided Student's unpaired *t* test. # Compared with normal group, ^*^compared with model group. ^#^ *p* < 0.05, ^##^ *p* < 0.01, ^###^ *p* < 0.001, ^*^ *p* < 0.05, ^**^ *p* < 0.01, ^***^ *p* < 0.001.

We evaluate the acute glucose‐lowering effect of a single dose of D13, D41, and D44 in the diabetic db/db mouse model. Non‐fasting glucose level was monitored at timepoints of 0, 0.5, 1, 2, 4, 8, 24, 48, 72, 96, 120 h. We observed that D13, D41, D44, and Semaglutide produced significant glucose‐lowering effects compared to the model group (**Figure** **4**A–C).

In the Semaglutide (10 nmol kg^−1^) group, non‐fasting blood glucose levels began to decrease at 0.5 h after administration and remained reduced until 24 h. Blood glucose levels reached their lowest point between 8 and 24 h and returned to baseline (model group levels) by 48 h (Figure 4A,B). In the D13 group, non‐fasting blood glucose levels began decreasing at 0.5 h post‐administration and persisted for up to 96 h. Blood glucose levels reached their lowest point between 24 and 72 h, returning to near baseline levels by 96 h (Figure 4A,C). The lowest blood glucose level in the D13 group (15.53 ± 8.06 mmol L^−1^) was comparable to that of the Semaglutide group (15.57 ± 5.16 mmol L^−1^). Notably, non‐fasting glucose levels after 24 h of administration in the D13 group were significantly reduced compared with the Semaglutide group at three time points, with P‐values of 0.0002, 0.0002, 0.1694, 0.0436 at 48, 72, 96, 120 h, respectively. The D41 group has a similar effect to the D13 group, blood glucose levels reached their lowest point between 8 and 48 h (20.6 ± 4.37 mmol L^−1^), returning to near baseline levels by 96 h.

D13 exhibits a lower drug plasma concentration than Semaglutide within the first 8 hours, which likely accounts for its weaker glucose‐lowering efficacy during this early period (0–8h) (Figure 3A). However, from 8 to 24 h, the drug plasma concentration of Semaglutide rapidly declines, the drug plasma concentration of D13 continues to increase, and maintains a higher concentration compared to Semaglutide. These findings were consistent with the PK data, suggesting that D13 may provide prolonged glucose control compared to Semaglutide, making it a promising candidate for sustained blood glucose management in T2DM.

To evaluate the long‐term glucose‐lowering, D13, D41, and D44 were administered subcutaneously over 28 days (every day for 28 days) in the diabetic db/db mouse model. At the end of the 28 days, non‐fasting glucose was monitored at timepoints of 0, 0.5, 1, 2, 4, 8, 24, 48, 72, 96, and 120 h. In the D13 group (10 nmol kg^−1^), non‐fasting blood glucose levels in db/db mice began decreasing at 0.5 h post‐administration and persisted for up to 96 h. The lowest blood glucose level in the D13 group (24.00 ± 4.24mmol L^−1^) was lower than that of the Semaglutide group (24.68 ± 3.34 mmol L^−1^). Notably, the blood glucose after 8 h in the D13 group was significantly reduced compared with the Semaglutide group at four time points, with P‐values of 0.0081, 0.0016, 0.0047, 0.028 at 24, 48, 72, 96 h, respectively. (Figure 4D–F). This may be attributed to the dose accumulation effect of D13, given its longer half‐life and higher C_max_. A similar effect was observed in the D41 group, where the blood glucose was significantly lower than the Semaglutide group after 8 h, with P‐values of 0.04, 0.0017, 0.0012, 0.04 at 24, 48, 72, 96 h, respectively (Figure 4D–F).

After 28 days of continuous administration, 4‐hour fasting blood glucose levels were measured once a week. The 4‐hour fasting blood glucose of the D13 group was also lower than that in the Semaglutide group, with no statistical significance (Figure 4G).

The intraperitoneal glucose tolerance test (iPGTT) was performed 120 h after multiple doses. Blood glucose was measured at 10, 30, 60, 120, and 180 min after glucose loading. Semaglutide, D13, D41, and D44 demonstrated improved glucose tolerance during 0–180 min (Figure 4H). D13 exhibited slightly greater efficacy than Semaglutide, though the difference was not statistically significant. The AUC_0‐180min_ for D13 (2725.75±491.03 mmol L*min^−1^) was lower than that of Semaglutide's 3414±1105.75 mmol/L*min, indicating enhanced glucose tolerance (Figure 4I).

Further investigation into renal protection revealed that Semaglutide and D13 significantly reduced markers of kidney injury in *db/db* mice, including blood urea nitrogen (BUN), serum creatinine (Scr), and urinary albumin levels, while concurrently increasing urinary creatinine levels (Figure 4J–M).

### 2.6 Weight Loss of GLP‐1RAs in Obesity

**Figure 5:**
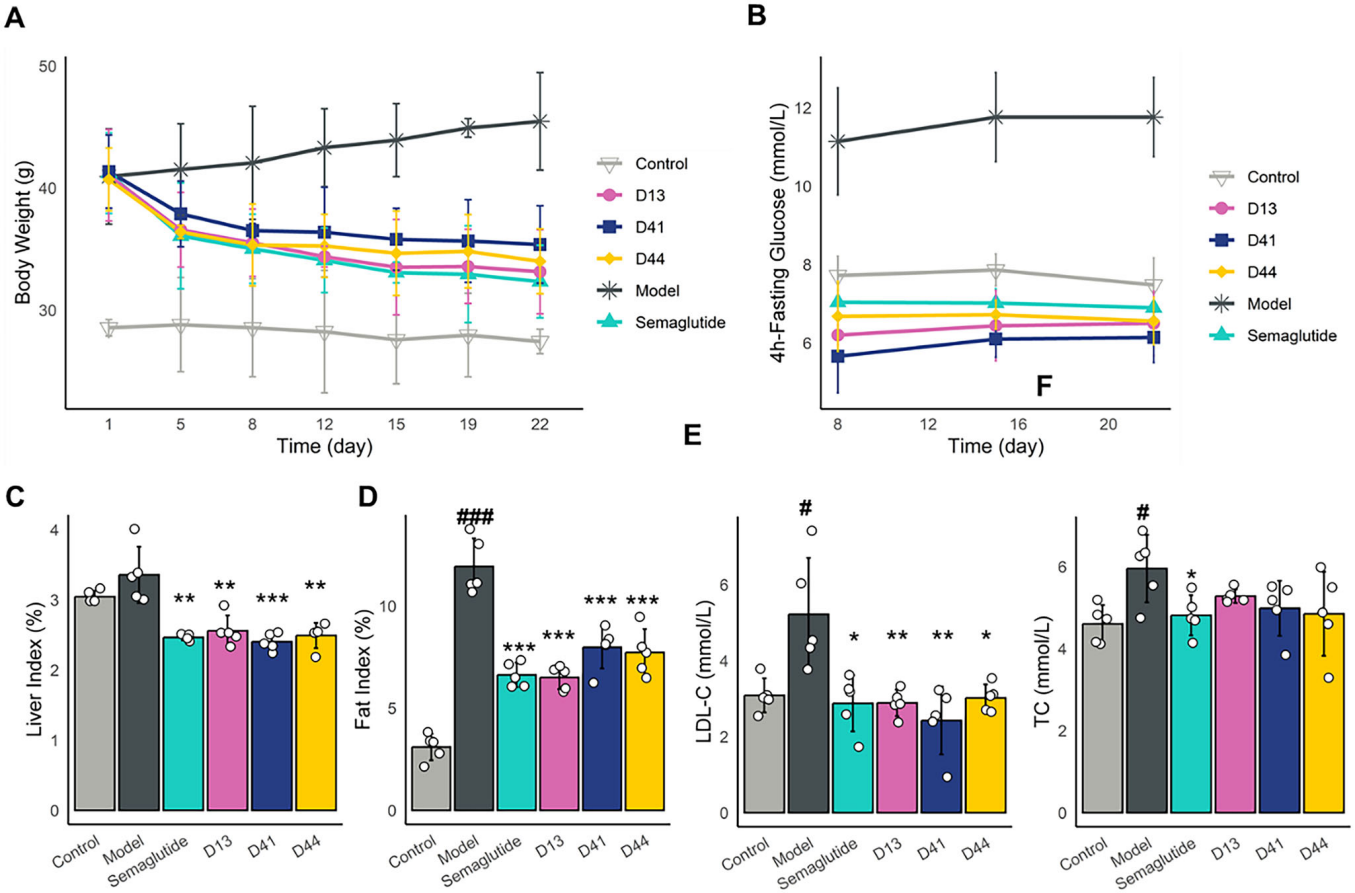
Weight loss of GLP‐1RAs in the Obesity model (Mean ± SD, *n*=5). The DIO mice were treated with GLP‐1RAs and Semaglutide from 0 to 22 days at 10 nmol kg^−1^, measuring A) body weight and B) 4‐h fasting blood glucose. C) Liver index. D) fat index. E) serum LDL‐C. F) serum TC. Data were analyzed with the two‐sided Student's unpaired *t* test. # Compared with normal group, *compared with model group, ^#^ *p* < 0.05, ^##^ *p* < 0.01, ^###^ *p* < 0.001, ^*^ *p* < 0.05, ^**^ *p* < 0.01, ^***^ *p* < 0.001.

To assess the weight loss efficacy of GLP‐1RAs, diet‐induced obese (DIO) mice were administered GLP‐1RAs and Semaglutide at 10 nmol kg^−1^.^[^ ^40^ ^]^ DIO mice exhibited a decrease in body weight during treatment with D13, D41, and D44. D13, D41, and D44 resulted in body weight reductions of 19.25%, 14.42%, and 16.46%, respectively, while semaglutide led to a 21.54% reduction (**Figure** **5**A). The weight loss efficacy of D13 was comparable to that of semaglutide, with no significant difference between the two treatments. These data demonstrate the effectiveness of D13 in reducing body weight in DIO mice.

The 4‐hour fasting blood glucose in DIO mice decreased in Semaglutide, D13, D41, and D44 groups, with D13 and D44 showing slightly lower levels than the Semaglutide group, though the difference was not statistically significant (P‐value >0.5) (Figure 5B). Semaglutide, D13, D41, and D44 groups exhibited reduced liver and fat index (Figure 5C,D). Serum LDL‐C in DIO mice was significantly reduced in the Semaglutide, D13, D41, and D44 (Figure 5E). While serum TC showed a decreasing trend in the Semaglutide, D13, D41, and D44, the difference was not statistically significant (Figure 5F).

### 2.7 Sequence and Structure Analysis of GLP‐1RAs

To gain insights into the sequence and structural characteristics of GLP‐1RAs associated with longer half‐life and improved efficacy, we performed a comprehensive analysis of sequence, structure, and evolutionary relationships. Our goal was to offer additional design principles for developing more effective GLP‐1RAs with prolonged half‐life and improved efficacy.

**Figure 6:**
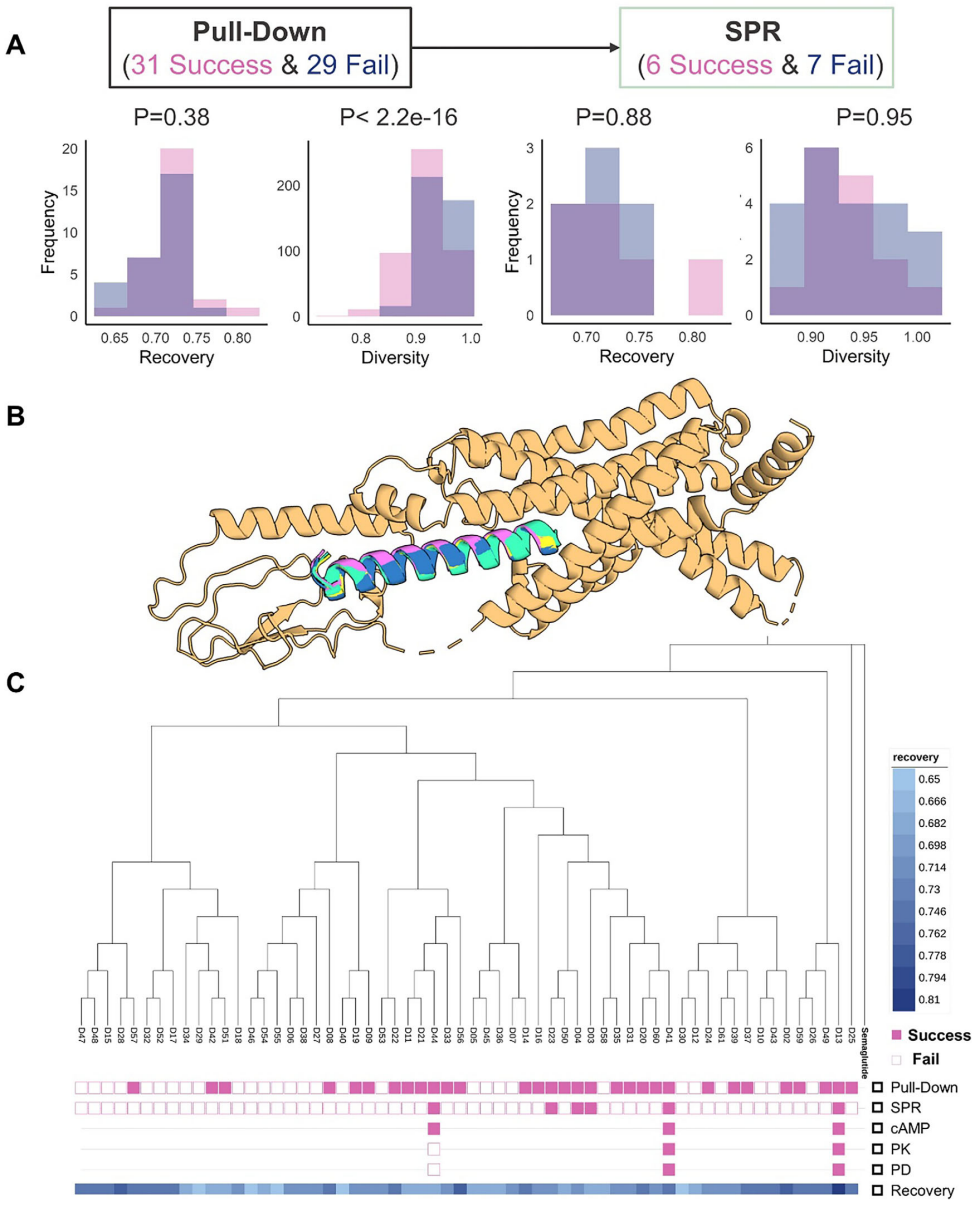
Sequence, structural, and evolutionary analyses of GLP‐1RAs. A) sequence analysis: Comparison of recovery and diversity distributions between successful and failed GLP‐1RAs using GST pulldown and SPR assays. B) structural analysis: AlphaFold2‐predicted complex structure of GLP‐1RA and GLP‐1R. C) evolutionary analysis: Phylogenetic tree of 60 GLP‐1RAs constructed to explore evolutionary relationships.

We first examined sequence differences between successful and failed GLP‐1RAs in GST pulldown and SPR assays. Interestingly, no significant differences were observed in the recovery and diversity distributions between successful and failed groups (**Figure** **6**A). These metrics, which are critical in AI‐based protein sequence design, suggest that factors beyond sequence recovery and diversity influence the success of GLP‐1RAs.

To further explore the structural basis of GLP‐1RA, we used AlphaFold2 to predict the complex structure of GLP‐1RA and GLP‐1R (Figure 6B). We used the fixed‐backbone design strategy, leading to the designed peptides characterized by a conserved α‐helix structure. While the α‐helix structure is common in many GLP‐1R agonists, recent studies—such as the Evobind platform—have demonstrated that cyclic peptides could activate GLP‐1R and GCGR.^[^ ^12^ ^]^ This suggests that the α‐helix structure is not universally conserved, and its necessity for GLP‐1R activation warrants further investigation.

Furthermore, to understand the evolutionary relationships among GLP‐1RAs, we constructed a phylogenetic tree comprising 60 GLP‐1RAs (Figure 6C; Figure S8, Supporting Information). Both successful and failed GLP‐1RAs were broadly distributed throughout the tree, indicating no distinct evolutionary clustering between the two groups. Notably, the root of the phylogenetic tree is Semaglutide. D13, which exhibits a longer half‐life and superior efficacy, is positioned close to Semaglutide in the tree, having the smallest phylogenetic distance of 0.19 (Figure S9, Supporting Information) and sequence similarity (recovery = 0.81). Additionally, D25 (recovery = 0.74), another GLP‐1RA not yet experimentally validated, is topologically proximity to Semaglutide. Although D25 was not included in this round of experimental validation, its sequence recovery and structural features make it a promising candidate for further exploration.

## 3 Conclusion

Peptides have emerged as a unique drug class for treating a wide range of diseases, including diabetes, cancer, and central nervous system disorders. However, designing biotechnologically essential peptides requires expensive, time‐consuming, and extensive validation. Deep learning‐based protein design method has proven to be a powerful tool for generating novel proteins with desired properties.^[^ ^41^ ^]^ Here, we introduce a deep learning‐based peptide design pipeline that combines deep learning protein sequence design and efficient functional screening, enabling the successful design of novel GLP‐1RAs with extended half‐life and enhanced efficacy compared to Semaglutide. For GLP‐1RAs design, 10,000 designed sequences were subsequently subjected to functional screening based on stability, efficacy, and diversity, resulting in a much‐reduced number of peptide candidates, ≈60 GLP‐1RAs for experimental evaluation.

Using the GST pulldown assay, we identified 31 GLP‐1RAs that interact with GLP‐1R. From these candidates, 13 were selected for the SPR assay, of which 6 exhibited binding affinities comparable to Semaglutide. Sequentially, 3 GLP‐1RAs (D13, D41, and D44) underwent evaluation in the cAMP assay. The EC_50_ rank was: D41 (0.011 nm) > D44 (0.012 nm) > Semaglutide (0.019 nm) > D13 (0.036 nm). As only a subset of candidates was validated, additional GLP‐1RAs may also be capable of activating the cAMP second messenger signaling pathway.

In Pharmacokinetics (PK), both D13 and D41 showed significantly improved pharmacokinetic properties relative to Semaglutide, including a longer half‐life, extended T_max_, and enhanced C_max_. These improvements suggest that D13 and D41 may offer prolonged therapeutic effects. The half‐life (T_1/2_) ranking was: D41 (23.16 h) > D13 (19.86 h) > Semaglutide (8.17 h) > D44 (4.19 h). The C_max_ ranking was: D13 (353.96 ng mL^−1^) > Semaglutide (329.82 ng mL^−1^) > D44 (116.60 ng mL^−1^) > D44 (116.60 ng mL^−1^). Notably, the in vivo PK findings did not fully correlate with the cAMP assay results, as D44 exhibited the poorest PK performance despite favorable cAMP activity.

In diabetic mice with simple administration and multiple administration, the hypoglycemic effects of D13 and D41 were notably prolonged compared to Semaglutide. These findings are consistent with the observed pharmacokinetic data, further supporting that an extended half‐life contributes to more durable blood glucose control. D13 demonstrated significantly lower blood glucose levels than Semaglutide in both single‐dose and multiple‐dose regimens. Weight reduction with D13 was slightly lower than Semaglutide, but the difference was not statistically significant. Efficacy outcomes varied across disease models, indicating that factors such as disease subtype might influence the treatment response of the same drug. This highlights the importance of designing GLP‐1RAs candidates tailored for specific diseases.

Our AI‐powered high‐throughput protein design, functional screening, and experiment validation pipelines provide a promising way for improving the stability and efficacy of biotechnologically important peptide drugs. This pipeline has been evaluated only in GLP‐1RAs. We are now designing GLP‐1R/GCGR/GIPR triple agonists and hope that the in vitro experiment will surpass the performance of Retatrutide.^[^ ^42^ ^]^

## 4 Experimental Section

### Design *De novo* GLP‐1RAs

For conserved site analysis, the sequences of GLP‐1RAs marked drugs were collected, including GLP‐1, Exenatide, Lixisenatide, Liraglutide, Semaglutide, and Albiglutide. Clustal X was used to identify highly conserved sites of GLP‐1RAs in multiple sequence alignments.

The conserved sites and crystal structure of the Semaglutide‐GLP‐1R complex (PDB 7KI0) were used as inputs for ProteinMPNN. To generate *de novo* GLP‐1RAs, with the GLP‐1R chain being fixed and the Semaglutide chain being selected for optimized sequences. ProteinMPNN was used to generate 10,000 sequences using the default parameters, including a sampling temperature of 0.1.

### Computational Screening of Designed GLP‐1RAs—Stability

Using ProteinMPNN, a total of 10,000 GLP‐1RA sequences was generated. Semaglutide, the initial template for the design, consists of 31 amino acids. Of these, 13 conserved residues were fixed, while the remaining 18 amino acids were designed by ProteinMPNN. Due to the limited design space and conserved structural constraints (α‐helix), a substantial number of repetitive sequences were generated during this process. After eliminating redundant sequences, 1,443 unique sequences were obtained.

In vitro studies revealed that the enzyme NEP‐24.11 can cleave GLP‐1 at six potential sites in the central and C‐terminal regions, with particular vulnerability between Glu27‐Phe28 and Trp31‐Leu32, along with additional cleavage sites at Asp15‐Val16, Ser18‐Tyr19, Tyr19‐Leu20, and Phe28‐Ile29. To improve stability and extend the half‐life of the designed GLP‐1RAs, sequences containing these cleavage sites was filtered out.

The net charge, hydrophobicity, and SAP were used to estimate the protein solubility. The net charge of a protein plays a crucial role in its solubility. SAP was calculated for each residue based on a combination of solvent accessibility and hydrophobicity. Hydrophobicity primarily controls the exposure of non‐polar residues on the protein surface.

### Computational Screening of Designed GLP‐1RAs—Efficacy

AlphaFold2 was employed to predict the complex structures of designed GLP‐1RA sequences and GLP‐1R. The best rank structure of each complex was further calculated TM‐score, RMSD, pLDDT, and interface pAE. TM‐score between the designed complex structure (GLP‐1 RA and GLP‐1R) and the native complex structure (Semaglutide and GLP‐1R); RMSD between the designed GLP‐1RA structure and Semaglutide native structures; pLDDT for the designed GLP‐1RA structures; The average pAE of interchain residue pairs (interface pAE) of GLP‐1 RA and GLP‐1R complex. The critical thresholds for these parameters were derived from the native complex structure between Semaglutide and GLP‐1R.

### Computational Screening of Designed GLP‐1RAs—Molecular Dynamics (MD) Simulations

MD simulations were conducted with Amber22 to assess the binding affinity of GLP‐1RAs and GLP‐1R. The ff03CMAP force field was used for MD simulation.^[^ ^43^ ^]^ The solvent model used was TIP3P, one of the most commonly used solvent models for protein simulations. First, energy minimization, heating, and equilibrium of the system were carried out. The energy of the system was minimized by the steepest descent method of 3000 steps and the conjugate gradient method of 3000 steps. After energy minimization, the system was heated from 0 to 310.15K in a time of 50 ps and then performed an energy balance of 100 ps at constant pressure and temperature of 310.15K. In the whole process, the long‐range electrostatic interaction was calculated by PME algorithm, and the covalent bonds of all hydrogen atoms were constrained by SHAKE algorithm. The cut‐off value for the van der Waals interaction and the short‐range electrostatic interaction was set at 10 Å. The final simulation process was carried out at NPT and temperature of 310.15K, and the simulation time was 20 ns. Binding affinity between GLP‐1RAs and GLP‐1R was calculated using MM/GBSA method based on MD trajectories.

### Computational Screening of Designed GLP‐1RAs—Diversity

Designed GLP‐1RAs were aligned using ClustalX 2.1. Phylogenetic trees were built using the maximum‐likelihood method implemented in MEGA (v10.2.2) employing the LG +F model with a maximum of 1000 rapid bootstraps. Obtained phylogenetic trees were visualized in iTOL (v6). To ensure sequence diversity, a single representative sequence was selected from each phylogenetic cluster.

### Study Design of In Vitro Experiment

For the pull‐down and SPR assay, peptides produced via biosynthesis in E. coli BL21 was used, which don't include modifications such as the substitution of position 8 with Aib and fatty acid chain at position 26. Since pulldown and SPR assay assess the binding affinity between GLP‐1RAs and GLP‐1R, modifications related to half‐life were not included during this preliminary screening stage. For cAMP and in vivo experiments, chemically synthesized peptides was used that incorporated both Aib and a fatty acid chain. Due to the high cost of chemical synthesis (approximately ¥5000 per peptide), only selected candidates from pulldown and SPR assay were synthesized with modifications for downstream cAMP and in vivo experiments. The GLP‐1RAs sequences for biosynthesis and chemical synthesis were shown in Table S1 (Supporting Information).

### Study Design of in vitro Experiment—GST Pulldown Assay

DNA coding sequences of GST‐Linker‐Peptides were cloned into PGEX‐4T‐1 (Cytiva, GE Healthcare) between BamHI and XhoI, respectively. The structure of GST‐Linker‐Peptides were shown in Figure S3 (Supporting Information). Constructs were transformed into *E. coli* BL21 (DE3) for GST‐peptide fusion protein expression. Human GLP‐1R protein (MedChemExpress LLC, HY‐P700468) were added to each GST‐fusion protein coupled beads and incubated at 4°C for 1 h. Two washes using equilibrate buffer were performed to remove residual GLP‐1R protein. Proteins binding to beads were eluted by 1% sodium dodecyl sulphonate. Samples were loaded on SDS‐PAGE and results were displayed by coomassi blue staining. GST‐Semaglutide was used as a positive control.

Of the 31 GLP‐1RAs that passed the GST pulldown assay, 5 peptides were excluded with relative expression values below 0.7. From the remaining 26 peptides, one representative peptide was selected in each phylogenetic cluster to ensure sequence diversity. Finally, 13 peptides were chosen for the SPR assay (Table 1).

(1) $$\mathrm{the}\;\mathrm{protein}\;\mathrm{relative}\;\mathrm{expression}=\frac{\mathrm{peptide}\;\mathrm{band}\;\mathrm{density}}{\mathrm{GLP}-1\;\mathrm{band}\;\mathrm{density}}$$

### Study Design of In Vitro Experiment—Surface Plasmon Resonance (SPR)

The binding affinity between GLP‐1R and GST‐fusion peptides was investigated by surface plasmon resonance on Biacore 8k system (Cytiva). Briefly, the CM5 sensor chip (Cytiva, 29149603) was used to measure the binding kinetics between GLP‐1R and GST‐fused peptides. Affinity K_D_ values were fitted by steady state affinity fitting models (1:1 binding model) using Biacore Insight Evaluation Software, and experimental data were analyzed using the Biacore Insight Evaluation Software. The detailed information of SPR was provided in Table S2 (Supporting Information). Purified GST protein was also loaded for kinetic analyses as a negative control. The SPR dose‐dependent saturation binding of GPL‐1 and GLP‐1R was tested at six or eight concentrations in a single run.

Among the six GLP‐1RAs (D03, D04, D13, D23, D41, and D44) that passed the SPR analysis, three (D13, D41 and D44) underwent cAMP accumulation assay. The selection was based on their recovery values. The recovery of D03, D04, D13, D23, D41, and D44 was 0.68, 0.71, 0.81, 0.71, 0.74, 0.68, respectively. D13 with the highest recovery was chosen, D44 has the lowest recovery, and D41 has an intermediate value.

### Study Design of In Vitro Experiment—Chemical Synthesis

D13, D41, and D44 used in cAMP and in vivo experiments were synthesized using standard Fmoc chemistry. All peptides were synthesized, purified, and characterized by Wuxi AppTec. Detailed characterization, including molecular weight and purity, was provided in Table S3 (Supporting Information). Semaglutide was obtained from Ozempic.

### Study Design of In Vitro Experiment—Human GLP‐1R Cell cAMP Assay

Intracellular cAMP accumulation was measured using the cAMP detection kit (Cisbio, 62AM4PEJ) based on the homogeneous time‐resolved fluorescence (HTRF) technology. Briefly, HEK293 cells stably expressing GLP‐1R were transferred to OptiPlate‐384 plate. Add GLP‐1RAs and Semaglutide were diluted with PBS buffer supplemented with 500 µm, and were incubated with cells for 30 min at room temperature. Time‐resolved FRET signals were measured on an EnVision (PerkinElmer) at 665 nm and 620 nm. All the dose‐response curve fits were analyzed with R using an equation of log (GLP‐1RAs) vs activity. Two independent experiments were performed in ten concentrations: 0.003, 0.012, 0.049, 0.195, 0.781, 3.125, 12.5, 50, 200, and 800nm. SPR and cAMP assay were measured across multiple concentrations, and these in vitro experiments were intended for preliminary screening purposes. Multiple independent validations will be conducted in vivo experiments.

### Study Design of In Vivo Experiment

Animals were cared for in accordance with the Guidelines and Principles of Laboratory Animal Care and the standard procedures established by the Medicilon Institutional Animal Care and Use Committee (approval number: IACUC‐2024‐6).

### Study Design of In Vivo Experiment—Study Design of Pharmacokinetics (PK)

Three SD male rats in each group were administered a single dose of 0.05 mg kg^−1^ of Semaglutide (Ozempic) and the GLP‐1RAs. Blood samples were collected at 10 post‐ administration time points: 2, 4, 8, 12, 24, 48, 72, 96, and 168h. Plasma samples were analyzed using LC‐MS/MS (Waters XEVO TQ‐XS), and pharmacokinetic parameters such as T_1/2_, C_max_, T_max_, AUC_0‐t_, and MRT_0‐t_ were calculated using WinNonlin. The sample preparation and detailed information of LC‐MS/MS are shown in Table S5 (Supporting Information).

### Study Design of In Vivo Experiment—Study Design of db/db Diabetic Nephropathy Models—Acute Glucose‐Lowering after Single Administration

Male db/db mice were randomly divided into five groups, with six mice per group: model group, Semaglutide group (10 nmol kg^−1^), D13 group (10 nmol kg^−1^), D41 group (10 nmol kg^−1^), and D44 group (10 nmol kg^−1^).^[^ ^40^ ^]^ Additionally, six db/m mice were included as the control group. Each animal was administered a single dose of the drug. Non‐fasting blood glucose levels were monitored at multiple time points post‐administration: 0, 0.5, 1, 2, 4, 8, 24, 48, 72, 96, and 120 h.

### Study Design of In Vivo Experiment—Study Design of db/db Diabetic Nephropathy Models—Long‐Term Effects of Glucose‐Lowering after Single Administration

Two weeks after completing the single‐dose administration study, once the fasting blood glucose of the db/db mice (measured after 4 h of fasting) returned to the level of the model group, a 4‐week continuous administration was conducted. During the continuous administration period, fasting blood glucose levels were measured once a week. On day 28, non‐fasting blood glucose levels were measured at 0, 0.5, 1, 2, 4, 8, 24, and 48 h. Afterward, the intraperitoneal glucose tolerance test (iPGTT) was performed after overnight fasting. Urine samples were collected for the analysis of urine albumin and urine creatinine content. The next day (after overnight fasting), animals were deeply anesthetized with CO_2_, and blood samples were collected for the measurement of glycated hemoglobin, creatinine, and blood urea nitrogen levels. Both kidneys were excised, weighed, and the kidney to body weight ratio was calculated.

### Study Design of In Vivo Experiment—Study Design of DIO Obesity Models

Male C57BL/6J mice were fed a high‐fat diet (D12492) for ≈10 weeks. The control group and model group were administered PBS, while the Semaglutide group (10 nmol kg^−1^)^[^ ^40^ ^]^ and D13 group (10 nmol kg^−1^), D41 group (10 nmol kg^−1^), and D44 group (10 nmol kg^−1^) received continuous administration for 4 weeks, with five mice per group. During the continuous administration period, body weight was monitored twice per week. After the administration, the mice were deeply anesthetized with CO2, and blood was collected to measure blood lipids, including serum total cholesterol (TC) and low‐density lipoprotein cholesterol (LDL‐C). The abdominal cavity was then opened, and white adipose tissue from the epididymis, abdominal wall, and mesentery was collected, weighed, and the coefficient of fat was calculated. The liver was excised, weighed, and the coefficient of liver was also calculated.

### Statistical Analysis

Data are presented as Mean ± SD. Sample size and the number of replicates are specified in the Figure legends. Statistical analyses were conducted using R (v4.3.1). In general, data were analyzed with the two‐sided Student's unpaired t test. Statistically significant was defined as follows: ^*^ *P* < 0.05, ^**^ *P* < 0.01, and ^***^ *P* < 0.001.

## Conflict of Interest

The authors declare no conflict of interest.

## Author Contributions

H.F.C., Z.Y., and T.W. designed the overall project. T.W., X.C., and J.L. designed the in vitro and in vivo experiments. T.W., X.C., and J.L. performed the experiments. T.W., Z.Z., T.C., L.C., X.L., J.Z., X.R., X.H., and J.M. analyzed the results. T.W. wrote the manuscript. H.F.C., Z.Y., J.M., and L.J. reviewed the manuscript. All authors critically revised the paper and approved the final version.

## Supporting information



Supporting Information

Supporting Information

## Data Availability

The data that support the findings of this study are available from the corresponding author upon reasonable request.
