# Supplementary material for: AI‐Driven De Novo Design of Ultra Long‐Acting GLP‐1 Receptor Agonists
Source: Adv Sci (Weinh). 2025 Aug 11;12(40):e07044. doi: 10.1002/advs.202507044 (PMC12561408; doi:10.1002/advs.202507044)
Supplement: Supplementary file 1 — Supporting Information [file ADVS-12-e07044-s002.docx]

**Supplementary Information**

**AI-Driven *De Novo* Design of Ultra Long-acting GLP-1 Receptor Agonists**

Ting Wei^1^, Jiating Ma^1^, Xiaochen Cui^2^, Jiahui Lin^2^, Zhuoqi Zheng^1^, Liu Cheng^1^, Taiying Cui^1^, Xiaoqian Lin^1^, Junjie Zhu^1^, Xuyang Ran^1^, Xiaokun Hong^3^ , Luke Johnston^4^, Zhangsheng Yu^1,5,*^ , Haifeng Chen^1,*^

^1^State Key Laboratory of Microbial Metabolism, Department of Bioinformatics and Biostatistics, SJTU-Yale Joint Center for Biostatistics, National Experimental Teaching Center for Life Sciences and Biotechnology, School of Life Sciences and Biotechnology, Shanghai Jiao Tong University, 200240, Shanghai, China.

^2^Intelligent Medicine Original Medical Technology (Shanghai) Co., Ltd., Shanghai, China

^3^College of Biological Science and Engineering, Fuzhou University, Fuzhou 350116, Fujian, China

^4^School of Mathematical Sciences, Shanghai Jiao Tong University, Shanghai, China

^5^Clinical Research Institute, Shanghai Jiao Tong University School of Medicine, Shanghai, China.

*Correspondence should be addressed to Z. Yu ([yuzhangsheng@sjtu.edu.cn](mailto:yuzhangsheng@sjtu.edu.cn)) and H.F. Chen ([haifengchen@sjtu.edu.cn](mailto:haifengchen@sjtu.edu.cn)).

## De novo GLP-1RAs Design

12 residues (7H, 8Aib, 9E, 10G, 11T, 12F, 13T, 14S, 15D, 17S, 26K, 34R, 37G) were fixed in the GLP-1RAs design. The sequences of GLP-1RAs marked drug, including GLP-1, Exenatide, Lixisenatide, Liraglutide, Semaglutide, and Albiglutide, are shown in Figure S1.


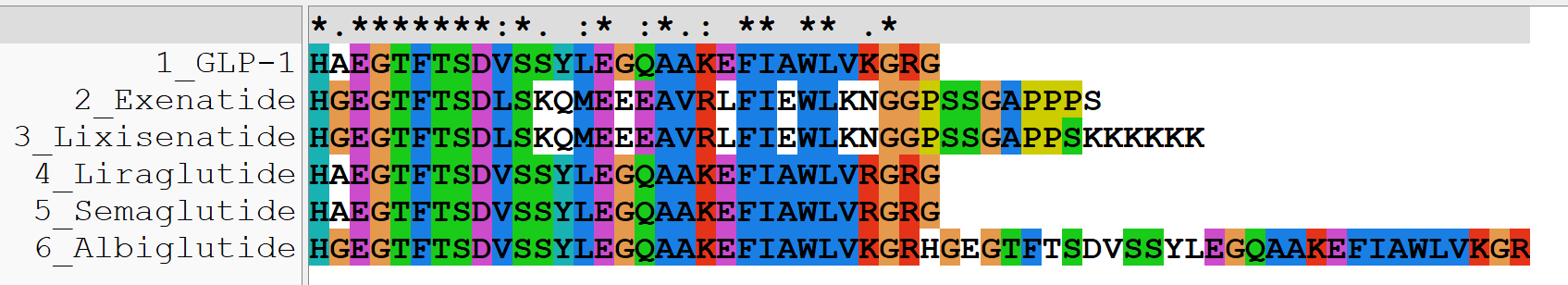


**Figure S1. The conserved sites of GLP-1RAs marked drug**

## Functional Screening of designed GLP-1RAs

Using ProteinMPNN, we generated a total of 10,000 GLP-1RA sequences. In vitro studies indicated that NEP-24.11 can cleave GLP-1 at six potential cleavage sites in the central and C-terminal regions. To improve stability and extend the half-life of the designed GLP-1RAs, sequences containing these cleavage sites were filtered out. After eliminating cleavage sites and redundant sequences, 787 unique candidates were obtained for further analysis.

AlphaFold2 was used to predict the complex structures of the designed GLP-1RA sequences and GLP-1R. The folding capability of the GLP-1RA designs was assessed using several key parameters: The predicted Local Distance Difference Test (pLDDT) scores, which evaluate the quality of the predicted protein structure; the TM-score and Root Mean Square Deviation (RMSD), which assess how well the designed structure aligns with the native structure; and the interface pAE, which evaluates the confidence in the interactions at the interface between GLP-1RA and GLP-1R in the complex. Based on these evaluations, 359 GLP-1RA sequences with favorable folding properties were selected.

After applying the net charge, hydrophobicity and spatial aggregation propensity (SAP) score, a total of 86 GLP-1RA sequences with favorable solubility profiles were retained for further analysis.

MD simulations were carried out for the 86 GLP-1RA and GLP-1R complexes to evaluate their stability and interaction mechanisms. MD simulations provided insights into the binding affinity, which measures the binding strength between GLP-1RA and GLP-1R. Among the tested sequences, 84 demonstrated binding affinities lower than that of Semaglutide, aligning with the expected interaction patterns and indicating enhanced binding efficiency.

Phylogenetic trees were generated, and select a single representative sequence in each phylogenetic cluster. This approach resulted in a final set of 60 unique GLP-1RA sequences, representing diverse candidates for subsequent experimental evaluation.


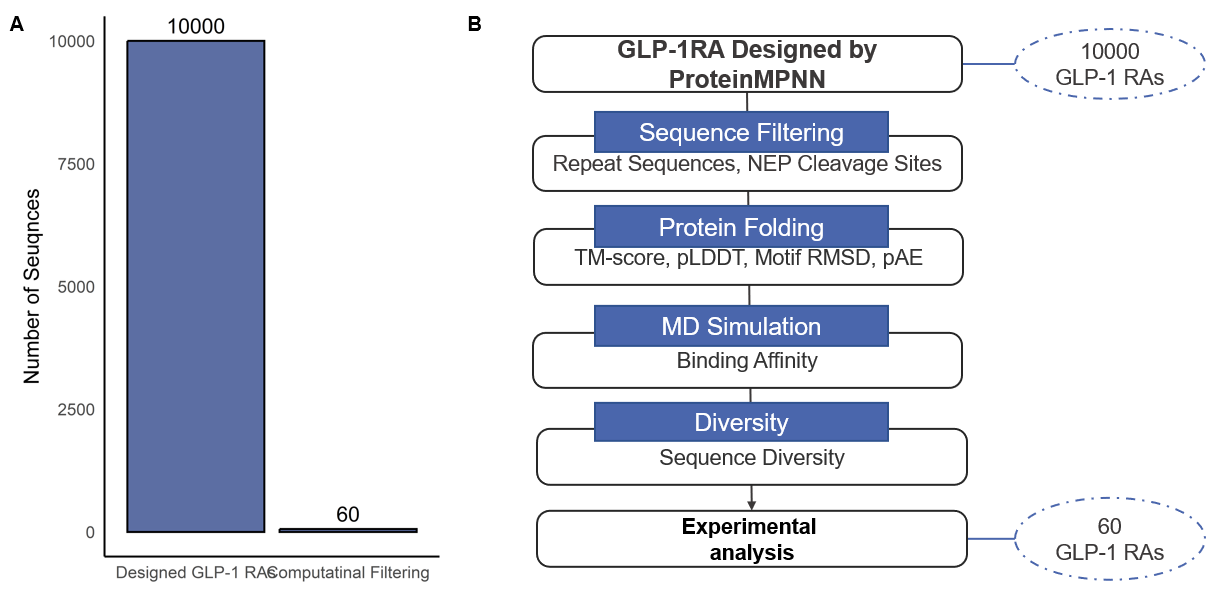


**Figure S2. The computational screening of designed GLP-1RAs. (A)** The number of computational screenings. **(B)** The steps of computational screening of designed GLP-1RAs.

## *In vitro* Experimental Validation

### The GST pulldown assay

The GST-peptide is connected by GST-Linker-Peptide, the sequences of biosynthesis are shown in Table S1. For Semaglutide as an example, the protein sequence is HAEGTFTSDVSSYLEGQAAKEFIAWLVRGRG, the linker is GGDDDDK, the GST-Linker-Peptide is GST-GGDDDDK- HAEGTFTSDVSSYLEGQAAKEFIAWLVRGR.

The purchase GLP-1R (MCE, HY-P700468) is the extracellular domain (R24-Y145) of the GLP-1R (blue in Figure S3). The C-terminal of GLP-1RA (yellow in Figure S3) was bound to the extracellular domain. We have fixed 12 residues (7H, 8Aib, 9E, 10G, 11T, 12F, 13T, 14S, 15D, 17S, 26K, 34R, 37G), that is mainly located on N-terminal of GLP-1R (red in Figure S3). We tested the binding affinity between C-terminal GLP-1RA and GLP-1R. We also used MD simulation to assess the binding affinity between GLP-1RA and GLP-1R. Firstly, we used AlphaFold3 to predict the structure of GST-Linker-Peptide, and used MD to calculate the binding affinity of GST-Linker-Peptide and GLP-1R. The binding affinity is -90 kcal/mol. In summary, the large tag (pink in Figure S3) will not affect binding.

**Table S1. GLP-1RAs sequences for biosynthesis and chemical synthesis**

| ID | **Biosynthesis** | | | | | **Chemical synthesis** |
| --- | --- | --- | --- | --- | --- | --- |
|  | **Protein Sequences** | **Affinity tag** | **Linker** | **GST-Linker-Peptide** | **DNA of Linker-Peptide** | **Protein Sequences** |
| Semaglutide | HAEGTFTSDVSSYLEGQAAKEFIAWLVRGRG | GST | GGDDDDK | MSPILGYWKIKGLVQPTRLLLEYLEEKYEEHLYERDEGDKWRNKKFELGLEFPNLPYYIDGDVKLTQSMAIIRYIADKHNMLGGCPKERAEISMLEGAVLDIRYGVSRIAYSKDFETLKVDFLSKLPEMLKMFEDRLCHKTYLNGDHVTHPDFMLYDALDVVLYMDPMCLDAFPKLVCFKKRIEAIPQIDKYLKSSKYIAWPLQGWQATFGGGDHPPKSDLVPRGSGGDDDDKHAEGTFTSDVSSYLEGQAAKEFIAWLVRGRG | GGAGGGGATGATGACGATAAACACGCCGAGGGAACATTCACGTCTGATGTTTCATCCTACCTGGAAGGACAGGCGGCAAAAGAGTTTATTGCCTGGCTTGTTCGCGGCCGCGGCtaatag | H-Aib-EGTFTSDVSSYLEGQAAK(AEEA-AEEA-γE-C18Diacid)EFIAWLVRGRG-COOH |
| D13 | HAEGTFTSDVSSYLEEQAAKAFVERLKRGRG | GST | GGDDDDK | MSPILGYWKIKGLVQPTRLLLEYLEEKYEEHLYERDEGDKWRNKKFELGLEFPNLPYYIDGDVKLTQSMAIIRYIADKHNMLGGCPKERAEISMLEGAVLDIRYGVSRIAYSKDFETLKVDFLSKLPEMLKMFEDRLCHKTYLNGDHVTHPDFMLYDALDVVLYMDPMCLDAFPKLVCFKKRIEAIPQIDKYLKSSKYIAWPLQGWQATFGGGDHPPKSDLVPRGSGGDDDDKHAEGTFTSDVSSYLEEQAAKAFVERLKRGRG | GGAGGGGATGATGACGATAAACACGCCGAGGGAACATTCACGTCTGATGTTTCATCCTACCTGGAAGAACAGGCGGCAAAAGCGTTTGTGGAACGCCTTAAACGCGGCCGCGGCtaatag | H-Aib-EGTFTSDVSSYLEEQAAK(AEEA-AEEA-γE-C20Diacid)AFVERLKRGRG-COOH |
| D41 | HAEGTFTSDVSSYLEEQQVKQFVERLKRGRG | GST | GGDDDDK | MSPILGYWKIKGLVQPTRLLLEYLEEKYEEHLYERDEGDKWRNKKFELGLEFPNLPYYIDGDVKLTQSMAIIRYIADKHNMLGGCPKERAEISMLEGAVLDIRYGVSRIAYSKDFETLKVDFLSKLPEMLKMFEDRLCHKTYLNGDHVTHPDFMLYDALDVVLYMDPMCLDAFPKLVCFKKRIEAIPQIDKYLKSSKYIAWPLQGWQATFGGGDHPPKSDLVPRGSGGDDDDKHAEGTFTSDVSSYLEEQQVKQFVERLKRGRG | GGAGGGGATGATGACGATAAACACGCCGAGGGAACATTCACGTCTGATGTTTCATCCTACCTGGAAGAACAGCAGGTGAAACAGTTTGTGGAACGCCTTAAACGCGGCCGCGGCtaatag | H-Aib-EGTFTSDVSSYLEEQQVK(AEEA-AEEA-γE-C20Diacid)QFVERLKRGRG- |
| D44 | HAEGTFTSDVSRYLEEKAVKDFVERLKRGAG | GST | GGDDDDK | MSPILGYWKIKGLVQPTRLLLEYLEEKYEEHLYERDEGDKWRNKKFELGLEFPNLPYYIDGDVKLTQSMAIIRYIADKHNMLGGCPKERAEISMLEGAVLDIRYGVSRIAYSKDFETLKVDFLSKLPEMLKMFEDRLCHKTYLNGDHVTHPDFMLYDALDVVLYMDPMCLDAFPKLVCFKKRIEAIPQIDKYLKSSKYIAWPLQGWQATFGGGDHPPKSDLVPRGSGGDDDDKHAEGTFTSDVSRYLEEKAVKDFVERLKRGAG | GGAGGGGATGATGACGATAAACACGCCGAGGGAACATTCACGTCTGATGTTTCACGCTACCTGGAAGAAAAAGCGGTGAAAGATTTTGTGGAACGCCTTAAACGCGGCGCGGGCtaatag | H-Aib-EGTFTSDVSRYLEEKAVK(AEEA-AEEA-γE-C20Diacid)DFVERLKRGAG-COOH |


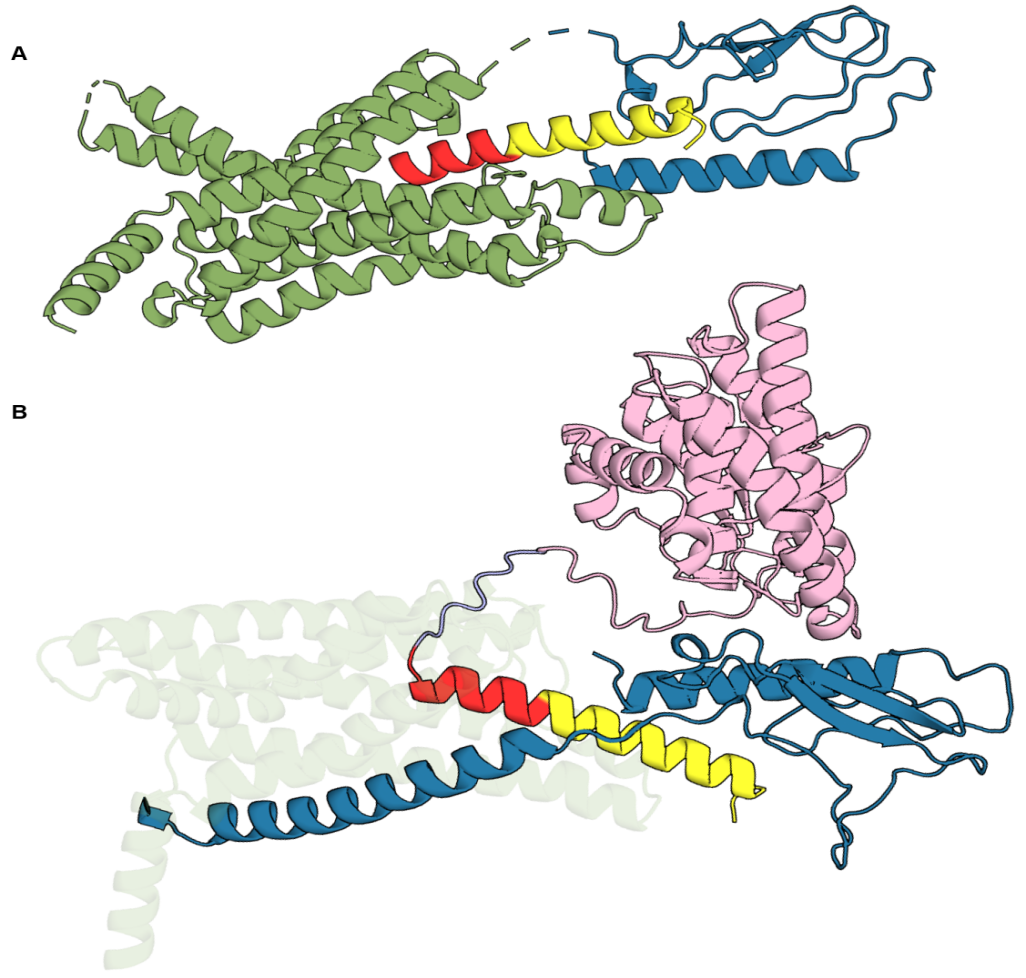


**Figure S3. The structure of GST-Linker-Peptide. (A)** The C-terminal of GLP-1RA is red and the N-terminal is yellow. The C-terminal of GLP-1RA (yellow) was bound to the extracellular domain of GLP-1R (blue). **(B)** The complex structure of GLP-1R and GST-linker-peptide. The GST link (pink) will not affect binding.


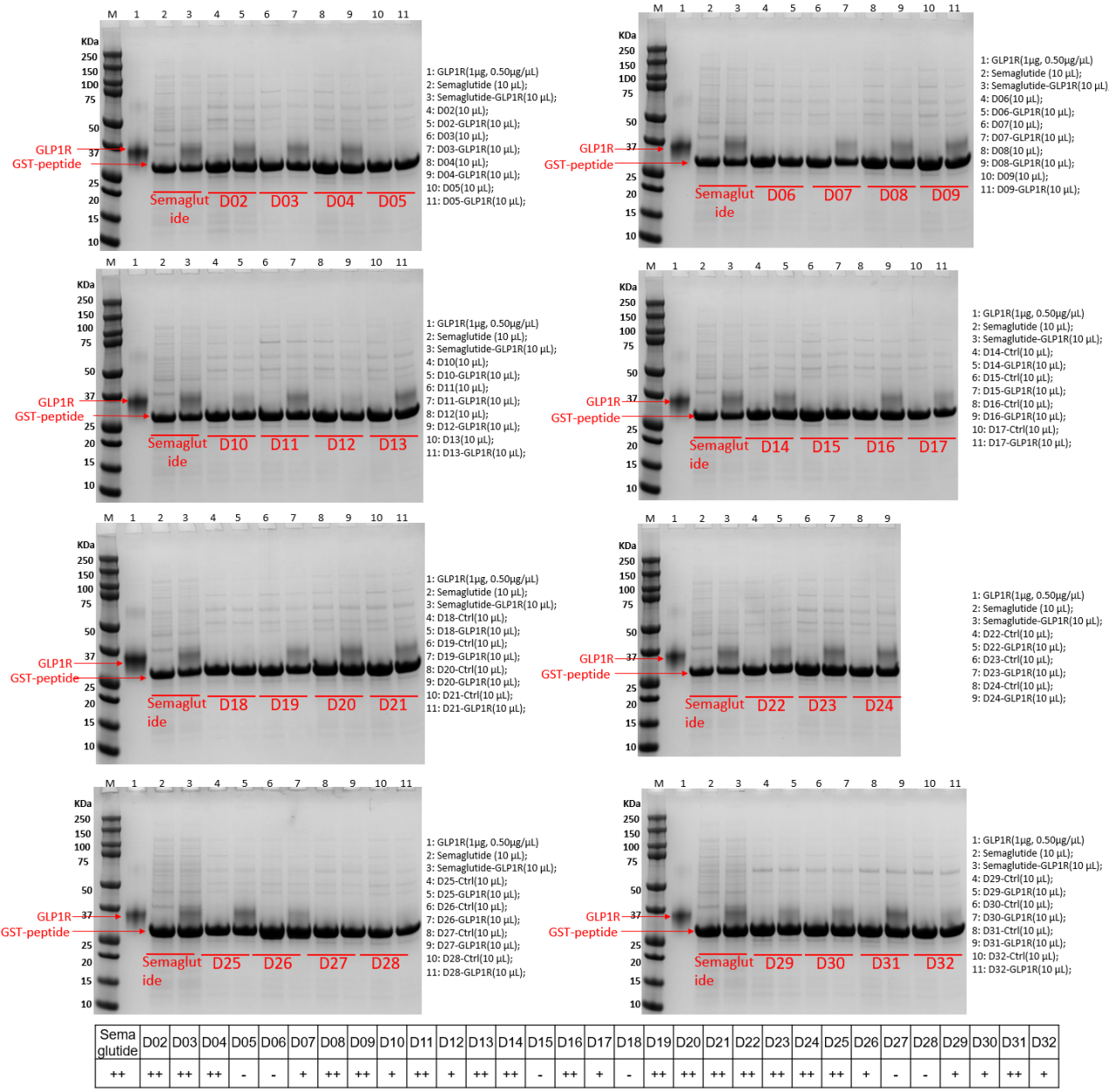


**Figure S4. SDS-PAGE images of D02-D32.** Semaglutide- GLP-1R was used as a positive control. “++” indicates that the peptide has a binding capability to GLP-1R comparable to that of Semaglutide. “+” indicates weaker binding, while “–” means the peptide does not bind to GLP-1R.


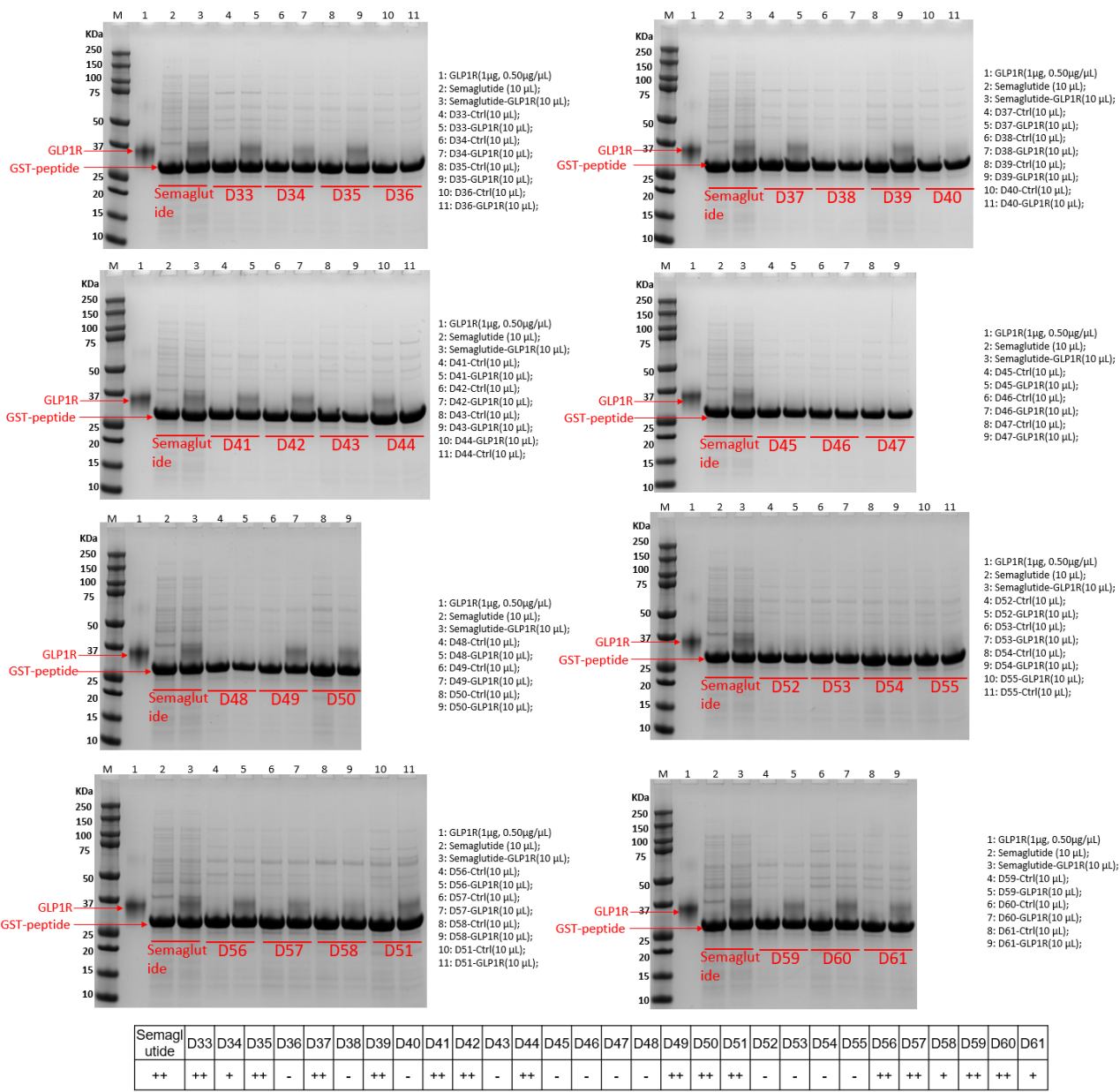


**Figure S5. SDS-PAGE images of D33-D61.** Semaglutide- GLP-1R was used as positive control. “++” indicates that the peptide has a binding capability to GLP-1R comparable to that of Semaglutide. “+” indicates weaker binding, while “–” means the peptide does not bind to GLP-1R.

### The SPR assay


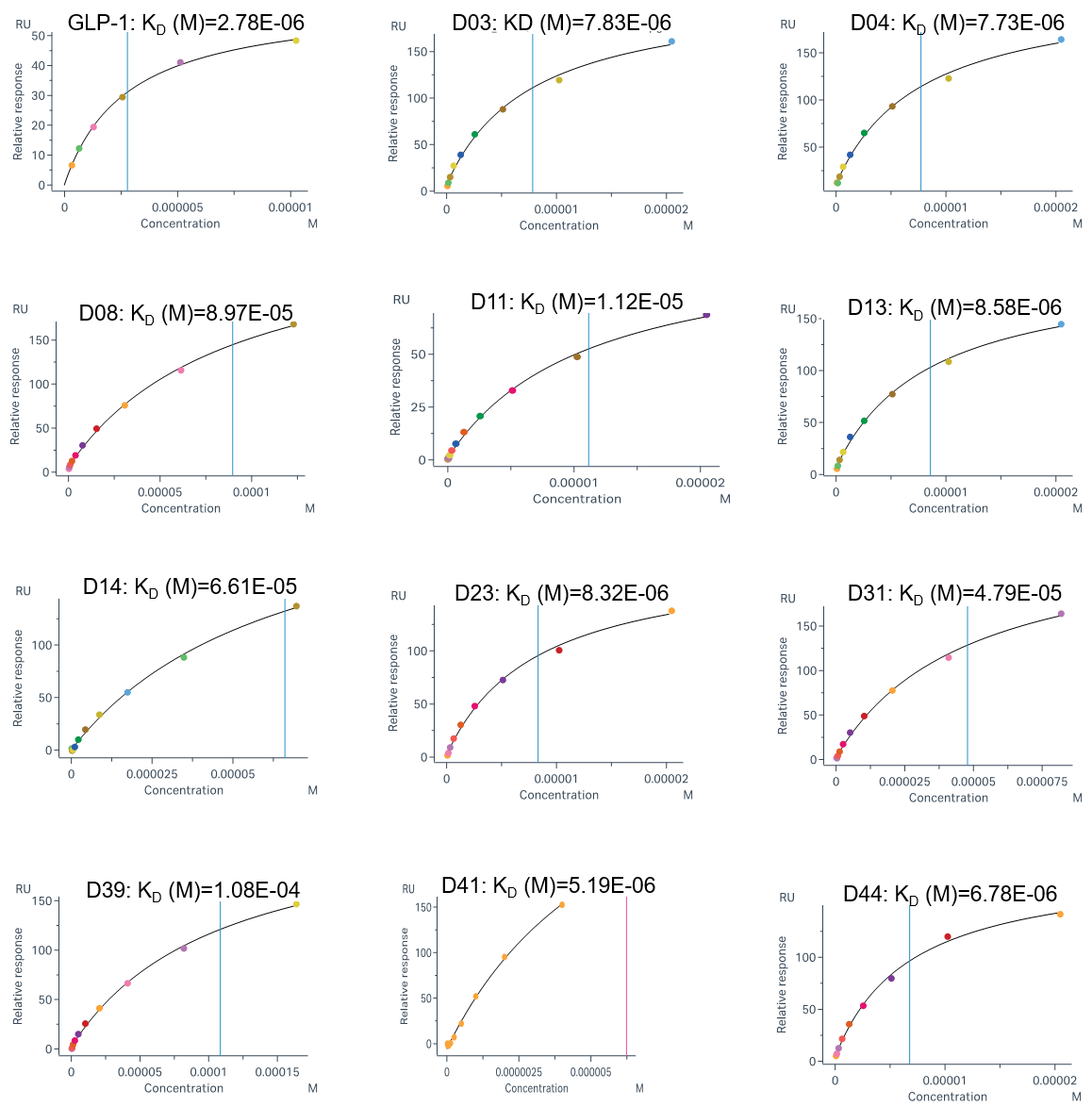


**Figure S6. The SPR dose-dependent saturation binding of GPL-1 and GLP-1R**

**Table S2. The detailed information of SPR**

| **Analytical method** | **Parameters** |
| --- | --- |
| Machine | Biacore 8K (Cytiva) |
| Basic | - Chip: CM5(Cytiva) - Running buffer:1×HBS-EP (pH 7.4) |
| Immobilization | - Capture molecule: HIS10-GLP1R-MYC - Concentration: 8 µg/mL - Contact time : 70 sec - Flow rate : 10 µL/min |
| Analyte Association / Dissociation | - Analyte: WBPD081 samples - Working concentration: - Association time : 240 sec - Dissociation time : 300 sec - Flow rate : 30 µL/min |
| Regeneration | - Regeneration buffer: 50 mM NaOH - Contact time : 30 sec - Flow rate : 30 µL/min |
| Evaluation | - Fitting model: steady state affinity model - Evaluation software: Biacore evaluation software (Cytiva) |

**Chemical synthesis**

**Table S3. The characters of peptides of Chemical synthesis**

| ID | Mw | amount(mg) | PURITY | SALT | physical property | color |
| --- | --- | --- | --- | --- | --- | --- |
| D13 | 4198.75 | 2.0*5 | 0.978 | TFA | solid | white |
| D41 | 4340.91 | 2.0*5 | 0.9803 | TFA | solid | white |
| D44 | 4254.86 | 2.0*5 | 0.9771 | TFA | solid | white |

## Study Design of in vivo Experiment


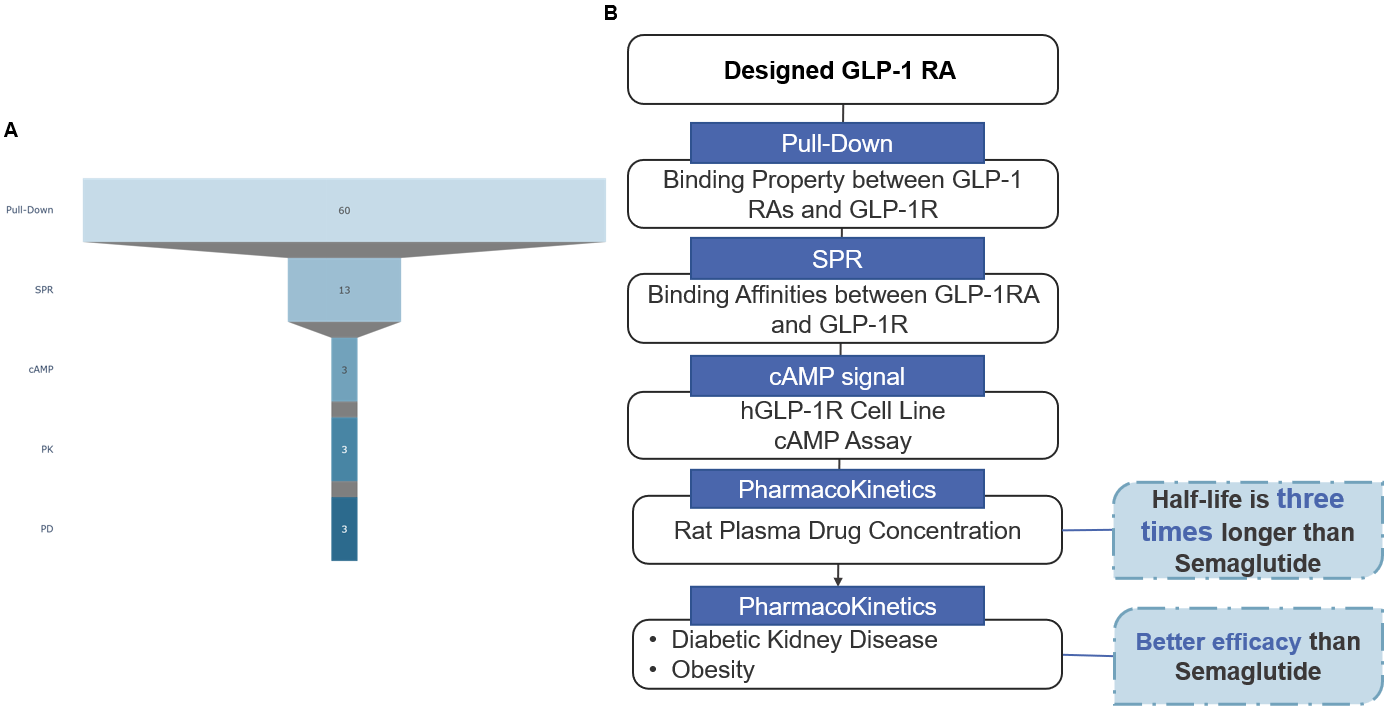


**Figure S7. The experiment of designed GLP-1RAs. (B)** The number of experimental validations through in vitro and in vivo studies. **(D)** The steps of experimental validations through in vitro and in vivo studies.

**Table S4. The PK results in rats (Mean±SD, n=3)**

| **Compounds** | **T1/2**  **(hour)** | **Tmax**  **(hour)** | **Cmax (ng/mL)** | **AUC_0-t_ (h*ng/mL)** | **MRT_0-t_**  **(hour)** |
| --- | --- | --- | --- | --- | --- |
| D13 | 19.86±1.55 | 10.67±2.31 | 353.96±41.90 | 15939.88±2952.53 | 32.02±2.89 |
| D41 | 23.16±3.69 | 12.00±0.00 | 294.13±37.18 | 15737.19±822.42 | 38.79±1.15 |
| D44 | 4.19±0.37 | 4.00±0.00 | 116.60±20.36 | 1276.31±139.04 | 7.69±0.35 |
| Semaglutide | 8.17±0.29 | 8.00±0.00 | 329.82±10.44 | 7538.16±75.63 | 14.21±0.23 |

**Table S5. The sample preparation and detailed information of LC-MS/MS**

| **Content** | **Parameters** |
| --- | --- |
| **Instrument** | LC-MS/MS (Waters XEVO TQ-XS) |
| **Matrix** | SD Rat Plasma(K2-EDTA) |
| **Internal standard(s)** | Tolbutamide |
| **MS conditions** | ESI: Positive  MRM detection   - D13: Q1/Q3 Masses is 840.6/1047.3 Da - D41: Q1/Q3 Masses is 1086/1330.7 Da - D44: Q1/Q3 Masses is 851.8/1008.9 Da - Semaglutide: Q1/Q3 Masses is 1029.1/690.0 Da - Tolbutamide: Q1/Q3 Masses is271.072/91.004 Da |
| **HPLC conditions** | Mobile phase:   - Mobile phase A: 0.1%FA in H2O - Mobile phase B: 0.1%FA in CAN   Column: ACQUITY Premier Peptide BEH C18 130A 1.7 μm (2.1×100 mm)  Oven: 60℃  Flow rate: 0.30 mL/min  Retention time:   - D13 is 3.04min - D41 is 3.01min - D44 is 3.00min - Semaglutide is 3.26 min - Tolbutamide is 3.31 min |
| **Sample preparation** | 40 µL plasma sample was added 160 µL 30%MeOH.ACN with 10ng/mL IS(Tolbutamide) to make protein precipitation. The mixture was vortexed for 1 min and centrifuged at 14000rpm under 4℃ for 10 min. Transfer 100 µL supernatant and add with 100 ul H2O. The mixture was vortexed for 3 min, and then 10 µL of solvent was injected for LC-MS/MS analysis. |
| **Calibration curve** | 2.00-1000ng/mL for D13, D41, D44, Semaglutide in rat plasma samples. |

## Sequence and Structure analysis of GLP-1RAs


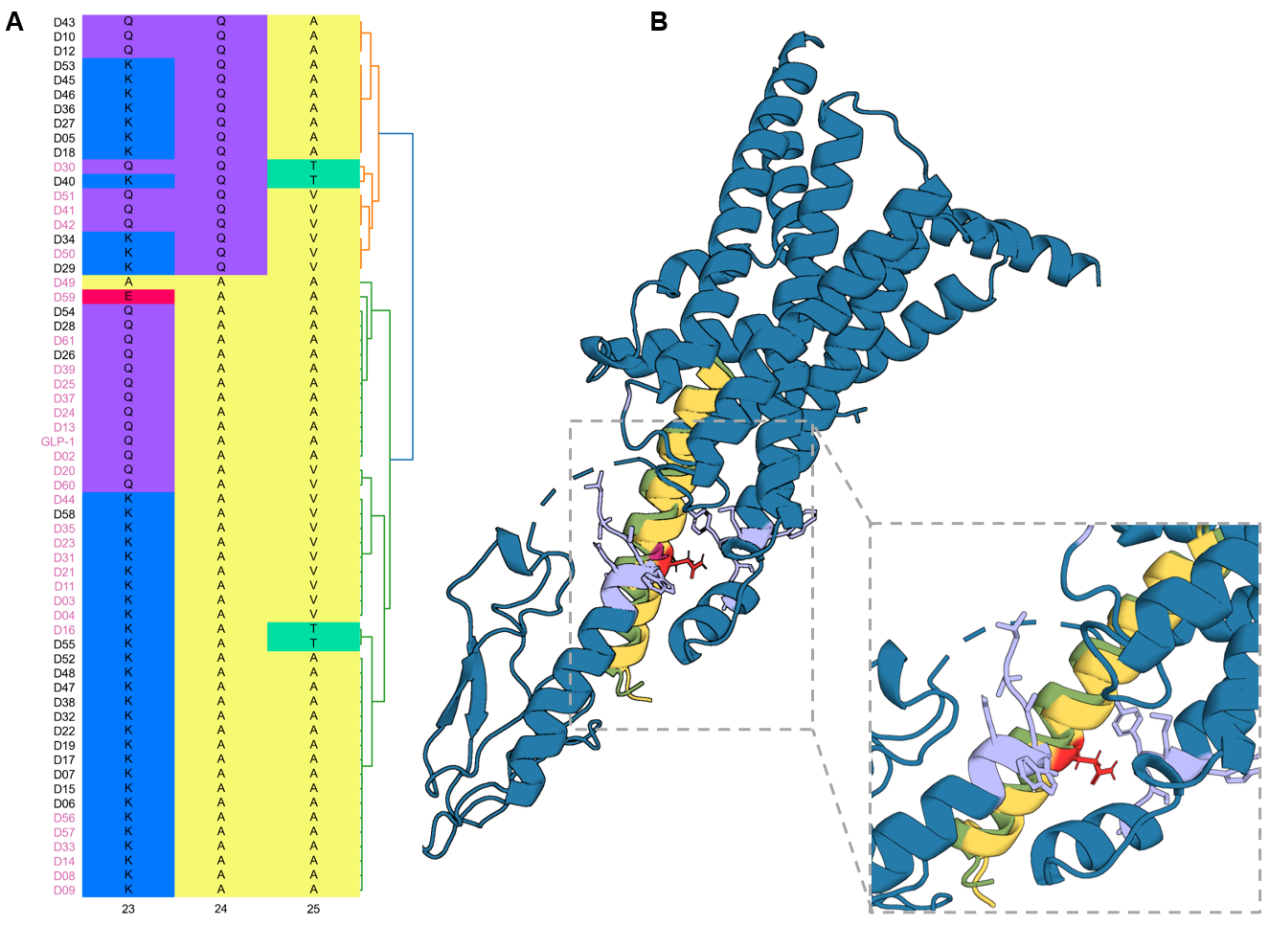


**Figure S8. Structure and sequence characteristics of designed GLP-1RAs.** (A) Sequence-based alignment for residue 23-26 from successful (red text) and failed (black text) GLP-1RAs in GST pulldown assays. Residues are annotated by their biochemical properties, and hierarchical clustering is shown on the right. Residue indices are based on the number from 7. (B) The residue 24Q, with its large side chain (shown in red) may clash with the pocket residues (shown in purple) of GLP-1R. This potential clash could explain why most sequences containing 24Q failed.


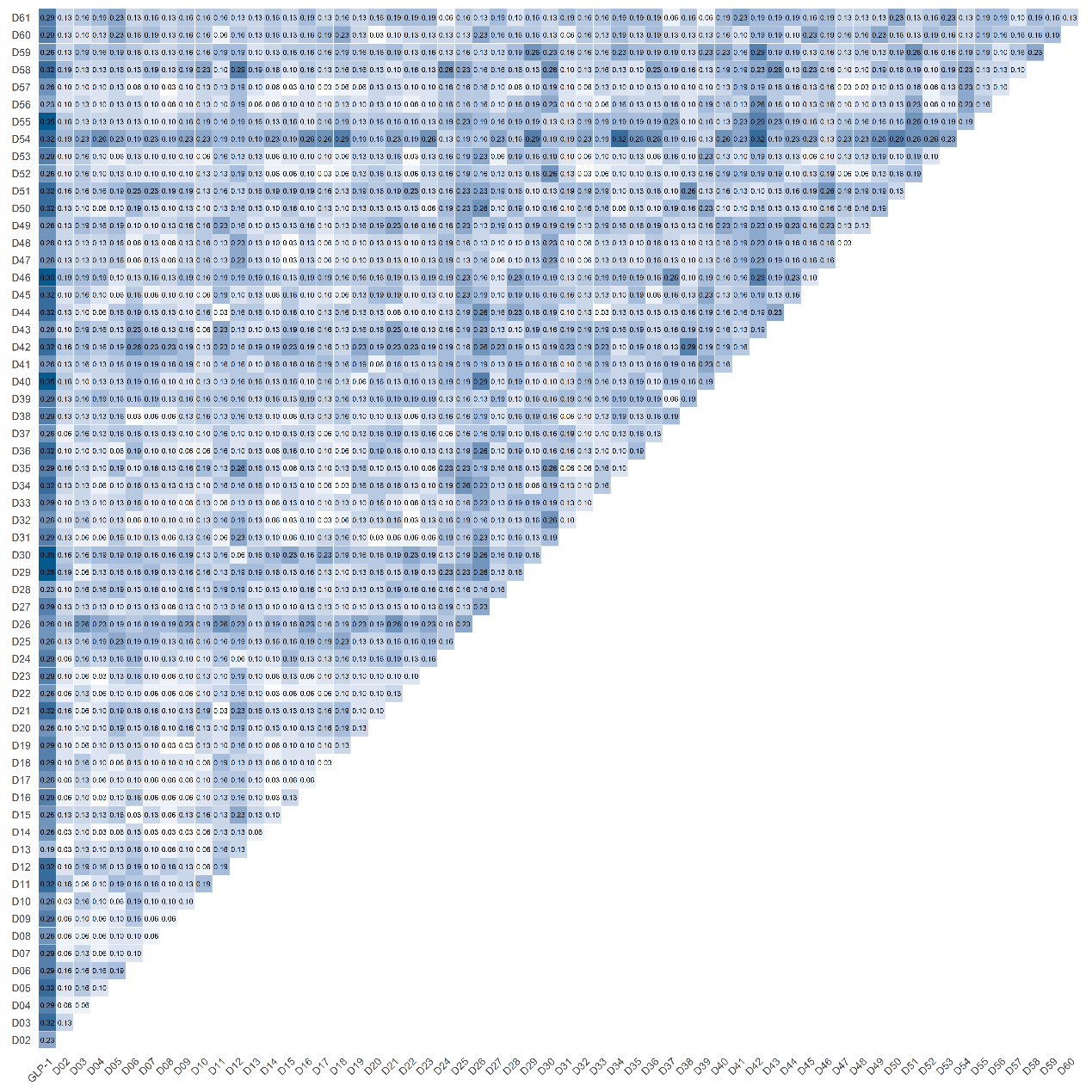


**Figure S9. The phylogenetic distance of designed GLP-1RAs.**
