# Supplementary material for: AI‐Driven De Novo Design of Ultra Long‐Acting GLP‐1 Receptor Agonists
Source: Adv Sci (Weinh). 2025 Aug 11;12(40):e07044. doi: 10.1002/advs.202507044 (PMC12561408; doi:10.1002/advs.202507044)
Supplement: Supplementary file 2 — Supporting Information [file ADVS-12-e07044-s001.zip › SI_The raw data of PK for D13.pdf]

Dataset: D:\Data\27013-24001-NG.PRO\20241212_WBPD081_013_SA_Reinjection_Processed-Tu.qld

Last Altered: Tuesday, July 15, 2025 15:44:36 China Standard Time
Printed: Tuesday, July 15, 2025 15:46:23 China Standard Time

Method: D:\Data\27013-24001-NG.PRO\MethDB\20241212_WBPD081_013_yyb.mdb 13 Dec 2024 11:13:31
Calibration: 15 Jul 2025 15:44:36

Compound name: WBPD081_013 (4)
Correlation coefficient: r = 0.998955, r^2 = 0.997911
Calibration curve: 0.000106248 * x + 7.30001e-006
Response type: Internal Std (Ref 2), Area * (IS Conc. / IS Area)
Curve type: Linear, Origin: Exclude, Weighting: 1/x^2, Axis trans: None

	Name	ID	Type	Std. Conc	RT	Area	IS Area	Response	Conc.	%Dev	Primar...
1	20241212_1_001	Solvent			3.06	148.147	951.956	0.156	1464.7		dd
2	20241212_1_002	Solvent			3.05	0.335	668.182	0.001	4.7		bb
3	20241212_1_003	B	Blank		3.13	0.581					bb
4	20241212_1_004	O	Blank		2.94	2.603	714914.250	0.000			bbI
5	20241212_1_005	STD1	Standard	2.000	3.02	139.793	637210.750	0.000	2.0	-0.2	bb
6	20241212_1_006	STD2	Standard	5.000	3.13	7.128	690256.813	0.000	0.0	-99.4	dbX
7	20241212_1_007	STD3	Standard	10.000	3.04	715.224	657628.000	0.001	10.2	1.7	bb
8	20241212_1_008	STD4	Standard	50.000	3.04	3465.741	678704.000	0.005	48.0	-4.0	bb
9	20241212_1_009	STD5	Standard	100.000	3.03	3.741	415.610	0.009	84.7	-15.3	bbX
10	20241212_1_010	STD6	Standard	500.000	3.04	38548.813	674419.375	0.057	537.9	7.6	bb
11	20241212_1_011	STD7	Standard	900.000	3.04	62607.488	660758.625	0.095	891.7	-0.9	bb
12	20241212_1_012	STD8	Standard	1000.000	3.04	68572.266	673131.625	0.102	958.7	-4.1	bb
13	20241212_1_013	Solvent			3.04	226.198	1079.635	0.210	1971.9		bb
14	20241212_1_014	Solvent			3.05	37.265	833.903	0.045	420.5		bb
15	20241212_1_015	B	Blank		3.10	5.481	572.221	0.010	90.1		db
16	20241212_1_016	O	Blank		3.03	24.326	550.533	0.044	415.8		bb
17	20241212_1_017	Q1	QC	6.000	3.04	544.577	722437.813	0.001	7.0	17.1	bb
18	20241212_1_018	Q2	QC	12.000	3.04	924.185	685338.063	0.001	12.6	5.2	bb
19	20241212_1_019	Q3	QC	80.000	3.04	6061.994	693016.563	0.009	82.3	2.8	bb
20	20241212_1_020	Q4	QC	800.000	3.04	66168.148	696033.000	0.095	894.7	11.8	bb
21	20241212_1_021	Solvent			3.04	256.707	811.893	0.316	2975.8		db
22	20241212_1_022	Solvent			3.04	33.200	804.311	0.041	388.4		bb
23	20241212_1_023	101-Predose			3.04	64.994	658671.750	0.000	0.9		bb
24	20241212_1_024	101-2h			3.04	9690.713	757266.688	0.013	120.4		bb
25	20241212_1_025	101-4h			3.04	17956.346	707963.813	0.025	238.6		bb
26	20241212_1_026	101-8h			3.04	26826.949	673099.563	0.040	375.1		bb
27	20241212_1_027	101-12h			3.04	27711.328	691105.000	0.040	377.3		bb
28	20241212_1_028	101-24h			3.04	21057.592	617150.688	0.034	321.1		bb
29	20241212_1_029	101-48h			3.04	9629.165	644218.500	0.015	140.6		bb
30	20241212_1_030	101-72h			3.04	3718.726	623248.063	0.006	56.1		bb
31	20241212_1_031	101-96h			3.04	1514.152	688654.375	0.002	20.6		bd
32	20241212_1_032	101-168h			3.04	175.776	632261.313	0.000	2.5		bd
33	20241212_1_033	Solvent			3.04	5.648	865.394	0.007	61.4		bb
34	20241212_1_034	Solvent			3.03	1.704	975.246	0.002	16.4		bb
35	20241212_1_035	102-Predose			3.05	16.923	678433.875	0.000	0.2		bb
36	20241212_1_036	102-2h			3.04	7769.788	682413.750	0.011	107.1		bb
37	20241212_1_037	102-4h			3.04	12721.282	658029.688	0.019	181.9		bb
38	20241212_1_038	102-8h			3.04	18223.262	645551.938	0.028	265.6		bb
39	20241212_1_039	102-12h			3.04	20340.768	626353.375	0.032	305.6		bb
40	20241212_1_040	102-24h			3.04	17606.041	651858.000	0.027	254.1		bb
41	20241212_1_041	102-48h			3.04	7386.808	642575.250	0.011	108.1		bb
42	20241212_1_042	102-72h			3.04	2904.449	684711.750	0.004	39.9		bb
43	20241212_1_043	102-96h			3.04	1169.926	662454.688	0.002	16.6		bb
44	20241212_1_044	102-168h			3.04	62.472	656252.813	0.000	0.8		bb

Dataset: D:\Data\27013-24001-NG.PRO\20241212_WBPD081_013_SA_Reinjection_Processed-Tu.qld

Last Altered: Tuesday, July 15, 2025 15:44:36 China Standard Time
Printed: Tuesday, July 15, 2025 15:46:23 China Standard Time

Compound name: WBPD081_013 (4)

	Name	ID	Type	Std. Conc	RT	Area	IS Area	Response	Conc.	%Dev	Primar...
45	20241212_1_045	Solvent					1030.409				
46	20241212_1_046	Solvent					807.360				
47	20241212_1_047	103-Predose			3.03	3.586	690653.500	0.000			bbI
48	20241212_1_048	103-2h			3.04	8905.885	688727.688	0.013	121.6		bb
49	20241212_1_049	103-4h			3.04	19327.619	653959.000	0.030	278.1		bb
50	20241212_1_050	103-8h			3.04	26810.475	665752.500	0.040	379.0		bb
51	20241212_1_051	103-12h			3.04	27258.576	683150.500	0.040	375.5		bb
52	20241212_1_052	103-24h			3.04	21991.320	654158.688	0.034	316.3		bb
53	20241212_1_053	103-48h			3.04	11913.680	661095.625	0.018	169.5		bb
54	20241212_1_054	103-72h			3.04	4105.340	642292.438	0.006	60.1		bb
55	20241212_1_055	103-96h			3.04	1890.755	666816.938	0.003	26.6		bb
56	20241212_1_056	103-168h			3.04	177.615	649023.250	0.000	2.5		bb
57	20241212_1_057	Solvent					779.521				
58	20241212_1_058	Solvent					727.294				
59	20241212_1_059	B	Blank		3.04	2.753	552.923	0.005	46.8		bb
60	20241212_1_060	O	Blank		3.04	7.929	452.709	0.018	164.8		bb
61	20241212_1_061	Q1	QC	6.000	3.04	510.812	693889.500	0.001	6.9	14.3	bb
62	20241212_1_062	Q2	QC	12.000	3.04	1170.124	703905.938	0.002	15.6	29.8	bb
63	20241212_1_078	Q3	QC	80.000	3.03	2405.955	245563.938	0.010	92.1	15.2	bb
64	20241212_1_079	Q4	QC	800.000	3.03	24577.082	244274.516	0.101	946.9	18.4	bb

Dataset: D:\Data\27013-24001-NG.PRO\20241212_WBPD081_013_SA_Reinjection_Processed-Tu.qld

Last Altered: Tuesday, July 15, 2025 15:44:36 China Standard Time
Printed: Tuesday, July 15, 2025 15:46:23 China Standard Time

Compound name: WBPD081_013 (4)

	Inj. Vol	Factor1	Vial
1	10.000	0.0	2:H,12
2	10.000	0.0	2:H,12
3	10.000	0.0	2:A,1
4	10.000	0.0	2:A,2
5	10.000	1.0	2:A,3
6	10.000	1.0	2:A,4
7	10.000	1.0	2:A,5
8	10.000	1.0	2:A,6
9	10.000	1.0	2:A,7
10	10.000	1.0	2:A,8
11	10.000	1.0	2:A,9
12	10.000	1.0	2:A,10
13	10.000	0.0	2:H,12
14	10.000	0.0	2:H,12
15	10.000	0.0	2:A,1
16	10.000	0.0	2:A,2
17	10.000	0.0	2:A,11
18	10.000	0.0	2:A,12
19	10.000	0.0	2:B,1
20	10.000	0.0	2:B,2
21	10.000	0.0	2:H,12
22	10.000	0.0	2:H,12
23	10.000	0.0	2:B,7
24	10.000	0.0	2:B,8
25	10.000	0.0	2:B,9
26	10.000	0.0	2:B,10
27	10.000	0.0	2:B,11
28	10.000	0.0	2:B,12
29	10.000	0.0	2:C,1
30	10.000	0.0	2:C,2
31	10.000	0.0	2:C,3
32	10.000	0.0	2:C,4
33	10.000	0.0	2:H,12
34	10.000	0.0	2:H,12
35	10.000	0.0	2:C,5
36	10.000	0.0	2:C,6
37	10.000	0.0	2:C,7
38	10.000	0.0	2:C,8
39	10.000	0.0	2:C,9
40	10.000	0.0	2:C,10
41	10.000	0.0	2:C,11
42	10.000	0.0	2:C,12
43	10.000	0.0	2:D,1
44	10.000	0.0	2:D,2
45	10.000	0.0	2:H,12
46	10.000	0.0	2:H,12
47	10.000	0.0	2:D,3
48	10.000	0.0	2:D,4
49	10.000	0.0	2:D,5
50	10.000	0.0	2:D,6
51	10.000	0.0	2:D,7

Dataset: D:\Data\27013-24001-NG.PRO\20241212_WBPD081_013_SA_Reinjection_Processed-Tu.qld

Last Altered: Tuesday, July 15, 2025 15:44:36 China Standard Time
Printed: Tuesday, July 15, 2025 15:46:23 China Standard Time

Compound name: WBPD081_013 (4)

	Inj. Vol	Factor1	Vial
52	10.000	0.0	2:D,8
53	10.000	0.0	2:D,9
54	10.000	0.0	2:D,10
55	10.000	0.0	2:D,11
56	10.000	0.0	2:D,12
57	10.000	0.0	2:H,12
58	10.000	0.0	2:H,12
59	10.000	0.0	2:A,1
60	10.000	0.0	2:A,2
61	10.000	0.0	2:B,3
62	10.000	0.0	2:B,4
63	10.000	0.0	2:B,5
64	10.000	0.0	2:B,6

Dataset: D:\Data\27013-24001-NG.PRO\20241212_WBPD081_013_SA_Reinjection_Processed-Tu.qld

Last Altered: Tuesday, July 15, 2025 15:44:36 China Standard Time
Printed: Tuesday, July 15, 2025 15:46:23 China Standard Time

Compound name: Tolbutamide (1)
Response Factor: 584066
RRF SD: 236370, Relative SD: 40.4697
Response type: External Std, Area
Curve type: RF

	Name	ID	Type	Std. Conc	RT	Area	IS Area	Response	Conc.	%Dev	Primar...
1	20241212_1_001	Solvent		1.000	3.39	951.956		951.956	0.0	-99.8	dd
2	20241212_1_002	Solvent		1.000	3.28	668.182		668.182	0.0	-99.9	dd
3	20241212_1_003	B	Blank	1.000							
4	20241212_1_004	O	Blank	1.000	3.32	714914.250		714914.250	1.2	22.4	bb
5	20241212_1_005	STD1	Standard	1.000	3.32	637210.750		637210.750	1.1	9.1	bb
6	20241212_1_006	STD2	Standard	1.000	3.32	690256.813		690256.813	1.2	18.2	bb
7	20241212_1_007	STD3	Standard	1.000	3.32	657628.000		657628.000	1.1	12.6	bb
8	20241212_1_008	STD4	Standard	1.000	3.32	678704.000		678704.000	1.2	16.2	bb
9	20241212_1_009	STD5	Standard	1.000	3.45	415.610		415.610	0.0	-99.9	bb
10	20241212_1_010	STD6	Standard	1.000	3.32	674419.375		674419.375	1.2	15.5	bb
11	20241212_1_011	STD7	Standard	1.000	3.33	660758.625		660758.625	1.1	13.1	bb
12	20241212_1_012	STD8	Standard	1.000	3.33	673131.625		673131.625	1.2	15.2	bb
13	20241212_1_013	Solvent		1.000	3.28	1079.635		1079.635	0.0	-99.8	bd
14	20241212_1_014	Solvent		1.000	3.30	833.903		833.903	0.0	-99.9	dd
15	20241212_1_015	B	Blank	1.000	3.40	572.221		572.221	0.0	-99.9	bd
16	20241212_1_016	O	Blank	1.000	3.47	550.533		550.533	0.0	-99.9	bb
17	20241212_1_017	Q1	QC	1.000	3.33	722437.813		722437.813	1.2	23.7	bb
18	20241212_1_018	Q2	QC	1.000	3.33	685338.063		685338.063	1.2	17.3	bb
19	20241212_1_019	Q3	QC	1.000	3.32	693016.563		693016.563	1.2	18.7	bb
20	20241212_1_020	Q4	QC	1.000	3.32	696033.000		696033.000	1.2	19.2	bb
21	20241212_1_021	Solvent		1.000	3.31	811.893		811.893	0.0	-99.9	bd
22	20241212_1_022	Solvent		1.000	3.34	804.311		804.311	0.0	-99.9	bd
23	20241212_1_023	101-Predose		1.000	3.33	658671.750		658671.750	1.1	12.8	bb
24	20241212_1_024	101-2h		1.000	3.33	757266.688		757266.688	1.3	29.7	bb
25	20241212_1_025	101-4h		1.000	3.33	707963.813		707963.813	1.2	21.2	bb
26	20241212_1_026	101-8h		1.000	3.33	673099.563		673099.563	1.2	15.2	bb
27	20241212_1_027	101-12h		1.000	3.33	691105.000		691105.000	1.2	18.3	bb
28	20241212_1_028	101-24h		1.000	3.33	617150.688		617150.688	1.1	5.7	bb
29	20241212_1_029	101-48h		1.000	3.33	644218.500		644218.500	1.1	10.3	bb
30	20241212_1_030	101-72h		1.000	3.33	623248.063		623248.063	1.1	6.7	bb
31	20241212_1_031	101-96h		1.000	3.32	688654.375		688654.375	1.2	17.9	bb
32	20241212_1_032	101-168h		1.000	3.33	632261.313		632261.313	1.1	8.3	bb
33	20241212_1_033	Solvent		1.000	3.29	865.394		865.394	0.0	-99.9	dd
34	20241212_1_034	Solvent		1.000	3.28	975.246		975.246	0.0	-99.8	bd
35	20241212_1_035	102-Predose		1.000	3.33	678433.875		678433.875	1.2	16.2	bb
36	20241212_1_036	102-2h		1.000	3.33	682413.750		682413.750	1.2	16.8	bb
37	20241212_1_037	102-4h		1.000	3.32	658029.688		658029.688	1.1	12.7	bb
38	20241212_1_038	102-8h		1.000	3.33	645551.938		645551.938	1.1	10.5	bb
39	20241212_1_039	102-12h		1.000	3.32	626353.375		626353.375	1.1	7.2	bb
40	20241212_1_040	102-24h		1.000	3.32	651858.000		651858.000	1.1	11.6	bb
41	20241212_1_041	102-48h		1.000	3.33	642575.250		642575.250	1.1	10.0	bb
42	20241212_1_042	102-72h		1.000	3.32	684711.750		684711.750	1.2	17.2	bb
43	20241212_1_043	102-96h		1.000	3.32	662454.688		662454.688	1.1	13.4	bb
44	20241212_1_044	102-168h		1.000	3.32	656252.813		656252.813	1.1	12.4	bb
45	20241212_1_045	Solvent		1.000	3.30	1030.409		1030.409	0.0	-99.8	bd
46	20241212_1_046	Solvent		1.000	3.29	807.360		807.360	0.0	-99.9	dd
47	20241212_1_047	103-Predose		1.000	3.32	690653.500		690653.500	1.2	18.2	bb
48	20241212_1_048	103-2h		1.000	3.32	688727.688		688727.688	1.2	17.9	bb

Dataset: D:\Data\27013-24001-NG.PRO\20241212_WBPD081_013_SA_Reinjection_Processed-Tu.qld

Last Altered: Tuesday, July 15, 2025 15:44:36 China Standard Time
Printed: Tuesday, July 15, 2025 15:46:23 China Standard Time

Compound name: Tolbutamide (1)

	Name	ID	Type	Std. Conc	RT	Area	IS Area	Response	Conc.	%Dev	Primar...
49	20241212_1_049	103-4h		1.000	3.33	653959.000		653959.000	1.1	12.0	bb
50	20241212_1_050	103-8h		1.000	3.32	665752.500		665752.500	1.1	14.0	bb
51	20241212_1_051	103-12h		1.000	3.32	683150.500		683150.500	1.2	17.0	bb
52	20241212_1_052	103-24h		1.000	3.33	654158.688		654158.688	1.1	12.0	bb
53	20241212_1_053	103-48h		1.000	3.32	661095.625		661095.625	1.1	13.2	bb
54	20241212_1_054	103-72h		1.000	3.32	642292.438		642292.438	1.1	10.0	bb
55	20241212_1_055	103-96h		1.000	3.32	666816.938		666816.938	1.1	14.2	bb
56	20241212_1_056	103-168h		1.000	3.32	649023.250		649023.250	1.1	11.1	bb
57	20241212_1_057	Solvent		1.000	3.30	779.521		779.521	0.0	-99.9	dd
58	20241212_1_058	Solvent		1.000	3.28	727.294		727.294	0.0	-99.9	bb
59	20241212_1_059	B	Blank	1.000	3.33	552.923		552.923	0.0	-99.9	bd
60	20241212_1_060	O	Blank	1.000	3.46	452.709		452.709	0.0	-99.9	bb
61	20241212_1_061	Q1	QC	1.000	3.32	693889.500		693889.500	1.2	18.8	bb
62	20241212_1_062	Q2	QC	1.000	3.32	703905.938		703905.938	1.2	20.5	bb
63	20241212_1_078	Q3	QC	1.000	3.31	245563.938		245563.938	0.4	-58.0	bb
64	20241212_1_079	Q4	QC	1.000	3.31	244274.516		244274.516	0.4	-58.2	bb

Dataset: D:\Data\27013-24001-NG.PRO\20241212_WBPD081_013_SA_Reinjection_Processed-Tu.qld

Last Altered: Tuesday, July 15, 2025 15:44:36 China Standard Time
Printed: Tuesday, July 15, 2025 15:46:23 China Standard Time

Compound name: Tolbutamide (1)

	Inj. Vol	Factor1	Vial
1	10.000	1.0	2:H,12
2	10.000	1.0	2:H,12
3	10.000	1.0	2:A,1
4	10.000	1.0	2:A,2
5	10.000	1.0	2:A,3
6	10.000	1.0	2:A,4
7	10.000	1.0	2:A,5
8	10.000	1.0	2:A,6
9	10.000	1.0	2:A,7
10	10.000	1.0	2:A,8
11	10.000	1.0	2:A,9
12	10.000	1.0	2:A,10
13	10.000	1.0	2:H,12
14	10.000	1.0	2:H,12
15	10.000	1.0	2:A,1
16	10.000	1.0	2:A,2
17	10.000	1.0	2:A,11
18	10.000	1.0	2:A,12
19	10.000	1.0	2:B,1
20	10.000	1.0	2:B,2
21	10.000	1.0	2:H,12
22	10.000	1.0	2:H,12
23	10.000	1.0	2:B,7
24	10.000	1.0	2:B,8
25	10.000	1.0	2:B,9
26	10.000	1.0	2:B,10
27	10.000	1.0	2:B,11
28	10.000	1.0	2:B,12
29	10.000	1.0	2:C,1
30	10.000	1.0	2:C,2
31	10.000	1.0	2:C,3
32	10.000	1.0	2:C,4
33	10.000	1.0	2:H,12
34	10.000	1.0	2:H,12
35	10.000	1.0	2:C,5
36	10.000	1.0	2:C,6
37	10.000	1.0	2:C,7
38	10.000	1.0	2:C,8
39	10.000	1.0	2:C,9
40	10.000	1.0	2:C,10
41	10.000	1.0	2:C,11
42	10.000	1.0	2:C,12
43	10.000	1.0	2:D,1
44	10.000	1.0	2:D,2
45	10.000	1.0	2:H,12
46	10.000	1.0	2:H,12
47	10.000	1.0	2:D,3
48	10.000	1.0	2:D,4
49	10.000	1.0	2:D,5
50	10.000	1.0	2:D,6
51	10.000	1.0	2:D,7

Dataset: D:\Data\27013-24001-NG.PRO\20241212_WBPD081_013_SA_Reinjection_Processed-Tu.qld

Last Altered: Tuesday, July 15, 2025 15:44:36 China Standard Time
Printed: Tuesday, July 15, 2025 15:46:23 China Standard Time

Compound name: Tolbutamide (1)

	Inj. Vol	Factor1	Vial
52	10.000	1.0	2:D,8
53	10.000	1.0	2:D,9
54	10.000	1.0	2:D,10
55	10.000	1.0	2:D,11
56	10.000	1.0	2:D,12
57	10.000	1.0	2:H,12
58	10.000	1.0	2:H,12
59	10.000	1.0	2:A,1
60	10.000	1.0	2:A,2
61	10.000	1.0	2:B,3
62	10.000	1.0	2:B,4
63	10.000	1.0	2:B,5
64	10.000	1.0	2:B,6

Dataset: D:\Data\27013-24001-NG.PRO\20241212_WBPD081_013_SA_Reinjection_Processed-Tu.qld

Last Altered: Tuesday, July 15, 2025 15:44:36 China Standard Time
Printed: Tuesday, July 15, 2025 15:46:23 China Standard Time

Method: D:\Data\27013-24001-NG.PRO\MethDB\20241212_WBPD081_013_yyb.mdb 13 Dec 2024 11:13:31
Calibration: 15 Jul 2025 15:44:36

Compound name: WBPD081_013 (4)
Correlation coefficient: r = 0.998955, r^2 = 0.997911
Calibration curve: 0.000106248 * x + 7.30001e-006
Response type: Internal Std (Ref 2), Area * (IS Conc. / IS Area)
Curve type: Linear, Origin: Exclude, Weighting: 1/x^2, Axis trans: None

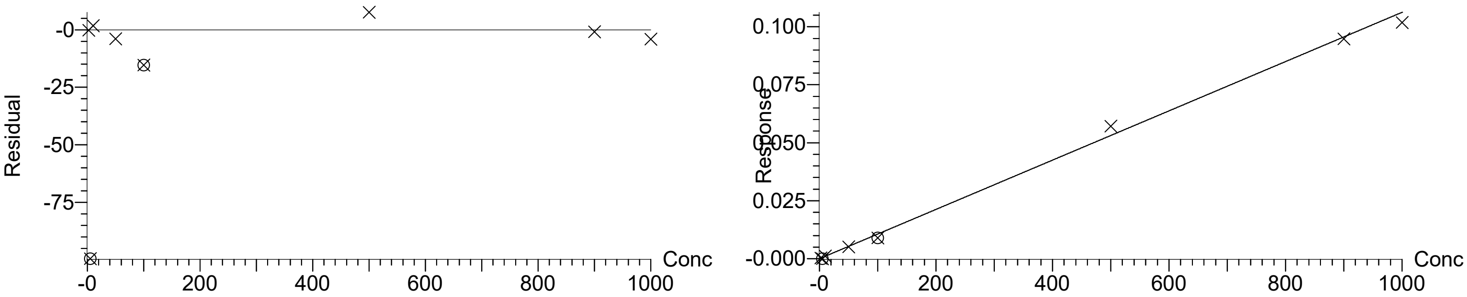

Compound name: Tolbutamide (1)
Response Factor: 584066
RRF SD: 236370, % Relative SD: 40.4697
Response type: External Std, Area
Curve type: RF

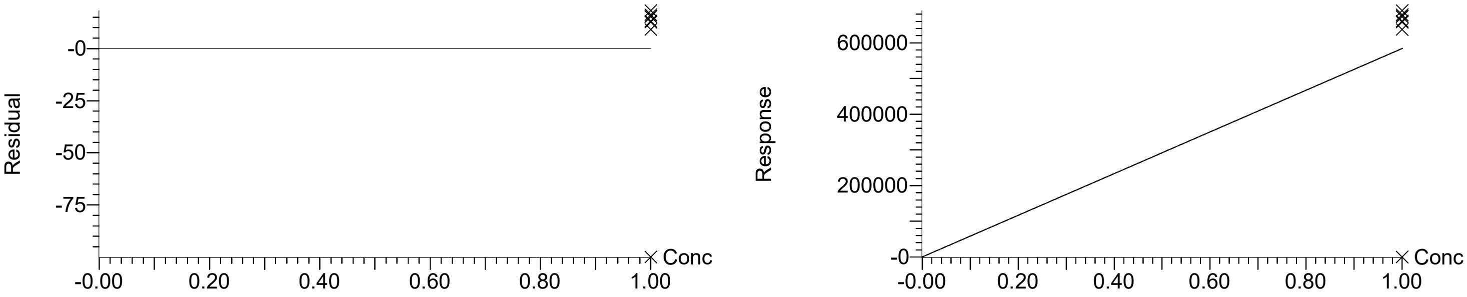

Dataset: D:\Data\27013-24001-NG.PRO\20241212_WBPD081_013_SA_Reinjection_Processed-Tu.qld

Last Altered: Tuesday, July 15, 2025 15:44:36 China Standard Time
Printed: Tuesday, July 15, 2025 15:46:23 China Standard Time

Method: D:\Data\27013-24001-NG.PRO\MethDB\20241212_WBPD081_013_yyb.mdb 13 Dec 2024 11:13:31
Calibration: 15 Jul 2025 15:44:36

Name: 20241212_1_001, ID: Solvent, Description:

WBPD081_013 (4)

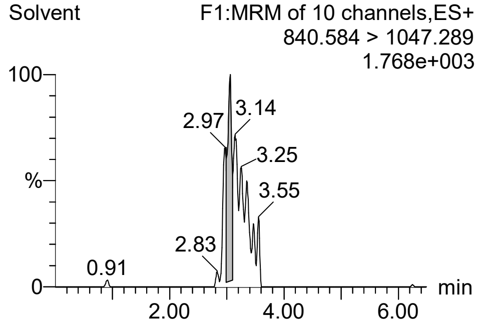

Tolbutamide (1)

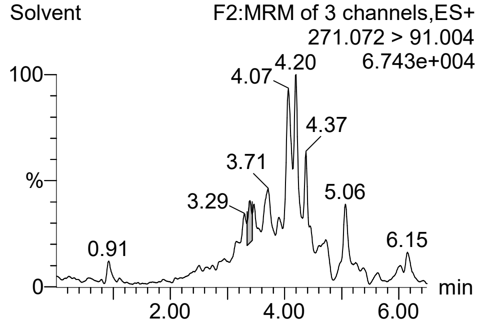

	#	Name	Trace	RT	Area	IS Area	Response	Primar...	Conc.	%Dev
1	1	WBPD081_013 (4)	840.584 > 1047....	3.06	148.147	951.956	0.156	dd	1464.7	
2	2	Tolbutamide (1)	271.072 > 91.004	3.39	951.956		951.956	dd	0.0	-99.8

Name: 20241212_1_002, ID: Solvent, Description:

WBPD081_013 (4)

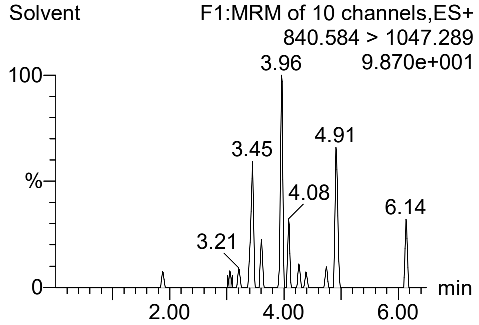

Tolbutamide (1)

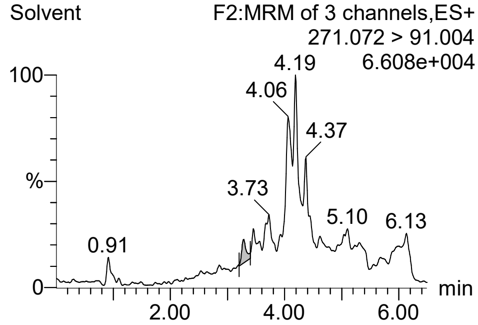

	#	Name	Trace	RT	Area	IS Area	Response	Primar...	Conc.	%Dev
1	1	WBPD081_013 (4)	840.584 > 1047....	3.05	0.335	668.182	0.001	bb	4.7	
2	2	Tolbutamide (1)	271.072 > 91.004	3.28	668.182		668.182	dd	0.0	-99.9

Name: 20241212_1_003, ID: B, Description:

WBPD081_013 (4)

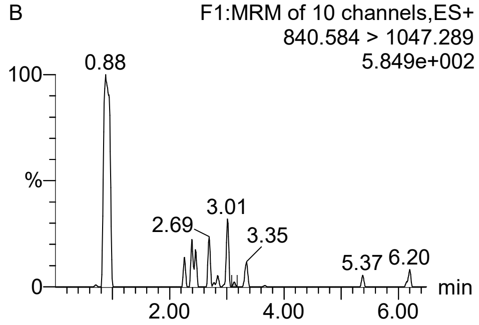

Tolbutamide (1)

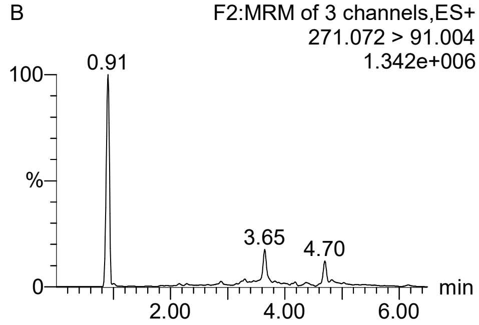

Dataset: D:\Data\27013-24001-NG.PRO\20241212_WBPD081_013_SA_Reinjection_Processed-Tu.qld

Last Altered: Tuesday, July 15, 2025 15:44:36 China Standard Time
Printed: Tuesday, July 15, 2025 15:46:23 China Standard Time

Name: 20241212_1_003, ID: B, Description:

	#	Name	Trace	RT	Area	IS Area	Response	Primar...	Conc.	%Dev
1	1	WBPD081_013 (4)	840.584 > 1047....	3.13	0.581			bb		
2	2	Tolbutamide (1)	271.072 > 91.004							

Name: 20241212_1_004, ID: O, Description:

WBPD081_013 (4)

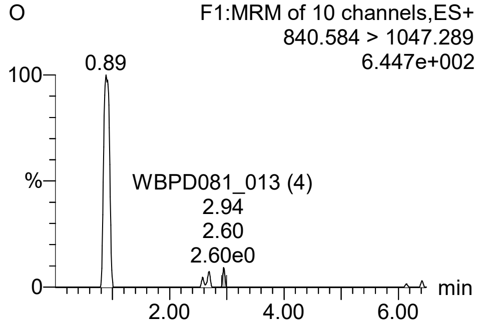

Tolbutamide (1)

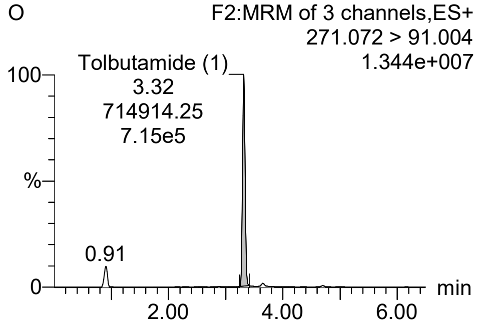

	#	Name	Trace	RT	Area	IS Area	Response	Primar...	Conc.	%Dev
1	1	WBPD081_013 (4)	840.584 > 1047....	2.94	2.603	714914.250	0.000	bbI		
2	2	Tolbutamide (1)	271.072 > 91.004	3.32	714914.250		714914.250	bb	1.2	22.4

Name: 20241212_1_005, ID: STD1, Description: WBPD081_013

WBPD081_013 (4)

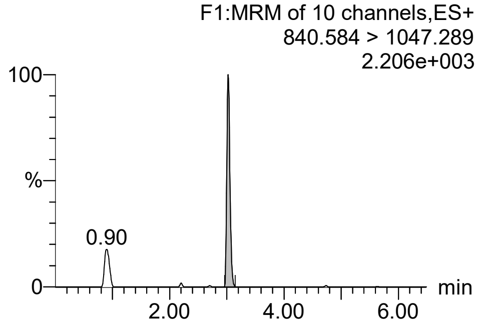

Tolbutamide (1)

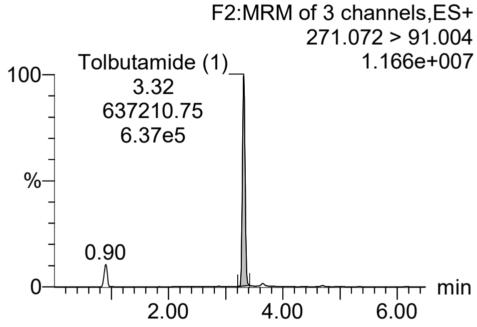

	#	Name	Trace	RT	Area	IS Area	Response	Primar...	Conc.	%Dev
1	1	WBPD081_013 (4)	840.584 > 1047....	3.02	139.793	637210.750	0.000	bb	2.0	-0.2
2	2	Tolbutamide (1)	271.072 > 91.004	3.32	637210.750		637210.750	bb	1.1	9.1

Name: 20241212_1_006, ID: STD2, Description: WBPD081_013

Dataset: D:\Data\27013-24001-NG.PRO\20241212_WBPD081_013_SA_Reinjection_Processed-Tu.qld

Last Altered: Tuesday, July 15, 2025 15:44:36 China Standard Time
Printed: Tuesday, July 15, 2025 15:46:23 China Standard Time

Name: 20241212_1_006, ID: STD2, Description: WBPD081_013

WBPD081_013 (4)

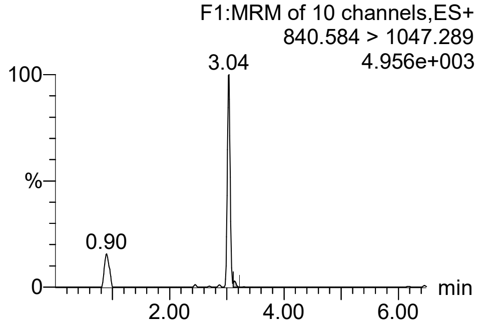

Tolbutamide (1)

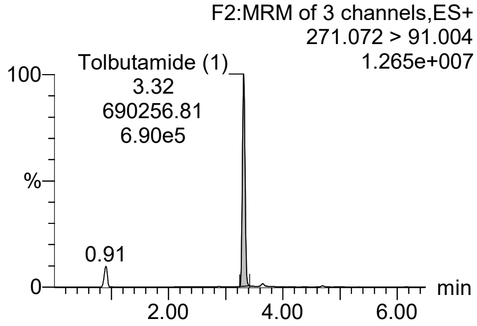

	# Name	Trace	RT	Area	IS Area	Response	Primar...	Conc.	%Dev
1	1 WBPD081_013 (4)	840.584 > 1047....	3.13	7.128	690256.813	0.000	dbX	0.0	-99.4
2	2 Tolbutamide (1)	271.072 > 91.004	3.32	690256.813		690256.813	bb	1.2	18.2

Name: 20241212_1_007, ID: STD3, Description: WBPD081_013

WBPD081_013 (4)

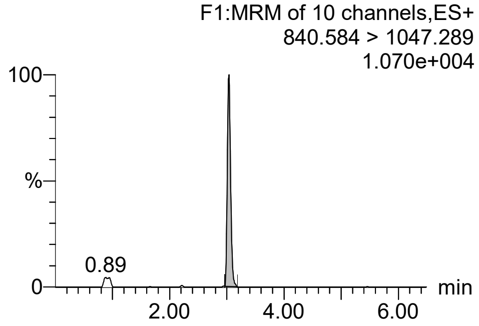

Tolbutamide (1)

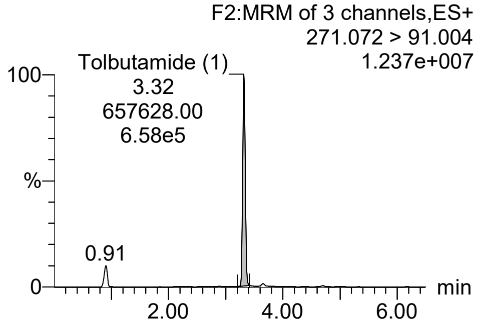

	# Name	Trace	RT	Area	IS Area	Response	Primar...	Conc.	%Dev
1	1 WBPD081_013 (4)	840.584 > 1047....	3.04	715.224	657628.000	0.001	bb	10.2	1.7
2	2 Tolbutamide (1)	271.072 > 91.004	3.32	657628.000		657628.000	bb	1.1	12.6

Name: 20241212_1_008, ID: STD4, Description: WBPD081_013

WBPD081_013 (4)

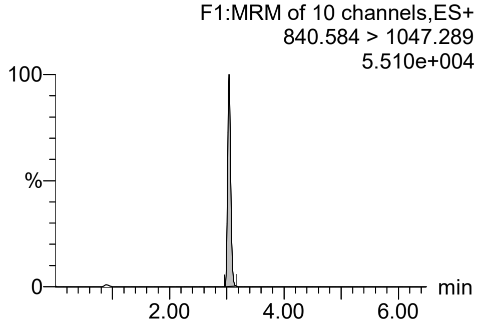

Tolbutamide (1)

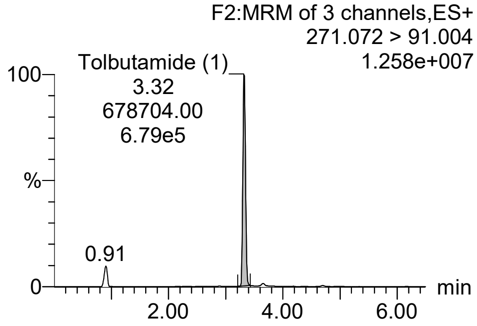

	# Name	Trace	RT	Area	IS Area	Response	Primar...	Conc.	%Dev
1	1 WBPD081_013 (4)	840.584 > 1047....	3.04	3465.741	678704.000	0.005	bb	48.0	-4.0
2	2 Tolbutamide (1)	271.072 > 91.004	3.32	678704.000		678704.000	bb	1.2	16.2

Dataset: D:\Data\27013-24001-NG.PRO\20241212_WBPD081_013_SA_Reinjection_Processed-Tu.qld

Last Altered: Tuesday, July 15, 2025 15:44:36 China Standard Time
Printed: Tuesday, July 15, 2025 15:46:23 China Standard Time

Name: 20241212_1_009, ID: STD5, Description: WBPD081_013

WBPD081_013 (4)

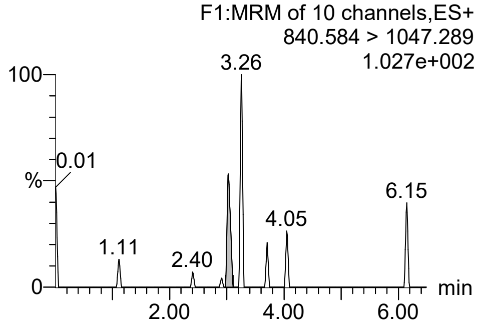

Tolbutamide (1)

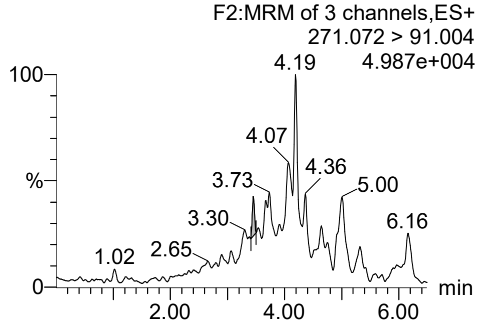

	# Name	Trace	RT	Area	IS Area	Response	Primar...	Conc.	%Dev
1	1 WBPD081_013 (4)	840.584 > 1047....	3.03	3.741	415.610	0.009	bbX	84.7	-15.3
2	2 Tolbutamide (1)	271.072 > 91.004	3.45	415.610		415.610	bb	0.0	-99.9

Name: 20241212_1_010, ID: STD6, Description: WBPD081_013

WBPD081_013 (4)

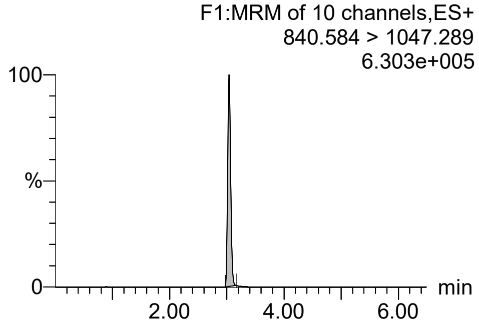

Tolbutamide (1)

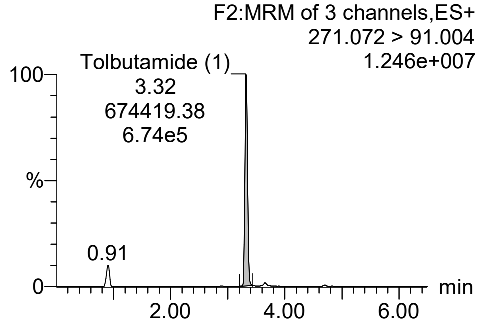

	# Name	Trace	RT	Area	IS Area	Response	Primar...	Conc.	%Dev
1	1 WBPD081_013 (4)	840.584 > 1047....	3.04	38548.813	674419.375	0.057	bb	537.9	7.6
2	2 Tolbutamide (1)	271.072 > 91.004	3.32	674419.375		674419.375	bb	1.2	15.5

Name: 20241212_1_011, ID: STD7, Description: WBPD081_013

WBPD081_013 (4)

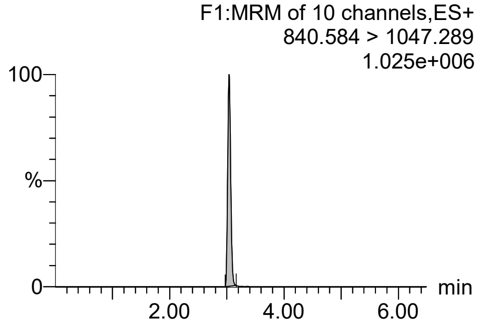

Tolbutamide (1)

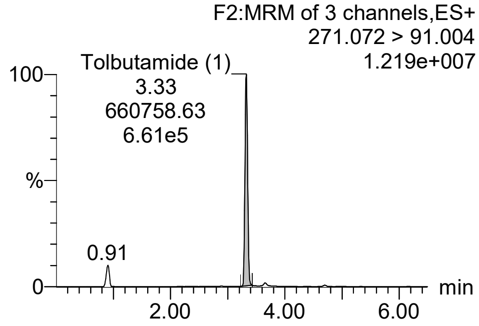

	# Name	Trace	RT	Area	IS Area	Response	Primar...	Conc.	%Dev
1	1 WBPD081_013 (4)	840.584 > 1047....	3.04	62607.488	660758.625	0.095	bb	891.7	-0.9
2	2 Tolbutamide (1)	271.072 > 91.004	3.33	660758.625		660758.625	bb	1.1	13.1

Dataset: D:\Data\27013-24001-NG.PRO\20241212_WBPD081_013_SA_Reinjection_Processed-Tu.qld

Last Altered: Tuesday, July 15, 2025 15:44:36 China Standard Time
Printed: Tuesday, July 15, 2025 15:46:23 China Standard Time

Name: 20241212_1_012, ID: STD8, Description: WBPD081_013

WBPD081_013 (4)

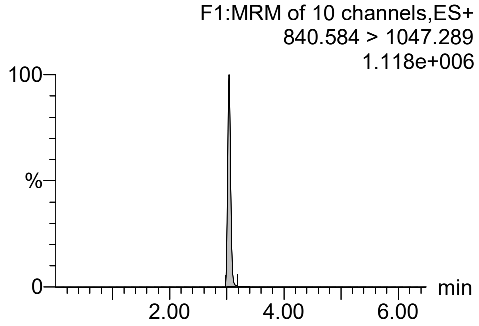

Tolbutamide (1)

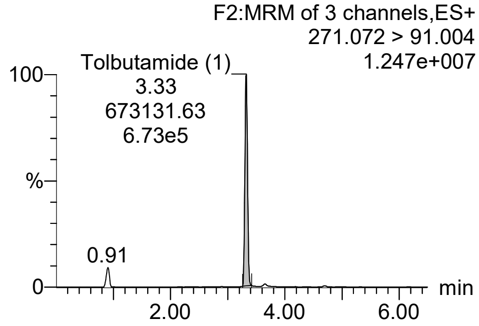

	#	Name	Trace	RT	Area	IS Area	Response	Primar...	Conc.	%Dev
1	1	WBPD081_013 (4)	840.584 > 1047....	3.04	68572.266	673131.625	0.102	bb	958.7	-4.1
2	2	Tolbutamide (1)	271.072 > 91.004	3.33	673131.625		673131.625	bb	1.2	15.2

Name: 20241212_1_013, ID: Solvent, Description:

WBPD081_013 (4)

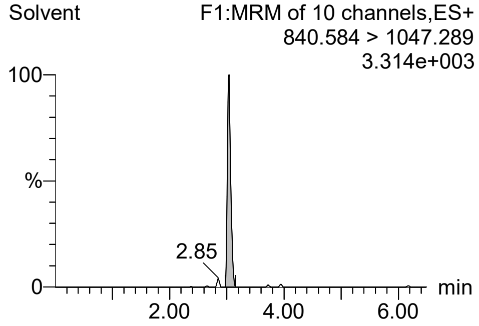

Tolbutamide (1)

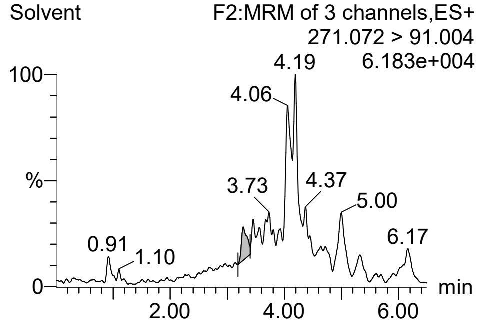

	#	Name	Trace	RT	Area	IS Area	Response	Primar...	Conc.	%Dev
1	1	WBPD081_013 (4)	840.584 > 1047....	3.04	226.198	1079.635	0.210	bb	1971.9	
2	2	Tolbutamide (1)	271.072 > 91.004	3.28	1079.635		1079.635	bd	0.0	-99.8

Name: 20241212_1_014, ID: Solvent, Description:

WBPD081_013 (4)

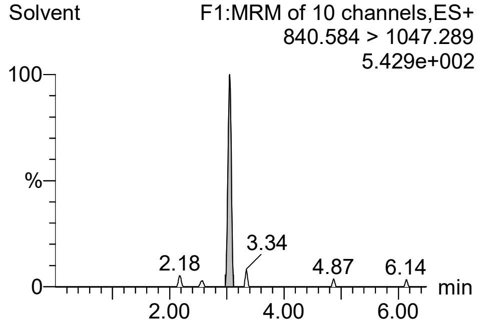

Tolbutamide (1)

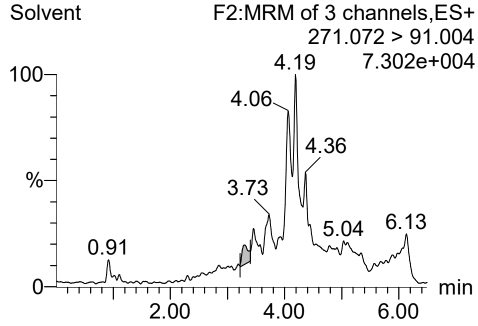

	#	Name	Trace	RT	Area	IS Area	Response	Primar...	Conc.	%Dev
1	1	WBPD081_013 (4)	840.584 > 1047....	3.05	37.265	833.903	0.045	bb	420.5	
2	2	Tolbutamide (1)	271.072 > 91.004	3.30	833.903		833.903	dd	0.0	-99.9

Dataset: D:\Data\27013-24001-NG.PRO\20241212_WBPD081_013_SA_Reinjection_Processed-Tu.qld

Last Altered: Tuesday, July 15, 2025 15:44:36 China Standard Time
Printed: Tuesday, July 15, 2025 15:46:23 China Standard Time

Name: 20241212_1_015, ID: B, Description:

WBPD081_013 (4)

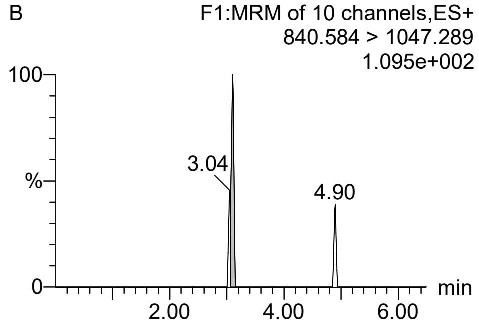

Tolbutamide (1)

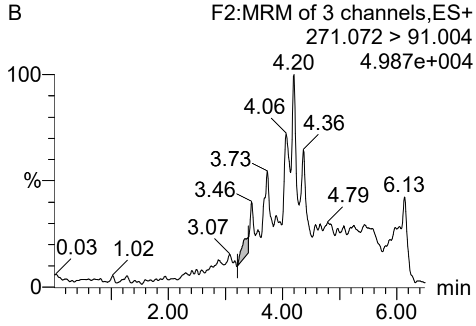

	# Name	Trace	RT	Area	IS Area	Response	Primar...	Conc.	%Dev
1	1 WBPD081_013 (4)	840.584 > 1047....	3.10	5.481	572.221	0.010	db	90.1	
2	2 Tolbutamide (1)	271.072 > 91.004	3.40	572.221		572.221	bd	0.0	-99.9

Name: 20241212_1_016, ID: O, Description:

WBPD081_013 (4)

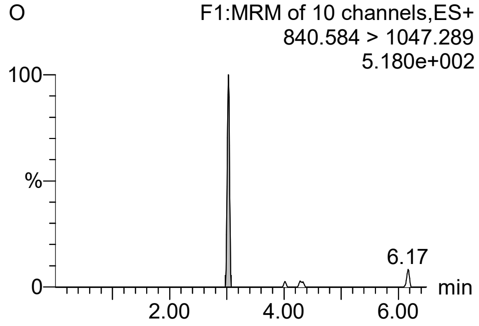

Tolbutamide (1)

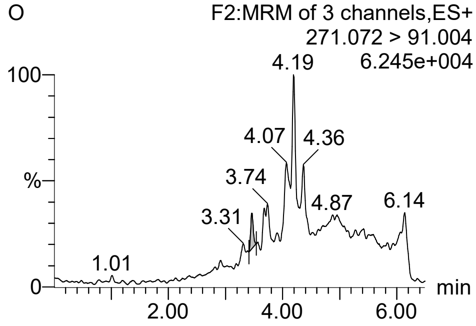

	# Name	Trace	RT	Area	IS Area	Response	Primar...	Conc.	%Dev
1	1 WBPD081_013 (4)	840.584 > 1047....	3.03	24.326	550.533	0.044	bb	415.8	
2	2 Tolbutamide (1)	271.072 > 91.004	3.47	550.533		550.533	bb	0.0	-99.9

Name: 20241212_1_017, ID: Q1, Description:

WBPD081_013 (4)

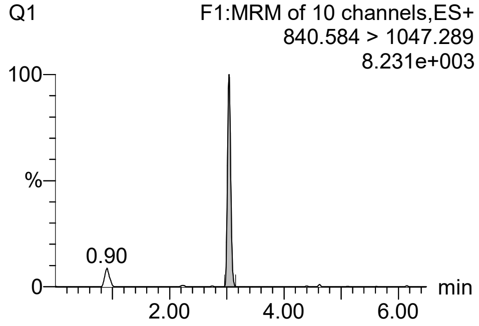

Tolbutamide (1)

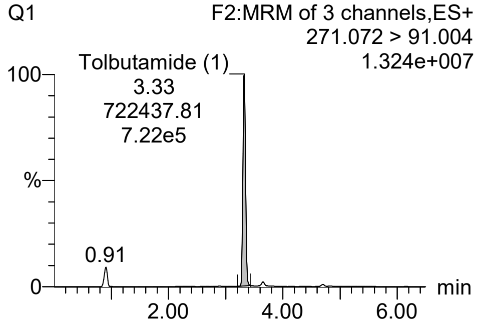

	# Name	Trace	RT	Area	IS Area	Response	Primar...	Conc.	%Dev
1	1 WBPD081_013 (4)	840.584 > 1047....	3.04	544.577	722437.813	0.001	bb	7.0	17.1
2	2 Tolbutamide (1)	271.072 > 91.004	3.33	722437.813		722437.813	bb	1.2	23.7

Dataset: D:\Data\27013-24001-NG.PRO\20241212_WBPD081_013_SA_Reinjection_Processed-Tu.qld

Last Altered: Tuesday, July 15, 2025 15:44:36 China Standard Time
Printed: Tuesday, July 15, 2025 15:46:23 China Standard Time

Name: 20241212_1_018, ID: Q2, Description:

WBPD081_013 (4)

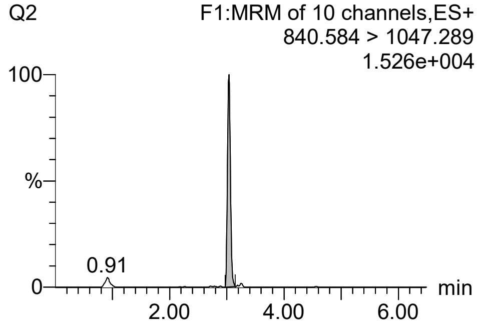

Tolbutamide (1)

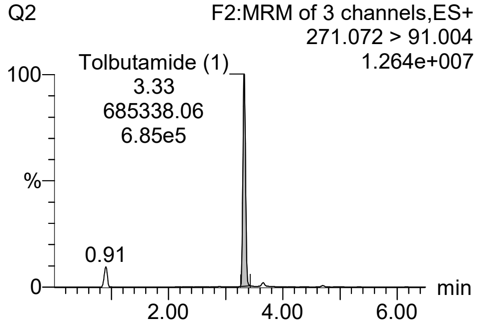

	#	Name	Trace	RT	Area	IS Area	Response	Primar...	Conc.	%Dev
1	1	WBPD081_013 (4)	840.584 > 1047....	3.04	924.185	685338.063	0.001	bb	12.6	5.2
2	2	Tolbutamide (1)	271.072 > 91.004	3.33	685338.063		685338.063	bb	1.2	17.3

Name: 20241212_1_019, ID: Q3, Description:

WBPD081_013 (4)

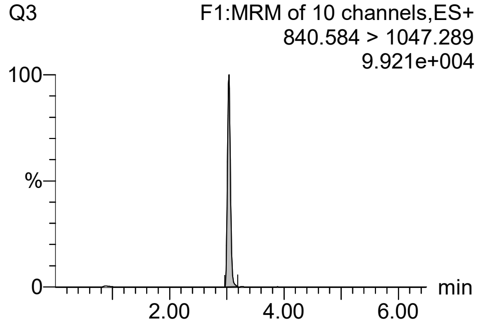

Tolbutamide (1)

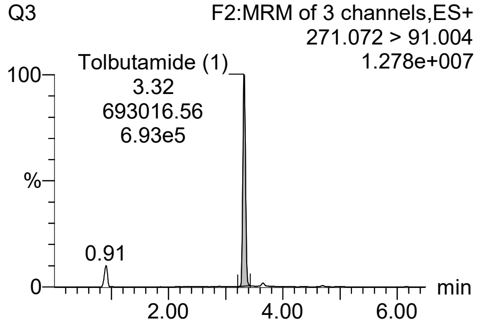

	#	Name	Trace	RT	Area	IS Area	Response	Primar...	Conc.	%Dev
1	1	WBPD081_013 (4)	840.584 > 1047....	3.04	6061.994	693016.563	0.009	bb	82.3	2.8
2	2	Tolbutamide (1)	271.072 > 91.004	3.32	693016.563		693016.563	bb	1.2	18.7

Name: 20241212_1_020, ID: Q4, Description:

WBPD081_013 (4)

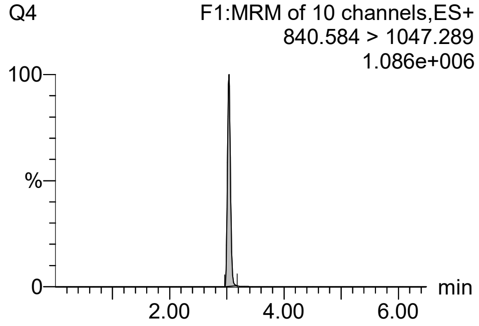

Tolbutamide (1)

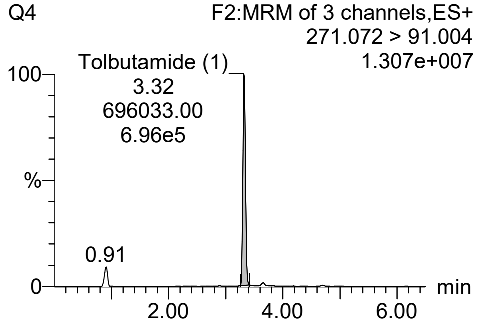

	#	Name	Trace	RT	Area	IS Area	Response	Primar...	Conc.	%Dev
1	1	WBPD081_013 (4)	840.584 > 1047....	3.04	66168.148	696033.000	0.095	bb	894.7	11.8
2	2	Tolbutamide (1)	271.072 > 91.004	3.32	696033.000		696033.000	bb	1.2	19.2

Dataset: D:\Data\27013-24001-NG.PRO\20241212_WBPD081_013_SA_Reinjection_Processed-Tu.qld

Last Altered: Tuesday, July 15, 2025 15:44:36 China Standard Time
Printed: Tuesday, July 15, 2025 15:46:23 China Standard Time

Name: 20241212_1_021, ID: Solvent, Description:

WBPD081_013 (4)

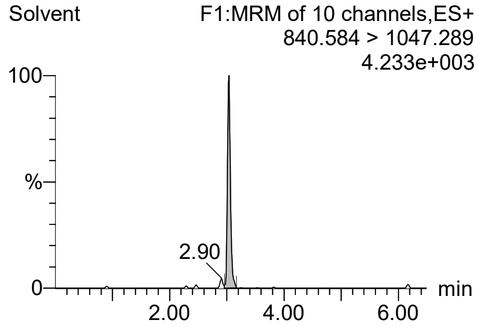

Tolbutamide (1)

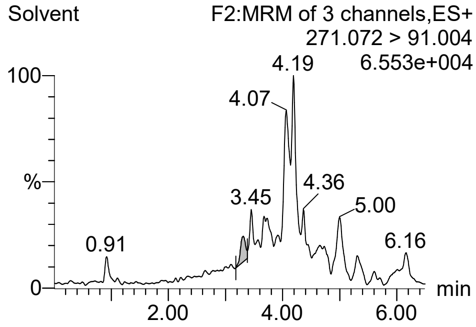

	#	Name	Trace	RT	Area	IS Area	Response	Primar...	Conc.	%Dev
1	1	WBPD081_013 (4)	840.584 > 1047....	3.04	256.707	811.893	0.316	db	2975.8	
2	2	Tolbutamide (1)	271.072 > 91.004	3.31	811.893		811.893	bd	0.0	-99.9

Name: 20241212_1_022, ID: Solvent, Description:

WBPD081_013 (4)

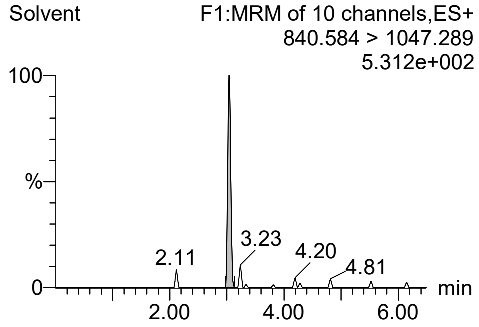

Tolbutamide (1)

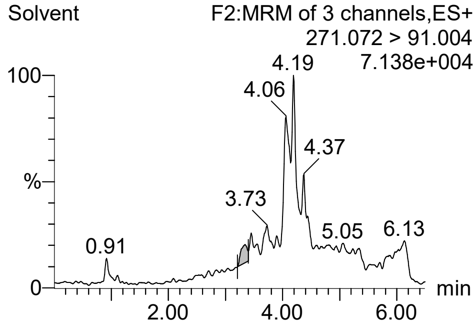

	#	Name	Trace	RT	Area	IS Area	Response	Primar...	Conc.	%Dev
1	1	WBPD081_013 (4)	840.584 > 1047....	3.04	33.200	804.311	0.041	bb	388.4	
2	2	Tolbutamide (1)	271.072 > 91.004	3.34	804.311		804.311	bd	0.0	-99.9

Name: 20241212_1_023, ID: 101-Predose, Description:

WBPD081_013 (4)

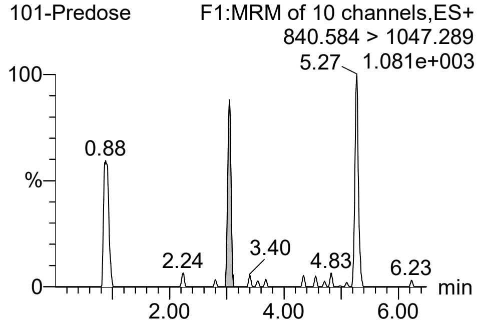

Tolbutamide (1)

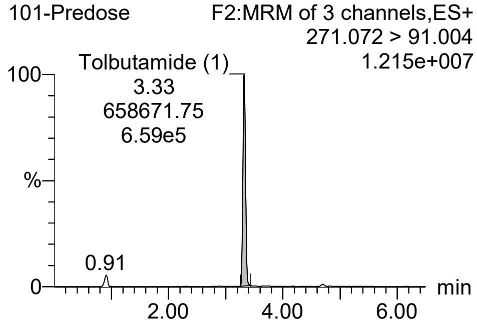

	#	Name	Trace	RT	Area	IS Area	Response	Primar...	Conc.	%Dev
1	1	WBPD081_013 (4)	840.584 > 1047....	3.04	64.994	658671.750	0.000	bb	0.9	
2	2	Tolbutamide (1)	271.072 > 91.004	3.33	658671.750		658671.750	bb	1.1	12.8

Dataset: D:\Data\27013-24001-NG.PRO\20241212_WBPD081_013_SA_Reinjection_Processed-Tu.qld

Last Altered: Tuesday, July 15, 2025 15:44:36 China Standard Time
Printed: Tuesday, July 15, 2025 15:46:23 China Standard Time

Name: 20241212_1_024, ID: 101-2h, Description:

WBPD081_013 (4)

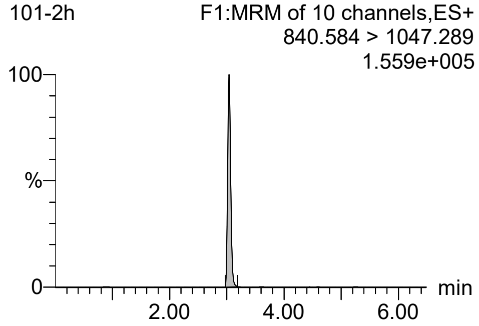

Tolbutamide (1)

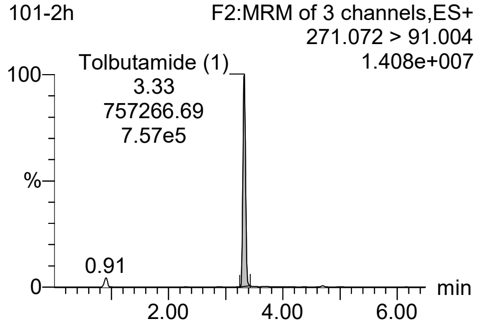

	# Name	Trace	RT	Area	IS Area	Response	Primar...	Conc.	%Dev
1	1 WBPD081_013 (4)	840.584 > 1047....	3.04	9690.713	757266.688	0.013	bb	120.4	
2	2 Tolbutamide (1)	271.072 > 91.004	3.33	757266.688		757266.688	bb	1.3	29.7

Name: 20241212_1_025, ID: 101-4h, Description:

WBPD081_013 (4)

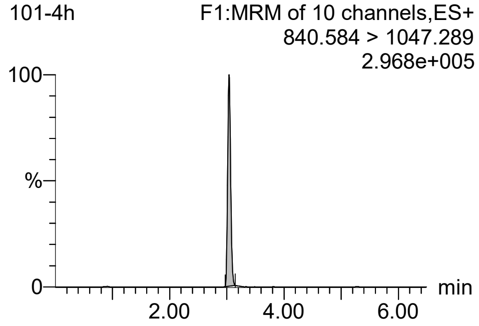

Tolbutamide (1)

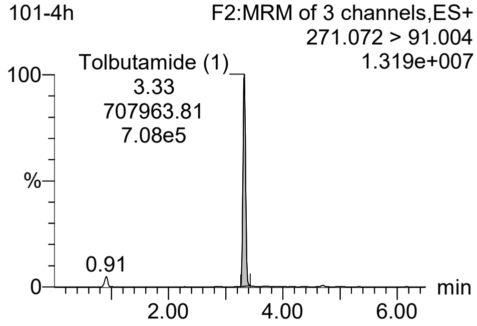

	# Name	Trace	RT	Area	IS Area	Response	Primar...	Conc.	%Dev
1	1 WBPD081_013 (4)	840.584 > 1047....	3.04	17956.346	707963.813	0.025	bb	238.6	
2	2 Tolbutamide (1)	271.072 > 91.004	3.33	707963.813		707963.813	bb	1.2	21.2

Name: 20241212_1_026, ID: 101-8h, Description:

WBPD081_013 (4)

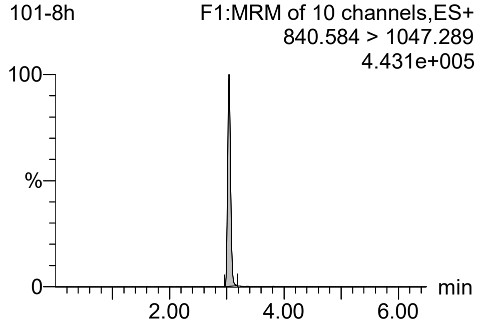

Tolbutamide (1)

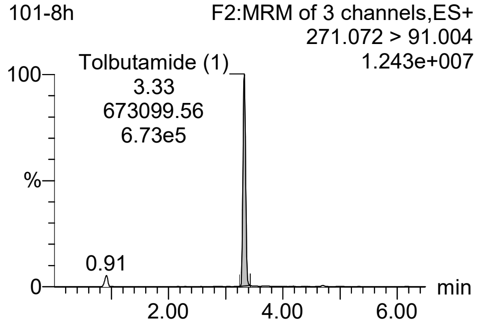

	# Name	Trace	RT	Area	IS Area	Response	Primar...	Conc.	%Dev
1	1 WBPD081_013 (4)	840.584 > 1047....	3.04	26826.949	673099.563	0.040	bb	375.1	
2	2 Tolbutamide (1)	271.072 > 91.004	3.33	673099.563		673099.563	bb	1.2	15.2

Dataset: D:\Data\27013-24001-NG.PRO\20241212_WBPD081_013_SA_Reinjection_Processed-Tu.qld

Last Altered: Tuesday, July 15, 2025 15:44:36 China Standard Time
Printed: Tuesday, July 15, 2025 15:46:23 China Standard Time

Name: 20241212_1_027, ID: 101-12h, Description:

WBPD081_013 (4)

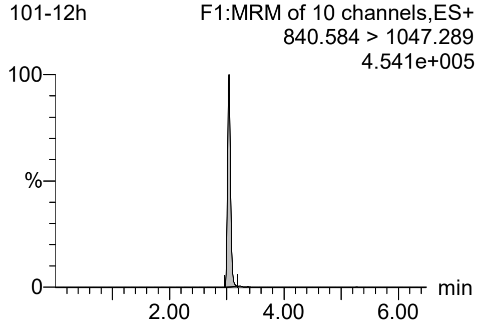

Tolbutamide (1)

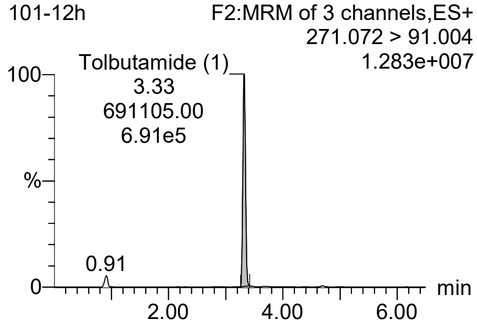

	#	Name	Trace	RT	Area	IS Area	Response	Primar...	Conc.	%Dev
1	1	WBPD081_013 (4)	840.584 > 1047....	3.04	27711.328	691105.000	0.040	bb	377.3	
2	2	Tolbutamide (1)	271.072 > 91.004	3.33	691105.000		691105.000	bb	1.2	18.3

Name: 20241212_1_028, ID: 101-24h, Description:

WBPD081_013 (4)

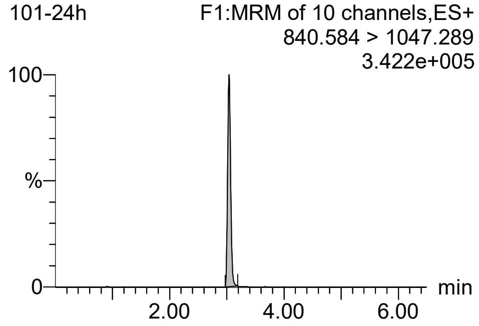

Tolbutamide (1)

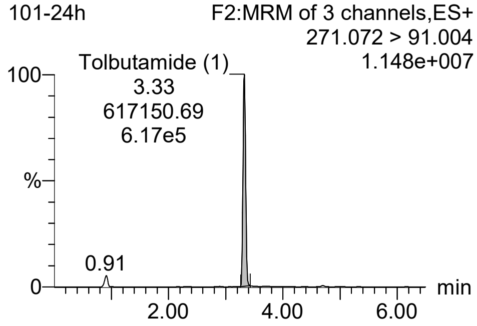

	#	Name	Trace	RT	Area	IS Area	Response	Primar...	Conc.	%Dev
1	1	WBPD081_013 (4)	840.584 > 1047....	3.04	21057.592	617150.688	0.034	bb	321.1	
2	2	Tolbutamide (1)	271.072 > 91.004	3.33	617150.688		617150.688	bb	1.1	5.7

Name: 20241212_1_029, ID: 101-48h, Description:

WBPD081_013 (4)

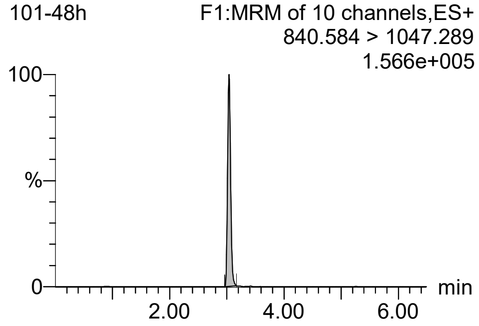

Tolbutamide (1)

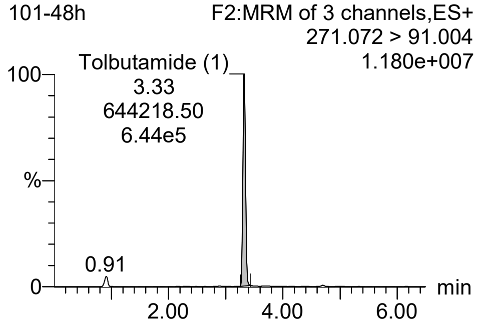

	#	Name	Trace	RT	Area	IS Area	Response	Primar...	Conc.	%Dev
1	1	WBPD081_013 (4)	840.584 > 1047....	3.04	9629.165	644218.500	0.015	bb	140.6	
2	2	Tolbutamide (1)	271.072 > 91.004	3.33	644218.500		644218.500	bb	1.1	10.3

Dataset: D:\Data\27013-24001-NG.PRO\20241212_WBPD081_013_SA_Reinjection_Processed-Tu.qld

Last Altered: Tuesday, July 15, 2025 15:44:36 China Standard Time
Printed: Tuesday, July 15, 2025 15:46:23 China Standard Time

Name: 20241212_1_030, ID: 101-72h, Description:

WBPD081_013 (4)

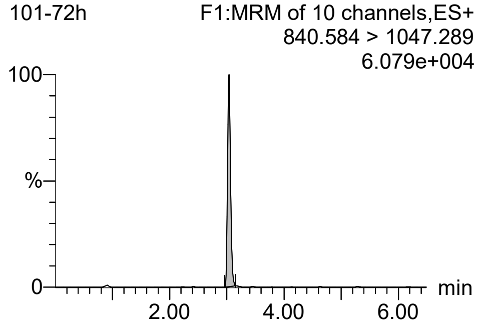

Tolbutamide (1)

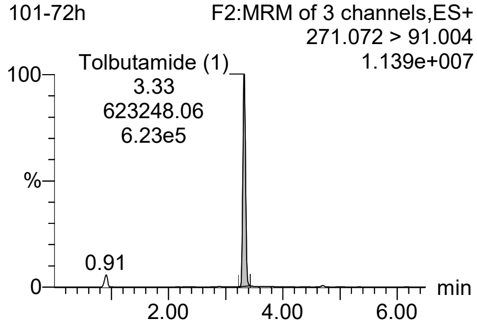

	#	Name	Trace	RT	Area	IS Area	Response	Primar...	Conc.	%Dev
1	1	WBPD081_013 (4)	840.584 > 1047....	3.04	3718.726	623248.063	0.006	bb	56.1	
2	2	Tolbutamide (1)	271.072 > 91.004	3.33	623248.063		623248.063	bb	1.1	6.7

Name: 20241212_1_031, ID: 101-96h, Description:

WBPD081_013 (4)

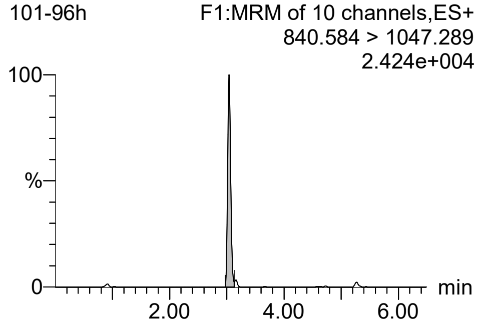

Tolbutamide (1)

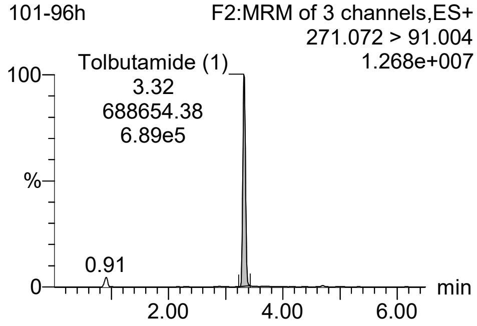

	#	Name	Trace	RT	Area	IS Area	Response	Primar...	Conc.	%Dev
1	1	WBPD081_013 (4)	840.584 > 1047....	3.04	1514.152	688654.375	0.002	bd	20.6	
2	2	Tolbutamide (1)	271.072 > 91.004	3.32	688654.375		688654.375	bb	1.2	17.9

Name: 20241212_1_032, ID: 101-168h, Description:

WBPD081_013 (4)

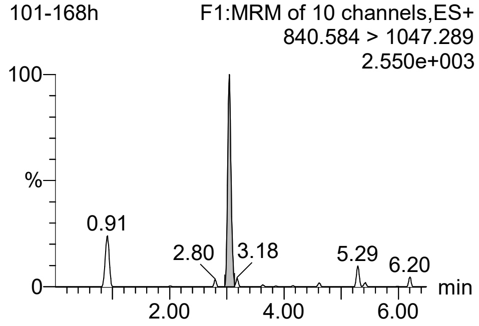

Tolbutamide (1)

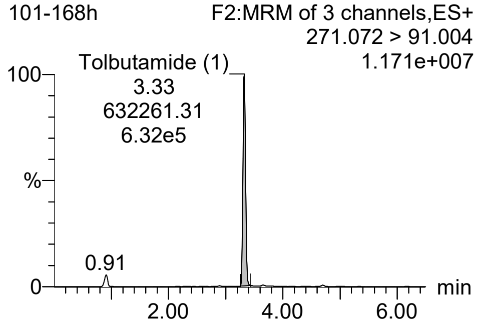

	#	Name	Trace	RT	Area	IS Area	Response	Primar...	Conc.	%Dev
1	1	WBPD081_013 (4)	840.584 > 1047....	3.04	175.776	632261.313	0.000	bd	2.5	
2	2	Tolbutamide (1)	271.072 > 91.004	3.33	632261.313		632261.313	bb	1.1	8.3

Dataset: D:\Data\27013-24001-NG.PRO\20241212_WBPD081_013_SA_Reinjection_Processed-Tu.qld

Last Altered: Tuesday, July 15, 2025 15:44:36 China Standard Time
Printed: Tuesday, July 15, 2025 15:46:23 China Standard Time

Name: 20241212_1_033, ID: Solvent, Description:

WBPD081_013 (4)

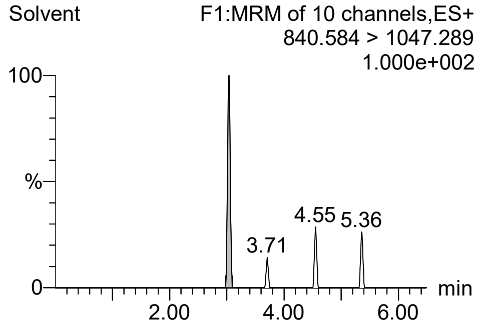

Tolbutamide (1)

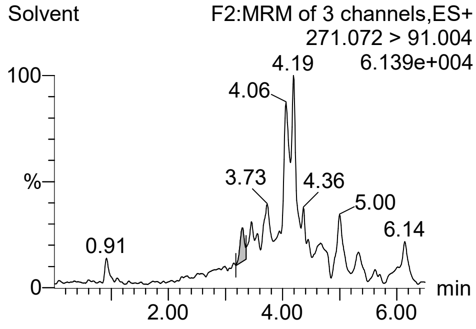

#	Name	Trace	RT	Area	IS Area	Response	Primar...	Conc.	%Dev
1	1 WBPD081_013 (4)	840.584 > 1047....	3.04	5.648	865.394	0.007	bb	61.4	
2	2 Tolbutamide (1)	271.072 > 91.004	3.29	865.394		865.394	dd	0.0	-99.9

Name: 20241212_1_034, ID: Solvent, Description:

WBPD081_013 (4)

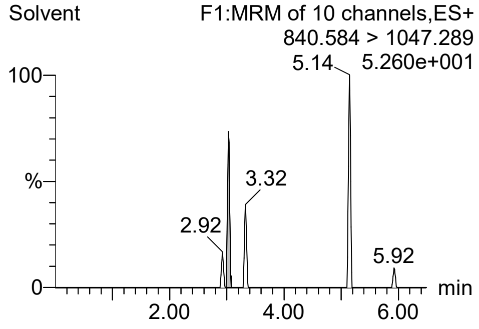

Tolbutamide (1)

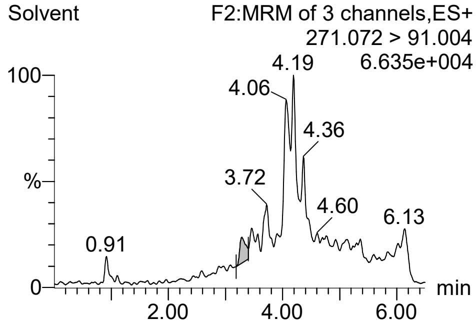

#	Name	Trace	RT	Area	IS Area	Response	Primar...	Conc.	%Dev
1	1 WBPD081_013 (4)	840.584 > 1047....	3.03	1.704	975.246	0.002	bb	16.4	
2	2 Tolbutamide (1)	271.072 > 91.004	3.28	975.246		975.246	bd	0.0	-99.8

Name: 20241212_1_035, ID: 102-Predose, Description:

WBPD081_013 (4)

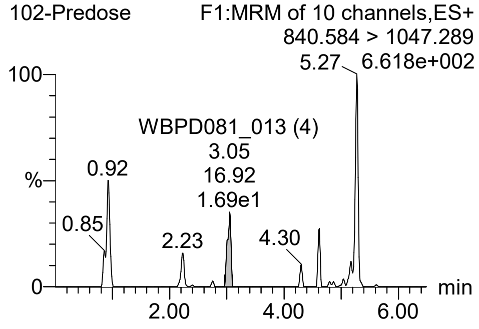

Tolbutamide (1)

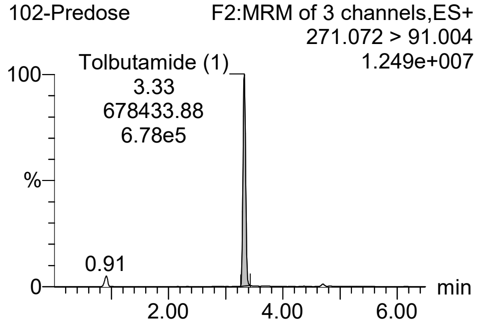

#	Name	Trace	RT	Area	IS Area	Response	Primar...	Conc.	%Dev
1	1 WBPD081_013 (4)	840.584 > 1047....	3.05	16.923	678433.875	0.000	bb	0.2	
2	2 Tolbutamide (1)	271.072 > 91.004	3.33	678433.875		678433.875	bb	1.2	16.2

Dataset: D:\Data\27013-24001-NG.PRO\20241212_WBPD081_013_SA_Reinjection_Processed-Tu.qld

Last Altered: Tuesday, July 15, 2025 15:44:36 China Standard Time
Printed: Tuesday, July 15, 2025 15:46:23 China Standard Time

Name: 20241212_1_036, ID: 102-2h, Description:

WBPD081_013 (4)

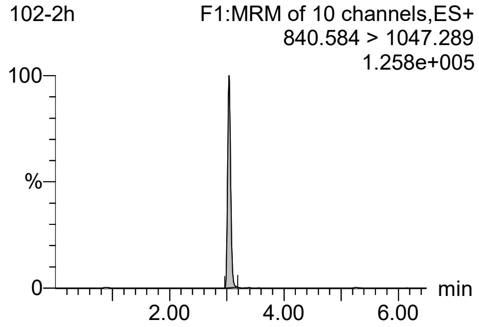

Tolbutamide (1)

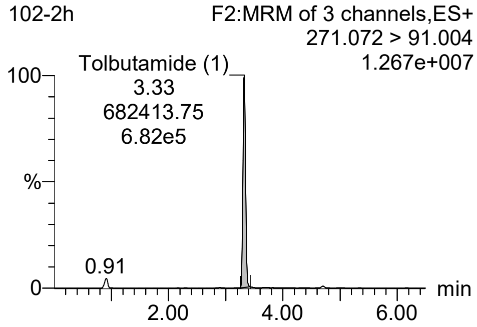

	#	Name	Trace	RT	Area	IS Area	Response	Primar...	Conc.	%Dev
1	1	WBPD081_013 (4)	840.584 > 1047....	3.04	7769.788	682413.750	0.011	bb	107.1	
2	2	Tolbutamide (1)	271.072 > 91.004	3.33	682413.750		682413.750	bb	1.2	16.8

Name: 20241212_1_037, ID: 102-4h, Description:

WBPD081_013 (4)

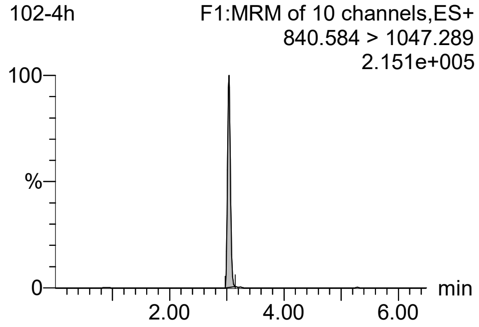

Tolbutamide (1)

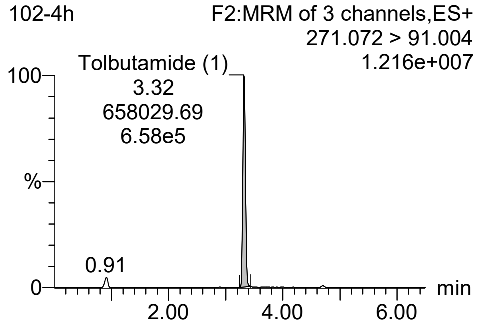

	#	Name	Trace	RT	Area	IS Area	Response	Primar...	Conc.	%Dev
1	1	WBPD081_013 (4)	840.584 > 1047....	3.04	12721.282	658029.688	0.019	bb	181.9	
2	2	Tolbutamide (1)	271.072 > 91.004	3.32	658029.688		658029.688	bb	1.1	12.7

Name: 20241212_1_038, ID: 102-8h, Description:

WBPD081_013 (4)

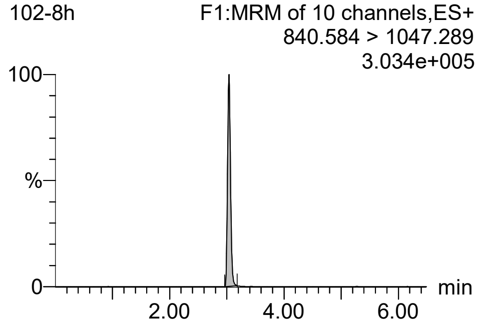

Tolbutamide (1)

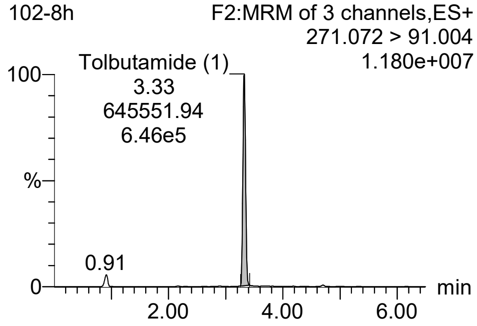

	#	Name	Trace	RT	Area	IS Area	Response	Primar...	Conc.	%Dev
1	1	WBPD081_013 (4)	840.584 > 1047....	3.04	18223.262	645551.938	0.028	bb	265.6	
2	2	Tolbutamide (1)	271.072 > 91.004	3.33	645551.938		645551.938	bb	1.1	10.5

Dataset: D:\Data\27013-24001-NG.PRO\20241212_WBPD081_013_SA_Reinjection_Processed-Tu.qld

Last Altered: Tuesday, July 15, 2025 15:44:36 China Standard Time
Printed: Tuesday, July 15, 2025 15:46:23 China Standard Time

Name: 20241212_1_039, ID: 102-12h, Description:

WBPD081_013 (4)

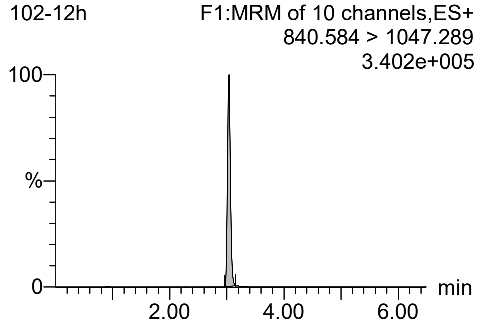

Tolbutamide (1)

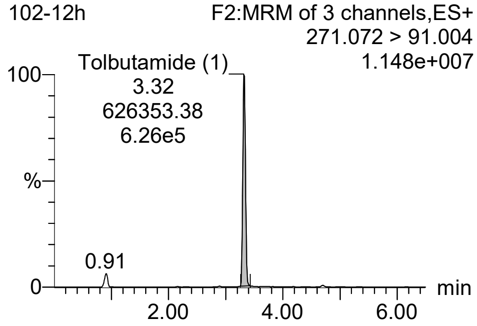

	#	Name	Trace	RT	Area	IS Area	Response	Primar...	Conc.	%Dev
1	1	WBPD081_013 (4)	840.584 > 1047....	3.04	20340.768	626353.375	0.032	bb	305.6	
2	2	Tolbutamide (1)	271.072 > 91.004	3.32	626353.375		626353.375	bb	1.1	7.2

Name: 20241212_1_040, ID: 102-24h, Description:

WBPD081_013 (4)

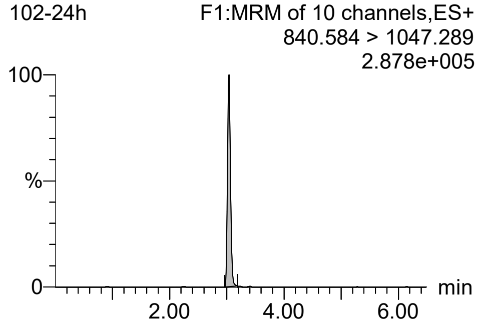

Tolbutamide (1)

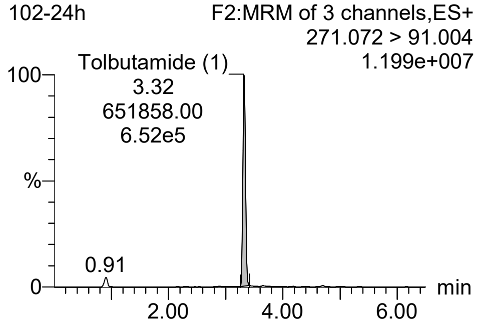

	#	Name	Trace	RT	Area	IS Area	Response	Primar...	Conc.	%Dev
1	1	WBPD081_013 (4)	840.584 > 1047....	3.04	17606.041	651858.000	0.027	bb	254.1	
2	2	Tolbutamide (1)	271.072 > 91.004	3.32	651858.000		651858.000	bb	1.1	11.6

Name: 20241212_1_041, ID: 102-48h, Description:

WBPD081_013 (4)

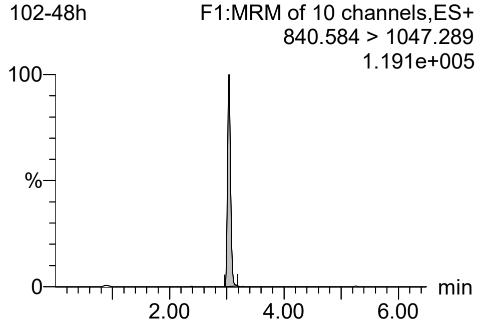

Tolbutamide (1)

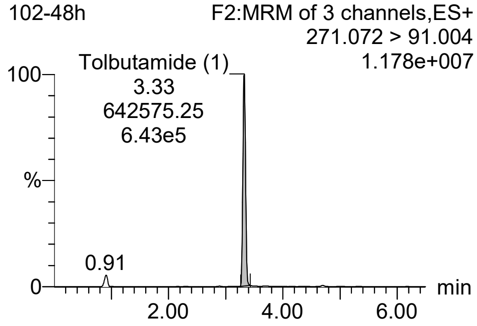

	#	Name	Trace	RT	Area	IS Area	Response	Primar...	Conc.	%Dev
1	1	WBPD081_013 (4)	840.584 > 1047....	3.04	7386.808	642575.250	0.011	bb	108.1	
2	2	Tolbutamide (1)	271.072 > 91.004	3.33	642575.250		642575.250	bb	1.1	10.0

Dataset: D:\Data\27013-24001-NG.PRO\20241212_WBPD081_013_SA_Reinjection_Processed-Tu.qld

Last Altered: Tuesday, July 15, 2025 15:44:36 China Standard Time
Printed: Tuesday, July 15, 2025 15:46:23 China Standard Time

Name: 20241212_1_042, ID: 102-72h, Description:

WBPD081_013 (4)

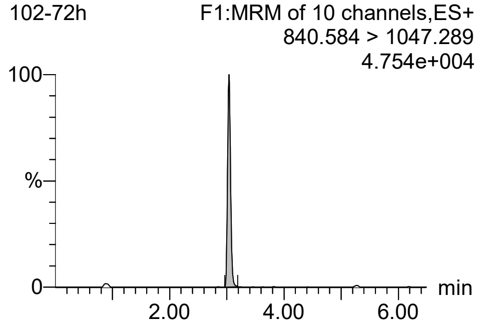

Tolbutamide (1)

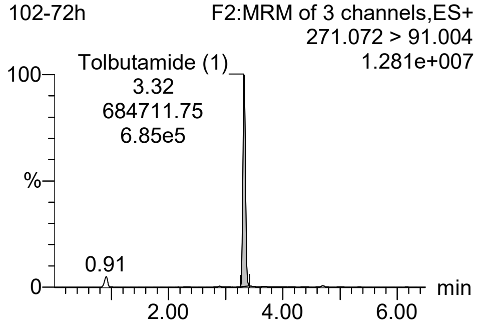

	#	Name	Trace	RT	Area	IS Area	Response	Primar...	Conc.	%Dev
1	1	WBPD081_013 (4)	840.584 > 1047....	3.04	2904.449	684711.750	0.004	bb	39.9	
2	2	Tolbutamide (1)	271.072 > 91.004	3.32	684711.750		684711.750	bb	1.2	17.2

Name: 20241212_1_043, ID: 102-96h, Description:

WBPD081_013 (4)

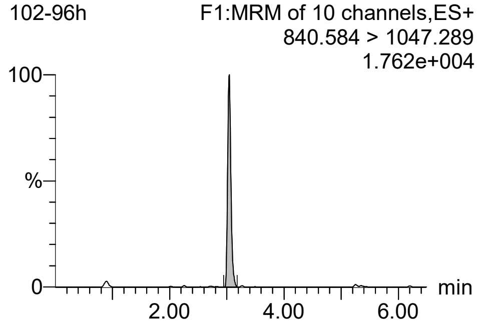

Tolbutamide (1)

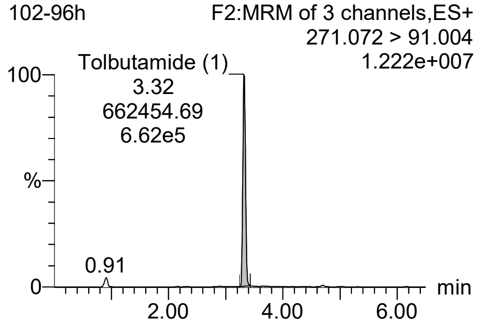

	#	Name	Trace	RT	Area	IS Area	Response	Primar...	Conc.	%Dev
1	1	WBPD081_013 (4)	840.584 > 1047....	3.04	1169.926	662454.688	0.002	bb	16.6	
2	2	Tolbutamide (1)	271.072 > 91.004	3.32	662454.688		662454.688	bb	1.1	13.4

Name: 20241212_1_044, ID: 102-168h, Description:

WBPD081_013 (4)

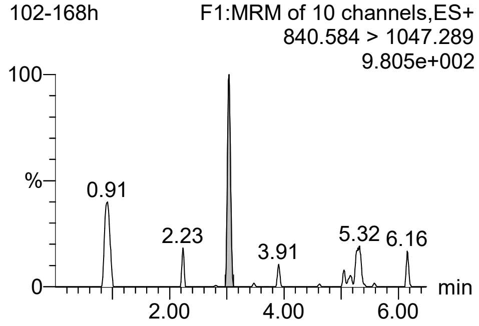

Tolbutamide (1)

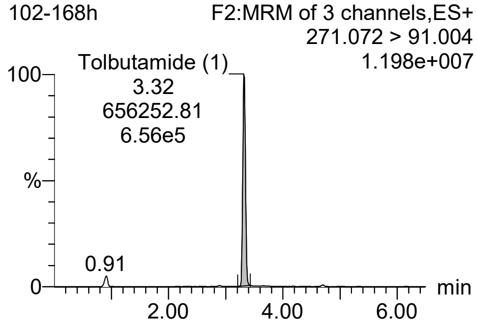

	#	Name	Trace	RT	Area	IS Area	Response	Primar...	Conc.	%Dev
1	1	WBPD081_013 (4)	840.584 > 1047....	3.04	62.472	656252.813	0.000	bb	0.8	
2	2	Tolbutamide (1)	271.072 > 91.004	3.32	656252.813		656252.813	bb	1.1	12.4

Dataset: D:\Data\27013-24001-NG.PRO\20241212_WBPD081_013_SA_Reinjection_Processed-Tu.qld

Last Altered: Tuesday, July 15, 2025 15:44:36 China Standard Time
Printed: Tuesday, July 15, 2025 15:46:23 China Standard Time

Name: 20241212_1_045, ID: Solvent, Description:

WBPD081_013 (4)

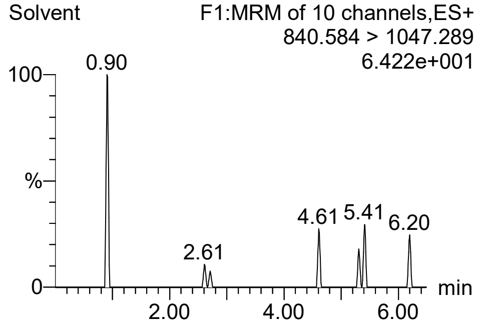

Tolbutamide (1)

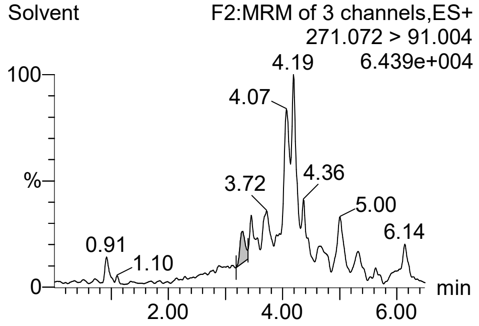

	#	Name	Trace	RT	Area	IS Area	Response	Primar...	Conc.	%Dev
1	1	WBPD081_013 (4)	840.584 > 1047....			1030.409				
2	2	Tolbutamide (1)	271.072 > 91.004	3.30	1030.409		1030.409	bd	0.0	-99.8

Name: 20241212_1_046, ID: Solvent, Description:

WBPD081_013 (4)

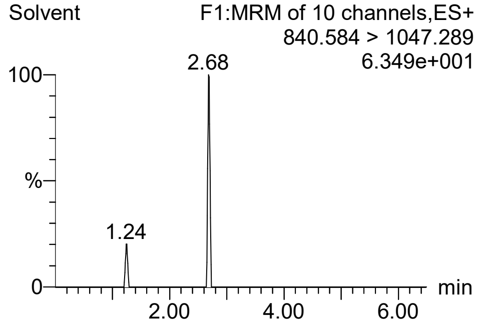

Tolbutamide (1)

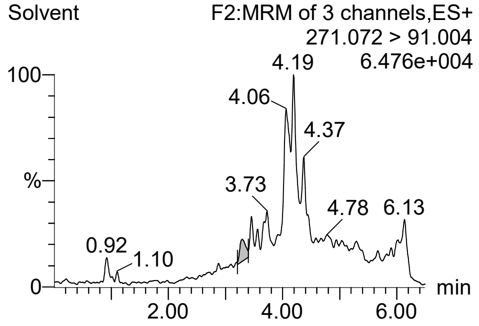

	#	Name	Trace	RT	Area	IS Area	Response	Primar...	Conc.	%Dev
1	1	WBPD081_013 (4)	840.584 > 1047....			807.360				
2	2	Tolbutamide (1)	271.072 > 91.004	3.29	807.360		807.360	dd	0.0	-99.9

Name: 20241212_1_047, ID: 103-Predose, Description:

WBPD081_013 (4)

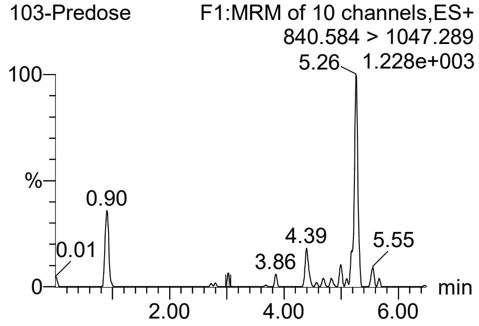

Tolbutamide (1)

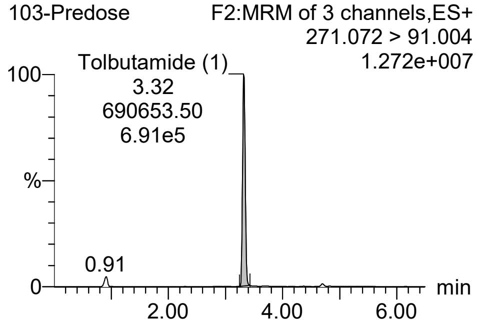

	#	Name	Trace	RT	Area	IS Area	Response	Primar...	Conc.	%Dev
1	1	WBPD081_013 (4)	840.584 > 1047....	3.03	3.586	690653.500	0.000	bbl		
2	2	Tolbutamide (1)	271.072 > 91.004	3.32	690653.500		690653.500	bb	1.2	18.2

Dataset: D:\Data\27013-24001-NG.PRO\20241212_WBPD081_013_SA_Reinjection_Processed-Tu.qld

Last Altered: Tuesday, July 15, 2025 15:44:36 China Standard Time
Printed: Tuesday, July 15, 2025 15:46:23 China Standard Time

Name: 20241212_1_048, ID: 103-2h, Description:

WBPD081_013 (4)

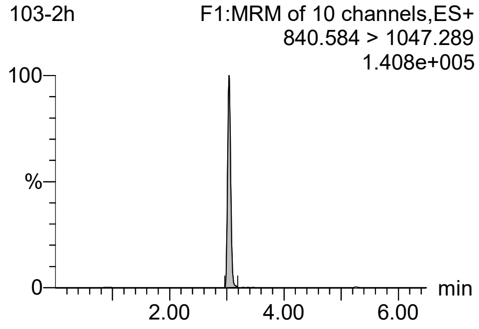

Tolbutamide (1)

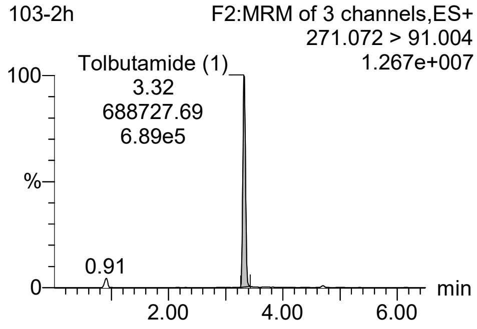

	#	Name	Trace	RT	Area	IS Area	Response	Primar...	Conc.	%Dev
1	1	WBPD081_013 (4)	840.584 > 1047....	3.04	8905.885	688727.688	0.013	bb	121.6	
2	2	Tolbutamide (1)	271.072 > 91.004	3.32	688727.688		688727.688	bb	1.2	17.9

Name: 20241212_1_049, ID: 103-4h, Description:

WBPD081_013 (4)

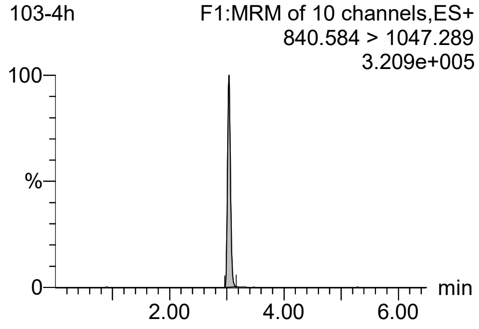

Tolbutamide (1)

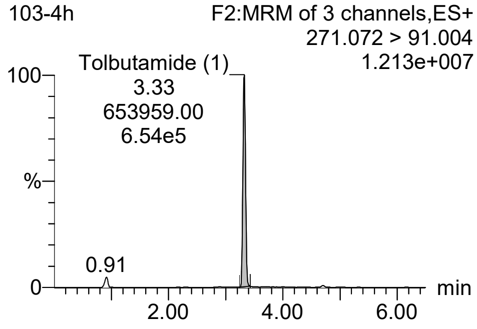

	#	Name	Trace	RT	Area	IS Area	Response	Primar...	Conc.	%Dev
1	1	WBPD081_013 (4)	840.584 > 1047....	3.04	19327.619	653959.000	0.030	bb	278.1	
2	2	Tolbutamide (1)	271.072 > 91.004	3.33	653959.000		653959.000	bb	1.1	12.0

Name: 20241212_1_050, ID: 103-8h, Description:

WBPD081_013 (4)

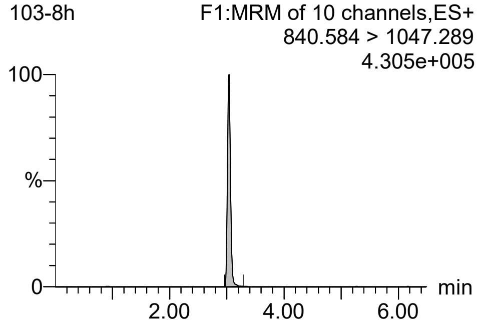

Tolbutamide (1)

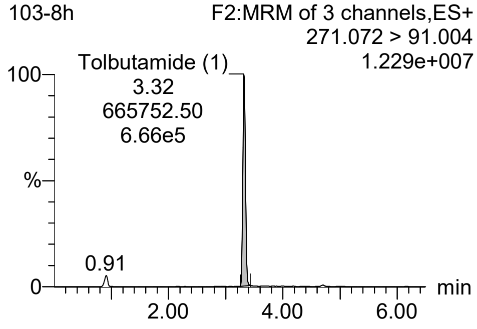

	#	Name	Trace	RT	Area	IS Area	Response	Primar...	Conc.	%Dev
1	1	WBPD081_013 (4)	840.584 > 1047....	3.04	26810.475	665752.500	0.040	bb	379.0	
2	2	Tolbutamide (1)	271.072 > 91.004	3.32	665752.500		665752.500	bb	1.1	14.0

Dataset: D:\Data\27013-24001-NG.PRO\20241212_WBPD081_013_SA_Reinjection_Processed-Tu.qld

Last Altered: Tuesday, July 15, 2025 15:44:36 China Standard Time
Printed: Tuesday, July 15, 2025 15:46:23 China Standard Time

Name: 20241212_1_051, ID: 103-12h, Description:

WBPD081_013 (4)

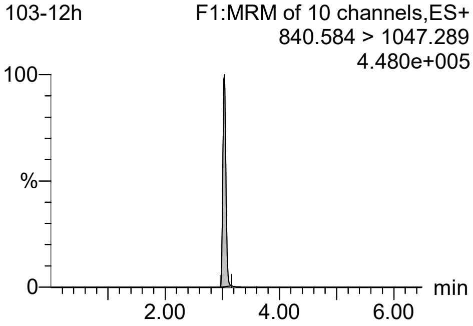

Tolbutamide (1)

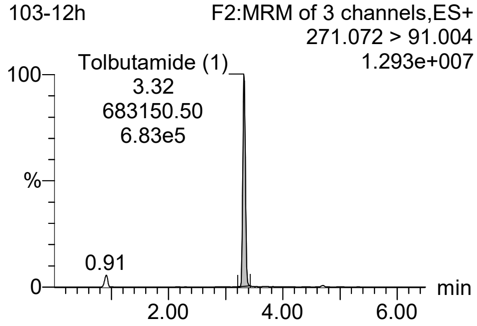

	#	Name	Trace	RT	Area	IS Area	Response	Primar...	Conc.	%Dev
1	1	WBPD081_013 (4)	840.584 > 1047....	3.04	27258.576	683150.500	0.040	bb	375.5	
2	2	Tolbutamide (1)	271.072 > 91.004	3.32	683150.500		683150.500	bb	1.2	17.0

Name: 20241212_1_052, ID: 103-24h, Description:

WBPD081_013 (4)

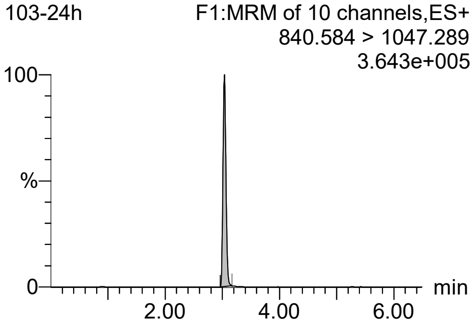

Tolbutamide (1)

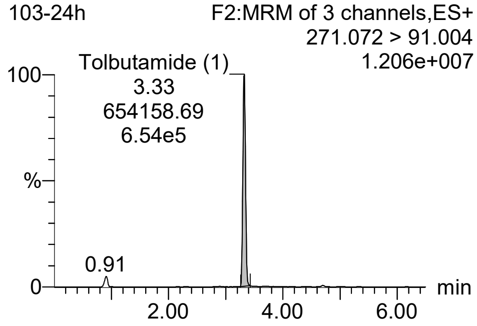

	#	Name	Trace	RT	Area	IS Area	Response	Primar...	Conc.	%Dev
1	1	WBPD081_013 (4)	840.584 > 1047....	3.04	21991.320	654158.688	0.034	bb	316.3	
2	2	Tolbutamide (1)	271.072 > 91.004	3.33	654158.688		654158.688	bb	1.1	12.0

Name: 20241212_1_053, ID: 103-48h, Description:

WBPD081_013 (4)

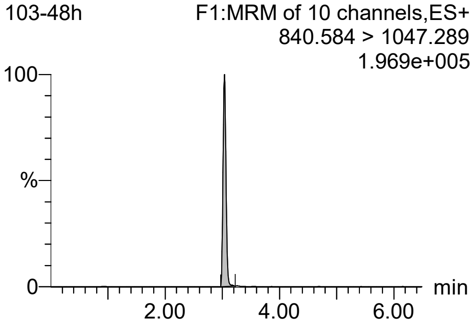

Tolbutamide (1)

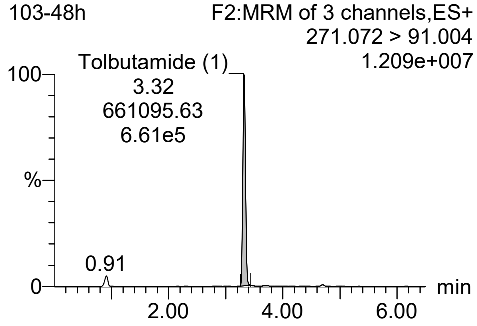

	#	Name	Trace	RT	Area	IS Area	Response	Primar...	Conc.	%Dev
1	1	WBPD081_013 (4)	840.584 > 1047....	3.04	11913.680	661095.625	0.018	bb	169.5	
2	2	Tolbutamide (1)	271.072 > 91.004	3.32	661095.625		661095.625	bb	1.1	13.2

Dataset: D:\Data\27013-24001-NG.PRO\20241212_WBPD081_013_SA_Reinjection_Processed-Tu.qld

Last Altered: Tuesday, July 15, 2025 15:44:36 China Standard Time
Printed: Tuesday, July 15, 2025 15:46:23 China Standard Time

Name: 20241212_1_054, ID: 103-72h, Description:

WBPD081_013 (4)

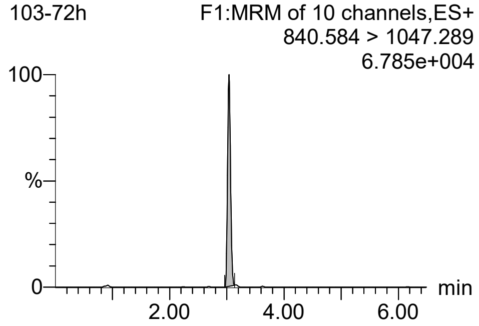

Tolbutamide (1)

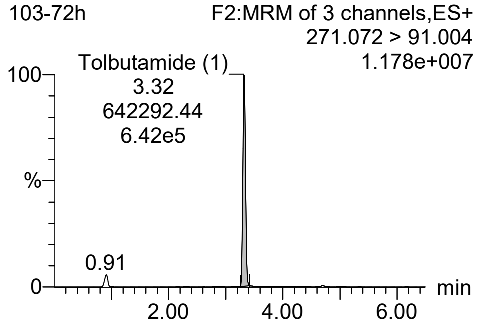

	#	Name	Trace	RT	Area	IS Area	Response	Primar...	Conc.	%Dev
1	1	WBPD081_013 (4)	840.584 > 1047....	3.04	4105.340	642292.438	0.006	bb	60.1	
2	2	Tolbutamide (1)	271.072 > 91.004	3.32	642292.438		642292.438	bb	1.1	10.0

Name: 20241212_1_055, ID: 103-96h, Description:

WBPD081_013 (4)

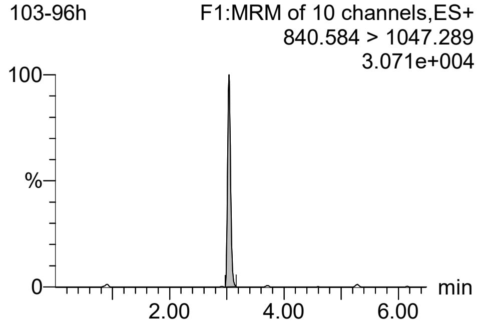

Tolbutamide (1)

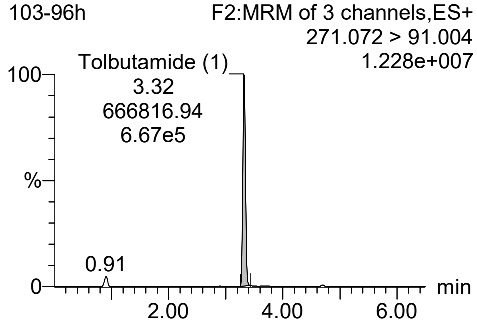

	#	Name	Trace	RT	Area	IS Area	Response	Primar...	Conc.	%Dev
1	1	WBPD081_013 (4)	840.584 > 1047....	3.04	1890.755	666816.938	0.003	bb	26.6	
2	2	Tolbutamide (1)	271.072 > 91.004	3.32	666816.938		666816.938	bb	1.1	14.2

Name: 20241212_1_056, ID: 103-168h, Description:

WBPD081_013 (4)

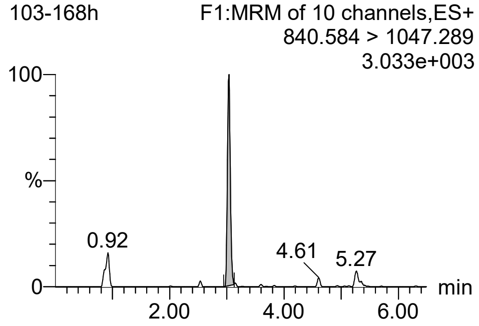

Tolbutamide (1)

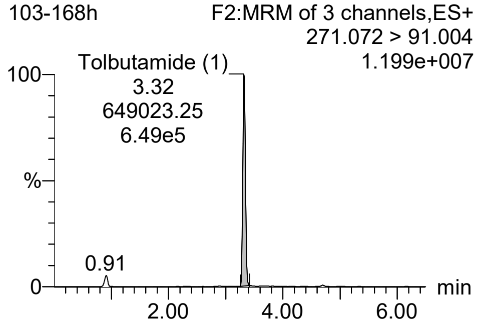

	#	Name	Trace	RT	Area	IS Area	Response	Primar...	Conc.	%Dev
1	1	WBPD081_013 (4)	840.584 > 1047....	3.04	177.615	649023.250	0.000	bb	2.5	
2	2	Tolbutamide (1)	271.072 > 91.004	3.32	649023.250		649023.250	bb	1.1	11.1

Dataset: D:\Data\27013-24001-NG.PRO\20241212_WBPD081_013_SA_Reinjection_Processed-Tu.qld

Last Altered: Tuesday, July 15, 2025 15:44:36 China Standard Time
Printed: Tuesday, July 15, 2025 15:46:23 China Standard Time

Name: 20241212_1_057, ID: Solvent, Description:

WBPD081_013 (4)

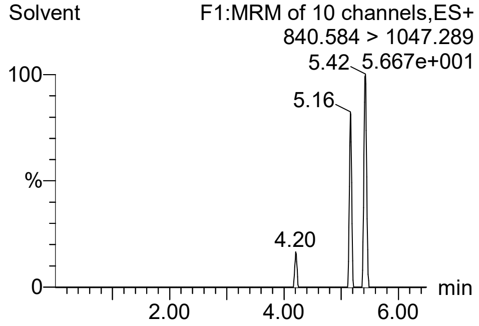

Tolbutamide (1)

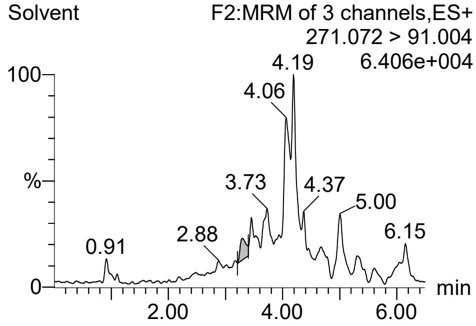

	# Name	Trace	RT	Area	IS Area	Response	Primar...	Conc.	%Dev
1	1 WBPD081_013 (4)	840.584 > 1047....			779.521				
2	2 Tolbutamide (1)	271.072 > 91.004	3.30	779.521		779.521	dd	0.0	-99.9

Name: 20241212_1_058, ID: Solvent, Description:

WBPD081_013 (4)

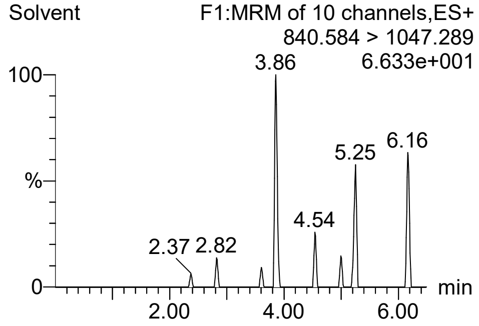

Tolbutamide (1)

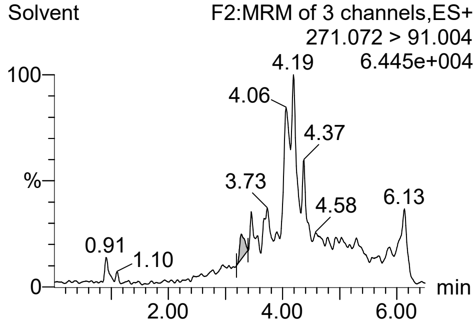

	# Name	Trace	RT	Area	IS Area	Response	Primar...	Conc.	%Dev
1	1 WBPD081_013 (4)	840.584 > 1047....			727.294				
2	2 Tolbutamide (1)	271.072 > 91.004	3.28	727.294		727.294	bb	0.0	-99.9

Name: 20241212_1_059, ID: B, Description:

WBPD081_013 (4)

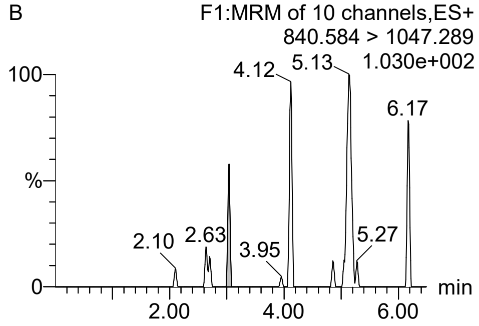

Tolbutamide (1)

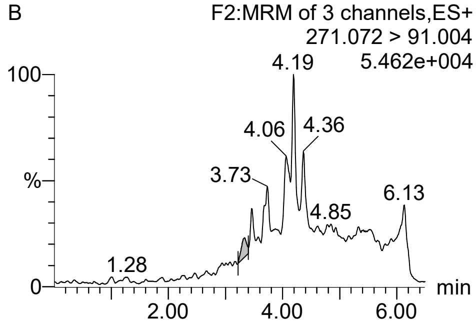

	# Name	Trace	RT	Area	IS Area	Response	Primar...	Conc.	%Dev
1	1 WBPD081_013 (4)	840.584 > 1047....	3.04	2.753	552.923	0.005	bb	46.8	
2	2 Tolbutamide (1)	271.072 > 91.004	3.33	552.923		552.923	bd	0.0	-99.9

Dataset: D:\Data\27013-24001-NG.PRO\20241212_WBPD081_013_SA_Reinjection_Processed-Tu.qld

Last Altered: Tuesday, July 15, 2025 15:44:36 China Standard Time
Printed: Tuesday, July 15, 2025 15:46:23 China Standard Time

Name: 20241212_1_060, ID: O, Description:

WBPD081_013 (4)

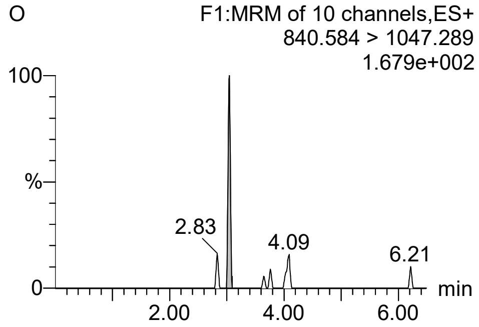

Tolbutamide (1)

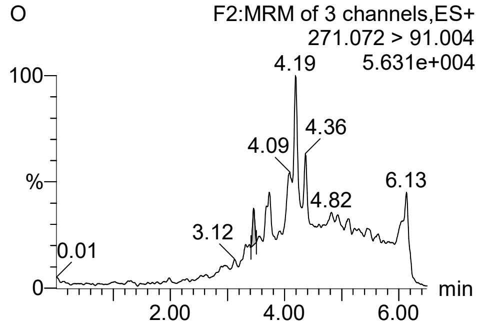

	#	Name	Trace	RT	Area	IS Area	Response	Primar...	Conc.	%Dev
1	1	WBPD081_013 (4)	840.584 > 1047....	3.04	7.929	452.709	0.018	bb	164.8	
2	2	Tolbutamide (1)	271.072 > 91.004	3.46	452.709		452.709	bb	0.0	-99.9

Name: 20241212_1_061, ID: Q1, Description:

WBPD081_013 (4)

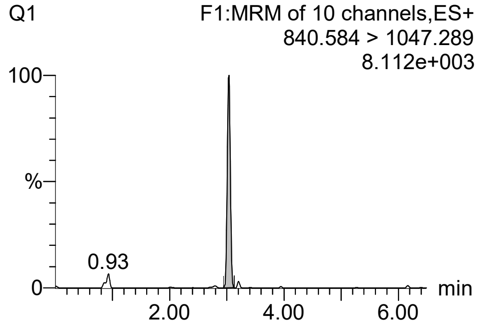

Tolbutamide (1)

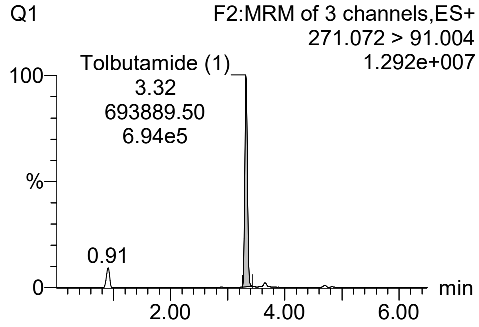

	#	Name	Trace	RT	Area	IS Area	Response	Primar...	Conc.	%Dev
1	1	WBPD081_013 (4)	840.584 > 1047....	3.04	510.812	693889.500	0.001	bb	6.9	14.3
2	2	Tolbutamide (1)	271.072 > 91.004	3.32	693889.500		693889.500	bb	1.2	18.8

Name: 20241212_1_062, ID: Q2, Description:

WBPD081_013 (4)

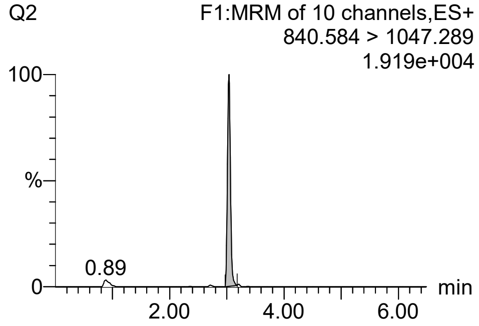

Tolbutamide (1)

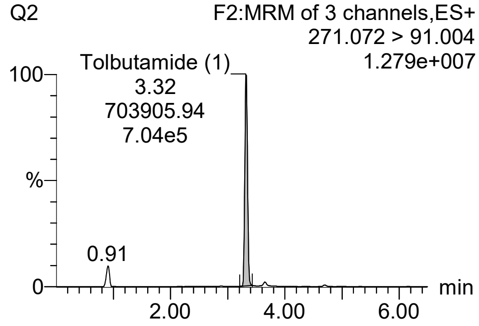

	#	Name	Trace	RT	Area	IS Area	Response	Primar...	Conc.	%Dev
1	1	WBPD081_013 (4)	840.584 > 1047....	3.04	1170.124	703905.938	0.002	bb	15.6	29.8
2	2	Tolbutamide (1)	271.072 > 91.004	3.32	703905.938		703905.938	bb	1.2	20.5

Dataset: D:\Data\27013-24001-NG.PRO\20241212_WBPD081_013_SA_Reinjection_Processed-Tu.qld

Last Altered: Tuesday, July 15, 2025 15:44:36 China Standard Time
Printed: Tuesday, July 15, 2025 15:46:23 China Standard Time

Name: 20241212_1_078, ID: Q3, Description:

WBPD081_013 (4)

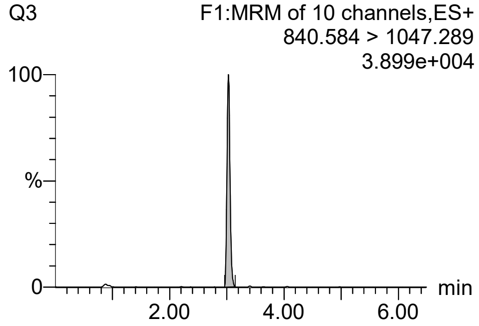

Tolbutamide (1)

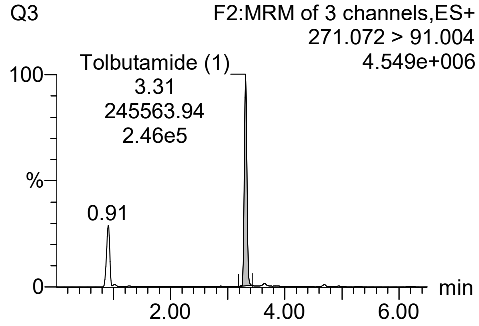

	#	Name	Trace	RT	Area	IS Area	Response	Primar...	Conc.	%Dev
1	1	WBPD081_013 (4)	840.584 > 1047....	3.03	2405.955	245563.938	0.010	bb	92.1	15.2
2	2	Tolbutamide (1)	271.072 > 91.004	3.31	245563.938		245563.938	bb	0.4	-58.0

Name: 20241212_1_079, ID: Q4, Description:

WBPD081_013 (4)

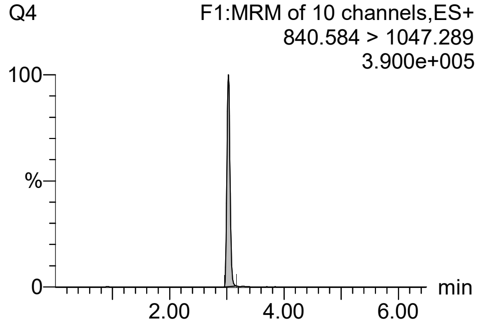

Tolbutamide (1)

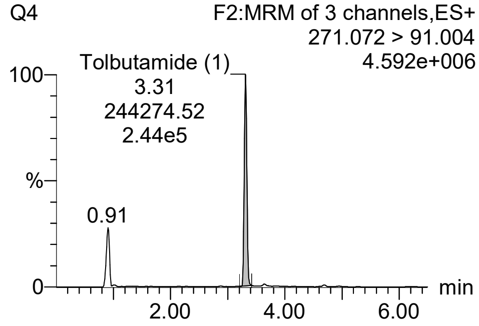

	#	Name	Trace	RT	Area	IS Area	Response	Primar...	Conc.	%Dev
1	1	WBPD081_013 (4)	840.584 > 1047....	3.03	24577.082	244274.516	0.101	bb	946.9	18.4
2	2	Tolbutamide (1)	271.072 > 91.004	3.31	244274.516		244274.516	bb	0.4	-58.2
