# Supplementary material for: AI‐Driven De Novo Design of Ultra Long‐Acting GLP‐1 Receptor Agonists
Source: Adv Sci (Weinh). 2025 Aug 11;12(40):e07044. doi: 10.1002/advs.202507044 (PMC12561408; doi:10.1002/advs.202507044)
Supplement: Supplementary file 2 — Supporting Information [file ADVS-12-e07044-s001.zip › SI_The raw data of PK for D41.pdf]

Dataset: D:\Data\27013-24001-NG.PRO\20241212_WBPD081_041_SA_Reinjection_Processed-Tu.qld

Last Altered: Tuesday, July 15, 2025 15:23:10 China Standard Time
Printed: Tuesday, July 15, 2025 15:40:15 China Standard Time

Method: D:\Data\27013-24001-NG.PRO\MethDB\20241212_WBPD081_041.mdb 13 Dec 2024 15:59:53
Calibration: 15 Jul 2025 15:23:10

Compound name: WBPD081_041
Correlation coefficient: r = 0.995915, r^2 = 0.991847
Calibration curve: 2.11296e-005 * x + -4.20167e-006
Response type: Internal Std (Ref 2), Area * (IS Conc. / IS Area)
Curve type: Linear, Origin: Exclude, Weighting: 1/x^2, Axis trans: None

	Name	ID	Type	Std. Conc	RT	Area	IS Area	Response	Conc.	%Dev	Primar...
1	20241212_2_001	Solvent			3.27	0.395	531.799	0.001	35.4		bb
2	20241212_2_002	Solvent					429.121				
3	20241212_2_003	B	Blank		3.07	11.240	1465.934	0.008	363.1		bb
4	20241212_2_004	O	Blank		3.08	35.353	678199.188	0.000	2.7		bb
5	20241212_2_005	STD1	Standard	2.000	3.00	27.684	712515.250	0.000	2.0	1.9	bb
6	20241212_2_006	STD2	Standard	5.000	3.01	105.409	681415.250	0.000	7.5	50.4	bbX
7	20241212_2_007	STD3	Standard	10.000	3.02	128.815	694686.438	0.000	9.0	-10.3	bb
8	20241212_2_008	STD4	Standard	50.000	3.01	723.305	689625.938	0.001	49.8	-0.3	bb
9	20241212_2_009	STD5	Standard	100.000	3.01	1657.563	731226.125	0.002	107.5	7.5	bb
10	20241212_2_010	STD6	Standard	500.000	3.01	7954.201	660907.813	0.012	569.8	14.0	bb
11	20241212_2_011	STD7	Standard	900.000	3.01	12480.547	686722.813	0.018	860.3	-4.4	bb
12	20241212_2_012	STD8	Standard	1000.000	3.01	13741.096	709628.625	0.019	916.6	-8.3	bb
13	20241212_2_013	Solvent			3.03	56.123	821.289	0.068	3234.3		bb
14	20241212_2_014	Solvent			2.99	12.011	674.333	0.018	843.2		bb
15	20241212_2_015	B	Blank		3.02	1.296	170.900	0.008	359.1		bb
16	20241212_2_016	O	Blank		3.08	32.065	757822.438	0.000	2.2		bb
17	20241212_2_017	Q1	QC	6.000	3.01	76.150	715774.438	0.000	5.2	-12.8	bb
18	20241212_2_018	Q2	QC	12.000	3.02	196.105	691734.438	0.000	13.6	13.5	bb
19	20241212_2_019	Q3	QC	80.000	3.01	1416.035	722977.875	0.002	92.9	16.1	bb
20	20241212_2_020	Q4	QC	800.000	3.00	14446.901	714955.875	0.020	956.5	19.6	bb
21	20241212_2_021	Solvent			3.00	63.312	622.146	0.102	4816.4		bb
22	20241212_2_022	Solvent			2.99	23.414	633.698	0.037	1748.8		bb
23	20241212_2_023	201-Predose			3.00	0.353	722507.438	0.000	0.2		bb
24	20241212_2_024	201-2h			3.01	1314.646	736286.063	0.002	84.7		bb
25	20241212_2_025	201-4h			3.01	2271.374	681147.250	0.003	158.0		bb
26	20241212_2_026	201-8h			3.00	3420.565	709101.375	0.005	228.5		bb
27	20241212_2_027	201-12h			3.01	4041.691	739200.313	0.005	259.0		bb
28	20241212_2_028	201-24h			3.01	3801.075	708719.500	0.005	254.0		bb
29	20241212_2_029	201-48h			3.01	2217.406	694993.000	0.003	151.2		bb
30	20241212_2_030	201-72h			3.01	1018.693	661765.000	0.002	73.1		bb
31	20241212_2_031	201-96h			3.02	406.466	683167.563	0.001	28.4		bb
32	20241212_2_032	201-168h			3.02	26.767	657618.500	0.000	2.1		bb
33	20241212_2_033	Solvent					816.099				
34	20241212_2_034	Solvent					638.108				
35	20241212_2_035	202-Predose			3.03	5.525	726615.875	0.000	0.6		bb
36	20241212_2_036	202-2h			3.01	1414.476	716542.625	0.002	93.6		bb
37	20241212_2_037	202-4h			3.01	3208.318	691756.688	0.005	219.7		bb
38	20241212_2_038	202-8h			3.01	4522.092	691804.438	0.007	309.6		bb
39	20241212_2_039	202-12h			3.01	4684.553	666091.625	0.007	333.0		bb
40	20241212_2_040	202-24h			3.01	4177.303	731026.375	0.006	270.6		bb
41	20241212_2_041	202-48h			3.01	2256.442	672157.500	0.003	159.1		bb
42	20241212_2_042	202-72h			3.01	1081.048	692803.938	0.002	74.0		bb
43	20241212_2_043	202-96h			3.00	372.069	699720.188	0.001	25.4		bb
44	20241212_2_044	202-168h			3.02	77.958	679645.250	0.000	5.6		bb

Dataset: D:\Data\27013-24001-NG.PRO\20241212_WBPD081_041_SA_Reinjection_Processed-Tu.qld

Last Altered: Tuesday, July 15, 2025 15:23:10 China Standard Time
Printed: Tuesday, July 15, 2025 15:40:15 China Standard Time

Compound name: WBPD081_041

	Name	ID	Type	Std. Conc	RT	Area	IS Area	Response	Conc.	%Dev	Primar...
45	20241212_2_045	Solvent					601.392				
46	20241212_2_046	Solvent			3.23	0.238	385.850	0.001	29.4		bb
47	20241212_2_047	203-Predose			3.03	9.530	689729.750	0.000	0.9		bb
48	20241212_2_048	203-2h			3.01	1203.902	715101.750	0.002	79.9		bb
49	20241212_2_049	203-4h			3.01	2523.818	693697.000	0.004	172.4		bb
50	20241212_2_050	203-8h			3.01	3483.185	682779.438	0.005	241.6		bb
51	20241212_2_051	203-12h			3.00	4081.217	665596.813	0.006	290.4		bb
52	20241212_2_052	203-24h			3.01	3843.513	656672.688	0.006	277.2		bb
53	20241212_2_053	203-48h			3.01	1880.726	688733.750	0.003	129.4		bb
54	20241212_2_054	203-72h			3.01	919.890	682752.438	0.001	64.0		bb
55	20241212_2_055	203-96h			3.01	515.954	661889.000	0.001	37.1		bb
56	20241212_2_056	203-168h			3.02	63.591	639825.250	0.000	4.9		bb
57	20241212_2_057	Solvent			3.02	2.048	693.137	0.003	140.0		bb
58	20241212_2_058	Solvent					755.455				
59	20241212_2_059	B	Blank				191.490				
60	20241212_2_060	O	Blank				108.018				
61	20241212_2_061	Q1	QC	6.000	3.01	78.004	717136.188	0.000	5.3	-10.9	bb
62	20241212_2_062	Q2	QC	12.000	3.02	214.106	734519.750	0.000	14.0	16.6	bb
63	20241212_2_063	Q3	QC	80.000	3.01	1545.050	701638.125	0.002	104.4	30.5	bb
64	20241212_2_064	Q4	QC	800.000	3.00	18393.049	676741.750	0.027	1286.5	60.8	bb

Dataset: D:\Data\27013-24001-NG.PRO\20241212_WBPD081_041_SA_Reinjection_Processed-Tu.qld

Last Altered: Tuesday, July 15, 2025 15:23:10 China Standard Time
Printed: Tuesday, July 15, 2025 15:40:15 China Standard Time

Compound name: WBPD081_041

	Inj. Vol	Factor1	Vial
1	10.000	0.0	2:H,12
2	10.000	0.0	2:H,12
3	10.000	0.0	2:E,1
4	10.000	0.0	2:E,2
5	10.000	1.0	2:E,3
6	10.000	1.0	2:E,4
7	10.000	1.0	2:E,5
8	10.000	1.0	2:E,6
9	10.000	1.0	2:E,7
10	10.000	1.0	2:E,8
11	10.000	1.0	2:E,9
12	10.000	1.0	2:E,10
13	10.000	0.0	2:H,12
14	10.000	0.0	2:H,12
15	10.000	0.0	2:E,1
16	10.000	0.0	2:E,2
17	10.000	0.0	2:E,11
18	10.000	0.0	2:E,12
19	10.000	0.0	2:F,1
20	10.000	0.0	2:F,2
21	10.000	0.0	2:H,12
22	10.000	0.0	2:H,12
23	10.000	0.0	2:F,7
24	10.000	0.0	2:F,8
25	10.000	0.0	2:F,9
26	10.000	0.0	2:F,10
27	10.000	0.0	2:F,11
28	10.000	0.0	2:F,12
29	10.000	0.0	2:G,1
30	10.000	0.0	2:G,2
31	10.000	0.0	2:G,3
32	10.000	0.0	2:G,4
33	10.000	0.0	2:H,12
34	10.000	0.0	2:H,12
35	10.000	0.0	2:G,5
36	10.000	0.0	2:G,6
37	10.000	0.0	2:G,7
38	10.000	0.0	2:G,8
39	10.000	0.0	2:G,9
40	10.000	0.0	2:G,10
41	10.000	0.0	2:G,11
42	10.000	0.0	2:G,12
43	10.000	0.0	2:H,1
44	10.000	0.0	2:H,2
45	10.000	0.0	2:H,12
46	10.000	0.0	2:H,12
47	10.000	0.0	2:H,3
48	10.000	0.0	2:H,4
49	10.000	0.0	2:H,5
50	10.000	0.0	2:H,6
51	10.000	0.0	2:H,7

Dataset: D:\Data\27013-24001-NG.PRO\20241212_WBPD081_041_SA_Reinjection_Processed-Tu.qld

Last Altered: Tuesday, July 15, 2025 15:23:10 China Standard Time
Printed: Tuesday, July 15, 2025 15:40:15 China Standard Time

Compound name: WBPD081_041

	Inj. Vol	Factor1	Vial
52	10.000	0.0	2:H,8
53	10.000	0.0	2:H,9
54	10.000	0.0	2:H,10
55	10.000	0.0	2:H,11
56	10.000	0.0	4:H,12
57	10.000	0.0	2:H,12
58	10.000	0.0	2:H,12
59	10.000	0.0	2:E,1
60	10.000	0.0	2:E,2
61	10.000	0.0	2:F,3
62	10.000	0.0	2:F,4
63	10.000	0.0	2:F,5
64	10.000	0.0	2:F,6

Dataset: D:\Data\27013-24001-NG.PRO\20241212_WBPD081_041_SA_Reinjection_Processed-Tu.qld

Last Altered: Tuesday, July 15, 2025 15:23:10 China Standard Time
Printed: Tuesday, July 15, 2025 15:40:15 China Standard Time

Compound name: Tolbutamide (1)
Response Factor: 695841
RRF SD: 21619.3, Relative SD: 3.10693
Response type: External Std, Area
Curve type: RF

	Name	ID	Type	Std. Conc	RT	Area	IS Area	Response	Conc.	%Dev	Primar...
1	20241212_2_001	Solvent		1.000	3.29	531.799		531.799	0.0	-99.9	bd
2	20241212_2_002	Solvent		1.000	3.32	429.121		429.121	0.0	-99.9	bd
3	20241212_2_003	B	Blank	1.000	3.29	1465.934		1465.934	0.0	-99.8	bb
4	20241212_2_004	O	Blank	1.000	3.32	678199.188		678199.188	1.0	-2.5	bb
5	20241212_2_005	STD1	Standard	1.000	3.32	712515.250		712515.250	1.0	2.4	bb
6	20241212_2_006	STD2	Standard	1.000	3.32	681415.250		681415.250	1.0	-2.1	bb
7	20241212_2_007	STD3	Standard	1.000	3.32	694686.438		694686.438	1.0	-0.2	bb
8	20241212_2_008	STD4	Standard	1.000	3.32	689625.938		689625.938	1.0	-0.9	bb
9	20241212_2_009	STD5	Standard	1.000	3.33	731226.125		731226.125	1.1	5.1	bb
10	20241212_2_010	STD6	Standard	1.000	3.32	660907.813		660907.813	0.9	-5.0	bb
11	20241212_2_011	STD7	Standard	1.000	3.32	686722.813		686722.813	1.0	-1.3	bb
12	20241212_2_012	STD8	Standard	1.000	3.32	709628.625		709628.625	1.0	2.0	bb
13	20241212_2_013	Solvent		1.000	3.31	821.289		821.289	0.0	-99.9	bd
14	20241212_2_014	Solvent		1.000	3.30	674.333		674.333	0.0	-99.9	bd
15	20241212_2_015	B	Blank	1.000	3.33	170.900		170.900	0.0	-100.0	bb
16	20241212_2_016	O	Blank	1.000	3.32	757822.438		757822.438	1.1	8.9	bb
17	20241212_2_017	Q1	QC	1.000	3.32	715774.438		715774.438	1.0	2.9	bb
18	20241212_2_018	Q2	QC	1.000	3.32	691734.438		691734.438	1.0	-0.6	bb
19	20241212_2_019	Q3	QC	1.000	3.32	722977.875		722977.875	1.0	3.9	bb
20	20241212_2_020	Q4	QC	1.000	3.32	714955.875		714955.875	1.0	2.7	bb
21	20241212_2_021	Solvent		1.000	3.29	622.146		622.146	0.0	-99.9	bd
22	20241212_2_022	Solvent		1.000	3.31	633.698		633.698	0.0	-99.9	bd
23	20241212_2_023	201-Predose		1.000	3.32	722507.438		722507.438	1.0	3.8	bb
24	20241212_2_024	201-2h		1.000	3.32	736286.063		736286.063	1.1	5.8	bb
25	20241212_2_025	201-4h		1.000	3.32	681147.250		681147.250	1.0	-2.1	bb
26	20241212_2_026	201-8h		1.000	3.32	709101.375		709101.375	1.0	1.9	bb
27	20241212_2_027	201-12h		1.000	3.32	739200.313		739200.313	1.1	6.2	bb
28	20241212_2_028	201-24h		1.000	3.32	708719.500		708719.500	1.0	1.9	bb
29	20241212_2_029	201-48h		1.000	3.32	694993.000		694993.000	1.0	-0.1	bb
30	20241212_2_030	201-72h		1.000	3.32	661765.000		661765.000	1.0	-4.9	bb
31	20241212_2_031	201-96h		1.000	3.32	683167.563		683167.563	1.0	-1.8	bb
32	20241212_2_032	201-168h		1.000	3.32	657618.500		657618.500	0.9	-5.5	bb
33	20241212_2_033	Solvent		1.000	3.31	816.099		816.099	0.0	-99.9	bd
34	20241212_2_034	Solvent		1.000	3.31	638.108		638.108	0.0	-99.9	bd
35	20241212_2_035	202-Predose		1.000	3.32	726615.875		726615.875	1.0	4.4	bb
36	20241212_2_036	202-2h		1.000	3.32	716542.625		716542.625	1.0	3.0	bb
37	20241212_2_037	202-4h		1.000	3.32	691756.688		691756.688	1.0	-0.6	bb
38	20241212_2_038	202-8h		1.000	3.32	691804.438		691804.438	1.0	-0.6	bb
39	20241212_2_039	202-12h		1.000	3.32	666091.625		666091.625	1.0	-4.3	bb
40	20241212_2_040	202-24h		1.000	3.32	731026.375		731026.375	1.1	5.1	bb
41	20241212_2_041	202-48h		1.000	3.32	672157.500		672157.500	1.0	-3.4	bb
42	20241212_2_042	202-72h		1.000	3.32	692803.938		692803.938	1.0	-0.4	bb
43	20241212_2_043	202-96h		1.000	3.32	699720.188		699720.188	1.0	0.6	bb
44	20241212_2_044	202-168h		1.000	3.32	679645.250		679645.250	1.0	-2.3	bb
45	20241212_2_045	Solvent		1.000	3.31	601.392		601.392	0.0	-99.9	bd
46	20241212_2_046	Solvent		1.000	3.30	385.850		385.850	0.0	-99.9	bd
47	20241212_2_047	203-Predose		1.000	3.32	689729.750		689729.750	1.0	-0.9	bb
48	20241212_2_048	203-2h		1.000	3.33	715101.750		715101.750	1.0	2.8	bb

Dataset: D:\Data\27013-24001-NG.PRO\20241212_WBPD081_041_SA_Reinjection_Processed-Tu.qld

Last Altered: Tuesday, July 15, 2025 15:23:10 China Standard Time
Printed: Tuesday, July 15, 2025 15:40:15 China Standard Time

Compound name: Tolbutamide (1)

	Name	ID	Type	Std. Conc	RT	Area	IS Area	Response	Conc.	%Dev	Primar...
49	20241212_2_049	203-4h		1.000	3.32	693697.000		693697.000	1.0	-0.3	bb
50	20241212_2_050	203-8h		1.000	3.32	682779.438		682779.438	1.0	-1.9	bb
51	20241212_2_051	203-12h		1.000	3.32	665596.813		665596.813	1.0	-4.3	bb
52	20241212_2_052	203-24h		1.000	3.32	656672.688		656672.688	0.9	-5.6	bb
53	20241212_2_053	203-48h		1.000	3.32	688733.750		688733.750	1.0	-1.0	bb
54	20241212_2_054	203-72h		1.000	3.32	682752.438		682752.438	1.0	-1.9	bb
55	20241212_2_055	203-96h		1.000	3.32	661889.000		661889.000	1.0	-4.9	bb
56	20241212_2_056	203-168h		1.000	3.32	639825.250		639825.250	0.9	-8.1	bb
57	20241212_2_057	Solvent		1.000	3.29	693.137		693.137	0.0	-99.9	bd
58	20241212_2_058	Solvent		1.000	3.29	755.455		755.455	0.0	-99.9	bd
59	20241212_2_059	B	Blank	1.000	3.38	191.490		191.490	0.0	-100.0	bd
60	20241212_2_060	O	Blank	1.000	3.34	108.018		108.018	0.0	-100.0	bb
61	20241212_2_061	Q1	QC	1.000	3.32	717136.188		717136.188	1.0	3.1	bb
62	20241212_2_062	Q2	QC	1.000	3.32	734519.750		734519.750	1.1	5.6	bb
63	20241212_2_063	Q3	QC	1.000	3.32	701638.125		701638.125	1.0	0.8	bb
64	20241212_2_064	Q4	QC	1.000	3.32	676741.750		676741.750	1.0	-2.7	bb

Dataset: D:\Data\27013-24001-NG.PRO\20241212_WBPD081_041_SA_Reinjection_Processed-Tu.qld

Last Altered: Tuesday, July 15, 2025 15:23:10 China Standard Time
Printed: Tuesday, July 15, 2025 15:40:15 China Standard Time

Compound name: Tolbutamide (1)

	Inj. Vol	Factor1	Vial
1	10.000	1.0	2:H,12
2	10.000	1.0	2:H,12
3	10.000	1.0	2:E,1
4	10.000	1.0	2:E,2
5	10.000	1.0	2:E,3
6	10.000	1.0	2:E,4
7	10.000	1.0	2:E,5
8	10.000	1.0	2:E,6
9	10.000	1.0	2:E,7
10	10.000	1.0	2:E,8
11	10.000	1.0	2:E,9
12	10.000	1.0	2:E,10
13	10.000	1.0	2:H,12
14	10.000	1.0	2:H,12
15	10.000	1.0	2:E,1
16	10.000	1.0	2:E,2
17	10.000	1.0	2:E,11
18	10.000	1.0	2:E,12
19	10.000	1.0	2:F,1
20	10.000	1.0	2:F,2
21	10.000	1.0	2:H,12
22	10.000	1.0	2:H,12
23	10.000	1.0	2:F,7
24	10.000	1.0	2:F,8
25	10.000	1.0	2:F,9
26	10.000	1.0	2:F,10
27	10.000	1.0	2:F,11
28	10.000	1.0	2:F,12
29	10.000	1.0	2:G,1
30	10.000	1.0	2:G,2
31	10.000	1.0	2:G,3
32	10.000	1.0	2:G,4
33	10.000	1.0	2:H,12
34	10.000	1.0	2:H,12
35	10.000	1.0	2:G,5
36	10.000	1.0	2:G,6
37	10.000	1.0	2:G,7
38	10.000	1.0	2:G,8
39	10.000	1.0	2:G,9
40	10.000	1.0	2:G,10
41	10.000	1.0	2:G,11
42	10.000	1.0	2:G,12
43	10.000	1.0	2:H,1
44	10.000	1.0	2:H,2
45	10.000	1.0	2:H,12
46	10.000	1.0	2:H,12
47	10.000	1.0	2:H,3
48	10.000	1.0	2:H,4
49	10.000	1.0	2:H,5
50	10.000	1.0	2:H,6
51	10.000	1.0	2:H,7

Dataset: D:\Data\27013-24001-NG.PRO\20241212_WBPD081_041_SA_Reinjection_Processed-Tu.qld

Last Altered: Tuesday, July 15, 2025 15:23:10 China Standard Time
Printed: Tuesday, July 15, 2025 15:40:15 China Standard Time

Compound name: Tolbutamide (1)

	Inj. Vol	Factor1	Vial
52	10.000	1.0	2:H,8
53	10.000	1.0	2:H,9
54	10.000	1.0	2:H,10
55	10.000	1.0	2:H,11
56	10.000	1.0	4:H,12
57	10.000	1.0	2:H,12
58	10.000	1.0	2:H,12
59	10.000	1.0	2:E,1
60	10.000	1.0	2:E,2
61	10.000	1.0	2:F,3
62	10.000	1.0	2:F,4
63	10.000	1.0	2:F,5
64	10.000	1.0	2:F,6

Dataset: D:\Data\27013-24001-NG.PRO\20241212_WBPD081_041_SA_Reinjection_Processed-Tu.qld

Last Altered: Tuesday, July 15, 2025 15:23:10 China Standard Time
Printed: Tuesday, July 15, 2025 15:40:15 China Standard Time

Method: D:\Data\27013-24001-NG.PRO\MethDB\20241212_WBPD081_041.mdb 13 Dec 2024 15:59:53
Calibration: 15 Jul 2025 15:23:10

Compound name: WBPD081_041
Correlation coefficient: r = 0.995915, r^2 = 0.991847
Calibration curve: 2.11296e-005 * x + -4.20167e-006
Response type: Internal Std (Ref 2), Area * (IS Conc. / IS Area)
Curve type: Linear, Origin: Exclude, Weighting: 1/x^2, Axis trans: None

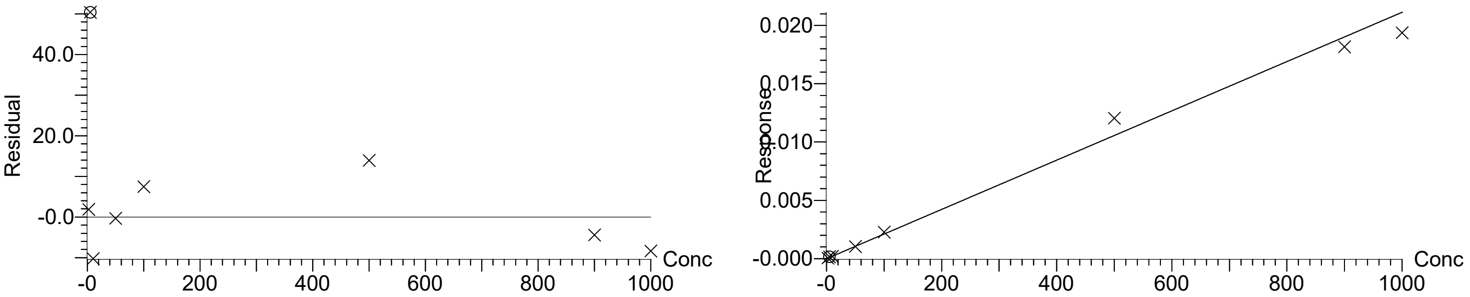

Compound name: Tolbutamide (1)
Response Factor: 695841
RRF SD: 21619.3, % Relative SD: 3.10693
Response type: External Std, Area
Curve type: RF

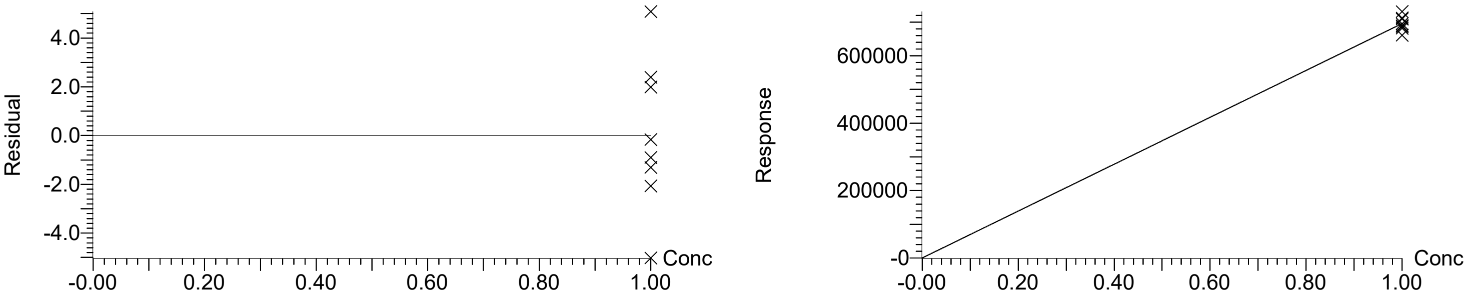

Dataset: D:\Data\27013-24001-NG.PRO\20241212_WBPD081_041_SA_Reinjection_Processed-Tu.qld

Last Altered: Tuesday, July 15, 2025 15:23:10 China Standard Time
Printed: Tuesday, July 15, 2025 15:40:15 China Standard Time

Method: D:\Data\27013-24001-NG.PRO\MethDB\20241212_WBPD081_041.mdb 13 Dec 2024 15:59:53
Calibration: 15 Jul 2025 15:23:10

Name: 20241212_2_001, ID: Solvent, Description:

WBPD081_041

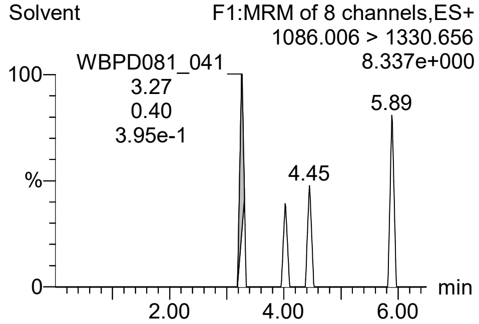

Tolbutamide (1)

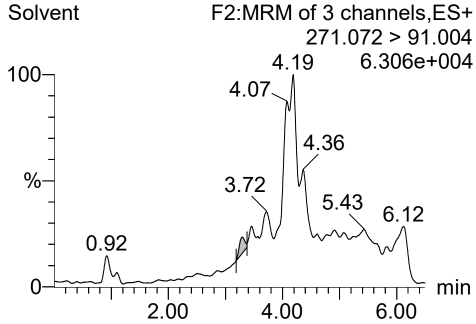

	#	Name	Trace	RT	Area	IS Area	Response	Primar...	Conc.	%Dev
1	1	WBPD081_041	1086.006 > 1330...	3.27	0.395	531.799	0.001	bb	35.4	
2	2	Tolbutamide (1)	271.072 > 91.004	3.29	531.799		531.799	bd	0.0	-99.9

Name: 20241212_2_002, ID: Solvent, Description:

WBPD081_041

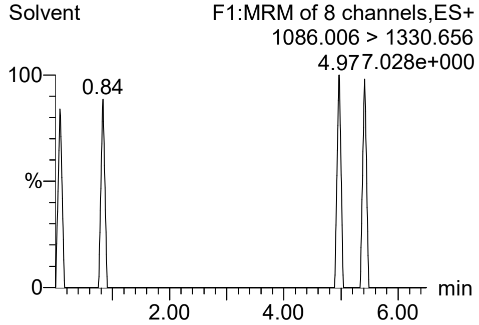

Tolbutamide (1)

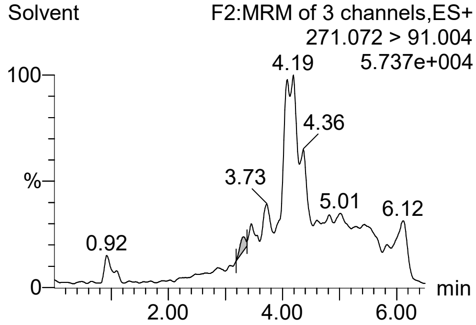

	#	Name	Trace	RT	Area	IS Area	Response	Primar...	Conc.	%Dev
1	1	WBPD081_041	1086.006 > 1330...			429.121				
2	2	Tolbutamide (1)	271.072 > 91.004	3.32	429.121		429.121	bd	0.0	-99.9

Name: 20241212_2_003, ID: B, Description:

WBPD081_041

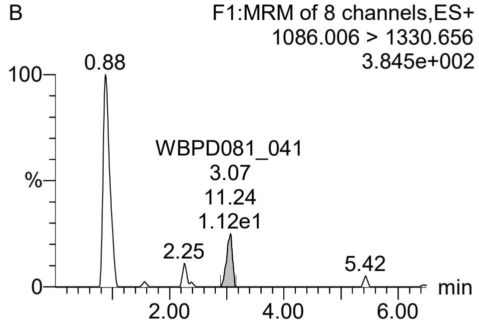

Tolbutamide (1)

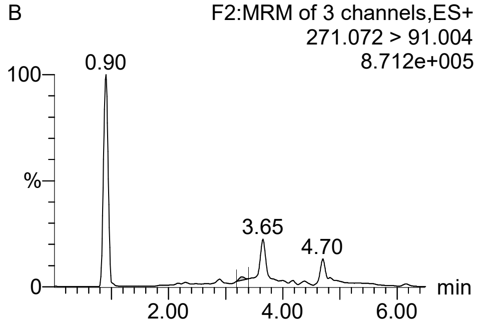

Dataset: D:\Data\27013-24001-NG.PRO\20241212_WBPD081_041_SA_Reinjection_Processed-Tu.qld

Last Altered: Tuesday, July 15, 2025 15:23:10 China Standard Time
Printed: Tuesday, July 15, 2025 15:40:15 China Standard Time

Name: 20241212_2_003, ID: B, Description:

	#	Name	Trace	RT	Area	IS Area	Response	Primar...	Conc.	%Dev
1	1	WBPD081_041	1086.006 > 1330...	3.07	11.240	1465.934	0.008	bb	363.1	
2	2	Tolbutamide (1)	271.072 > 91.004	3.29	1465.934		1465.934	bb	0.0	-99.8

Name: 20241212_2_004, ID: O, Description:

WBPD081_041

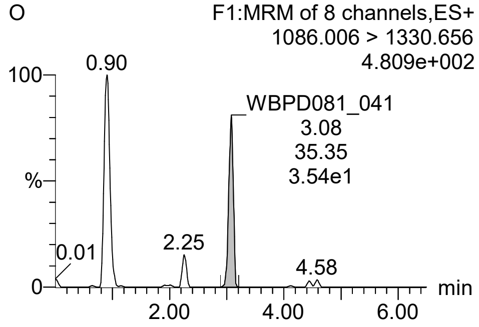

Tolbutamide (1)

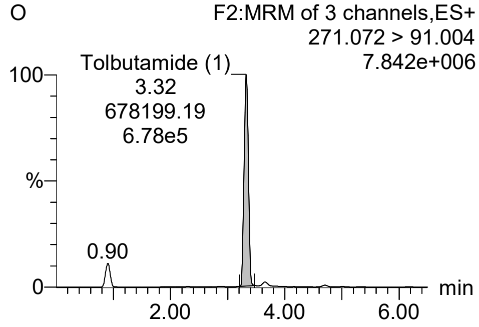

	#	Name	Trace	RT	Area	IS Area	Response	Primar...	Conc.	%Dev
1	1	WBPD081_041	1086.006 > 1330...	3.08	35.353	678199.188	0.000	bb	2.7	
2	2	Tolbutamide (1)	271.072 > 91.004	3.32	678199.188		678199.188	bb	1.0	-2.5

Name: 20241212_2_005, ID: STD1, Description: WBPD081_041

WBPD081_041

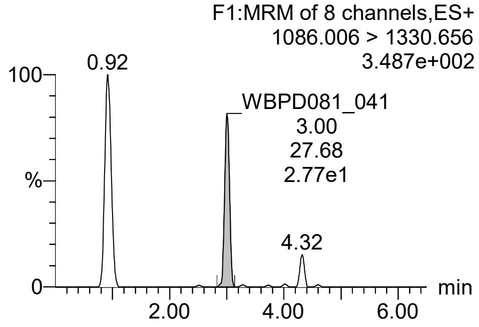

Tolbutamide (1)

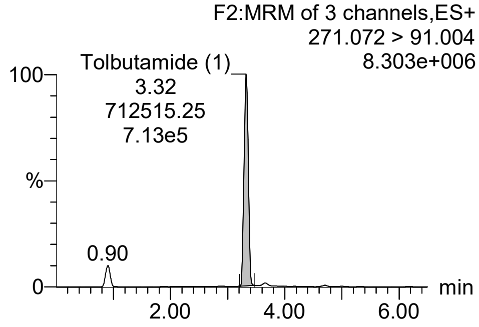

	#	Name	Trace	RT	Area	IS Area	Response	Primar...	Conc.	%Dev
1	1	WBPD081_041	1086.006 > 1330...	3.00	27.684	712515.250	0.000	bb	2.0	1.9
2	2	Tolbutamide (1)	271.072 > 91.004	3.32	712515.250		712515.250	bb	1.0	2.4

Name: 20241212_2_006, ID: STD2, Description: WBPD081_041

Dataset: D:\Data\27013-24001-NG.PRO\20241212_WBPD081_041_SA_Reinjection_Processed-Tu.qld

Last Altered: Tuesday, July 15, 2025 15:23:10 China Standard Time
Printed: Tuesday, July 15, 2025 15:40:15 China Standard Time

Name: 20241212_2_006, ID: STD2, Description: WBPD081_041

WBPD081_041

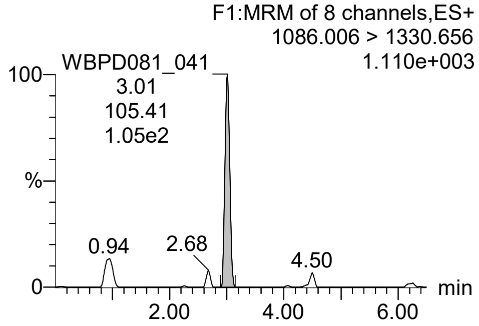

Tolbutamide (1)

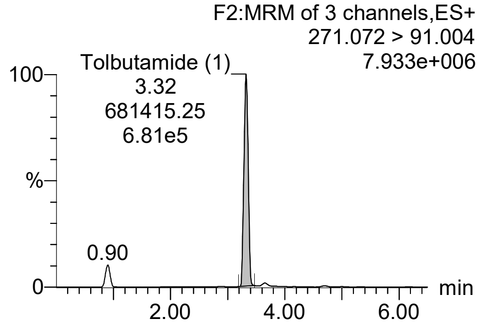

	#	Name	Trace	RT	Area	IS Area	Response	Primar...	Conc.	%Dev
1	1	WBPD081_041	1086.006 > 1330...	3.01	105.409	681415.250	0.000	bbX	7.5	50.4
2	2	Tolbutamide (1)	271.072 > 91.004	3.32	681415.250		681415.250	bb	1.0	-2.1

Name: 20241212_2_007, ID: STD3, Description: WBPD081_041

WBPD081_041

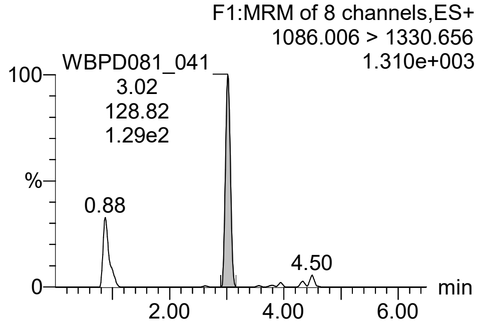

Tolbutamide (1)

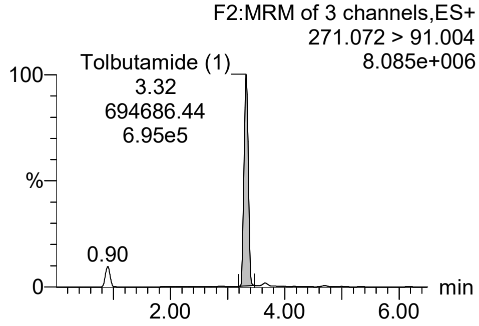

	#	Name	Trace	RT	Area	IS Area	Response	Primar...	Conc.	%Dev
1	1	WBPD081_041	1086.006 > 1330...	3.02	128.815	694686.438	0.000	bb	9.0	-10.3
2	2	Tolbutamide (1)	271.072 > 91.004	3.32	694686.438		694686.438	bb	1.0	-0.2

Name: 20241212_2_008, ID: STD4, Description: WBPD081_041

WBPD081_041

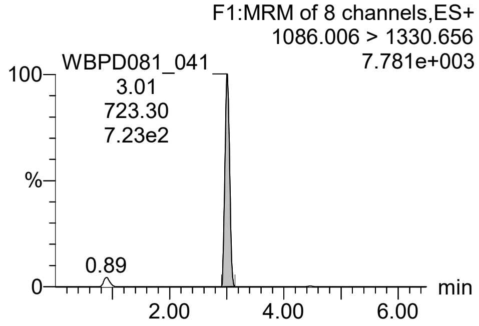

Tolbutamide (1)

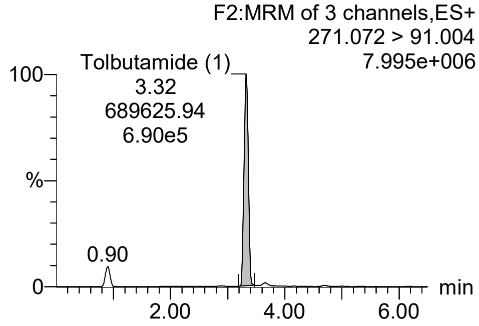

	#	Name	Trace	RT	Area	IS Area	Response	Primar...	Conc.	%Dev
1	1	WBPD081_041	1086.006 > 1330...	3.01	723.305	689625.938	0.001	bb	49.8	-0.3
2	2	Tolbutamide (1)	271.072 > 91.004	3.32	689625.938		689625.938	bb	1.0	-0.9

Dataset: D:\Data\27013-24001-NG.PRO\20241212_WBPD081_041_SA_Reinjection_Processed-Tu.qld

Last Altered: Tuesday, July 15, 2025 15:23:10 China Standard Time
Printed: Tuesday, July 15, 2025 15:40:15 China Standard Time

Name: 20241212_2_009, ID: STD5, Description: WBPD081_041

WBPD081_041

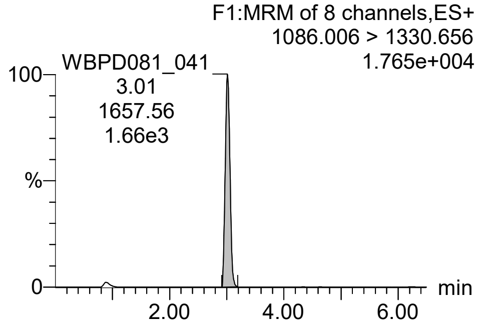

Tolbutamide (1)

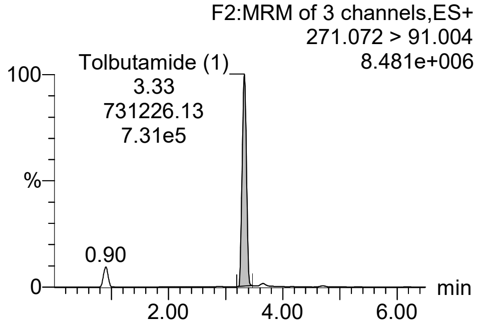

	#	Name	Trace	RT	Area	IS Area	Response	Primar...	Conc.	%Dev
1	1	WBPD081_041	1086.006 > 1330...	3.01	1657.563	731226.125	0.002	bb	107.5	7.5
2	2	Tolbutamide (1)	271.072 > 91.004	3.33	731226.125		731226.125	bb	1.1	5.1

Name: 20241212_2_010, ID: STD6, Description: WBPD081_041

WBPD081_041

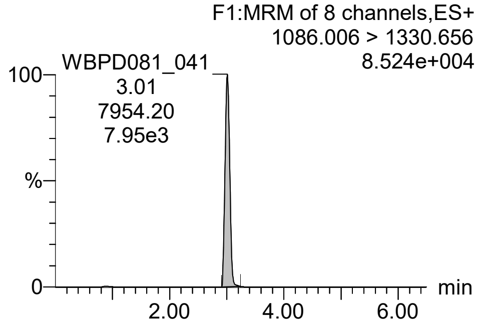

Tolbutamide (1)

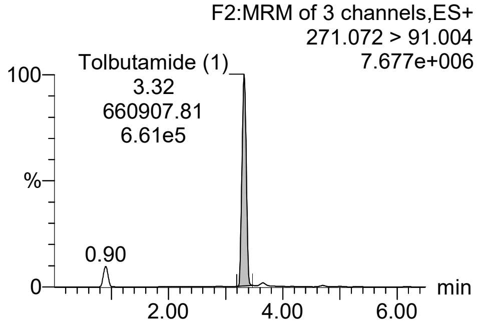

	#	Name	Trace	RT	Area	IS Area	Response	Primar...	Conc.	%Dev
1	1	WBPD081_041	1086.006 > 1330...	3.01	7954.201	660907.813	0.012	bb	569.8	14.0
2	2	Tolbutamide (1)	271.072 > 91.004	3.32	660907.813		660907.813	bb	0.9	-5.0

Name: 20241212_2_011, ID: STD7, Description: WBPD081_041

WBPD081_041

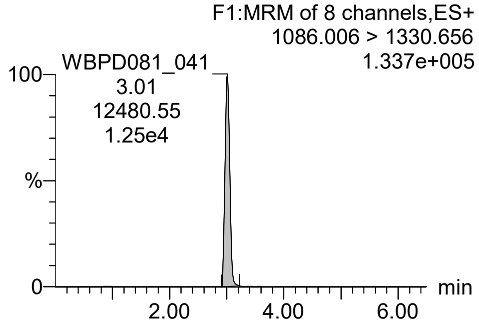

Tolbutamide (1)

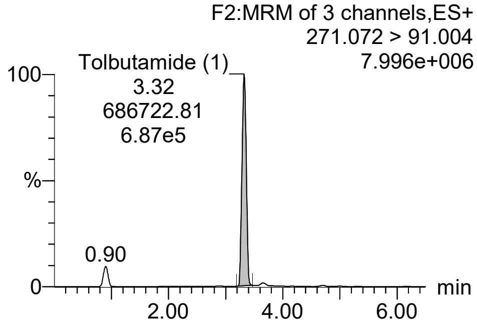

	#	Name	Trace	RT	Area	IS Area	Response	Primar...	Conc.	%Dev
1	1	WBPD081_041	1086.006 > 1330...	3.01	12480.547	686722.813	0.018	bb	860.3	-4.4
2	2	Tolbutamide (1)	271.072 > 91.004	3.32	686722.813		686722.813	bb	1.0	-1.3

Dataset: D:\Data\27013-24001-NG.PRO\20241212_WBPD081_041_SA_Reinjection_Processed-Tu.qld

Last Altered: Tuesday, July 15, 2025 15:23:10 China Standard Time
Printed: Tuesday, July 15, 2025 15:40:15 China Standard Time

Name: 20241212_2_012, ID: STD8, Description: WBPD081_041

WBPD081_041

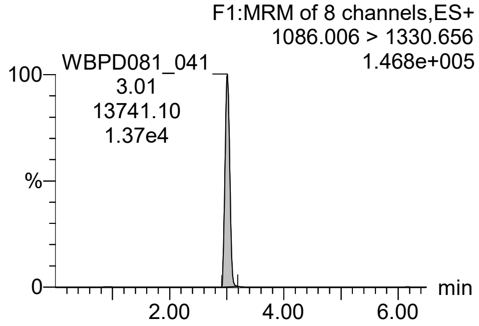

Tolbutamide (1)

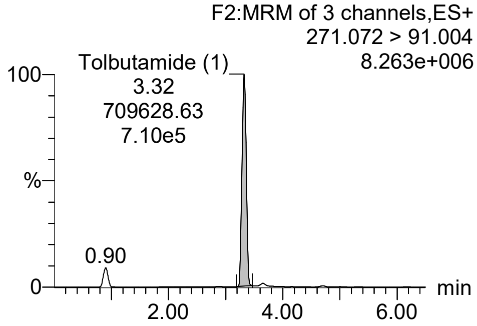

	#	Name	Trace	RT	Area	IS Area	Response	Primar...	Conc.	%Dev
1	1	WBPD081_041	1086.006 > 1330...	3.01	13741.096	709628.625	0.019	bb	916.6	-8.3
2	2	Tolbutamide (1)	271.072 > 91.004	3.32	709628.625		709628.625	bb	1.0	2.0

Name: 20241212_2_013, ID: Solvent, Description:

WBPD081_041

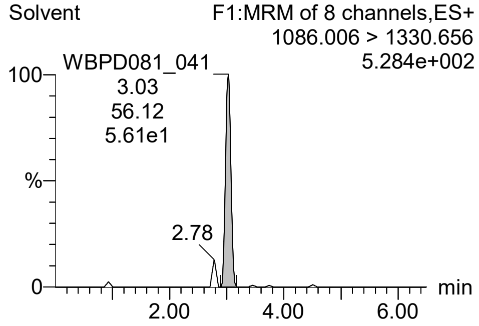

Tolbutamide (1)

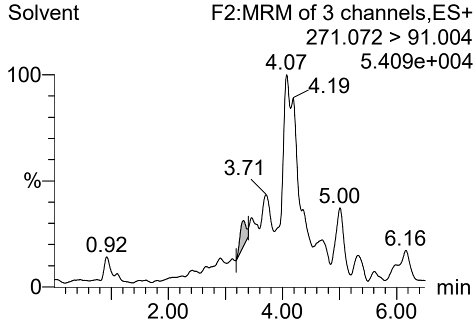

	#	Name	Trace	RT	Area	IS Area	Response	Primar...	Conc.	%Dev
1	1	WBPD081_041	1086.006 > 1330...	3.03	56.123	821.289	0.068	bb	3234.3	
2	2	Tolbutamide (1)	271.072 > 91.004	3.31	821.289		821.289	bd	0.0	-99.9

Name: 20241212_2_014, ID: Solvent, Description:

WBPD081_041

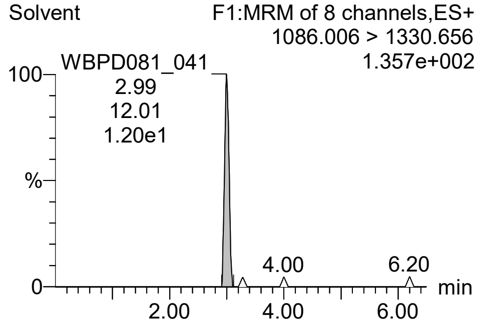

Tolbutamide (1)

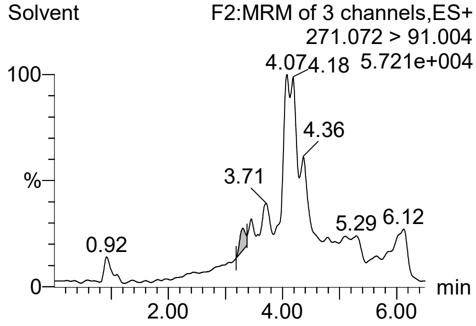

	#	Name	Trace	RT	Area	IS Area	Response	Primar...	Conc.	%Dev
1	1	WBPD081_041	1086.006 > 1330...	2.99	12.011	674.333	0.018	bb	843.2	
2	2	Tolbutamide (1)	271.072 > 91.004	3.30	674.333		674.333	bd	0.0	-99.9

Dataset: D:\Data\27013-24001-NG.PRO\20241212_WBPD081_041_SA_Reinjection_Processed-Tu.qld

Last Altered: Tuesday, July 15, 2025 15:23:10 China Standard Time
Printed: Tuesday, July 15, 2025 15:40:15 China Standard Time

Name: 20241212_2_015, ID: B, Description:

WBPD081_041

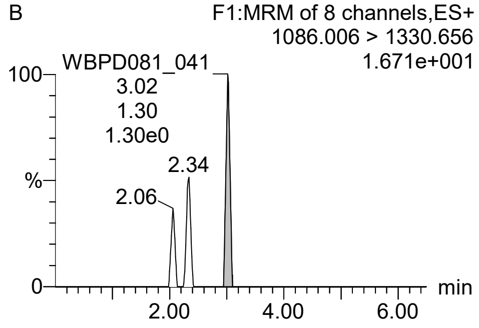

Tolbutamide (1)

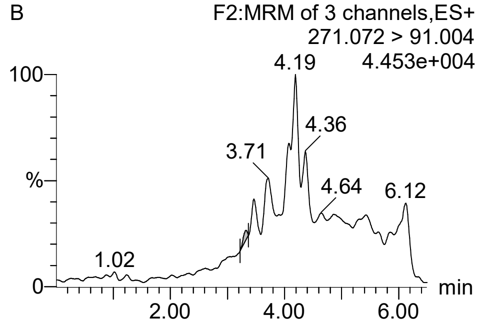

	#	Name	Trace	RT	Area	IS Area	Response	Primar...	Conc.	%Dev
1	1	WBPD081_041	1086.006 > 1330...	3.02	1.296	170.900	0.008	bb	359.1	
2	2	Tolbutamide (1)	271.072 > 91.004	3.33	170.900		170.900	bb	0.0	-100.0

Name: 20241212_2_016, ID: O, Description:

WBPD081_041

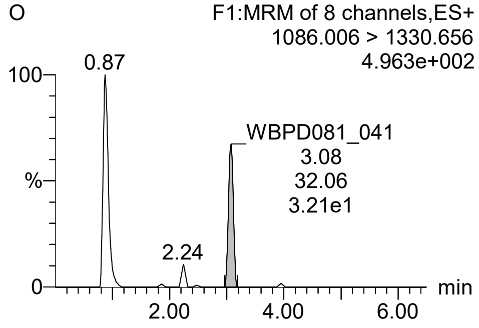

Tolbutamide (1)

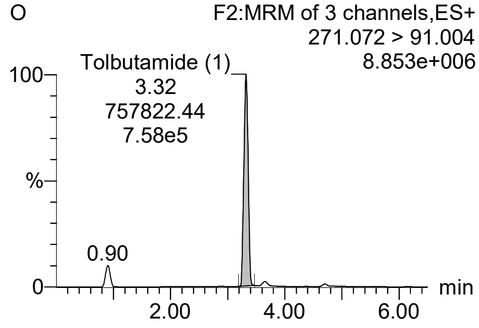

	#	Name	Trace	RT	Area	IS Area	Response	Primar...	Conc.	%Dev
1	1	WBPD081_041	1086.006 > 1330...	3.08	32.065	757822.438	0.000	bb	2.2	
2	2	Tolbutamide (1)	271.072 > 91.004	3.32	757822.438		757822.438	bb	1.1	8.9

Name: 20241212_2_017, ID: Q1, Description:

WBPD081_041

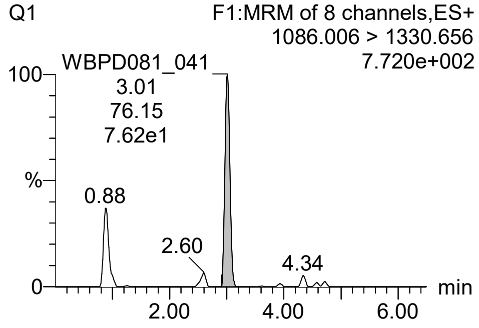

Tolbutamide (1)

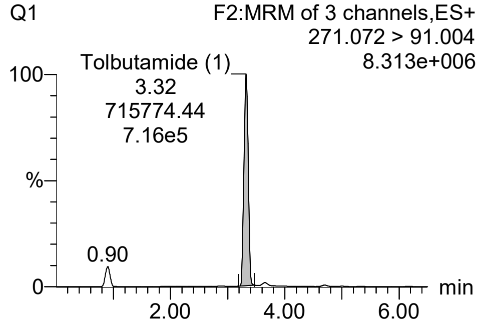

	#	Name	Trace	RT	Area	IS Area	Response	Primar...	Conc.	%Dev
1	1	WBPD081_041	1086.006 > 1330...	3.01	76.150	715774.438	0.000	bb	5.2	-12.8
2	2	Tolbutamide (1)	271.072 > 91.004	3.32	715774.438		715774.438	bb	1.0	2.9

Dataset: D:\Data\27013-24001-NG.PRO\20241212_WBPD081_041_SA_Reinjection_Processed-Tu.qld

Last Altered: Tuesday, July 15, 2025 15:23:10 China Standard Time
Printed: Tuesday, July 15, 2025 15:40:15 China Standard Time

Name: 20241212_2_018, ID: Q2, Description:

WBPD081_041

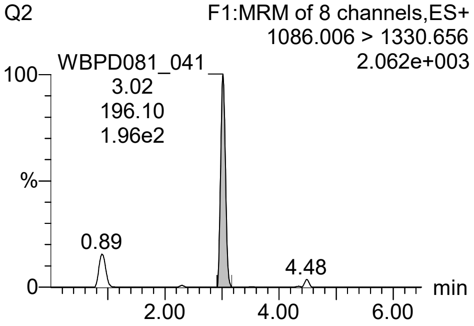

Tolbutamide (1)

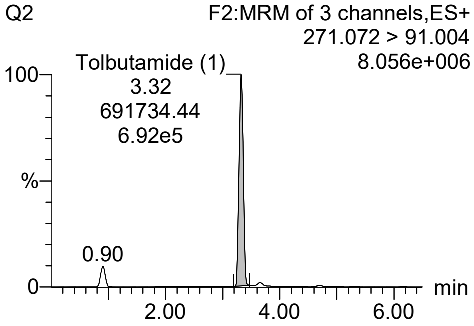

	# Name	Trace	RT	Area	IS Area	Response	Primar...	Conc.	%Dev
1	1 WBPD081_041	1086.006 > 1330...	3.02	196.105	691734.438	0.000	bb	13.6	13.5
2	2 Tolbutamide (1)	271.072 > 91.004	3.32	691734.438		691734.438	bb	1.0	-0.6

Name: 20241212_2_019, ID: Q3, Description:

WBPD081_041

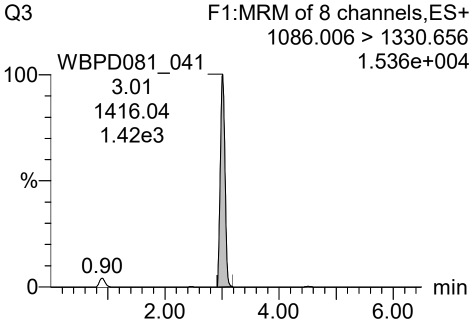

Tolbutamide (1)

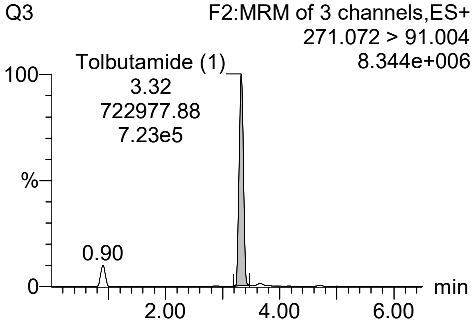

	# Name	Trace	RT	Area	IS Area	Response	Primar...	Conc.	%Dev
1	1 WBPD081_041	1086.006 > 1330...	3.01	1416.035	722977.875	0.002	bb	92.9	16.1
2	2 Tolbutamide (1)	271.072 > 91.004	3.32	722977.875		722977.875	bb	1.0	3.9

Name: 20241212_2_020, ID: Q4, Description:

WBPD081_041

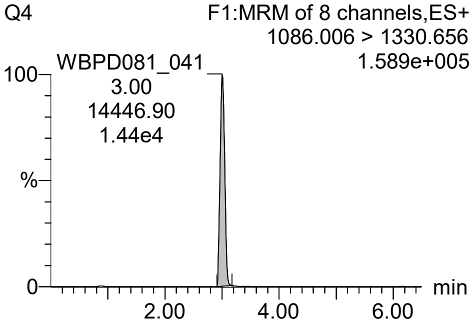

Tolbutamide (1)

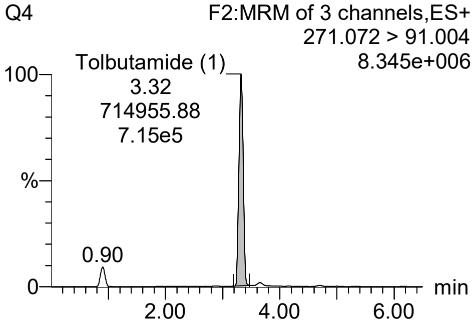

	# Name	Trace	RT	Area	IS Area	Response	Primar...	Conc.	%Dev
1	1 WBPD081_041	1086.006 > 1330...	3.00	14446.901	714955.875	0.020	bb	956.5	19.6
2	2 Tolbutamide (1)	271.072 > 91.004	3.32	714955.875		714955.875	bb	1.0	2.7

Dataset: D:\Data\27013-24001-NG.PRO\20241212_WBPD081_041_SA_Reinjection_Processed-Tu.qld

Last Altered: Tuesday, July 15, 2025 15:23:10 China Standard Time
Printed: Tuesday, July 15, 2025 15:40:15 China Standard Time

Name: 20241212_2_021, ID: Solvent, Description:

WBPD081_041

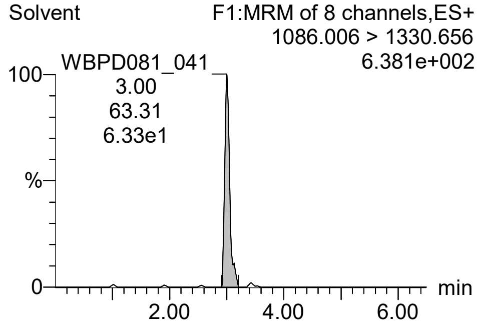

Tolbutamide (1)

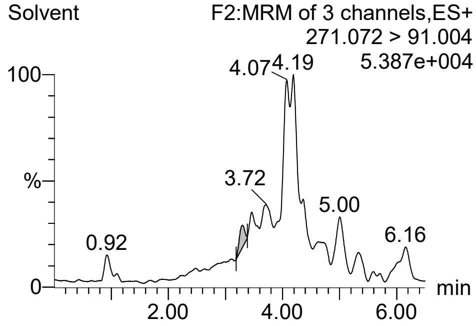

	#	Name	Trace	RT	Area	IS Area	Response	Primar...	Conc.	%Dev
1	1	WBPD081_041	1086.006 > 1330...	3.00	63.312	622.146	0.102	bb	4816.4	
2	2	Tolbutamide (1)	271.072 > 91.004	3.29	622.146		622.146	bd	0.0	-99.9

Name: 20241212_2_022, ID: Solvent, Description:

WBPD081_041

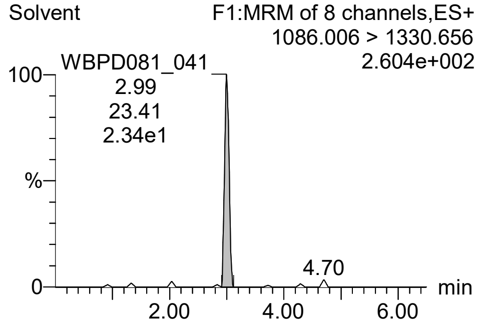

Tolbutamide (1)

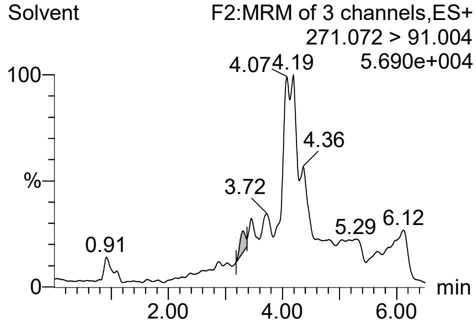

	#	Name	Trace	RT	Area	IS Area	Response	Primar...	Conc.	%Dev
1	1	WBPD081_041	1086.006 > 1330...	2.99	23.414	633.698	0.037	bb	1748.8	
2	2	Tolbutamide (1)	271.072 > 91.004	3.31	633.698		633.698	bd	0.0	-99.9

Name: 20241212_2_023, ID: 201-Predose, Description:

WBPD081_041

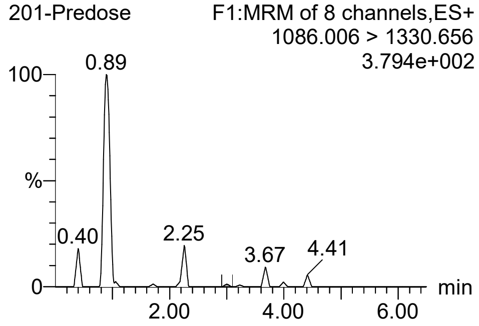

Tolbutamide (1)

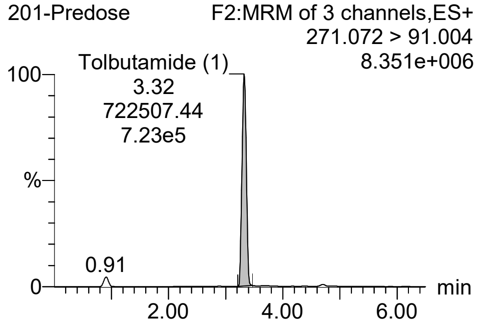

	#	Name	Trace	RT	Area	IS Area	Response	Primar...	Conc.	%Dev
1	1	WBPD081_041	1086.006 > 1330...	3.00	0.353	722507.438	0.000	bb	0.2	
2	2	Tolbutamide (1)	271.072 > 91.004	3.32	722507.438		722507.438	bb	1.0	3.8

Dataset: D:\Data\27013-24001-NG.PRO\20241212_WBPD081_041_SA_Reinjection_Processed-Tu.qld

Last Altered: Tuesday, July 15, 2025 15:23:10 China Standard Time
Printed: Tuesday, July 15, 2025 15:40:15 China Standard Time

Name: 20241212_2_024, ID: 201-2h, Description:

WBPD081_041

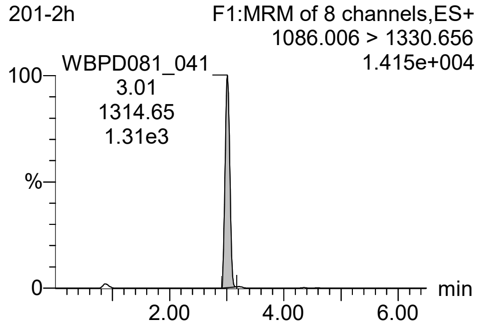

Tolbutamide (1)

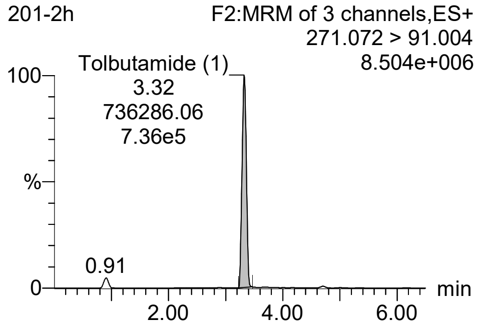

	#	Name	Trace	RT	Area	IS Area	Response	Primar...	Conc.	%Dev
1	1	WBPD081_041	1086.006 > 1330...	3.01	1314.646	736286.063	0.002	bb	84.7	
2	2	Tolbutamide (1)	271.072 > 91.004	3.32	736286.063		736286.063	bb	1.1	5.8

Name: 20241212_2_025, ID: 201-4h, Description:

WBPD081_041

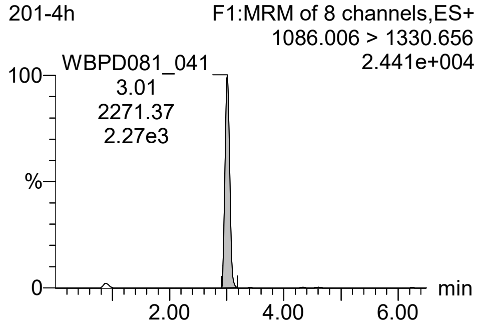

Tolbutamide (1)

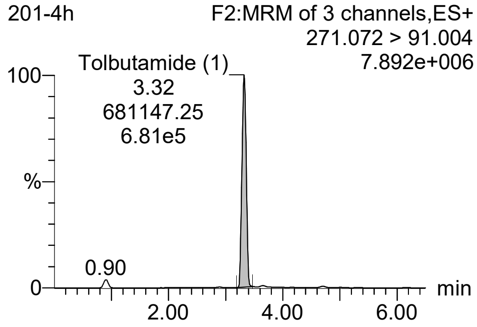

	#	Name	Trace	RT	Area	IS Area	Response	Primar...	Conc.	%Dev
1	1	WBPD081_041	1086.006 > 1330...	3.01	2271.374	681147.250	0.003	bb	158.0	
2	2	Tolbutamide (1)	271.072 > 91.004	3.32	681147.250		681147.250	bb	1.0	-2.1

Name: 20241212_2_026, ID: 201-8h, Description:

WBPD081_041

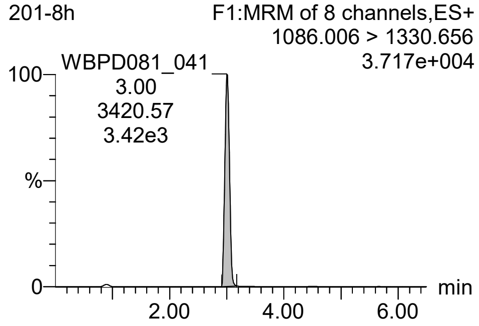

Tolbutamide (1)

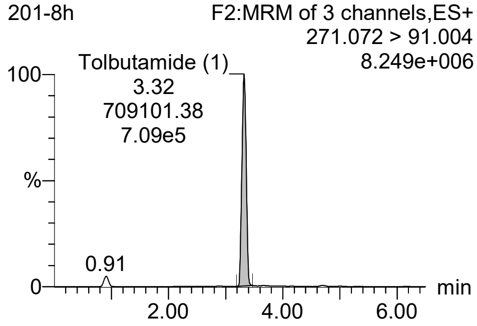

	#	Name	Trace	RT	Area	IS Area	Response	Primar...	Conc.	%Dev
1	1	WBPD081_041	1086.006 > 1330...	3.00	3420.565	709101.375	0.005	bb	228.5	
2	2	Tolbutamide (1)	271.072 > 91.004	3.32	709101.375		709101.375	bb	1.0	1.9

Dataset: D:\Data\27013-24001-NG.PRO\20241212_WBPD081_041_SA_Reinjection_Processed-Tu.qld

Last Altered: Tuesday, July 15, 2025 15:23:10 China Standard Time
Printed: Tuesday, July 15, 2025 15:40:15 China Standard Time

Name: 20241212_2_027, ID: 201-12h, Description:

WBPD081_041

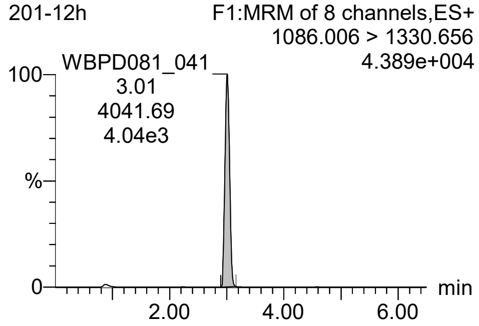

Tolbutamide (1)

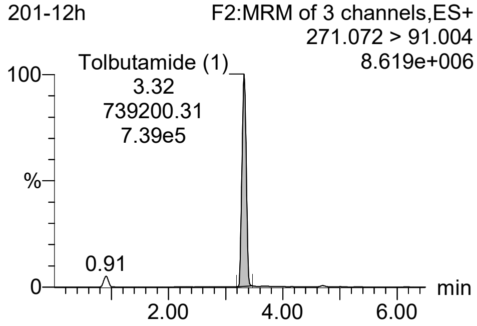

	#	Name	Trace	RT	Area	IS Area	Response	Primar...	Conc.	%Dev
1	1	WBPD081_041	1086.006 > 1330...	3.01	4041.691	739200.313	0.005	bb	259.0	
2	2	Tolbutamide (1)	271.072 > 91.004	3.32	739200.313		739200.313	bb	1.1	6.2

Name: 20241212_2_028, ID: 201-24h, Description:

WBPD081_041

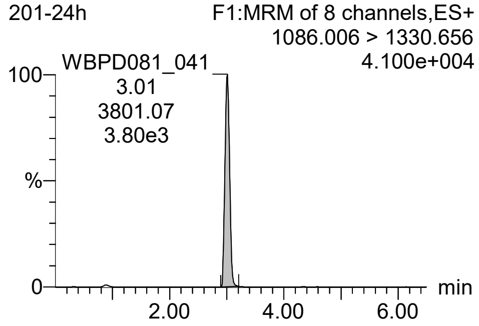

Tolbutamide (1)

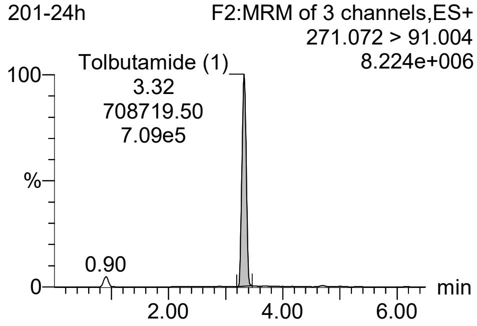

	#	Name	Trace	RT	Area	IS Area	Response	Primar...	Conc.	%Dev
1	1	WBPD081_041	1086.006 > 1330...	3.01	3801.075	708719.500	0.005	bb	254.0	
2	2	Tolbutamide (1)	271.072 > 91.004	3.32	708719.500		708719.500	bb	1.0	1.9

Name: 20241212_2_029, ID: 201-48h, Description:

WBPD081_041

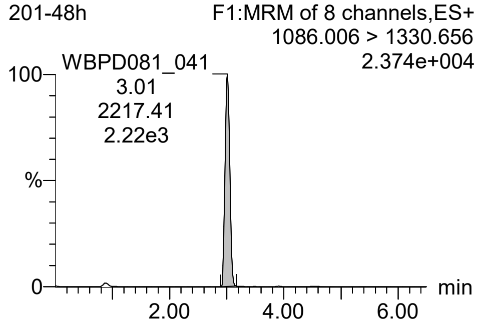

Tolbutamide (1)

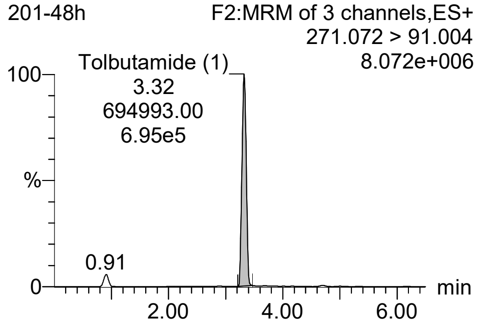

	#	Name	Trace	RT	Area	IS Area	Response	Primar...	Conc.	%Dev
1	1	WBPD081_041	1086.006 > 1330...	3.01	2217.406	694993.000	0.003	bb	151.2	
2	2	Tolbutamide (1)	271.072 > 91.004	3.32	694993.000		694993.000	bb	1.0	-0.1

Dataset: D:\Data\27013-24001-NG.PRO\20241212_WBPD081_041_SA_Reinjection_Processed-Tu.qld

Last Altered: Tuesday, July 15, 2025 15:23:10 China Standard Time
Printed: Tuesday, July 15, 2025 15:40:15 China Standard Time

Name: 20241212_2_030, ID: 201-72h, Description:

WBPD081_041

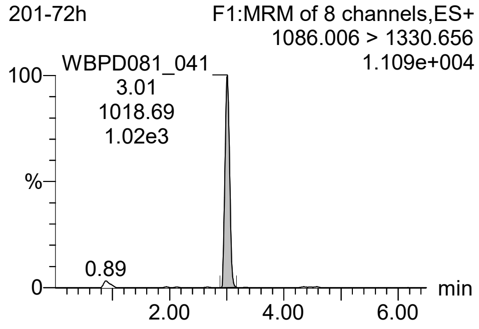

Tolbutamide (1)

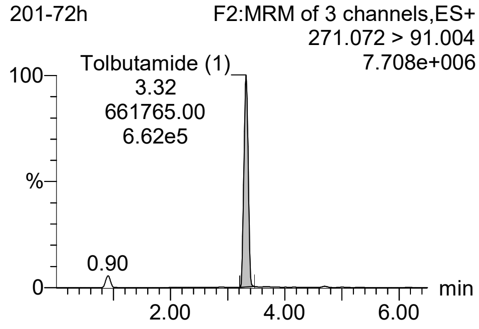

	#	Name	Trace	RT	Area	IS Area	Response	Primar...	Conc.	%Dev
1	1	WBPD081_041	1086.006 > 1330...	3.01	1018.693	661765.000	0.002	bb	73.1	
2	2	Tolbutamide (1)	271.072 > 91.004	3.32	661765.000		661765.000	bb	1.0	-4.9

Name: 20241212_2_031, ID: 201-96h, Description:

WBPD081_041

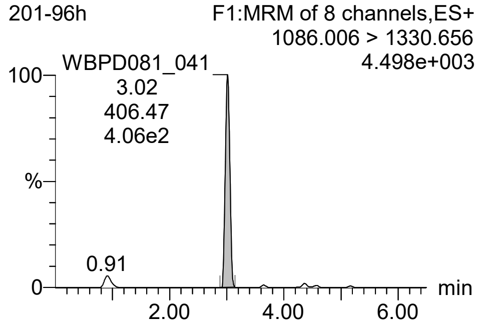

Tolbutamide (1)

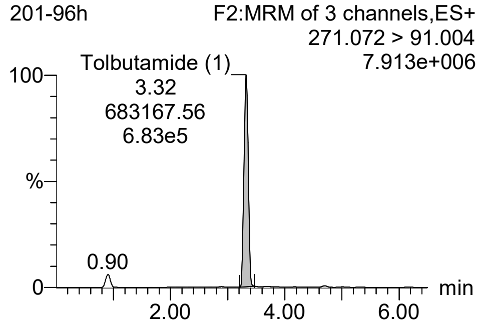

	#	Name	Trace	RT	Area	IS Area	Response	Primar...	Conc.	%Dev
1	1	WBPD081_041	1086.006 > 1330...	3.02	406.466	683167.563	0.001	bb	28.4	
2	2	Tolbutamide (1)	271.072 > 91.004	3.32	683167.563		683167.563	bb	1.0	-1.8

Name: 20241212_2_032, ID: 201-168h, Description:

WBPD081_041

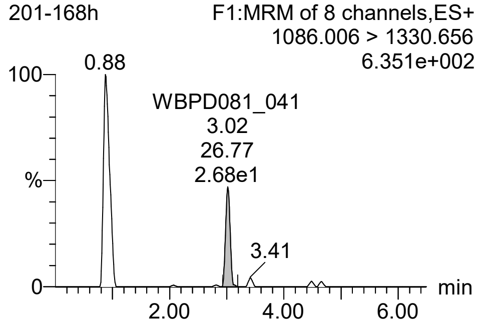

Tolbutamide (1)

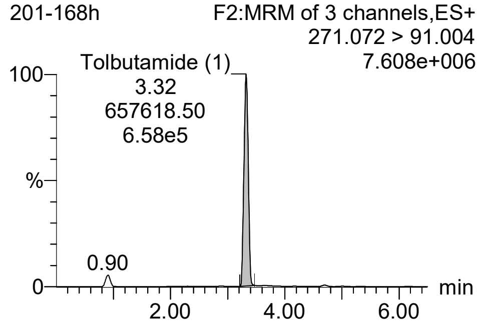

	#	Name	Trace	RT	Area	IS Area	Response	Primar...	Conc.	%Dev
1	1	WBPD081_041	1086.006 > 1330...	3.02	26.767	657618.500	0.000	bb	2.1	
2	2	Tolbutamide (1)	271.072 > 91.004	3.32	657618.500		657618.500	bb	0.9	-5.5

Dataset: D:\Data\27013-24001-NG.PRO\20241212_WBPD081_041_SA_Reinjection_Processed-Tu.qld

Last Altered: Tuesday, July 15, 2025 15:23:10 China Standard Time
Printed: Tuesday, July 15, 2025 15:40:15 China Standard Time

Name: 20241212_2_033, ID: Solvent, Description:

WBPD081_041

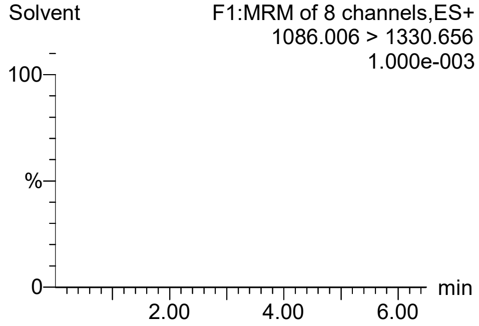

Tolbutamide (1)

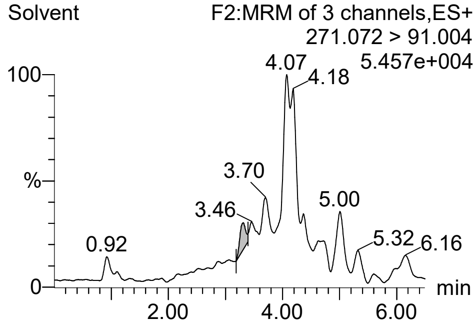

	#	Name	Trace	RT	Area	IS Area	Response	Primar...	Conc.	%Dev
1	1	WBPD081_041	1086.006 > 1330...			816.099				
2	2	Tolbutamide (1)	271.072 > 91.004	3.31	816.099		816.099	bd	0.0	-99.9

Name: 20241212_2_034, ID: Solvent, Description:

WBPD081_041

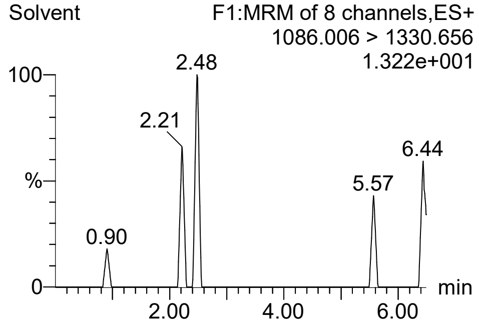

Tolbutamide (1)

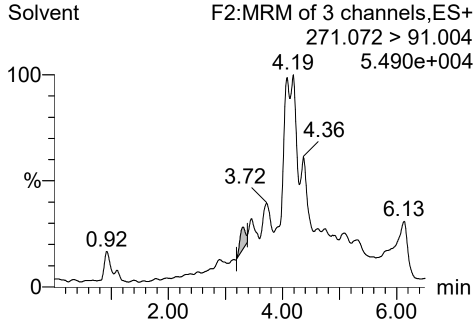

	#	Name	Trace	RT	Area	IS Area	Response	Primar...	Conc.	%Dev
1	1	WBPD081_041	1086.006 > 1330...			638.108				
2	2	Tolbutamide (1)	271.072 > 91.004	3.31	638.108		638.108	bd	0.0	-99.9

Name: 20241212_2_035, ID: 202-Predose, Description:

WBPD081_041

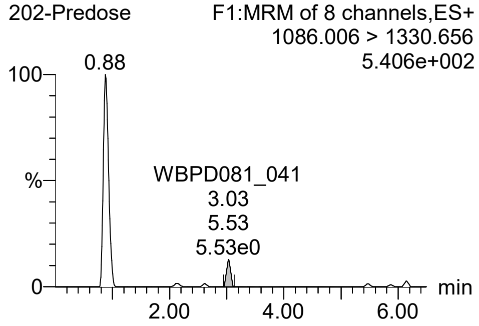

Tolbutamide (1)

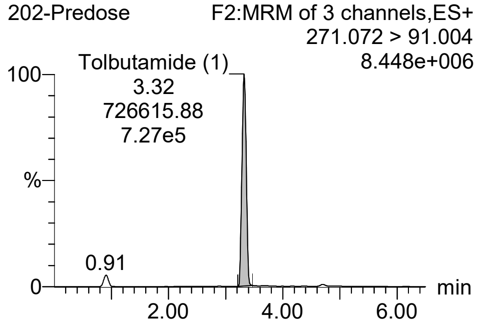

	#	Name	Trace	RT	Area	IS Area	Response	Primar...	Conc.	%Dev
1	1	WBPD081_041	1086.006 > 1330...	3.03	5.525	726615.875	0.000	bb	0.6	
2	2	Tolbutamide (1)	271.072 > 91.004	3.32	726615.875		726615.875	bb	1.0	4.4

Dataset: D:\Data\27013-24001-NG.PRO\20241212_WBPD081_041_SA_Reinjection_Processed-Tu.qld

Last Altered: Tuesday, July 15, 2025 15:23:10 China Standard Time
Printed: Tuesday, July 15, 2025 15:40:15 China Standard Time

Name: 20241212_2_036, ID: 202-2h, Description:

WBPD081_041

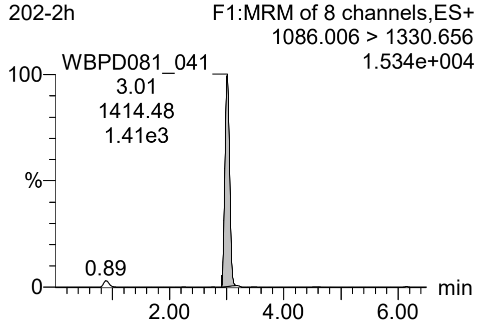

Tolbutamide (1)

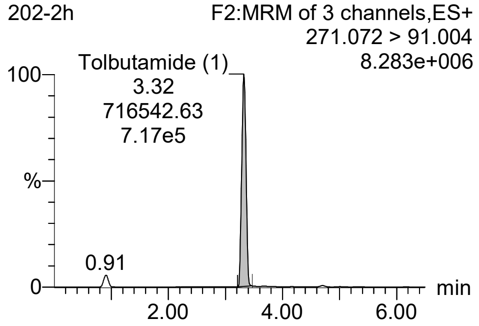

	#	Name	Trace	RT	Area	IS Area	Response	Primar...	Conc.	%Dev
1	1	WBPD081_041	1086.006 > 1330...	3.01	1414.476	716542.625	0.002	bb	93.6	
2	2	Tolbutamide (1)	271.072 > 91.004	3.32	716542.625		716542.625	bb	1.0	3.0

Name: 20241212_2_037, ID: 202-4h, Description:

WBPD081_041

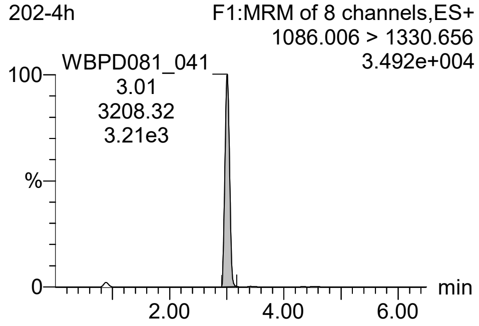

Tolbutamide (1)

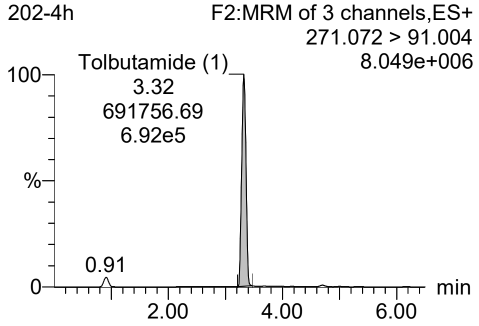

	#	Name	Trace	RT	Area	IS Area	Response	Primar...	Conc.	%Dev
1	1	WBPD081_041	1086.006 > 1330...	3.01	3208.318	691756.688	0.005	bb	219.7	
2	2	Tolbutamide (1)	271.072 > 91.004	3.32	691756.688		691756.688	bb	1.0	-0.6

Name: 20241212_2_038, ID: 202-8h, Description:

WBPD081_041

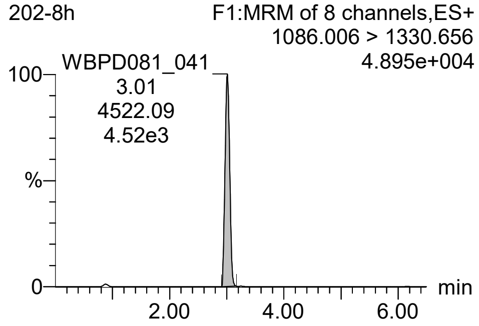

Tolbutamide (1)

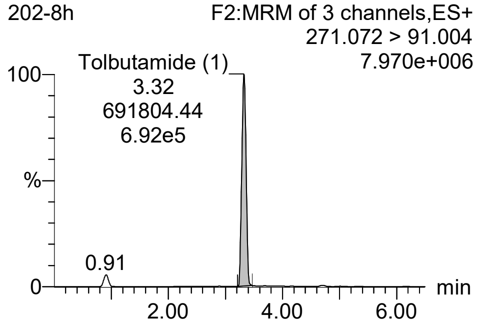

	#	Name	Trace	RT	Area	IS Area	Response	Primar...	Conc.	%Dev
1	1	WBPD081_041	1086.006 > 1330...	3.01	4522.092	691804.438	0.007	bb	309.6	
2	2	Tolbutamide (1)	271.072 > 91.004	3.32	691804.438		691804.438	bb	1.0	-0.6

Dataset: D:\Data\27013-24001-NG.PRO\20241212_WBPD081_041_SA_Reinjection_Processed-Tu.qld

Last Altered: Tuesday, July 15, 2025 15:23:10 China Standard Time
Printed: Tuesday, July 15, 2025 15:40:15 China Standard Time

Name: 20241212_2_039, ID: 202-12h, Description:

WBPD081_041

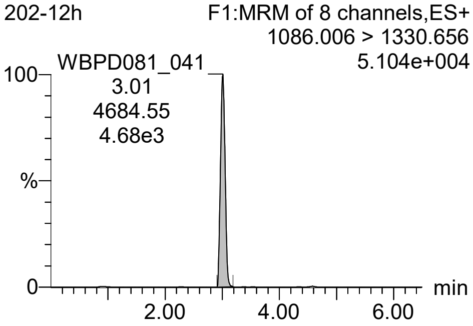

Tolbutamide (1)

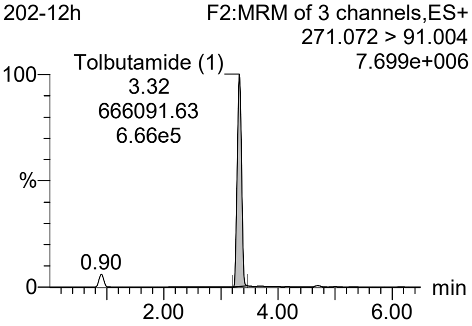

	#	Name	Trace	RT	Area	IS Area	Response	Primar...	Conc.	%Dev
1	1	WBPD081_041	1086.006 > 1330...	3.01	4684.553	666091.625	0.007	bb	333.0	
2	2	Tolbutamide (1)	271.072 > 91.004	3.32	666091.625		666091.625	bb	1.0	-4.3

Name: 20241212_2_040, ID: 202-24h, Description:

WBPD081_041

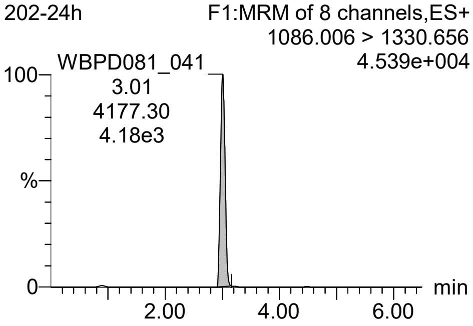

Tolbutamide (1)

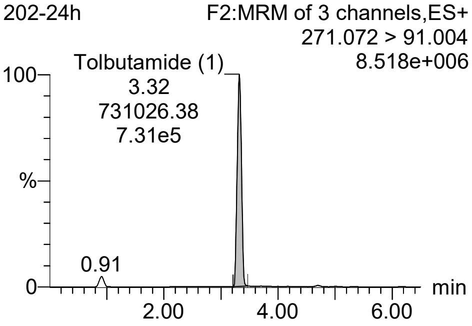

	#	Name	Trace	RT	Area	IS Area	Response	Primar...	Conc.	%Dev
1	1	WBPD081_041	1086.006 > 1330...	3.01	4177.303	731026.375	0.006	bb	270.6	
2	2	Tolbutamide (1)	271.072 > 91.004	3.32	731026.375		731026.375	bb	1.1	5.1

Name: 20241212_2_041, ID: 202-48h, Description:

WBPD081_041

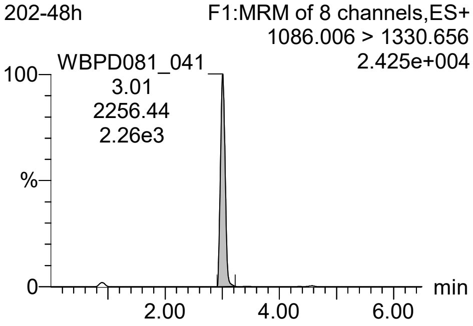

Tolbutamide (1)

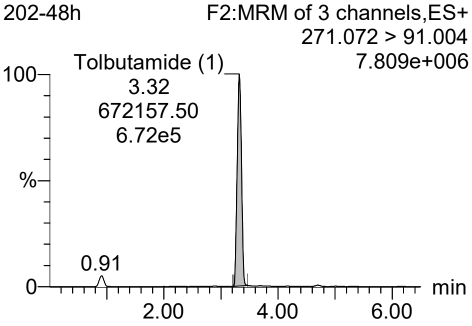

	#	Name	Trace	RT	Area	IS Area	Response	Primar...	Conc.	%Dev
1	1	WBPD081_041	1086.006 > 1330...	3.01	2256.442	672157.500	0.003	bb	159.1	
2	2	Tolbutamide (1)	271.072 > 91.004	3.32	672157.500		672157.500	bb	1.0	-3.4

Dataset: D:\Data\27013-24001-NG.PRO\20241212_WBPD081_041_SA_Reinjection_Processed-Tu.qld

Last Altered: Tuesday, July 15, 2025 15:23:10 China Standard Time
Printed: Tuesday, July 15, 2025 15:40:15 China Standard Time

Name: 20241212_2_042, ID: 202-72h, Description:

WBPD081_041

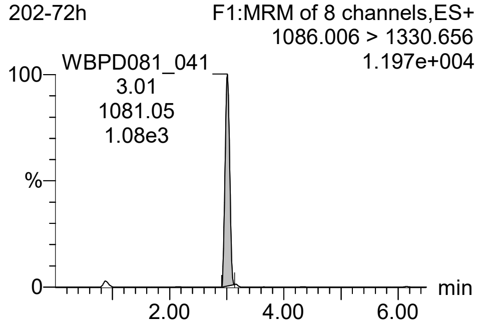

Tolbutamide (1)

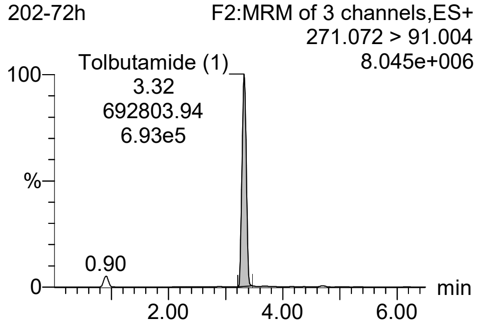

	#	Name	Trace	RT	Area	IS Area	Response	Primar...	Conc.	%Dev
1	1	WBPD081_041	1086.006 > 1330...	3.01	1081.048	692803.938	0.002	bb	74.0	
2	2	Tolbutamide (1)	271.072 > 91.004	3.32	692803.938		692803.938	bb	1.0	-0.4

Name: 20241212_2_043, ID: 202-96h, Description:

WBPD081_041

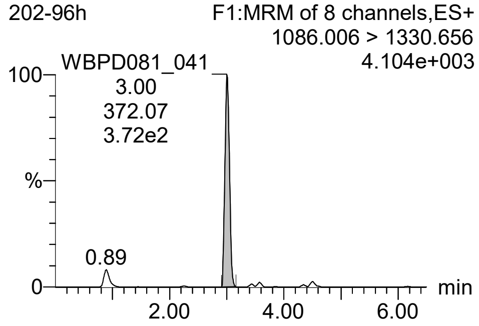

Tolbutamide (1)

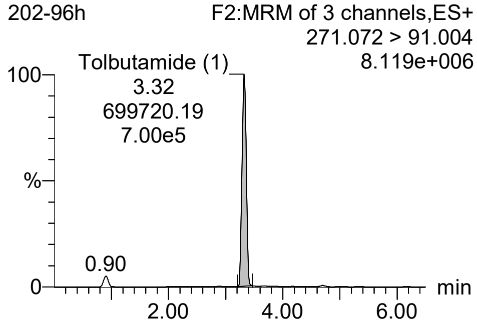

	#	Name	Trace	RT	Area	IS Area	Response	Primar...	Conc.	%Dev
1	1	WBPD081_041	1086.006 > 1330...	3.00	372.069	699720.188	0.001	bb	25.4	
2	2	Tolbutamide (1)	271.072 > 91.004	3.32	699720.188		699720.188	bb	1.0	0.6

Name: 20241212_2_044, ID: 202-168h, Description:

WBPD081_041

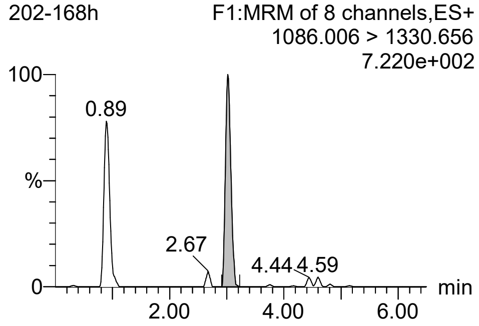

Tolbutamide (1)

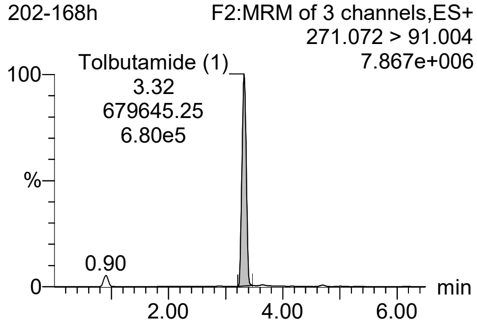

	#	Name	Trace	RT	Area	IS Area	Response	Primar...	Conc.	%Dev
1	1	WBPD081_041	1086.006 > 1330...	3.02	77.958	679645.250	0.000	bb	5.6	
2	2	Tolbutamide (1)	271.072 > 91.004	3.32	679645.250		679645.250	bb	1.0	-2.3

Dataset: D:\Data\27013-24001-NG.PRO\20241212_WBPD081_041_SA_Reinjection_Processed-Tu.qld

Last Altered: Tuesday, July 15, 2025 15:23:10 China Standard Time
Printed: Tuesday, July 15, 2025 15:40:15 China Standard Time

Name: 20241212_2_045, ID: Solvent, Description:

WBPD081_041

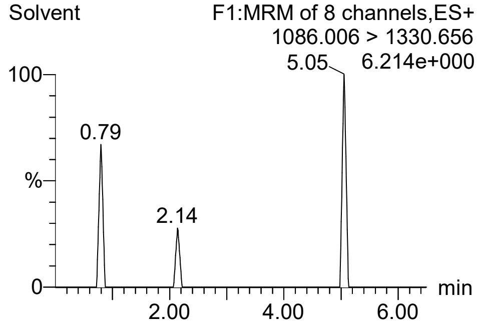

Tolbutamide (1)

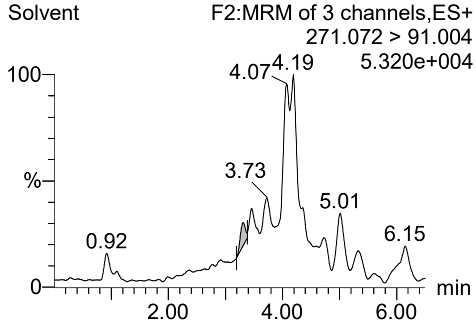

	#	Name	Trace	RT	Area	IS Area	Response	Primar...	Conc.	%Dev
1	1	WBPD081_041	1086.006 > 1330...			601.392				
2	2	Tolbutamide (1)	271.072 > 91.004	3.31	601.392		601.392	bd	0.0	-99.9

Name: 20241212_2_046, ID: Solvent, Description:

WBPD081_041

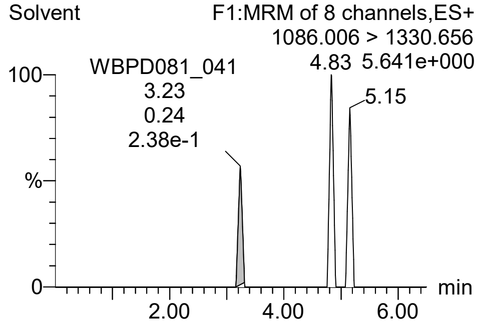

Tolbutamide (1)

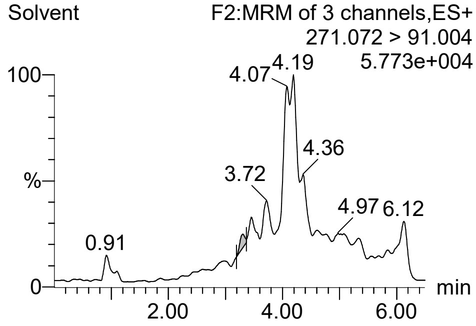

	#	Name	Trace	RT	Area	IS Area	Response	Primar...	Conc.	%Dev
1	1	WBPD081_041	1086.006 > 1330...	3.23	0.238	385.850	0.001	bb	29.4	
2	2	Tolbutamide (1)	271.072 > 91.004	3.30	385.850		385.850	bd	0.0	-99.9

Name: 20241212_2_047, ID: 203-Predose, Description:

WBPD081_041

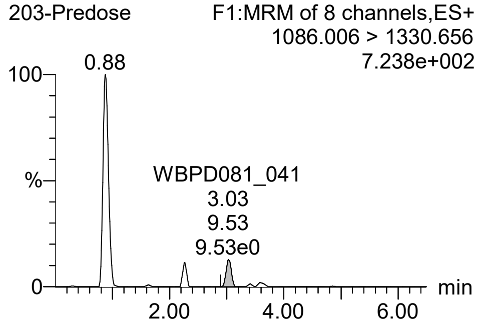

Tolbutamide (1)

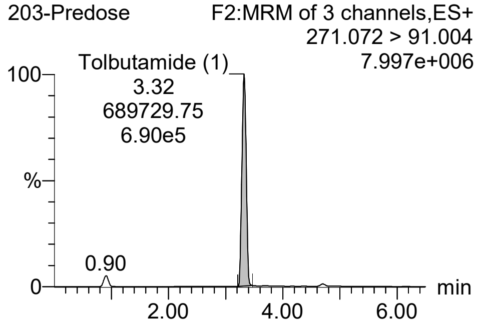

	#	Name	Trace	RT	Area	IS Area	Response	Primar...	Conc.	%Dev
1	1	WBPD081_041	1086.006 > 1330...	3.03	9.530	689729.750	0.000	bb	0.9	
2	2	Tolbutamide (1)	271.072 > 91.004	3.32	689729.750		689729.750	bb	1.0	-0.9

Dataset: D:\Data\27013-24001-NG.PRO\20241212_WBPD081_041_SA_Reinjection_Processed-Tu.qld

Last Altered: Tuesday, July 15, 2025 15:23:10 China Standard Time
Printed: Tuesday, July 15, 2025 15:40:15 China Standard Time

Name: 20241212_2_048, ID: 203-2h, Description:

WBPD081_041

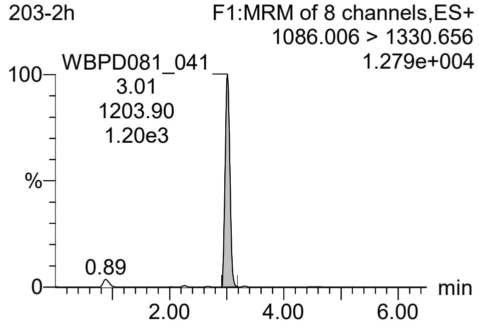

Tolbutamide (1)

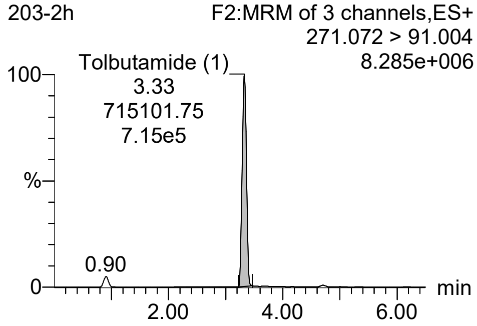

	# Name	Trace	RT	Area	IS Area	Response	Primar...	Conc.	%Dev
1	1 WBPD081_041	1086.006 > 1330...	3.01	1203.902	715101.750	0.002	bb	79.9	
2	2 Tolbutamide (1)	271.072 > 91.004	3.33	715101.750		715101.750	bb	1.0	2.8

Name: 20241212_2_049, ID: 203-4h, Description:

WBPD081_041

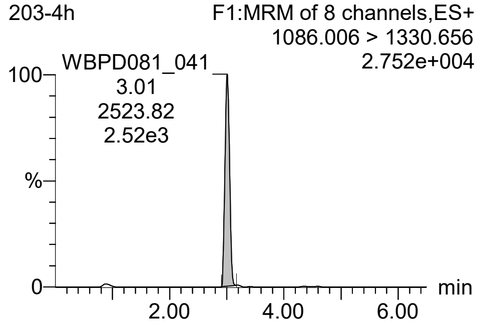

Tolbutamide (1)

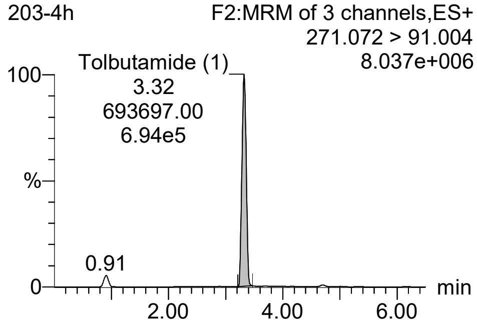

	# Name	Trace	RT	Area	IS Area	Response	Primar...	Conc.	%Dev
1	1 WBPD081_041	1086.006 > 1330...	3.01	2523.818	693697.000	0.004	bb	172.4	
2	2 Tolbutamide (1)	271.072 > 91.004	3.32	693697.000		693697.000	bb	1.0	-0.3

Name: 20241212_2_050, ID: 203-8h, Description:

WBPD081_041

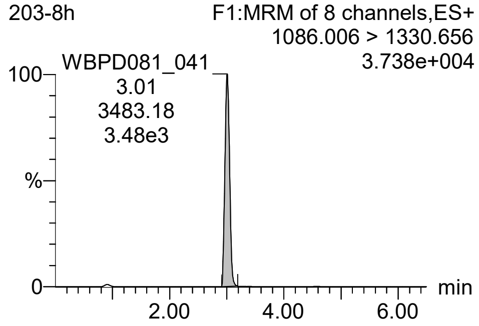

Tolbutamide (1)

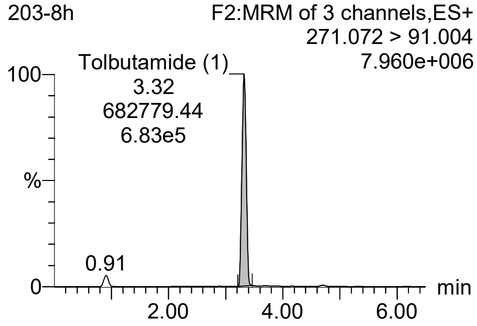

	# Name	Trace	RT	Area	IS Area	Response	Primar...	Conc.	%Dev
1	1 WBPD081_041	1086.006 > 1330...	3.01	3483.185	682779.438	0.005	bb	241.6	
2	2 Tolbutamide (1)	271.072 > 91.004	3.32	682779.438		682779.438	bb	1.0	-1.9

Dataset: D:\Data\27013-24001-NG.PRO\20241212_WBPD081_041_SA_Reinjection_Processed-Tu.qld

Last Altered: Tuesday, July 15, 2025 15:23:10 China Standard Time
Printed: Tuesday, July 15, 2025 15:40:15 China Standard Time

Name: 20241212_2_051, ID: 203-12h, Description:

WBPD081_041

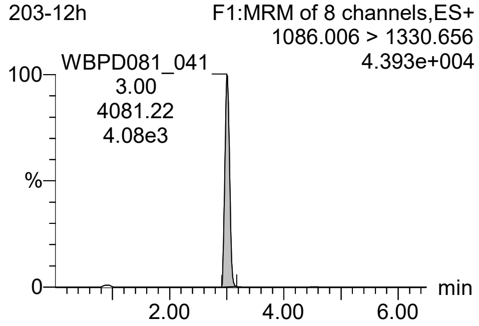

Tolbutamide (1)

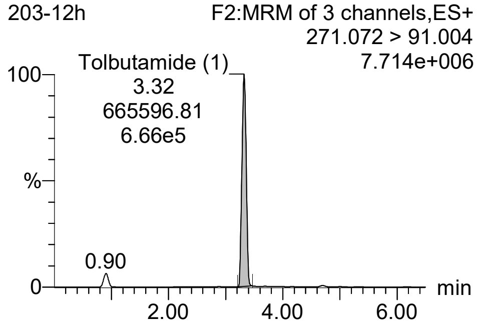

	#	Name	Trace	RT	Area	IS Area	Response	Primar...	Conc.	%Dev
1	1	WBPD081_041	1086.006 > 1330...	3.00	4081.217	665596.813	0.006	bb	290.4	
2	2	Tolbutamide (1)	271.072 > 91.004	3.32	665596.813		665596.813	bb	1.0	-4.3

Name: 20241212_2_052, ID: 203-24h, Description:

WBPD081_041

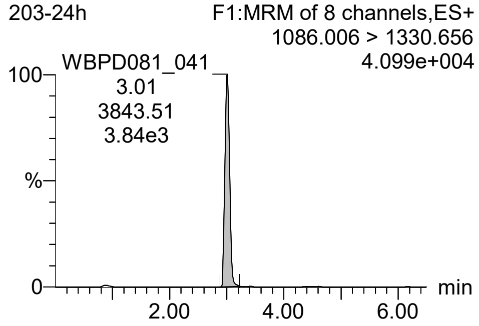

Tolbutamide (1)

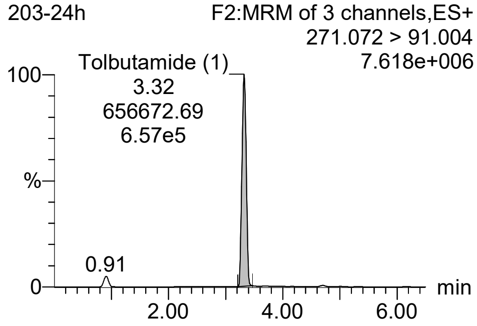

	#	Name	Trace	RT	Area	IS Area	Response	Primar...	Conc.	%Dev
1	1	WBPD081_041	1086.006 > 1330...	3.01	3843.513	656672.688	0.006	bb	277.2	
2	2	Tolbutamide (1)	271.072 > 91.004	3.32	656672.688		656672.688	bb	0.9	-5.6

Name: 20241212_2_053, ID: 203-48h, Description:

WBPD081_041

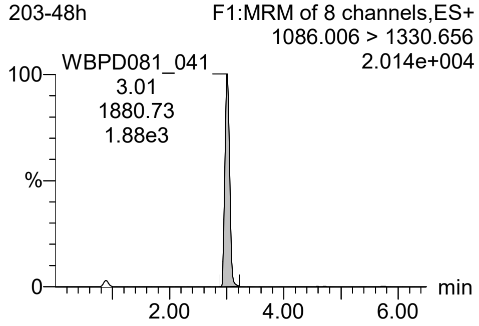

Tolbutamide (1)

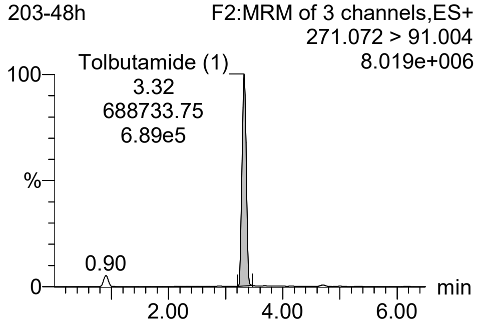

	#	Name	Trace	RT	Area	IS Area	Response	Primar...	Conc.	%Dev
1	1	WBPD081_041	1086.006 > 1330...	3.01	1880.726	688733.750	0.003	bb	129.4	
2	2	Tolbutamide (1)	271.072 > 91.004	3.32	688733.750		688733.750	bb	1.0	-1.0

Dataset: D:\Data\27013-24001-NG.PRO\20241212_WBPD081_041_SA_Reinjection_Processed-Tu.qld

Last Altered: Tuesday, July 15, 2025 15:23:10 China Standard Time
Printed: Tuesday, July 15, 2025 15:40:15 China Standard Time

Name: 20241212_2_054, ID: 203-72h, Description:

WBPD081_041

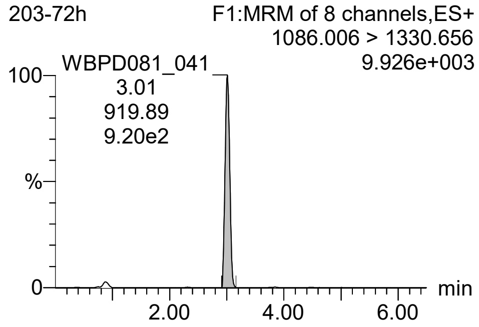

Tolbutamide (1)

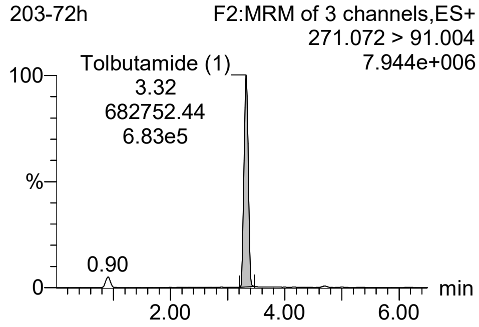

	#	Name	Trace	RT	Area	IS Area	Response	Primar...	Conc.	%Dev
1	1	WBPD081_041	1086.006 > 1330...	3.01	919.890	682752.438	0.001	bb	64.0	
2	2	Tolbutamide (1)	271.072 > 91.004	3.32	682752.438		682752.438	bb	1.0	-1.9

Name: 20241212_2_055, ID: 203-96h, Description:

WBPD081_041

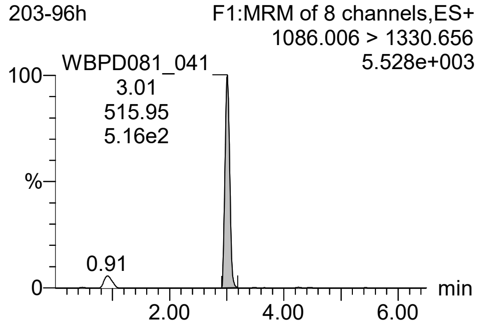

Tolbutamide (1)

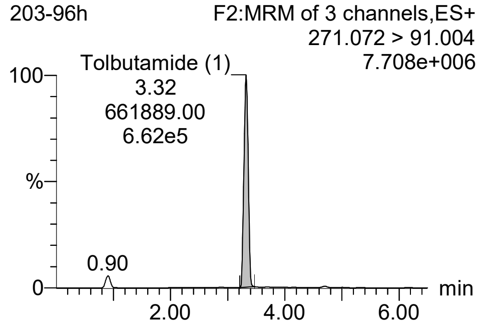

	#	Name	Trace	RT	Area	IS Area	Response	Primar...	Conc.	%Dev
1	1	WBPD081_041	1086.006 > 1330...	3.01	515.954	661889.000	0.001	bb	37.1	
2	2	Tolbutamide (1)	271.072 > 91.004	3.32	661889.000		661889.000	bb	1.0	-4.9

Name: 20241212_2_056, ID: 203-168h, Description:

WBPD081_041

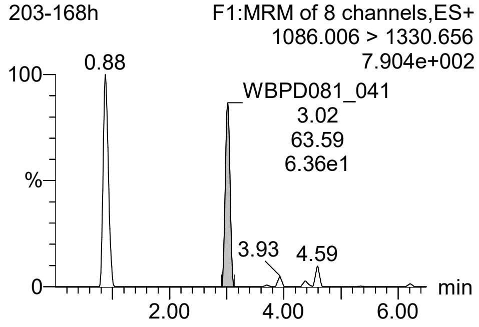

Tolbutamide (1)

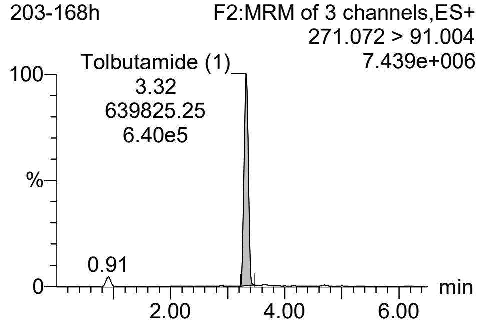

	#	Name	Trace	RT	Area	IS Area	Response	Primar...	Conc.	%Dev
1	1	WBPD081_041	1086.006 > 1330...	3.02	63.591	639825.250	0.000	bb	4.9	
2	2	Tolbutamide (1)	271.072 > 91.004	3.32	639825.250		639825.250	bb	0.9	-8.1

Dataset: D:\Data\27013-24001-NG.PRO\20241212_WBPD081_041_SA_Reinjection_Processed-Tu.qld

Last Altered: Tuesday, July 15, 2025 15:23:10 China Standard Time
Printed: Tuesday, July 15, 2025 15:40:15 China Standard Time

Name: 20241212_2_057, ID: Solvent, Description:

WBPD081_041

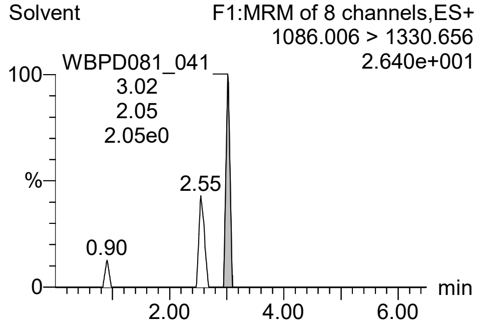

Tolbutamide (1)

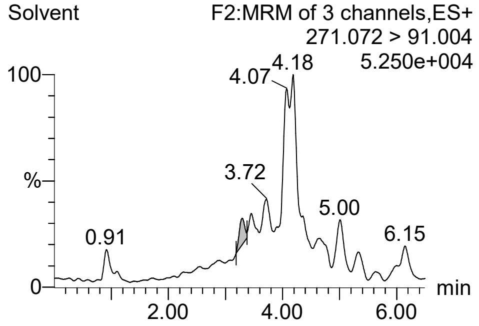

	#	Name	Trace	RT	Area	IS Area	Response	Primar...	Conc.	%Dev
1	1	WBPD081_041	1086.006 > 1330...	3.02	2.048	693.137	0.003	bb	140.0	
2	2	Tolbutamide (1)	271.072 > 91.004	3.29	693.137		693.137	bd	0.0	-99.9

Name: 20241212_2_058, ID: Solvent, Description:

WBPD081_041

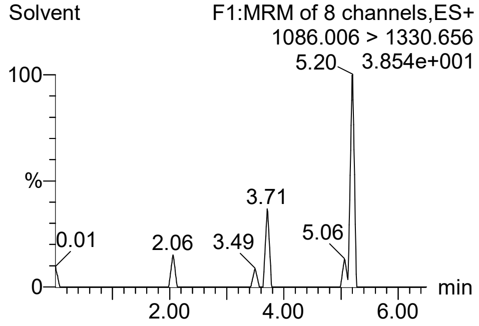

Tolbutamide (1)

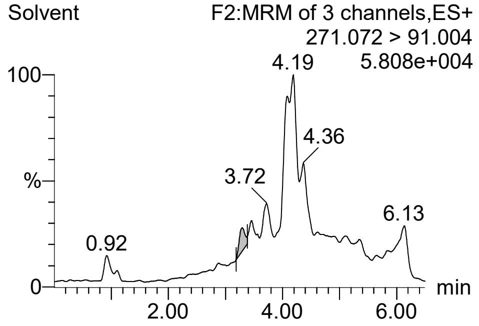

	#	Name	Trace	RT	Area	IS Area	Response	Primar...	Conc.	%Dev
1	1	WBPD081_041	1086.006 > 1330...			755.455				
2	2	Tolbutamide (1)	271.072 > 91.004	3.29	755.455		755.455	bd	0.0	-99.9

Name: 20241212_2_059, ID: B, Description:

WBPD081_041

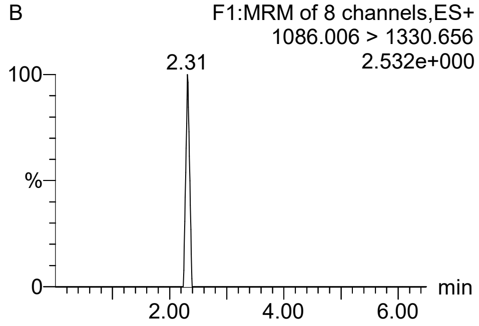

Tolbutamide (1)

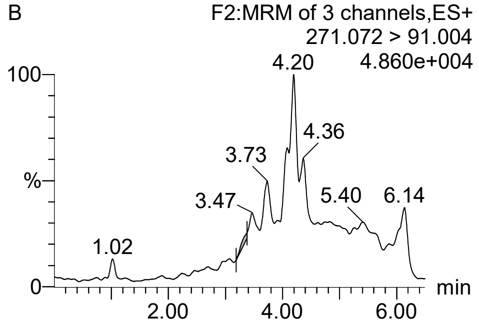

	#	Name	Trace	RT	Area	IS Area	Response	Primar...	Conc.	%Dev
1	1	WBPD081_041	1086.006 > 1330...			191.490				
2	2	Tolbutamide (1)	271.072 > 91.004	3.38	191.490		191.490	bd	0.0	-100.0

Dataset: D:\Data\27013-24001-NG.PRO\20241212_WBPD081_041_SA_Reinjection_Processed-Tu.qld

Last Altered: Tuesday, July 15, 2025 15:23:10 China Standard Time
Printed: Tuesday, July 15, 2025 15:40:15 China Standard Time

Name: 20241212_2_060, ID: O, Description:

WBPD081_041

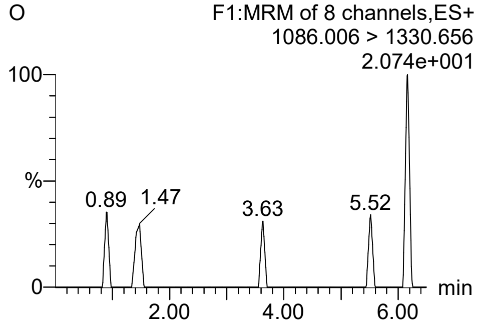

Tolbutamide (1)

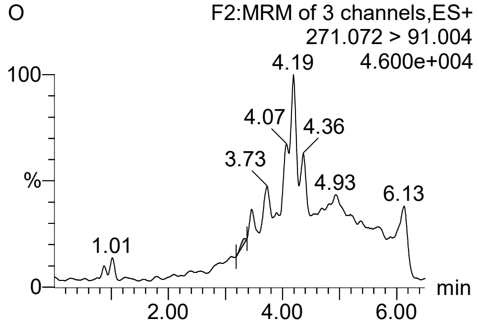

	# Name	Trace	RT	Area	IS Area	Response	Primar...	Conc.	%Dev
1	1 WBPD081_041	1086.006 > 1330...			108.018				
2	2 Tolbutamide (1)	271.072 > 91.004	3.34	108.018		108.018	bb	0.0	-100.0

Name: 20241212_2_061, ID: Q1, Description:

WBPD081_041

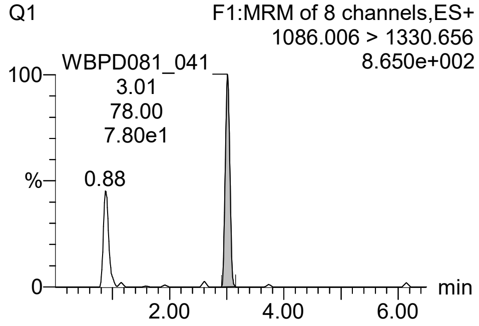

Tolbutamide (1)

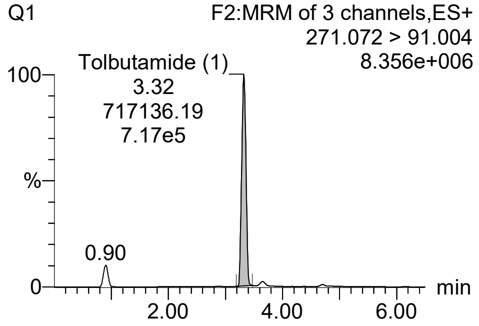

	# Name	Trace	RT	Area	IS Area	Response	Primar...	Conc.	%Dev
1	1 WBPD081_041	1086.006 > 1330...	3.01	78.004	717136.188	0.000	bb	5.3	-10.9
2	2 Tolbutamide (1)	271.072 > 91.004	3.32	717136.188		717136.188	bb	1.0	3.1

Name: 20241212_2_062, ID: Q2, Description:

WBPD081_041

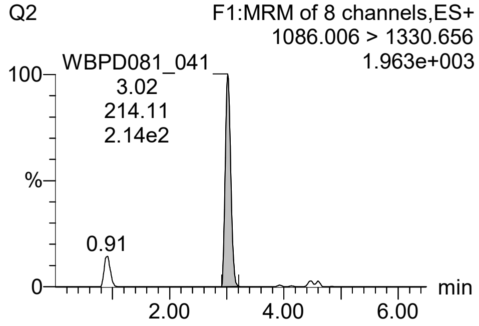

Tolbutamide (1)

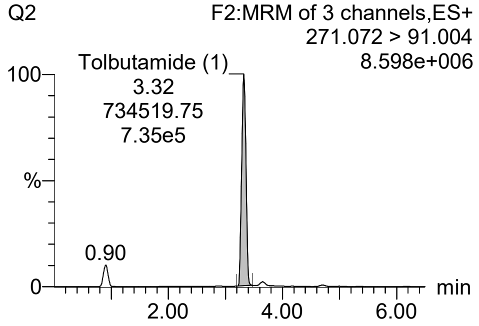

	# Name	Trace	RT	Area	IS Area	Response	Primar...	Conc.	%Dev
1	1 WBPD081_041	1086.006 > 1330...	3.02	214.106	734519.750	0.000	bb	14.0	16.6
2	2 Tolbutamide (1)	271.072 > 91.004	3.32	734519.750		734519.750	bb	1.1	5.6

Dataset: D:\Data\27013-24001-NG.PRO\20241212_WBPD081_041_SA_Reinjection_Processed-Tu.qld

Last Altered: Tuesday, July 15, 2025 15:23:10 China Standard Time
Printed: Tuesday, July 15, 2025 15:40:15 China Standard Time

Name: 20241212_2_063, ID: Q3, Description:

WBPD081_041

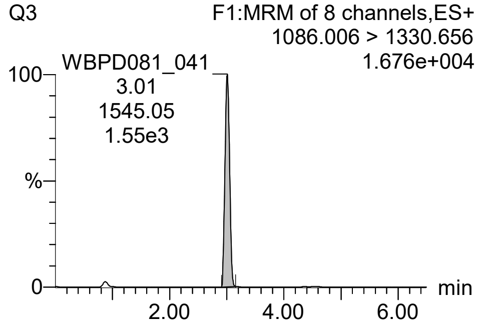

Tolbutamide (1)

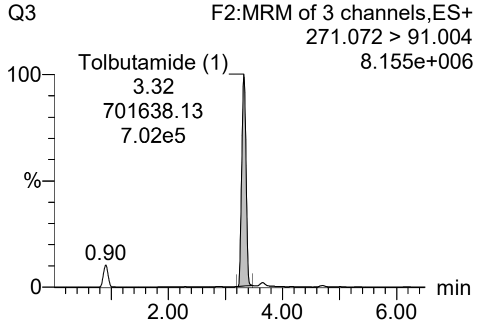

	#	Name	Trace	RT	Area	IS Area	Response	Primar...	Conc.	%Dev
1	1	WBPD081_041	1086.006 > 1330...	3.01	1545.050	701638.125	0.002	bb	104.4	30.5
2	2	Tolbutamide (1)	271.072 > 91.004	3.32	701638.125		701638.125	bb	1.0	0.8

Name: 20241212_2_064, ID: Q4, Description:

WBPD081_041

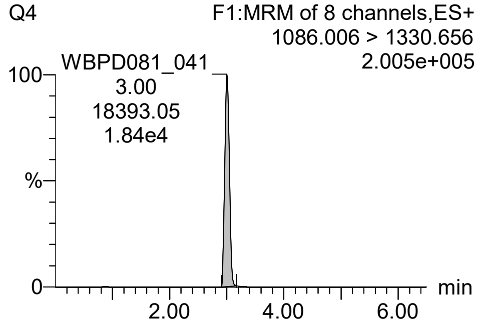

Tolbutamide (1)

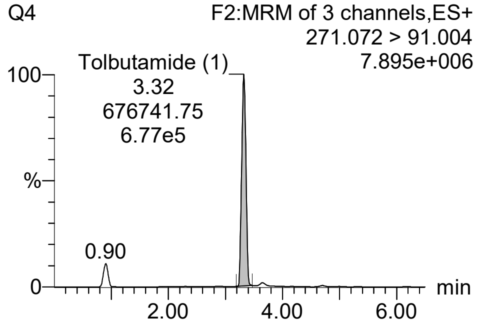

	#	Name	Trace	RT	Area	IS Area	Response	Primar...	Conc.	%Dev
1	1	WBPD081_041	1086.006 > 1330...	3.00	18393.049	676741.750	0.027	bb	1286.5	60.8
2	2	Tolbutamide (1)	271.072 > 91.004	3.32	676741.750		676741.750	bb	1.0	-2.7
