# Supplementary material for: AI‐Driven De Novo Design of Ultra Long‐Acting GLP‐1 Receptor Agonists
Source: Adv Sci (Weinh). 2025 Aug 11;12(40):e07044. doi: 10.1002/advs.202507044 (PMC12561408; doi:10.1002/advs.202507044)
Supplement: Supplementary file 2 — Supporting Information [file ADVS-12-e07044-s001.zip › SI_The raw data of PK for D44 .pdf]

Dataset: D:\Data\27013-24001-NG.PRO\20241214_WBPD081_044_SA-Tu.qld

Last Altered: Tuesday, July 15, 2025 15:34:14 China Standard Time
Printed: Tuesday, July 15, 2025 15:38:44 China Standard Time

Method: D:\Data\27013-24001-NG.PRO\MethDB\20241214_WBPD081_044.mdb 14 Dec 2024 09:14:43
Calibration: 15 Jul 2025 15:34:14

Compound name: WBPD081_044 (2)
Correlation coefficient: r = 0.996092, r^2 = 0.992198
Calibration curve: 0.000257808 * x + 7.07839e-005
Response type: Internal Std (Ref 2), Area * (IS Conc. / IS Area)
Curve type: Linear, Origin: Exclude, Weighting: 1/x^2, Axis trans: None

	Name	ID	Type	Std. Conc	RT	Area	IS Area	Response	Conc.	%Dev	Primar...
1	20241213_1_001	Solvent					223.212				
2	20241213_1_002	Solvent					105.950				
3	20241213_1_003	B	Blank				966.763				
4	20241213_1_004	O	Blank				166100.984				
5	20241213_1_005	STD1	Standard	2.000	3.00	105.531	172633.469	0.001	2.1	4.8	bb
6	20241213_1_006	STD2	Standard	5.000	3.00	218.925	184224.625	0.001	4.3	-13.3	bb
7	20241213_1_007	STD3	Standard	10.000	3.00	320.699	184940.984	0.002	6.5	-35.5	bbX
8	20241213_1_008	STD4	Standard	50.000	2.99	2617.288	185643.375	0.014	54.4	8.8	bb
9	20241213_1_009	STD5	Standard	100.000	2.99	4811.378	173205.813	0.028	107.5	7.5	bb
10	20241213_1_010	STD6	Standard	500.000	2.99	23121.529	173955.016	0.133	515.3	3.1	bb
11	20241213_1_011	STD7	Standard	900.000	2.99	41948.273	193185.141	0.217	842.0	-6.4	bb
12	20241213_1_012	STD8	Standard	1000.000	2.99	43337.594	175851.391	0.246	955.6	-4.4	bb
13	20241213_1_013	Solvent			3.00	285.179	207.174	1.377	5339.0		bb
14	20241213_1_014	Solvent			3.00	3.125	209.998	0.015	57.4		bb
15	20241213_1_015	B	Blank		3.01	57.816	686.876	0.084	326.2		bb
16	20241213_1_016	O	Blank		3.02	36.346	166523.125	0.000	0.6		bb
17	20241213_1_017	Q1	QC	6.000	2.99	275.202	174480.031	0.002	5.8	-2.6	bb
18	20241213_1_018	Q2	QC	12.000	3.00	478.111	176341.109	0.003	10.2	-14.6	bb
19	20241213_1_019	Q3	QC	80.000	3.00	3304.283	174852.719	0.019	73.0	-8.7	bb
20	20241213_1_020	Q4	QC	800.000	2.99	37337.980	174910.000	0.213	827.7	3.5	bb
21	20241213_1_021	Solvent			2.99	164.682	238.528	0.690	2677.7		bb
22	20241213_1_022	Solvent			3.26	1.192	371.020	0.003	12.2		bb
23	20241213_1_023	301-Predose			3.00	26.116	173215.938	0.000	0.3		bb
24	20241213_1_024	301-2h			3.00	5388.515	167079.500	0.032	124.8		bb
25	20241213_1_025	301-4h			2.99	6280.532	179547.266	0.035	135.4		bb
26	20241213_1_026	301-8h			2.99	4037.921	185515.109	0.022	84.2		bb
27	20241213_1_027	301-12h			3.00	2196.241	181237.422	0.012	46.7		bb
28	20241213_1_028	301-24h			3.00	319.508	171527.063	0.002	7.0		bb
29	20241213_1_029	301-48h			2.98	12.961	164376.797	0.000	0.0		db
30	20241213_1_030	301-72h			3.04	3.368	172990.563	0.000			bbI
31	20241213_1_031	301-96h					174580.047				
32	20241213_1_032	301-168h			3.05	4.666	173876.813	0.000			bbI
33	20241213_1_033	Solvent					252.404				
34	20241213_1_034	Solvent									
35	20241213_1_035	302-Predose			3.05	6.513	169676.547	0.000			dbI
36	20241213_1_036	302-2h			2.99	3231.922	168015.891	0.019	74.3		bb
37	20241213_1_037	302-4h			2.99	5348.600	173324.406	0.031	119.4		bb
38	20241213_1_038	302-8h			2.99	4186.138	174418.766	0.024	92.8		bb
39	20241213_1_039	302-12h			3.00	2234.172	180458.297	0.012	47.7		bb
40	20241213_1_040	302-24h			3.00	234.392	173674.313	0.001	5.0		bb
41	20241213_1_041	302-48h			3.04	18.022	178341.938	0.000	0.1		bb
42	20241213_1_042	302-72h			3.03	7.655	174252.781	0.000			bbI
43	20241213_1_043	302-96h			3.06	0.244	183150.172	0.000			bbI
44	20241213_1_044	302-168h			3.01	1.142	179493.016	0.000			bbI

Dataset: D:\Data\27013-24001-NG.PRO\20241214_WBPD081_044_SA-Tu.qld

Last Altered: Tuesday, July 15, 2025 15:34:14 China Standard Time
Printed: Tuesday, July 15, 2025 15:38:44 China Standard Time

Compound name: WBPD081_044 (2)

	Name	ID	Type	Std. Conc	RT	Area	IS Area	Response	Conc.	%Dev	Primar...
45	20241213_1_045	Solvent					207.711				
46	20241213_1_046	Solvent					207.678				
47	20241213_1_047	303-Predose					174508.656				
48	20241213_1_048	303-2h			3.00	3124.932	170822.516	0.018	70.7		bb
49	20241213_1_049	303-4h			3.00	4292.446	174797.438	0.025	95.0		bb
50	20241213_1_050	303-8h			2.99	3767.555	181532.547	0.021	80.2		bb
51	20241213_1_051	303-12h			2.99	2004.371	179024.906	0.011	43.2		bb
52	20241213_1_052	303-24h			3.00	290.145	168124.250	0.002	6.4		bb
53	20241213_1_053	303-48h			3.00	33.458	178857.688	0.000	0.5		bb
54	20241213_1_054	303-72h			3.05	1.415	180031.281	0.000			bbI
55	20241213_1_055	303-96h					176112.359				
56	20241213_1_056	303-168h			2.90	2.180	183235.672	0.000			bbI
57	20241213_1_057	Solvent					270.256				
58	20241213_1_058	Solvent					320.126				
59	20241213_1_059	B	Blank		3.02	6.577	1105.917	0.006	22.8		bb
60	20241213_1_060	O	Blank		3.00	3.643	187645.547	0.000			bbI
61	20241213_1_061	Q1	QC	6.000	3.00	263.514	186933.891	0.001	5.2	-13.4	bb
62	20241213_1_062	Q2	QC	12.000	3.00	698.430	188219.719	0.004	14.1	17.7	bb
63	20241213_1_063	Q3	QC	80.000	2.99	4134.252	182677.516	0.023	87.5	9.4	bb
64	20241213_1_064	Q4	QC	800.000	2.99	46000.078	181096.953	0.254	985.0	23.1	bb
65	20241213_1_065	Solvent			2.98	228.355	321.493	0.710	2754.9		bb
66	20241213_1_066	Solvent					374.246				

Dataset: D:\Data\27013-24001-NG.PRO\20241214_WBPD081_044_SA-Tu.qld

Last Altered: Tuesday, July 15, 2025 15:34:14 China Standard Time
Printed: Tuesday, July 15, 2025 15:38:44 China Standard Time

Compound name: WBPD081_044 (2)

	Inj. Vol	Factor1	Vial
1	10.000	0.0	3:H,12
2	10.000	0.0	3:H,12
3	10.000	0.0	3:A,1
4	10.000	0.0	3:A,2
5	10.000	1.0	3:A,3
6	10.000	1.0	3:A,4
7	10.000	1.0	3:A,5
8	10.000	1.0	3:A,6
9	10.000	1.0	3:A,7
10	10.000	1.0	3:A,8
11	10.000	1.0	3:A,9
12	10.000	1.0	3:A,10
13	10.000	0.0	3:H,12
14	10.000	0.0	3:H,12
15	10.000	0.0	3:A,1
16	10.000	0.0	3:A,2
17	10.000	0.0	3:A,11
18	10.000	0.0	3:A,12
19	10.000	0.0	3:B,1
20	10.000	0.0	3:B,2
21	10.000	0.0	3:H,12
22	10.000	0.0	3:H,12
23	10.000	0.0	3:B,7
24	10.000	0.0	3:B,8
25	10.000	0.0	3:B,9
26	10.000	0.0	3:B,10
27	10.000	0.0	3:B,11
28	10.000	0.0	3:B,12
29	10.000	0.0	3:C,1
30	10.000	0.0	3:C,2
31	10.000	0.0	3:C,3
32	10.000	0.0	3:C,4
33	10.000	0.0	3:H,12
34	10.000	0.0	3:H,12
35	10.000	0.0	3:C,5
36	10.000	0.0	3:C,6
37	10.000	0.0	3:C,7
38	10.000	0.0	3:C,8
39	10.000	0.0	3:C,9
40	10.000	0.0	3:C,10
41	10.000	0.0	3:C,11
42	10.000	0.0	3:C,12
43	10.000	0.0	3:D,1
44	10.000	0.0	3:D,2
45	10.000	0.0	3:H,12
46	10.000	0.0	3:H,12
47	10.000	0.0	3:D,3
48	10.000	0.0	3:D,4
49	10.000	0.0	3:D,5
50	10.000	0.0	3:D,6
51	10.000	0.0	3:D,7

Dataset: D:\Data\27013-24001-NG.PRO\20241214_WBPD081_044_SA-Tu.qld

Last Altered: Tuesday, July 15, 2025 15:34:14 China Standard Time
Printed: Tuesday, July 15, 2025 15:38:44 China Standard Time

Compound name: WBPD081_044 (2)

	Inj. Vol	Factor1	Vial
52	10.000	0.0	3:D,8
53	10.000	0.0	3:D,9
54	10.000	0.0	3:D,10
55	10.000	0.0	3:D,11
56	10.000	0.0	3:D,12
57	10.000	0.0	3:H,12
58	10.000	0.0	3:H,12
59	10.000	0.0	3:A,1
60	10.000	0.0	3:A,2
61	10.000	0.0	3:B,3
62	10.000	0.0	3:B,4
63	10.000	0.0	3:B,5
64	10.000	0.0	3:B,6
65	10.000	0.0	3:H,12
66	10.000	0.0	3:H,12

Dataset: D:\Data\27013-24001-NG.PRO\20241214_WBPD081_044_SA-Tu.qld

Last Altered: Tuesday, July 15, 2025 15:34:14 China Standard Time
Printed: Tuesday, July 15, 2025 15:38:44 China Standard Time

Compound name: Tolbutamide (1)
Response Factor: 180455
RRF SD: 7563.82, Relative SD: 4.19153
Response type: External Std, Area
Curve type: RF

	Name	ID	Type	Std. Conc	RT	Area	IS Area	Response	Conc.	%Dev	Primar...
1	20241213_1_001	Solvent		1.000	3.30	223.212		223.212	0.0	-99.9	bb
2	20241213_1_002	Solvent		1.000	3.30	105.950		105.950	0.0	-99.9	bb
3	20241213_1_003	B	Blank	1.000	3.30	966.763		966.763	0.0	-99.5	bb
4	20241213_1_004	O	Blank	1.000	3.32	166100.984		166100.984	0.9	-8.0	bb
5	20241213_1_005	STD1	Standard	1.000	3.32	172633.469		172633.469	1.0	-4.3	bb
6	20241213_1_006	STD2	Standard	1.000	3.32	184224.625		184224.625	1.0	2.1	bb
7	20241213_1_007	STD3	Standard	1.000	3.32	184940.984		184940.984	1.0	2.5	bb
8	20241213_1_008	STD4	Standard	1.000	3.31	185643.375		185643.375	1.0	2.9	bb
9	20241213_1_009	STD5	Standard	1.000	3.31	173205.813		173205.813	1.0	-4.0	bb
10	20241213_1_010	STD6	Standard	1.000	3.31	173955.016		173955.016	1.0	-3.6	bb
11	20241213_1_011	STD7	Standard	1.000	3.31	193185.141		193185.141	1.1	7.1	bb
12	20241213_1_012	STD8	Standard	1.000	3.31	175851.391		175851.391	1.0	-2.6	bb
13	20241213_1_013	Solvent		1.000	3.35	207.174		207.174	0.0	-99.9	bb
14	20241213_1_014	Solvent		1.000	3.30	209.998		209.998	0.0	-99.9	db
15	20241213_1_015	B	Blank	1.000	3.30	686.876		686.876	0.0	-99.6	bb
16	20241213_1_016	O	Blank	1.000	3.31	166523.125		166523.125	0.9	-7.7	bb
17	20241213_1_017	Q1	QC	1.000	3.31	174480.031		174480.031	1.0	-3.3	bb
18	20241213_1_018	Q2	QC	1.000	3.31	176341.109		176341.109	1.0	-2.3	bb
19	20241213_1_019	Q3	QC	1.000	3.31	174852.719		174852.719	1.0	-3.1	bb
20	20241213_1_020	Q4	QC	1.000	3.31	174910.000		174910.000	1.0	-3.1	bb
21	20241213_1_021	Solvent		1.000	3.29	238.528		238.528	0.0	-99.9	db
22	20241213_1_022	Solvent		1.000	3.26	371.020		371.020	0.0	-99.8	bd
23	20241213_1_023	301-Predose		1.000	3.31	173215.938		173215.938	1.0	-4.0	bb
24	20241213_1_024	301-2h		1.000	3.31	167079.500		167079.500	0.9	-7.4	bb
25	20241213_1_025	301-4h		1.000	3.30	179547.266		179547.266	1.0	-0.5	bb
26	20241213_1_026	301-8h		1.000	3.31	185515.109		185515.109	1.0	2.8	bb
27	20241213_1_027	301-12h		1.000	3.31	181237.422		181237.422	1.0	0.4	bb
28	20241213_1_028	301-24h		1.000	3.31	171527.063		171527.063	1.0	-4.9	bb
29	20241213_1_029	301-48h		1.000	3.31	164376.797		164376.797	0.9	-8.9	bb
30	20241213_1_030	301-72h		1.000	3.31	172990.563		172990.563	1.0	-4.1	bb
31	20241213_1_031	301-96h		1.000	3.31	174580.047		174580.047	1.0	-3.3	bb
32	20241213_1_032	301-168h		1.000	3.31	173876.813		173876.813	1.0	-3.6	bb
33	20241213_1_033	Solvent		1.000	3.30	252.404		252.404	0.0	-99.9	bd
34	20241213_1_034	Solvent		1.000							
35	20241213_1_035	302-Predose		1.000	3.31	169676.547		169676.547	0.9	-6.0	bb
36	20241213_1_036	302-2h		1.000	3.31	168015.891		168015.891	0.9	-6.9	bb
37	20241213_1_037	302-4h		1.000	3.31	173324.406		173324.406	1.0	-4.0	bb
38	20241213_1_038	302-8h		1.000	3.31	174418.766		174418.766	1.0	-3.3	bb
39	20241213_1_039	302-12h		1.000	3.31	180458.297		180458.297	1.0	0.0	bb
40	20241213_1_040	302-24h		1.000	3.31	173674.313		173674.313	1.0	-3.8	bb
41	20241213_1_041	302-48h		1.000	3.31	178341.938		178341.938	1.0	-1.2	bb
42	20241213_1_042	302-72h		1.000	3.31	174252.781		174252.781	1.0	-3.4	bb
43	20241213_1_043	302-96h		1.000	3.31	183150.172		183150.172	1.0	1.5	bb
44	20241213_1_044	302-168h		1.000	3.31	179493.016		179493.016	1.0	-0.5	bb
45	20241213_1_045	Solvent		1.000	3.27	207.711		207.711	0.0	-99.9	bd
46	20241213_1_046	Solvent		1.000	3.30	207.678		207.678	0.0	-99.9	bd
47	20241213_1_047	303-Predose		1.000	3.31	174508.656		174508.656	1.0	-3.3	bb
48	20241213_1_048	303-2h		1.000	3.31	170822.516		170822.516	0.9	-5.3	bb

Dataset: D:\Data\27013-24001-NG.PRO\20241214_WBPD081_044_SA-Tu.qld

Last Altered: Tuesday, July 15, 2025 15:34:14 China Standard Time
Printed: Tuesday, July 15, 2025 15:38:44 China Standard Time

Compound name: Tolbutamide (1)

	Name	ID	Type	Std. Conc	RT	Area	IS Area	Response	Conc.	%Dev	Primar...
49	20241213_1_049	303-4h		1.000	3.31	174797.438		174797.438	1.0	-3.1	bb
50	20241213_1_050	303-8h		1.000	3.31	181532.547		181532.547	1.0	0.6	bb
51	20241213_1_051	303-12h		1.000	3.31	179024.906		179024.906	1.0	-0.8	bb
52	20241213_1_052	303-24h		1.000	3.31	168124.250		168124.250	0.9	-6.8	bb
53	20241213_1_053	303-48h		1.000	3.31	178857.688		178857.688	1.0	-0.9	bb
54	20241213_1_054	303-72h		1.000	3.31	180031.281		180031.281	1.0	-0.2	bb
55	20241213_1_055	303-96h		1.000	3.31	176112.359		176112.359	1.0	-2.4	bb
56	20241213_1_056	303-168h		1.000	3.31	183235.672		183235.672	1.0	1.5	bb
57	20241213_1_057	Solvent		1.000	3.29	270.256		270.256	0.0	-99.9	bb
58	20241213_1_058	Solvent		1.000	3.27	320.126		320.126	0.0	-99.8	bb
59	20241213_1_059	B	Blank	1.000	3.28	1105.917		1105.917	0.0	-99.4	bb
60	20241213_1_060	O	Blank	1.000	3.31	187645.547		187645.547	1.0	4.0	bb
61	20241213_1_061	Q1	QC	1.000	3.31	186933.891		186933.891	1.0	3.6	bb
62	20241213_1_062	Q2	QC	1.000	3.31	188219.719		188219.719	1.0	4.3	bb
63	20241213_1_063	Q3	QC	1.000	3.31	182677.516		182677.516	1.0	1.2	bb
64	20241213_1_064	Q4	QC	1.000	3.31	181096.953		181096.953	1.0	0.4	bb
65	20241213_1_065	Solvent		1.000	3.30	321.493		321.493	0.0	-99.8	bd
66	20241213_1_066	Solvent		1.000	3.27	374.246		374.246	0.0	-99.8	bd

Dataset: D:\Data\27013-24001-NG.PRO\20241214_WBPD081_044_SA-Tu.qld

Last Altered: Tuesday, July 15, 2025 15:34:14 China Standard Time
Printed: Tuesday, July 15, 2025 15:38:44 China Standard Time

Compound name: Tolbutamide (1)

	Inj. Vol	Factor1	Vial
1	10.000	1.0	3:H,12
2	10.000	1.0	3:H,12
3	10.000	1.0	3:A,1
4	10.000	1.0	3:A,2
5	10.000	1.0	3:A,3
6	10.000	1.0	3:A,4
7	10.000	1.0	3:A,5
8	10.000	1.0	3:A,6
9	10.000	1.0	3:A,7
10	10.000	1.0	3:A,8
11	10.000	1.0	3:A,9
12	10.000	1.0	3:A,10
13	10.000	1.0	3:H,12
14	10.000	1.0	3:H,12
15	10.000	1.0	3:A,1
16	10.000	1.0	3:A,2
17	10.000	1.0	3:A,11
18	10.000	1.0	3:A,12
19	10.000	1.0	3:B,1
20	10.000	1.0	3:B,2
21	10.000	1.0	3:H,12
22	10.000	1.0	3:H,12
23	10.000	1.0	3:B,7
24	10.000	1.0	3:B,8
25	10.000	1.0	3:B,9
26	10.000	1.0	3:B,10
27	10.000	1.0	3:B,11
28	10.000	1.0	3:B,12
29	10.000	1.0	3:C,1
30	10.000	1.0	3:C,2
31	10.000	1.0	3:C,3
32	10.000	1.0	3:C,4
33	10.000	1.0	3:H,12
34	10.000	1.0	3:H,12
35	10.000	1.0	3:C,5
36	10.000	1.0	3:C,6
37	10.000	1.0	3:C,7
38	10.000	1.0	3:C,8
39	10.000	1.0	3:C,9
40	10.000	1.0	3:C,10
41	10.000	1.0	3:C,11
42	10.000	1.0	3:C,12
43	10.000	1.0	3:D,1
44	10.000	1.0	3:D,2
45	10.000	1.0	3:H,12
46	10.000	1.0	3:H,12
47	10.000	1.0	3:D,3
48	10.000	1.0	3:D,4
49	10.000	1.0	3:D,5
50	10.000	1.0	3:D,6
51	10.000	1.0	3:D,7

Dataset: D:\Data\27013-24001-NG.PRO\20241214_WBPD081_044_SA-Tu.qld

Last Altered: Tuesday, July 15, 2025 15:34:14 China Standard Time
Printed: Tuesday, July 15, 2025 15:38:44 China Standard Time

Compound name: Tolbutamide (1)

	Inj. Vol	Factor1	Vial
52	10.000	1.0	3:D,8
53	10.000	1.0	3:D,9
54	10.000	1.0	3:D,10
55	10.000	1.0	3:D,11
56	10.000	1.0	3:D,12
57	10.000	1.0	3:H,12
58	10.000	1.0	3:H,12
59	10.000	1.0	3:A,1
60	10.000	1.0	3:A,2
61	10.000	1.0	3:B,3
62	10.000	1.0	3:B,4
63	10.000	1.0	3:B,5
64	10.000	1.0	3:B,6
65	10.000	1.0	3:H,12
66	10.000	1.0	3:H,12

Dataset: D:\Data\27013-24001-NG.PRO\20241214_WBPD081_044_SA-Tu.qld

Last Altered: Tuesday, July 15, 2025 15:34:14 China Standard Time
Printed: Tuesday, July 15, 2025 15:38:44 China Standard Time

Method: D:\Data\27013-24001-NG.PRO\MethDB\20241214_WBPD081_044.mdb 14 Dec 2024 09:14:43
Calibration: 15 Jul 2025 15:34:14

Compound name: WBPD081_044 (2)
Correlation coefficient: r = 0.996092, r^2 = 0.992198
Calibration curve: 0.000257808 * x + 7.07839e-005
Response type: Internal Std (Ref 2), Area * (IS Conc. / IS Area)
Curve type: Linear, Origin: Exclude, Weighting: 1/x^2, Axis trans: None

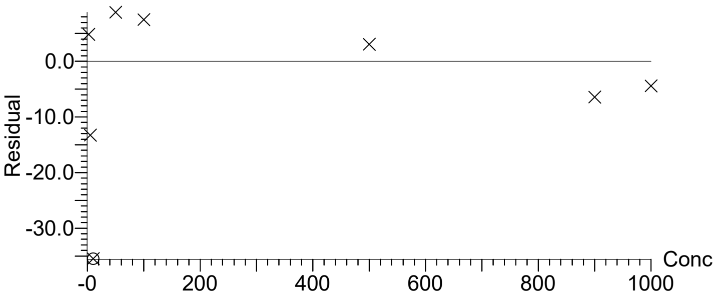

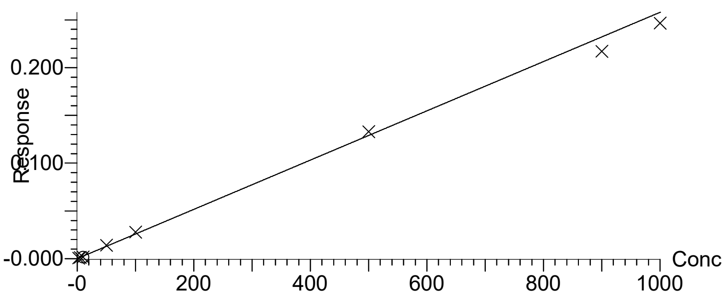

Compound name: Tolbutamide (1)
Response Factor: 180455
RRF SD: 7563.82, % Relative SD: 4.19153
Response type: External Std, Area
Curve type: RF

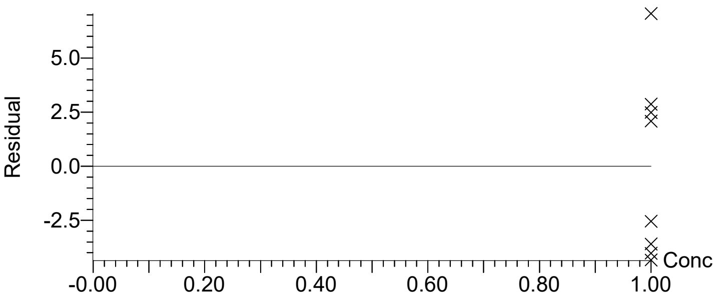

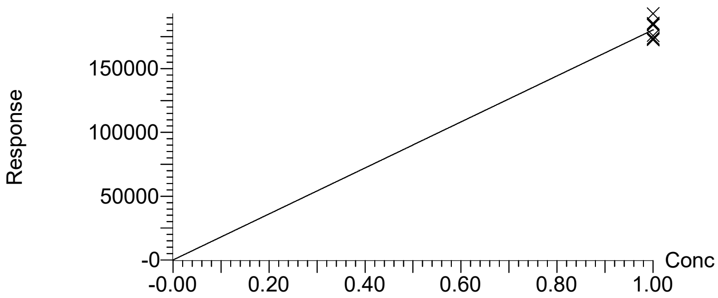

Dataset: D:\Data\27013-24001-NG.PRO\20241214_WBPD081_044_SA-Tu.qld

Last Altered: Tuesday, July 15, 2025 15:34:14 China Standard Time
Printed: Tuesday, July 15, 2025 15:38:44 China Standard Time

Method: D:\Data\27013-24001-NG.PRO\MethDB\20241214_WBPD081_044.mdb 14 Dec 2024 09:14:43
Calibration: 15 Jul 2025 15:34:14

Name: 20241213_1_001, ID: Solvent, Description:

WBPD081_044 (2)

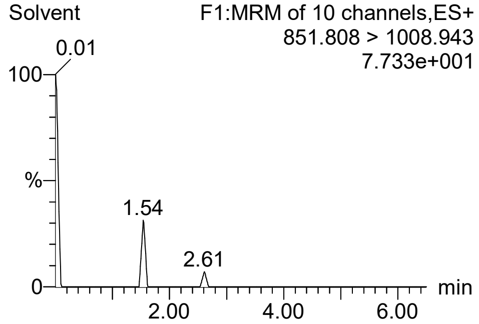

Tolbutamide (1)

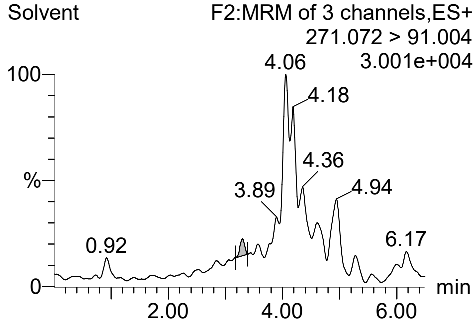

	# Name	Trace	RT	Area	IS Area	Response	Primar...	Conc.	%Dev
1	1 WBPD081_044 (2)	851.808 > 1008....			223.212				
2	2 Tolbutamide (1)	271.072 > 91.004	3.30	223.212		223.212	bb	0.0	-99.9

Name: 20241213_1_002, ID: Solvent, Description:

WBPD081_044 (2)

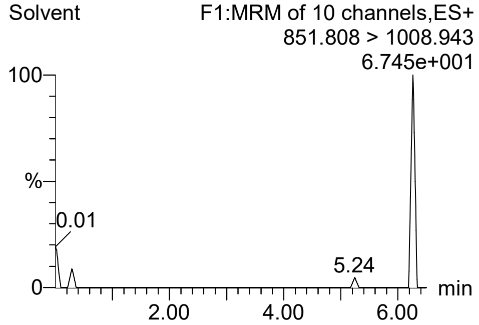

Tolbutamide (1)

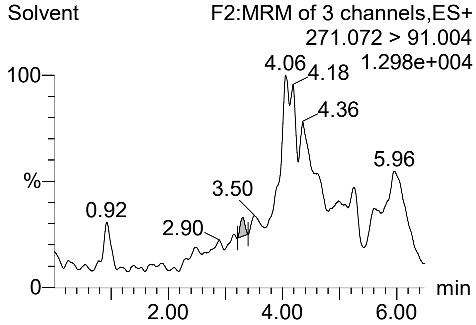

	# Name	Trace	RT	Area	IS Area	Response	Primar...	Conc.	%Dev
1	1 WBPD081_044 (2)	851.808 > 1008....			105.950				
2	2 Tolbutamide (1)	271.072 > 91.004	3.30	105.950		105.950	bb	0.0	-99.9

Name: 20241213_1_003, ID: B, Description:

WBPD081_044 (2)

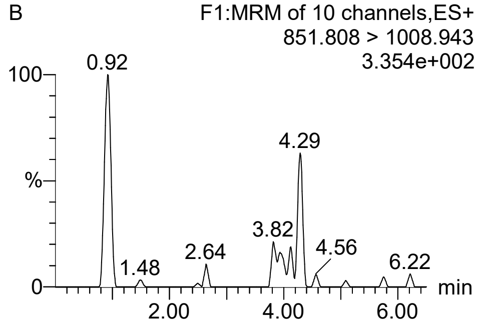

Tolbutamide (1)

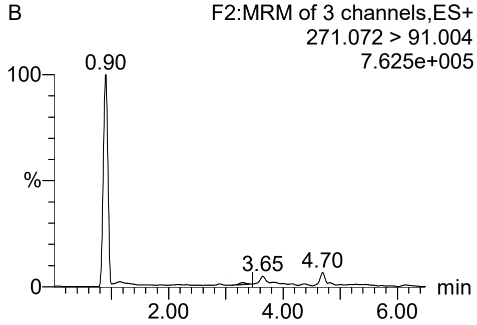

Dataset: D:\Data\27013-24001-NG.PRO\20241214_WBPD081_044_SA-Tu.qld

Last Altered: Tuesday, July 15, 2025 15:34:14 China Standard Time
Printed: Tuesday, July 15, 2025 15:38:44 China Standard Time

Name: 20241213_1_003, ID: B, Description:

	#	Name	Trace	RT	Area	IS Area	Response	Primar...	Conc.	%Dev
1	1	WBPD081_044 (2)	851.808 > 1008....			966.763				
2	2	Tolbutamide (1)	271.072 > 91.004	3.30	966.763		966.763	bb	0.0	-99.5

Name: 20241213_1_004, ID: O, Description:

WBPD081_044 (2)

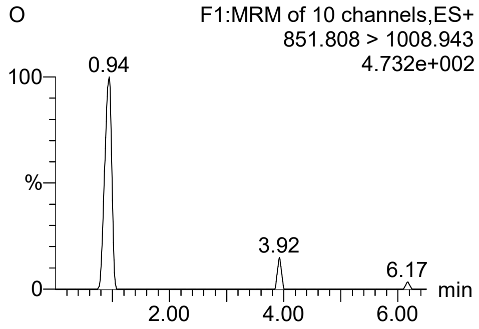

Tolbutamide (1)

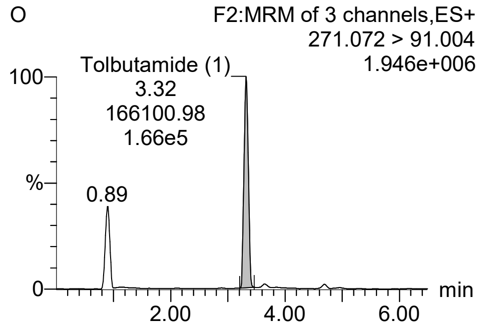

	#	Name	Trace	RT	Area	IS Area	Response	Primar...	Conc.	%Dev
1	1	WBPD081_044 (2)	851.808 > 1008....			166100.984				
2	2	Tolbutamide (1)	271.072 > 91.004	3.32	166100.984		166100.984	bb	0.9	-8.0

Name: 20241213_1_005, ID: STD1, Description: WBPD081_044

WBPD081_044 (2)

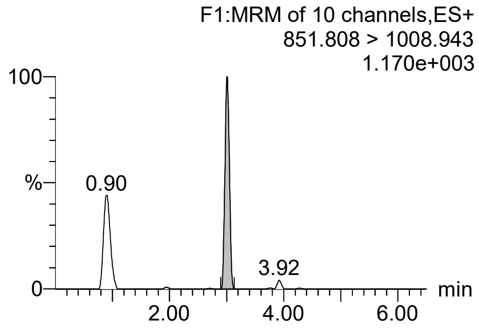

Tolbutamide (1)

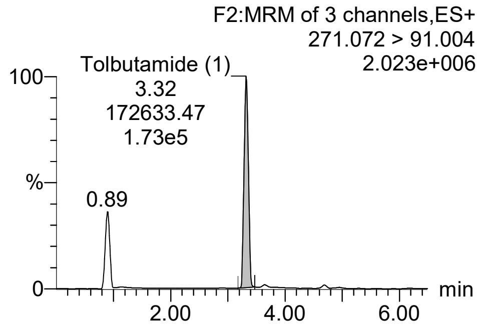

	#	Name	Trace	RT	Area	IS Area	Response	Primar...	Conc.	%Dev
1	1	WBPD081_044 (2)	851.808 > 1008....	3.00	105.531	172633.469	0.001	bb	2.1	4.8
2	2	Tolbutamide (1)	271.072 > 91.004	3.32	172633.469		172633.469	bb	1.0	-4.3

Name: 20241213_1_006, ID: STD2, Description: WBPD081_044

Dataset: D:\Data\27013-24001-NG.PRO\20241214_WBPD081_044_SA-Tu.qld

Last Altered: Tuesday, July 15, 2025 15:34:14 China Standard Time
Printed: Tuesday, July 15, 2025 15:38:44 China Standard Time

Name: 20241213_1_006, ID: STD2, Description: WBPD081_044

WBPD081_044 (2)

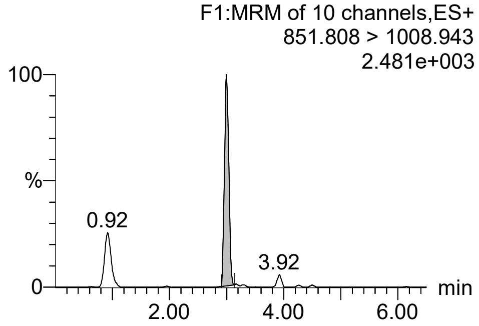

Tolbutamide (1)

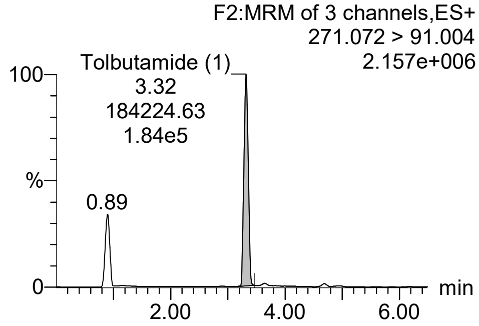

	#	Name	Trace	RT	Area	IS Area	Response	Primar...	Conc.	%Dev
1	1	WBPD081_044 (2)	851.808 > 1008....	3.00	218.925	184224.625	0.001	bb	4.3	-13.3
2	2	Tolbutamide (1)	271.072 > 91.004	3.32	184224.625		184224.625	bb	1.0	2.1

Name: 20241213_1_007, ID: STD3, Description: WBPD081_044

WBPD081_044 (2)

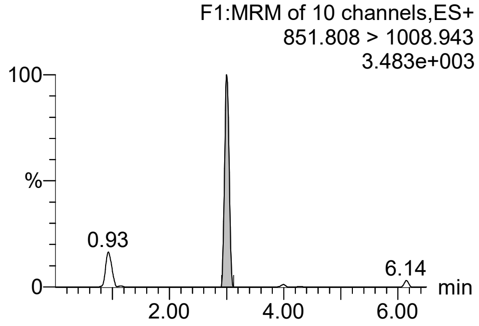

Tolbutamide (1)

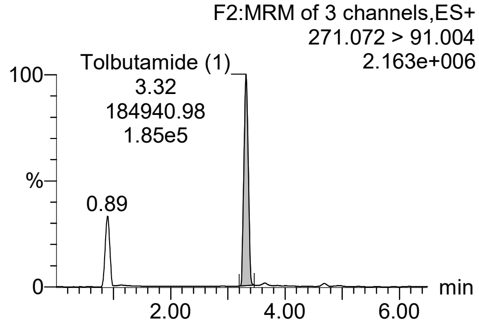

	#	Name	Trace	RT	Area	IS Area	Response	Primar...	Conc.	%Dev
1	1	WBPD081_044 (2)	851.808 > 1008....	3.00	320.699	184940.984	0.002	bbX	6.5	-35.5
2	2	Tolbutamide (1)	271.072 > 91.004	3.32	184940.984		184940.984	bb	1.0	2.5

Name: 20241213_1_008, ID: STD4, Description: WBPD081_044

WBPD081_044 (2)

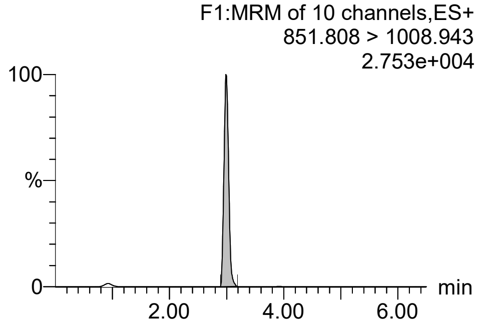

Tolbutamide (1)

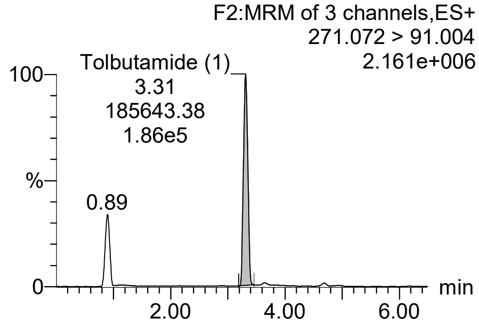

	#	Name	Trace	RT	Area	IS Area	Response	Primar...	Conc.	%Dev
1	1	WBPD081_044 (2)	851.808 > 1008....	2.99	2617.288	185643.375	0.014	bb	54.4	8.8
2	2	Tolbutamide (1)	271.072 > 91.004	3.31	185643.375		185643.375	bb	1.0	2.9

Dataset: D:\Data\27013-24001-NG.PRO\20241214_WBPD081_044_SA-Tu.qld

Last Altered: Tuesday, July 15, 2025 15:34:14 China Standard Time
Printed: Tuesday, July 15, 2025 15:38:44 China Standard Time

Name: 20241213_1_009, ID: STD5, Description: WBPD081_044

WBPD081_044 (2)

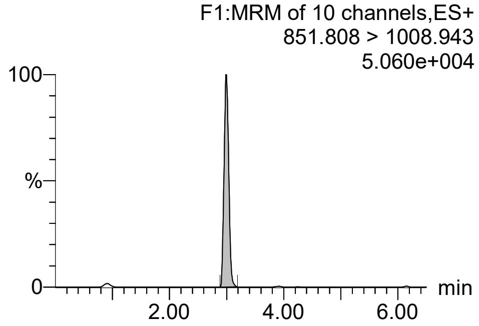

Tolbutamide (1)

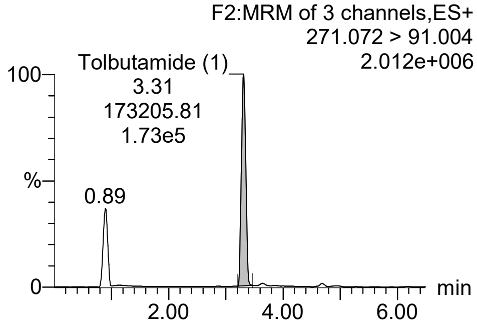

	# Name	Trace	RT	Area	IS Area	Response	Primar...	Conc.	%Dev
1	1 WBPD081_044 (2)	851.808 > 1008....	2.99	4811.378	173205.813	0.028	bb	107.5	7.5
2	2 Tolbutamide (1)	271.072 > 91.004	3.31	173205.813		173205.813	bb	1.0	-4.0

Name: 20241213_1_010, ID: STD6, Description: WBPD081_044

WBPD081_044 (2)

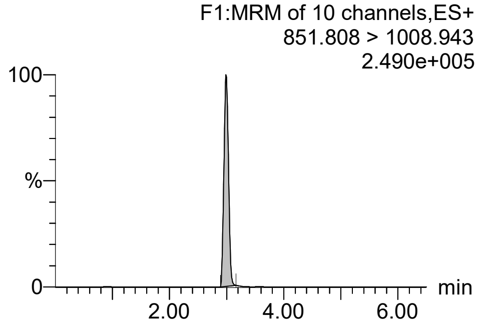

Tolbutamide (1)

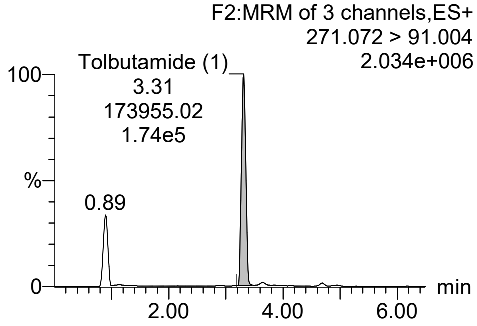

	# Name	Trace	RT	Area	IS Area	Response	Primar...	Conc.	%Dev
1	1 WBPD081_044 (2)	851.808 > 1008....	2.99	23121.529	173955.016	0.133	bb	515.3	3.1
2	2 Tolbutamide (1)	271.072 > 91.004	3.31	173955.016		173955.016	bb	1.0	-3.6

Name: 20241213_1_011, ID: STD7, Description: WBPD081_044

WBPD081_044 (2)

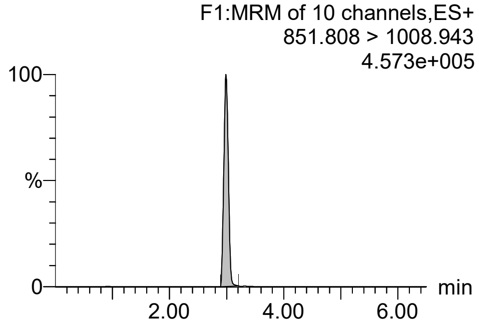

Tolbutamide (1)

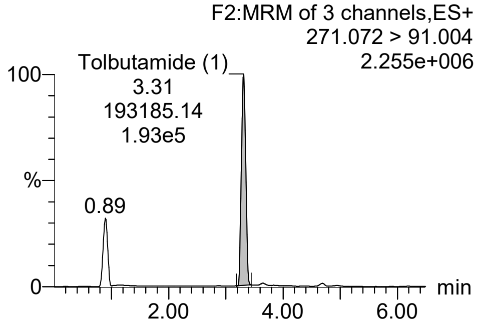

	# Name	Trace	RT	Area	IS Area	Response	Primar...	Conc.	%Dev
1	1 WBPD081_044 (2)	851.808 > 1008....	2.99	41948.273	193185.141	0.217	bb	842.0	-6.4
2	2 Tolbutamide (1)	271.072 > 91.004	3.31	193185.141		193185.141	bb	1.1	7.1

Dataset: D:\Data\27013-24001-NG.PRO\20241214_WBPD081_044_SA-Tu.qld

Last Altered: Tuesday, July 15, 2025 15:34:14 China Standard Time
Printed: Tuesday, July 15, 2025 15:38:44 China Standard Time

Name: 20241213_1_012, ID: STD8, Description: WBPD081_044

WBPD081_044 (2)

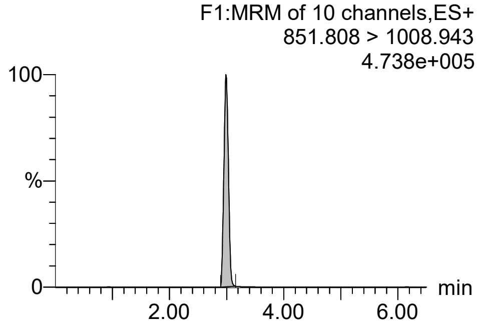

Tolbutamide (1)

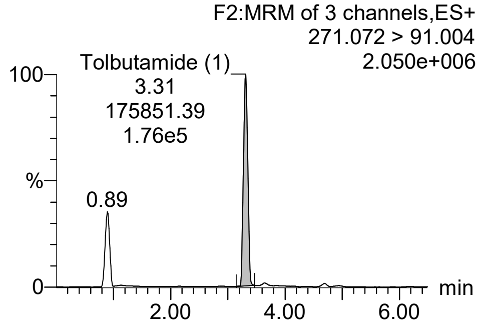

	#	Name	Trace	RT	Area	IS Area	Response	Primar...	Conc.	%Dev
1	1	WBPD081_044 (2)	851.808 > 1008....	2.99	43337.594	175851.391	0.246	bb	955.6	-4.4
2	2	Tolbutamide (1)	271.072 > 91.004	3.31	175851.391		175851.391	bb	1.0	-2.6

Name: 20241213_1_013, ID: Solvent, Description:

WBPD081_044 (2)

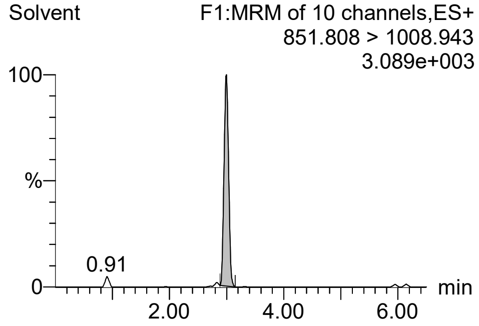

Tolbutamide (1)

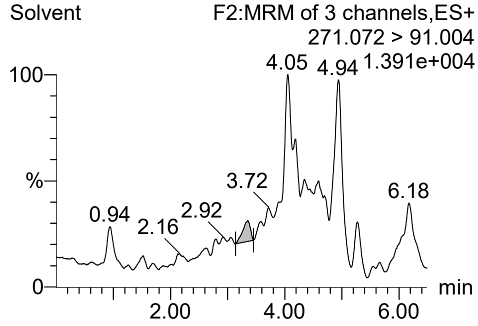

	#	Name	Trace	RT	Area	IS Area	Response	Primar...	Conc.	%Dev
1	1	WBPD081_044 (2)	851.808 > 1008....	3.00	285.179	207.174	1.377	bb	5339.0	
2	2	Tolbutamide (1)	271.072 > 91.004	3.35	207.174		207.174	bb	0.0	-99.9

Name: 20241213_1_014, ID: Solvent, Description:

WBPD081_044 (2)

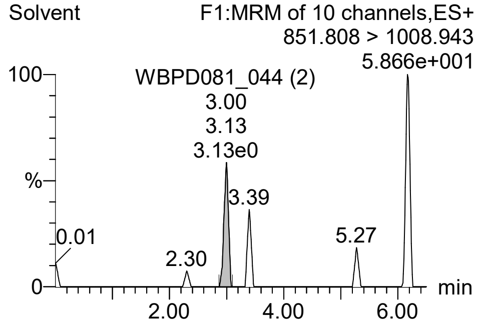

Tolbutamide (1)

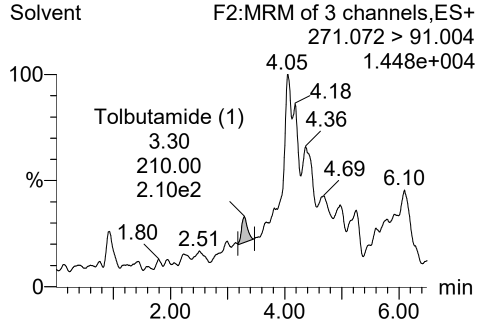

	#	Name	Trace	RT	Area	IS Area	Response	Primar...	Conc.	%Dev
1	1	WBPD081_044 (2)	851.808 > 1008....	3.00	3.125	209.998	0.015	bb	57.4	
2	2	Tolbutamide (1)	271.072 > 91.004	3.30	209.998		209.998	db	0.0	-99.9

Dataset: D:\Data\27013-24001-NG.PRO\20241214_WBPD081_044_SA-Tu.qld

Last Altered: Tuesday, July 15, 2025 15:34:14 China Standard Time
Printed: Tuesday, July 15, 2025 15:38:44 China Standard Time

Name: 20241213_1_015, ID: B, Description:

WBPD081_044 (2)

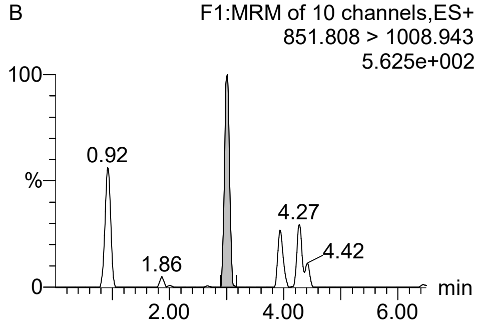

Tolbutamide (1)

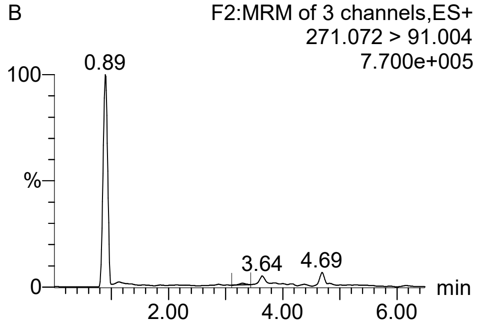

	#	Name	Trace	RT	Area	IS Area	Response	Primar...	Conc.	%Dev
1	1	WBPD081_044 (2)	851.808 > 1008....	3.01	57.816	686.876	0.084	bb	326.2	
2	2	Tolbutamide (1)	271.072 > 91.004	3.30	686.876		686.876	bb	0.0	-99.6

Name: 20241213_1_016, ID: O, Description:

WBPD081_044 (2)

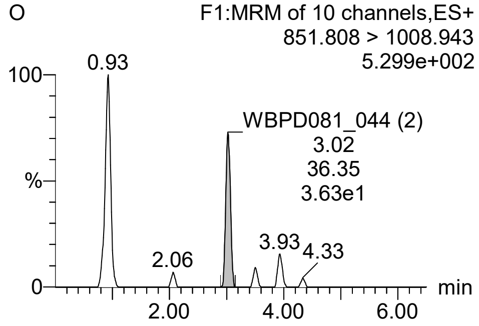

Tolbutamide (1)

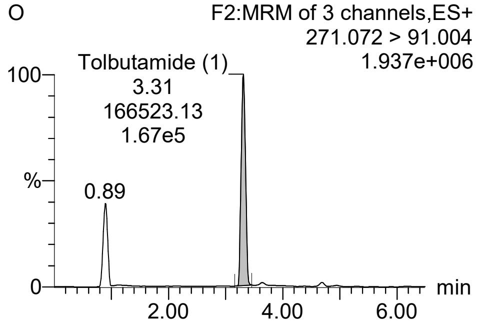

	#	Name	Trace	RT	Area	IS Area	Response	Primar...	Conc.	%Dev
1	1	WBPD081_044 (2)	851.808 > 1008....	3.02	36.346	166523.125	0.000	bb	0.6	
2	2	Tolbutamide (1)	271.072 > 91.004	3.31	166523.125		166523.125	bb	0.9	-7.7

Name: 20241213_1_017, ID: Q1, Description:

WBPD081_044 (2)

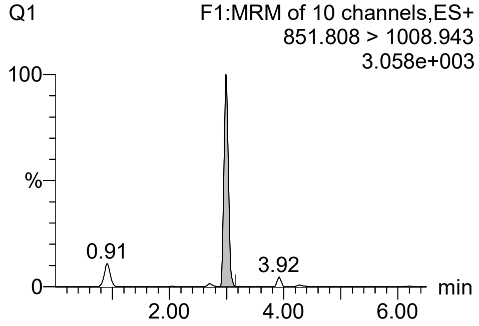

Tolbutamide (1)

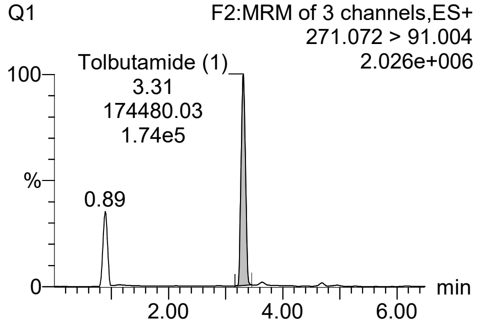

	#	Name	Trace	RT	Area	IS Area	Response	Primar...	Conc.	%Dev
1	1	WBPD081_044 (2)	851.808 > 1008....	2.99	275.202	174480.031	0.002	bb	5.8	-2.6
2	2	Tolbutamide (1)	271.072 > 91.004	3.31	174480.031		174480.031	bb	1.0	-3.3

Dataset: D:\Data\27013-24001-NG.PRO\20241214_WBPD081_044_SA-Tu.qld

Last Altered: Tuesday, July 15, 2025 15:34:14 China Standard Time
Printed: Tuesday, July 15, 2025 15:38:44 China Standard Time

Name: 20241213_1_018, ID: Q2, Description:

WBPD081_044 (2)

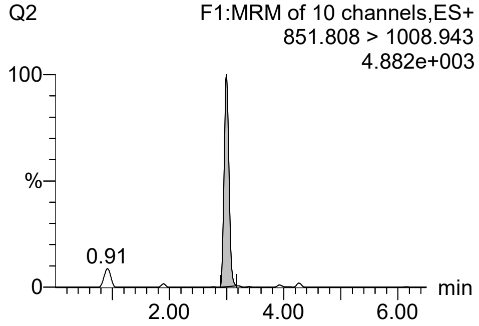

Tolbutamide (1)

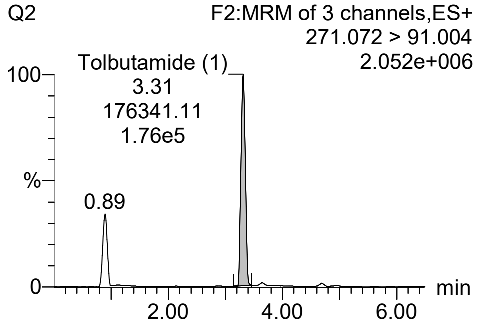

	#	Name	Trace	RT	Area	IS Area	Response	Primar...	Conc.	%Dev
1	1	WBPD081_044 (2)	851.808 > 1008....	3.00	478.111	176341.109	0.003	bb	10.2	-14.6
2	2	Tolbutamide (1)	271.072 > 91.004	3.31	176341.109		176341.109	bb	1.0	-2.3

Name: 20241213_1_019, ID: Q3, Description:

WBPD081_044 (2)

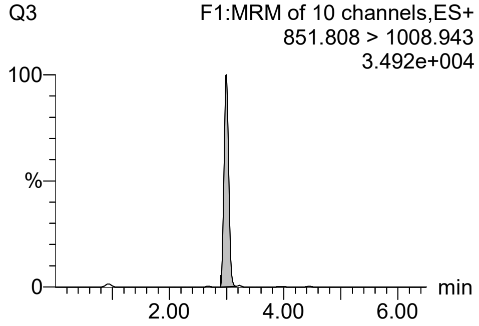

Tolbutamide (1)

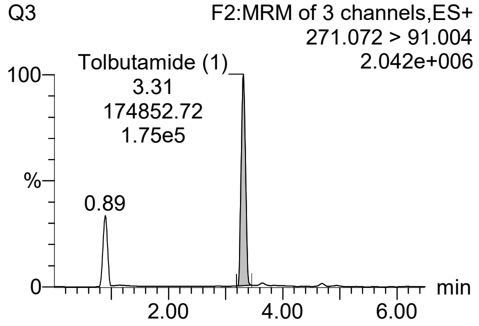

	#	Name	Trace	RT	Area	IS Area	Response	Primar...	Conc.	%Dev
1	1	WBPD081_044 (2)	851.808 > 1008....	3.00	3304.283	174852.719	0.019	bb	73.0	-8.7
2	2	Tolbutamide (1)	271.072 > 91.004	3.31	174852.719		174852.719	bb	1.0	-3.1

Name: 20241213_1_020, ID: Q4, Description:

WBPD081_044 (2)

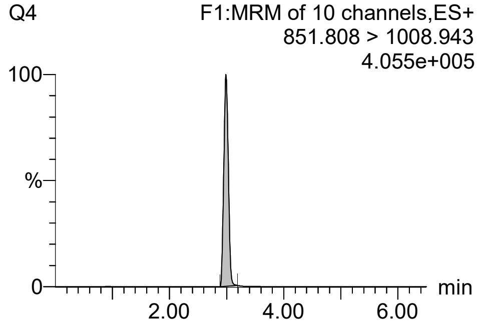

Tolbutamide (1)

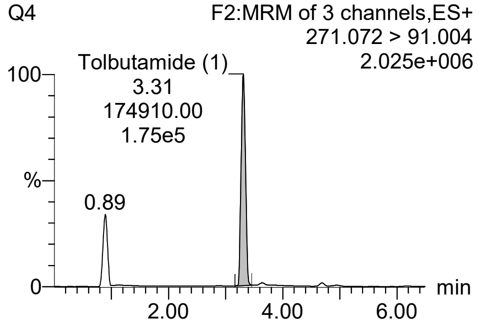

	#	Name	Trace	RT	Area	IS Area	Response	Primar...	Conc.	%Dev
1	1	WBPD081_044 (2)	851.808 > 1008....	2.99	37337.980	174910.000	0.213	bb	827.7	3.5
2	2	Tolbutamide (1)	271.072 > 91.004	3.31	174910.000		174910.000	bb	1.0	-3.1

Dataset: D:\Data\27013-24001-NG.PRO\20241214_WBPD081_044_SA-Tu.qld

Last Altered: Tuesday, July 15, 2025 15:34:14 China Standard Time
Printed: Tuesday, July 15, 2025 15:38:44 China Standard Time

Name: 20241213_1_021, ID: Solvent, Description:

WBPD081_044 (2)

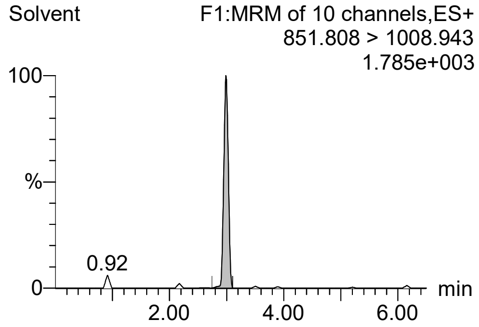

Tolbutamide (1)

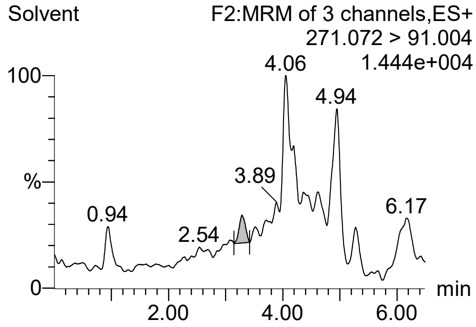

	#	Name	Trace	RT	Area	IS Area	Response	Primar...	Conc.	%Dev
1	1	WBPD081_044 (2)	851.808 > 1008....	2.99	164.682	238.528	0.690	bb	2677.7	
2	2	Tolbutamide (1)	271.072 > 91.004	3.29	238.528		238.528	db	0.0	-99.9

Name: 20241213_1_022, ID: Solvent, Description:

WBPD081_044 (2)

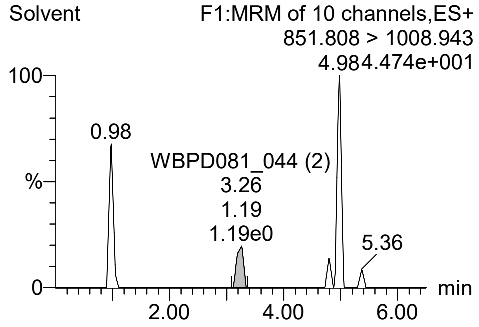

Tolbutamide (1)

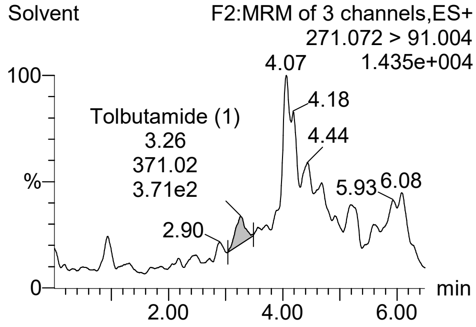

	#	Name	Trace	RT	Area	IS Area	Response	Primar...	Conc.	%Dev
1	1	WBPD081_044 (2)	851.808 > 1008....	3.26	1.192	371.020	0.003	bb	12.2	
2	2	Tolbutamide (1)	271.072 > 91.004	3.26	371.020		371.020	bd	0.0	-99.8

Name: 20241213_1_023, ID: 301-Predose, Description:

WBPD081_044 (2)

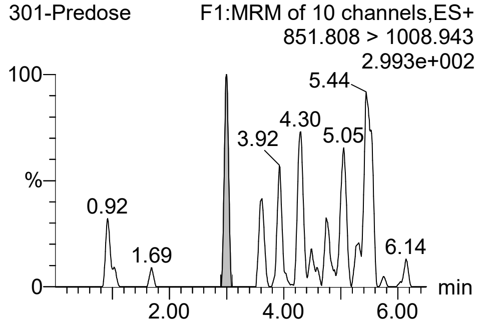

Tolbutamide (1)

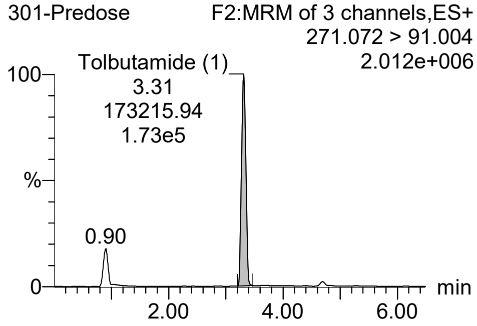

	#	Name	Trace	RT	Area	IS Area	Response	Primar...	Conc.	%Dev
1	1	WBPD081_044 (2)	851.808 > 1008....	3.00	26.116	173215.938	0.000	bb	0.3	
2	2	Tolbutamide (1)	271.072 > 91.004	3.31	173215.938		173215.938	bb	1.0	-4.0

Dataset: D:\Data\27013-24001-NG.PRO\20241214_WBPD081_044_SA-Tu.qld

Last Altered: Tuesday, July 15, 2025 15:34:14 China Standard Time
Printed: Tuesday, July 15, 2025 15:38:44 China Standard Time

Name: 20241213_1_024, ID: 301-2h, Description:

WBPD081_044 (2)

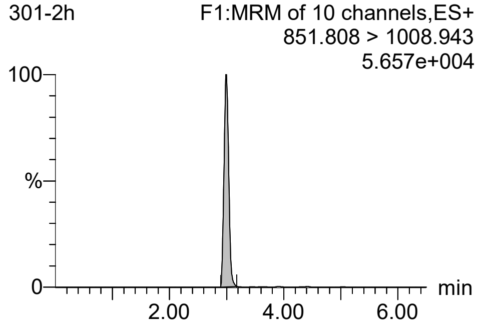

Tolbutamide (1)

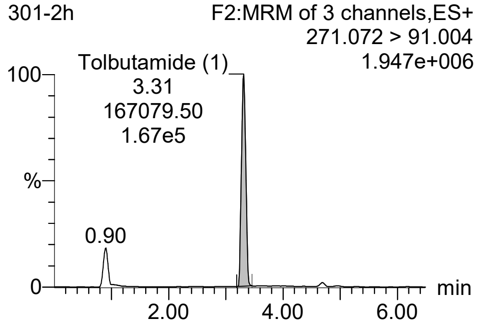

	#	Name	Trace	RT	Area	IS Area	Response	Primar...	Conc.	%Dev
1	1	WBPD081_044 (2)	851.808 > 1008....	3.00	5388.515	167079.500	0.032	bb	124.8	
2	2	Tolbutamide (1)	271.072 > 91.004	3.31	167079.500		167079.500	bb	0.9	-7.4

Name: 20241213_1_025, ID: 301-4h, Description:

WBPD081_044 (2)

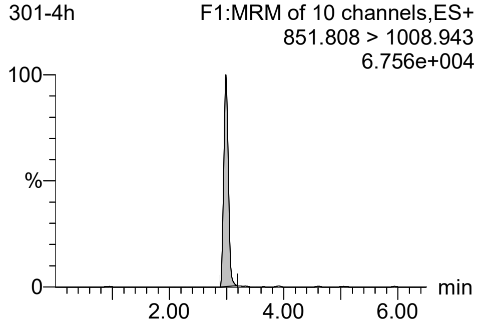

Tolbutamide (1)

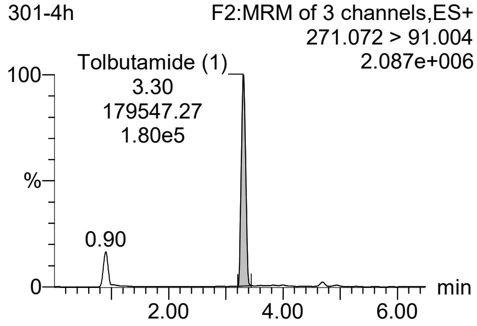

	#	Name	Trace	RT	Area	IS Area	Response	Primar...	Conc.	%Dev
1	1	WBPD081_044 (2)	851.808 > 1008....	2.99	6280.532	179547.266	0.035	bb	135.4	
2	2	Tolbutamide (1)	271.072 > 91.004	3.30	179547.266		179547.266	bb	1.0	-0.5

Name: 20241213_1_026, ID: 301-8h, Description:

WBPD081_044 (2)

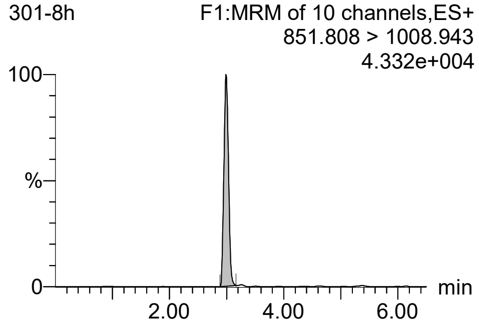

Tolbutamide (1)

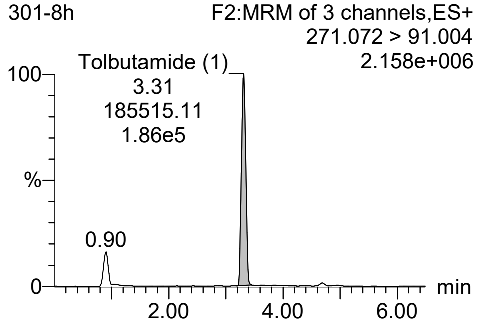

	#	Name	Trace	RT	Area	IS Area	Response	Primar...	Conc.	%Dev
1	1	WBPD081_044 (2)	851.808 > 1008....	2.99	4037.921	185515.109	0.022	bb	84.2	
2	2	Tolbutamide (1)	271.072 > 91.004	3.31	185515.109		185515.109	bb	1.0	2.8

Dataset: D:\Data\27013-24001-NG.PRO\20241214_WBPD081_044_SA-Tu.qld

Last Altered: Tuesday, July 15, 2025 15:34:14 China Standard Time
Printed: Tuesday, July 15, 2025 15:38:44 China Standard Time

Name: 20241213_1_027, ID: 301-12h, Description:

WBPD081_044 (2)

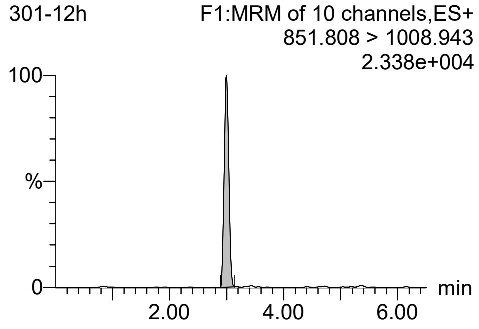

Tolbutamide (1)

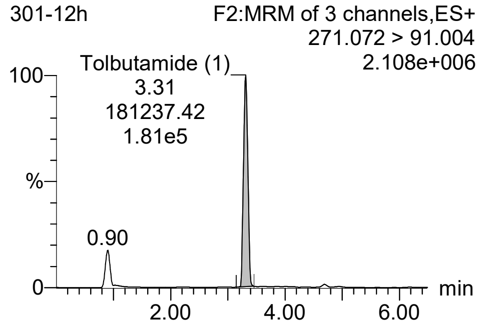

	#	Name	Trace	RT	Area	IS Area	Response	Primar...	Conc.	%Dev
1	1	WBPD081_044 (2)	851.808 > 1008....	3.00	2196.241	181237.422	0.012	bb	46.7	
2	2	Tolbutamide (1)	271.072 > 91.004	3.31	181237.422		181237.422	bb	1.0	0.4

Name: 20241213_1_028, ID: 301-24h, Description:

WBPD081_044 (2)

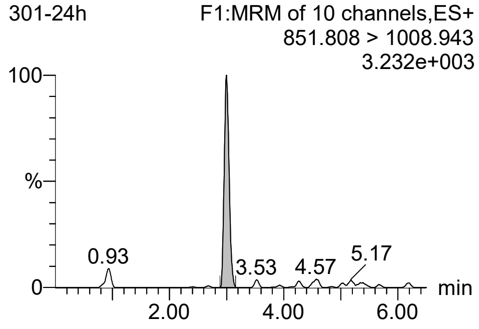

Tolbutamide (1)

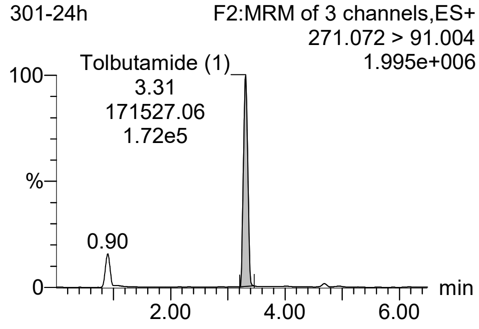

	#	Name	Trace	RT	Area	IS Area	Response	Primar...	Conc.	%Dev
1	1	WBPD081_044 (2)	851.808 > 1008....	3.00	319.508	171527.063	0.002	bb	7.0	
2	2	Tolbutamide (1)	271.072 > 91.004	3.31	171527.063		171527.063	bb	1.0	-4.9

Name: 20241213_1_029, ID: 301-48h, Description:

WBPD081_044 (2)

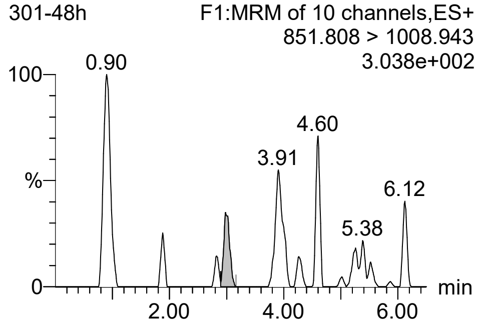

Tolbutamide (1)

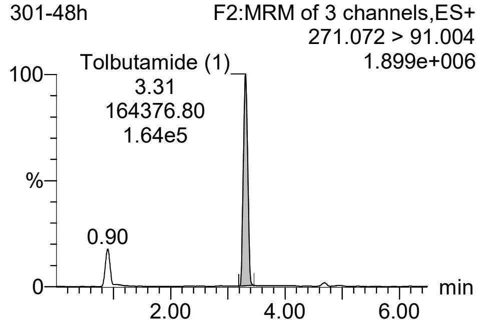

	#	Name	Trace	RT	Area	IS Area	Response	Primar...	Conc.	%Dev
1	1	WBPD081_044 (2)	851.808 > 1008....	2.98	12.961	164376.797	0.000	db	0.0	
2	2	Tolbutamide (1)	271.072 > 91.004	3.31	164376.797		164376.797	bb	0.9	-8.9

Dataset: D:\Data\27013-24001-NG.PRO\20241214_WBPD081_044_SA-Tu.qld

Last Altered: Tuesday, July 15, 2025 15:34:14 China Standard Time
Printed: Tuesday, July 15, 2025 15:38:44 China Standard Time

Name: 20241213_1_030, ID: 301-72h, Description:

WBPD081_044 (2)

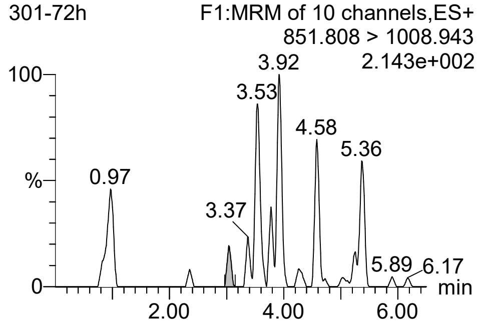

Tolbutamide (1)

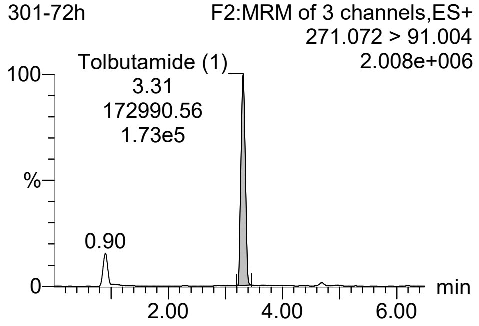

	#	Name	Trace	RT	Area	IS Area	Response	Primar...	Conc.	%Dev
1	1	WBPD081_044 (2)	851.808 > 1008....	3.04	3.368	172990.563	0.000	bbl		
2	2	Tolbutamide (1)	271.072 > 91.004	3.31	172990.563		172990.563	bb	1.0	-4.1

Name: 20241213_1_031, ID: 301-96h, Description:

WBPD081_044 (2)

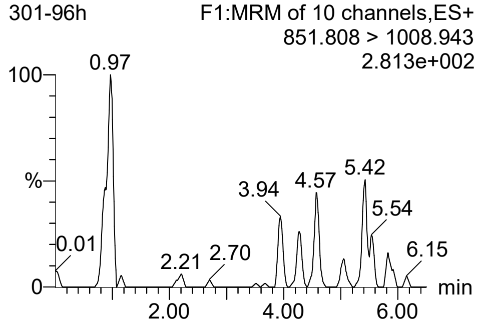

Tolbutamide (1)

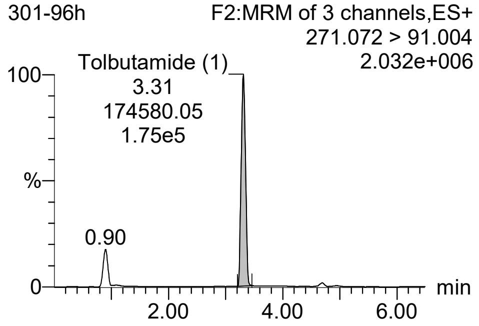

	#	Name	Trace	RT	Area	IS Area	Response	Primar...	Conc.	%Dev
1	1	WBPD081_044 (2)	851.808 > 1008....			174580.047				
2	2	Tolbutamide (1)	271.072 > 91.004	3.31	174580.047		174580.047	bb	1.0	-3.3

Name: 20241213_1_032, ID: 301-168h, Description:

WBPD081_044 (2)

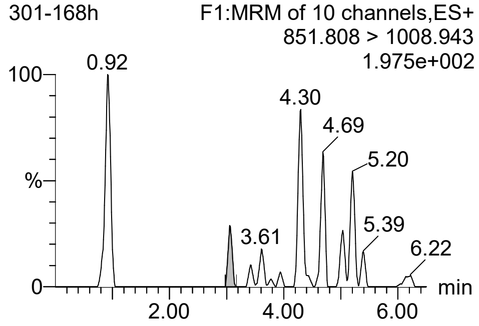

Tolbutamide (1)

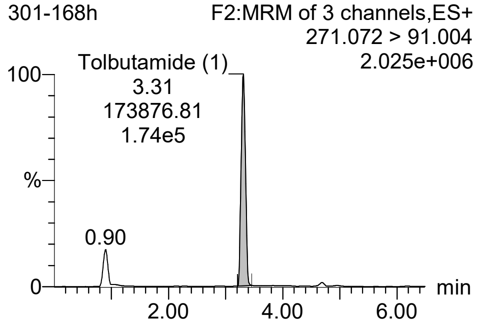

	#	Name	Trace	RT	Area	IS Area	Response	Primar...	Conc.	%Dev
1	1	WBPD081_044 (2)	851.808 > 1008....	3.05	4.666	173876.813	0.000	bbl		
2	2	Tolbutamide (1)	271.072 > 91.004	3.31	173876.813		173876.813	bb	1.0	-3.6

Dataset: D:\Data\27013-24001-NG.PRO\20241214_WBPD081_044_SA-Tu.qld

Last Altered: Tuesday, July 15, 2025 15:34:14 China Standard Time
Printed: Tuesday, July 15, 2025 15:38:44 China Standard Time

Name: 20241213_1_033, ID: Solvent, Description:

WBPD081_044 (2)

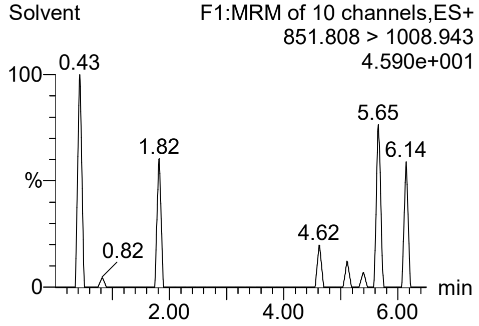

Tolbutamide (1)

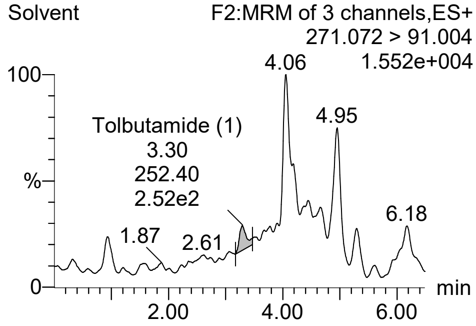

	#	Name	Trace	RT	Area	IS Area	Response	Primar...	Conc.	%Dev
1	1	WBPD081_044 (2)	851.808 > 1008....			252.404				
2	2	Tolbutamide (1)	271.072 > 91.004	3.30	252.404		252.404	bd	0.0	-99.9

Name: 20241213_1_034, ID: Solvent, Description:

WBPD081_044 (2)

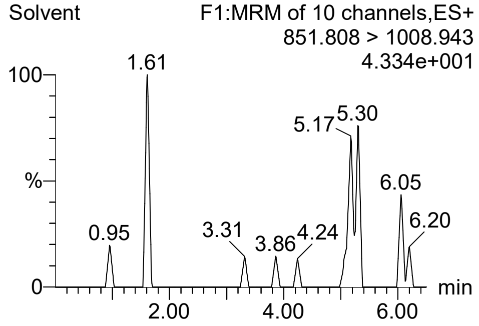

Tolbutamide (1)

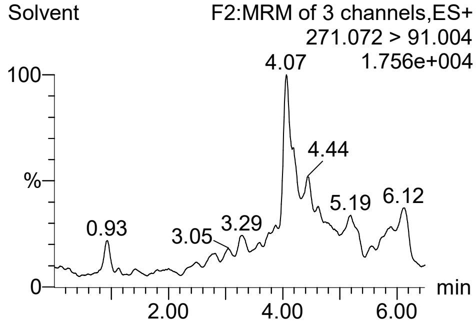

	#	Name	Trace	RT	Area	IS Area	Response	Primar...	Conc.	%Dev
1	1	WBPD081_044 (2)	851.808 > 1008....							
2	2	Tolbutamide (1)	271.072 > 91.004							

Name: 20241213_1_035, ID: 302-Predose, Description:

WBPD081_044 (2)

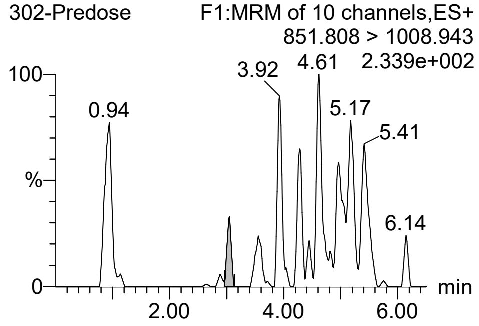

Tolbutamide (1)

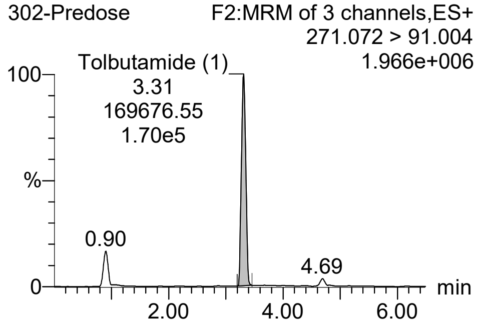

	#	Name	Trace	RT	Area	IS Area	Response	Primar...	Conc.	%Dev
1	1	WBPD081_044 (2)	851.808 > 1008....	3.05	6.513	169676.547	0.000	dbl		
2	2	Tolbutamide (1)	271.072 > 91.004	3.31	169676.547		169676.547	bb	0.9	-6.0

Dataset: D:\Data\27013-24001-NG.PRO\20241214_WBPD081_044_SA-Tu.qld

Last Altered: Tuesday, July 15, 2025 15:34:14 China Standard Time
Printed: Tuesday, July 15, 2025 15:38:44 China Standard Time

Name: 20241213_1_036, ID: 302-2h, Description:

WBPD081_044 (2)

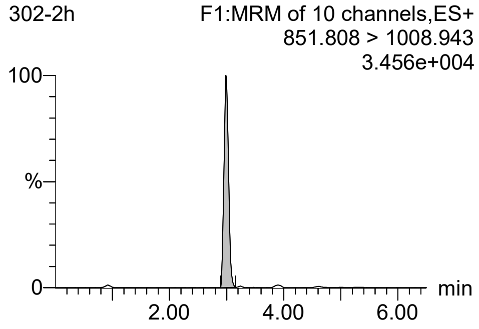

Tolbutamide (1)

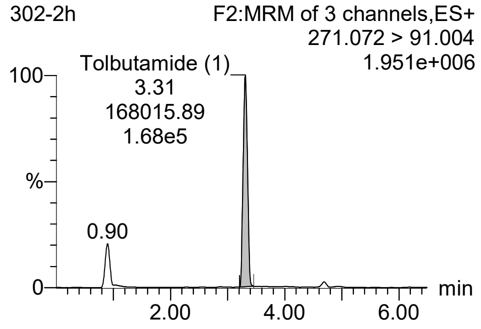

	# Name	Trace	RT	Area	IS Area	Response	Primar...	Conc.	%Dev
1	1 WBPD081_044 (2)	851.808 > 1008....	2.99	3231.922	168015.891	0.019	bb	74.3	
2	2 Tolbutamide (1)	271.072 > 91.004	3.31	168015.891		168015.891	bb	0.9	-6.9

Name: 20241213_1_037, ID: 302-4h, Description:

WBPD081_044 (2)

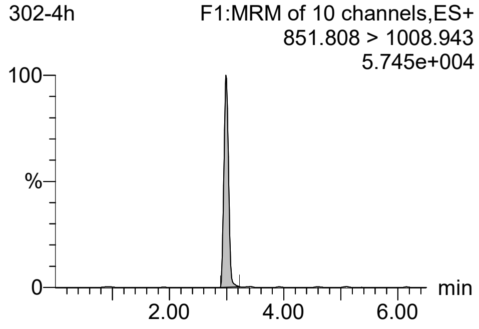

Tolbutamide (1)

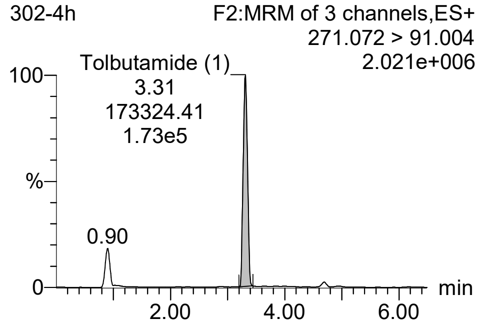

	# Name	Trace	RT	Area	IS Area	Response	Primar...	Conc.	%Dev
1	1 WBPD081_044 (2)	851.808 > 1008....	2.99	5348.600	173324.406	0.031	bb	119.4	
2	2 Tolbutamide (1)	271.072 > 91.004	3.31	173324.406		173324.406	bb	1.0	-4.0

Name: 20241213_1_038, ID: 302-8h, Description:

WBPD081_044 (2)

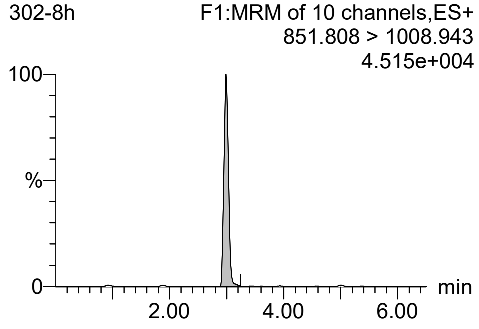

Tolbutamide (1)

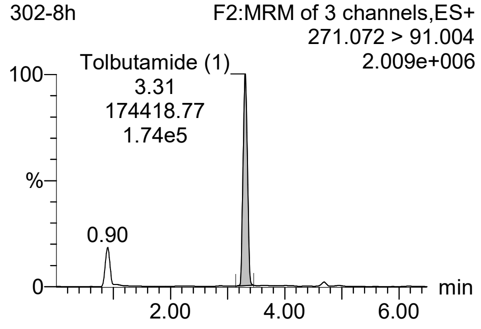

	# Name	Trace	RT	Area	IS Area	Response	Primar...	Conc.	%Dev
1	1 WBPD081_044 (2)	851.808 > 1008....	2.99	4186.138	174418.766	0.024	bb	92.8	
2	2 Tolbutamide (1)	271.072 > 91.004	3.31	174418.766		174418.766	bb	1.0	-3.3

Dataset: D:\Data\27013-24001-NG.PRO\20241214_WBPD081_044_SA-Tu.qld

Last Altered: Tuesday, July 15, 2025 15:34:14 China Standard Time
Printed: Tuesday, July 15, 2025 15:38:44 China Standard Time

Name: 20241213_1_039, ID: 302-12h, Description:

WBPD081_044 (2)

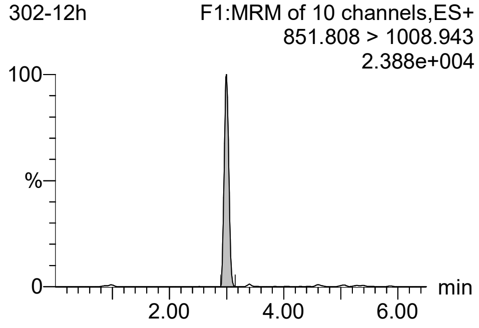

Tolbutamide (1)

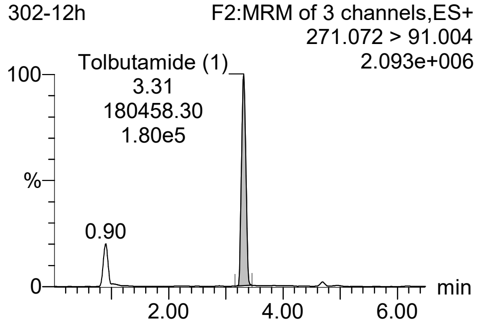

	#	Name	Trace	RT	Area	IS Area	Response	Primar...	Conc.	%Dev
1	1	WBPD081_044 (2)	851.808 > 1008....	3.00	2234.172	180458.297	0.012	bb	47.7	
2	2	Tolbutamide (1)	271.072 > 91.004	3.31	180458.297		180458.297	bb	1.0	0.0

Name: 20241213_1_040, ID: 302-24h, Description:

WBPD081_044 (2)

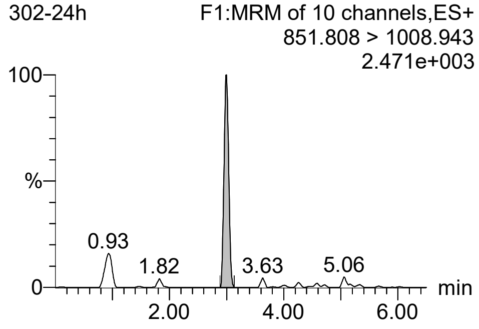

Tolbutamide (1)

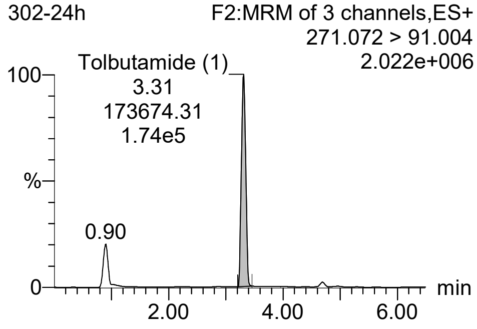

	#	Name	Trace	RT	Area	IS Area	Response	Primar...	Conc.	%Dev
1	1	WBPD081_044 (2)	851.808 > 1008....	3.00	234.392	173674.313	0.001	bb	5.0	
2	2	Tolbutamide (1)	271.072 > 91.004	3.31	173674.313		173674.313	bb	1.0	-3.8

Name: 20241213_1_041, ID: 302-48h, Description:

WBPD081_044 (2)

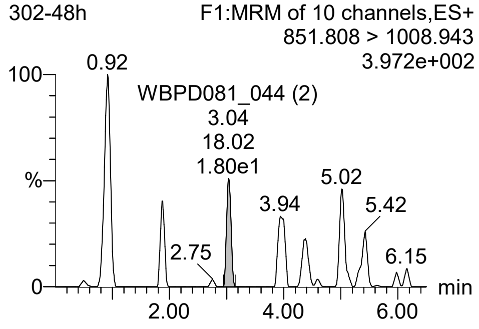

Tolbutamide (1)

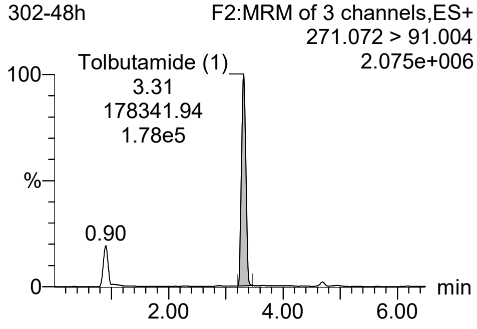

	#	Name	Trace	RT	Area	IS Area	Response	Primar...	Conc.	%Dev
1	1	WBPD081_044 (2)	851.808 > 1008....	3.04	18.022	178341.938	0.000	bb	0.1	
2	2	Tolbutamide (1)	271.072 > 91.004	3.31	178341.938		178341.938	bb	1.0	-1.2

Dataset: D:\Data\27013-24001-NG.PRO\20241214_WBPD081_044_SA-Tu.qld

Last Altered: Tuesday, July 15, 2025 15:34:14 China Standard Time
Printed: Tuesday, July 15, 2025 15:38:44 China Standard Time

Name: 20241213_1_042, ID: 302-72h, Description:

WBPD081_044 (2)

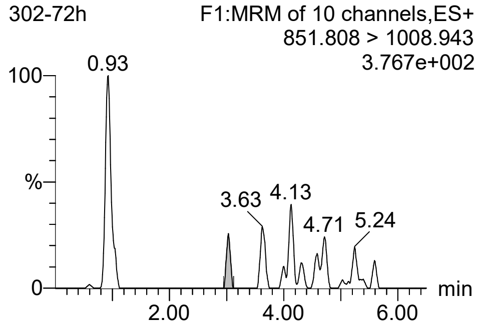

Tolbutamide (1)

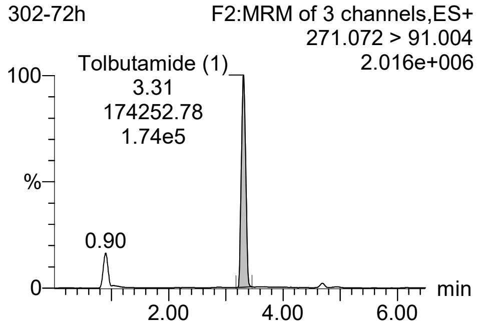

	# Name	Trace	RT	Area	IS Area	Response	Primar...	Conc.	%Dev
1	1 WBPD081_044 (2)	851.808 > 1008....	3.03	7.655	174252.781	0.000	bbl		
2	2 Tolbutamide (1)	271.072 > 91.004	3.31	174252.781		174252.781	bb	1.0	-3.4

Name: 20241213_1_043, ID: 302-96h, Description:

WBPD081_044 (2)

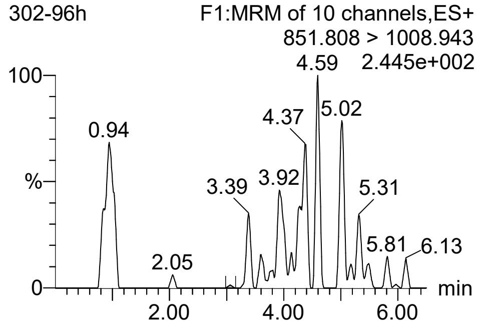

Tolbutamide (1)

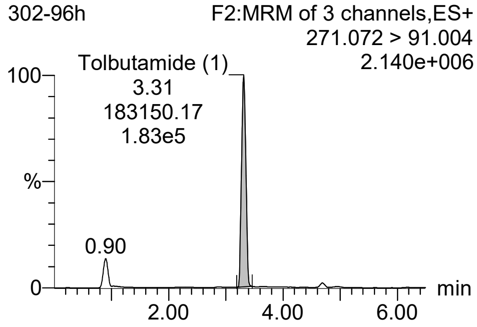

	# Name	Trace	RT	Area	IS Area	Response	Primar...	Conc.	%Dev
1	1 WBPD081_044 (2)	851.808 > 1008....	3.06	0.244	183150.172	0.000	bbl		
2	2 Tolbutamide (1)	271.072 > 91.004	3.31	183150.172		183150.172	bb	1.0	1.5

Name: 20241213_1_044, ID: 302-168h, Description:

WBPD081_044 (2)

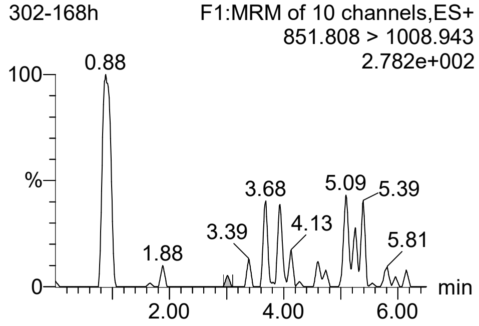

Tolbutamide (1)

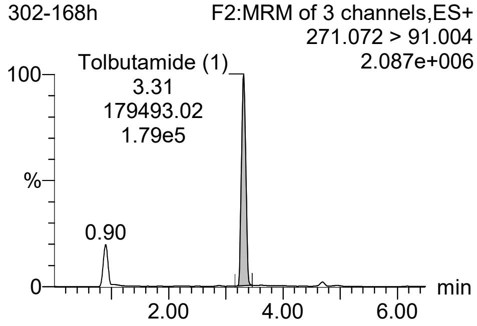

	# Name	Trace	RT	Area	IS Area	Response	Primar...	Conc.	%Dev
1	1 WBPD081_044 (2)	851.808 > 1008....	3.01	1.142	179493.016	0.000	bbl		
2	2 Tolbutamide (1)	271.072 > 91.004	3.31	179493.016		179493.016	bb	1.0	-0.5

Dataset: D:\Data\27013-24001-NG.PRO\20241214_WBPD081_044_SA-Tu.qld

Last Altered: Tuesday, July 15, 2025 15:34:14 China Standard Time
Printed: Tuesday, July 15, 2025 15:38:44 China Standard Time

Name: 20241213_1_045, ID: Solvent, Description:

WBPD081_044 (2)

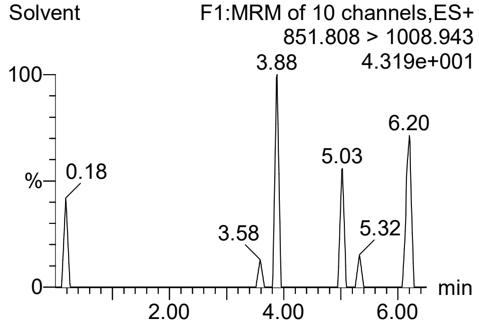

Tolbutamide (1)

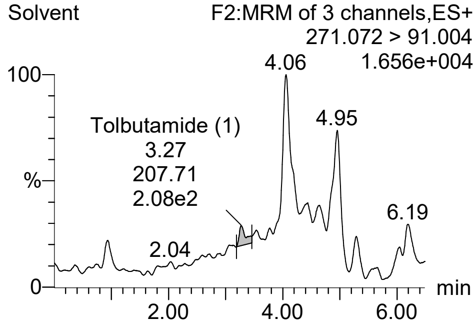

	#	Name	Trace	RT	Area	IS Area	Response	Primar...	Conc.	%Dev
1	1	WBPD081_044 (2)	851.808 > 1008....			207.711				
2	2	Tolbutamide (1)	271.072 > 91.004	3.27	207.711		207.711	bd	0.0	-99.9

Name: 20241213_1_046, ID: Solvent, Description:

WBPD081_044 (2)

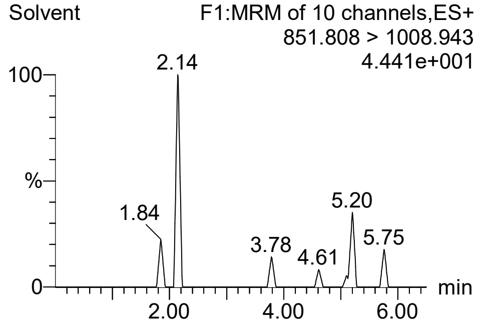

Tolbutamide (1)

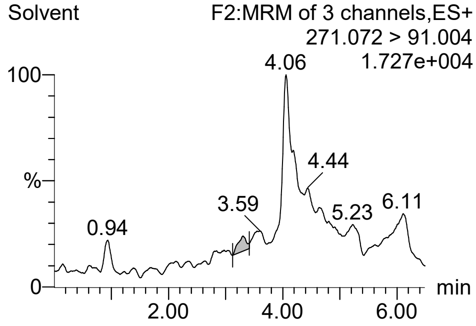

	#	Name	Trace	RT	Area	IS Area	Response	Primar...	Conc.	%Dev
1	1	WBPD081_044 (2)	851.808 > 1008....			207.678				
2	2	Tolbutamide (1)	271.072 > 91.004	3.30	207.678		207.678	bd	0.0	-99.9

Name: 20241213_1_047, ID: 303-Predose, Description:

WBPD081_044 (2)

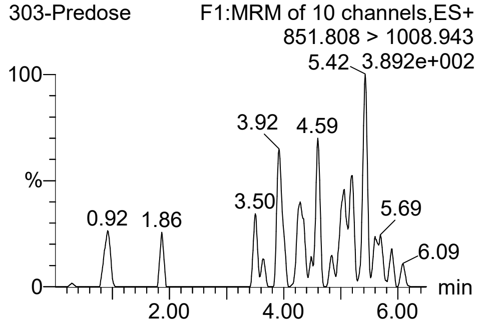

Tolbutamide (1)

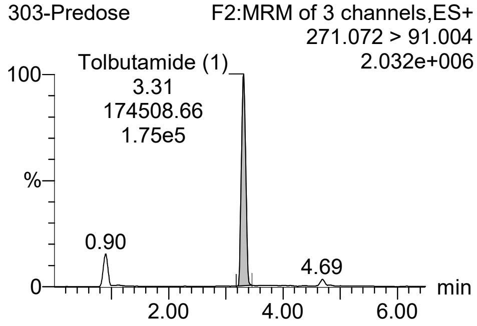

	#	Name	Trace	RT	Area	IS Area	Response	Primar...	Conc.	%Dev
1	1	WBPD081_044 (2)	851.808 > 1008....			174508.656				
2	2	Tolbutamide (1)	271.072 > 91.004	3.31	174508.656		174508.656	bb	1.0	-3.3

Dataset: D:\Data\27013-24001-NG.PRO\20241214_WBPD081_044_SA-Tu.qld

Last Altered: Tuesday, July 15, 2025 15:34:14 China Standard Time
Printed: Tuesday, July 15, 2025 15:38:44 China Standard Time

Name: 20241213_1_048, ID: 303-2h, Description:

WBPD081_044 (2)

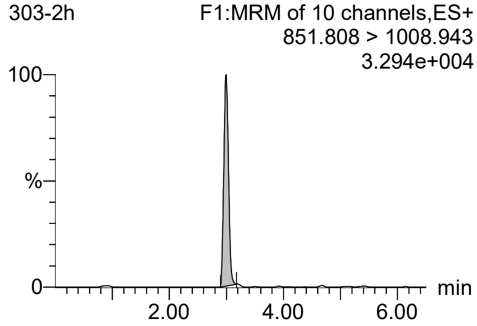

Tolbutamide (1)

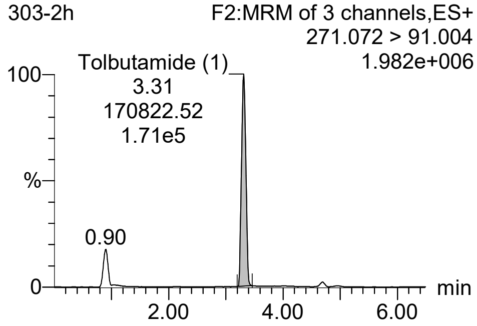

	#	Name	Trace	RT	Area	IS Area	Response	Primar...	Conc.	%Dev
1	1	WBPD081_044 (2)	851.808 > 1008....	3.00	3124.932	170822.516	0.018	bb	70.7	
2	2	Tolbutamide (1)	271.072 > 91.004	3.31	170822.516		170822.516	bb	0.9	-5.3

Name: 20241213_1_049, ID: 303-4h, Description:

WBPD081_044 (2)

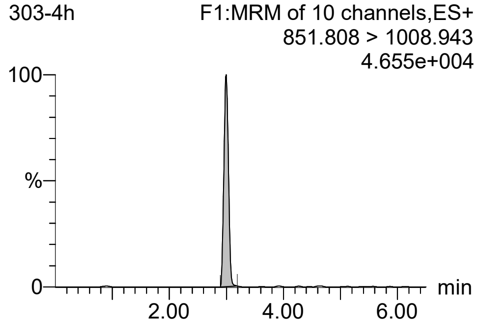

Tolbutamide (1)

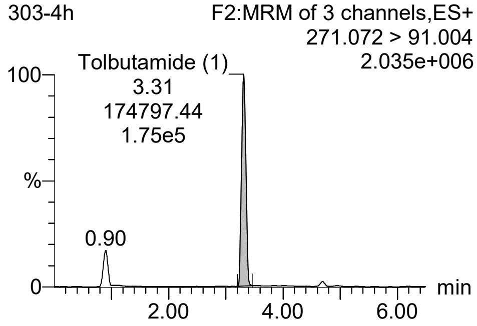

	#	Name	Trace	RT	Area	IS Area	Response	Primar...	Conc.	%Dev
1	1	WBPD081_044 (2)	851.808 > 1008....	3.00	4292.446	174797.438	0.025	bb	95.0	
2	2	Tolbutamide (1)	271.072 > 91.004	3.31	174797.438		174797.438	bb	1.0	-3.1

Name: 20241213_1_050, ID: 303-8h, Description:

WBPD081_044 (2)

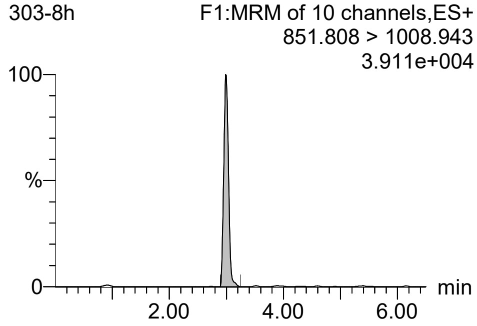

Tolbutamide (1)

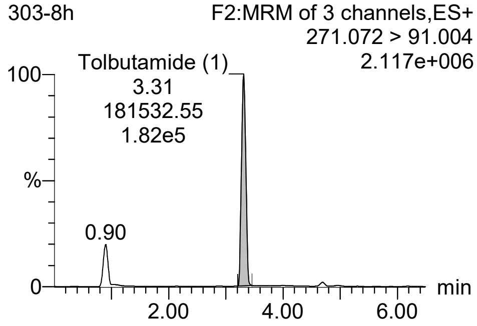

	#	Name	Trace	RT	Area	IS Area	Response	Primar...	Conc.	%Dev
1	1	WBPD081_044 (2)	851.808 > 1008....	2.99	3767.555	181532.547	0.021	bb	80.2	
2	2	Tolbutamide (1)	271.072 > 91.004	3.31	181532.547		181532.547	bb	1.0	0.6

Dataset: D:\Data\27013-24001-NG.PRO\20241214_WBPD081_044_SA-Tu.qld

Last Altered: Tuesday, July 15, 2025 15:34:14 China Standard Time
Printed: Tuesday, July 15, 2025 15:38:44 China Standard Time

Name: 20241213_1_051, ID: 303-12h, Description:

WBPD081_044 (2)

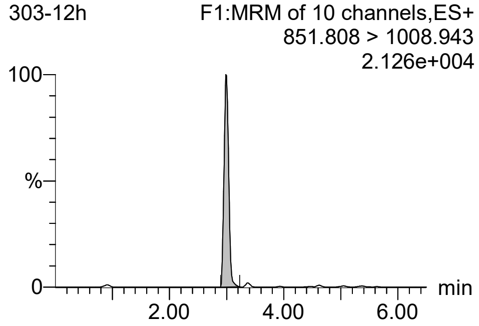

Tolbutamide (1)

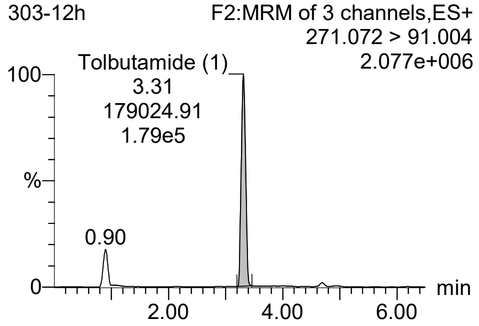

	# Name	Trace	RT	Area	IS Area	Response	Primar...	Conc.	%Dev
1	1 WBPD081_044 (2)	851.808 > 1008....	2.99	2004.371	179024.906	0.011	bb	43.2	
2	2 Tolbutamide (1)	271.072 > 91.004	3.31	179024.906		179024.906	bb	1.0	-0.8

Name: 20241213_1_052, ID: 303-24h, Description:

WBPD081_044 (2)

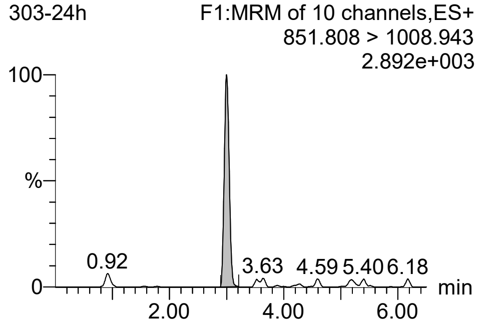

Tolbutamide (1)

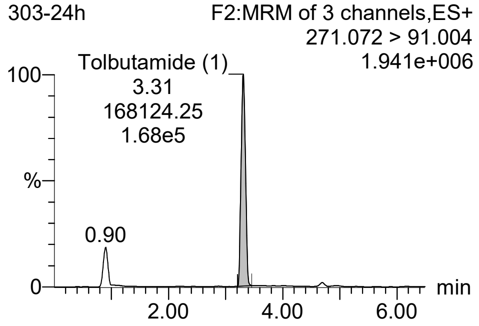

	# Name	Trace	RT	Area	IS Area	Response	Primar...	Conc.	%Dev
1	1 WBPD081_044 (2)	851.808 > 1008....	3.00	290.145	168124.250	0.002	bb	6.4	
2	2 Tolbutamide (1)	271.072 > 91.004	3.31	168124.250		168124.250	bb	0.9	-6.8

Name: 20241213_1_053, ID: 303-48h, Description:

WBPD081_044 (2)

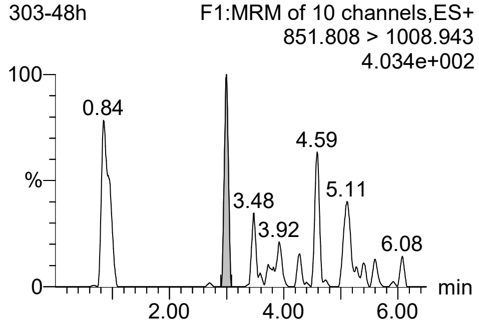

Tolbutamide (1)

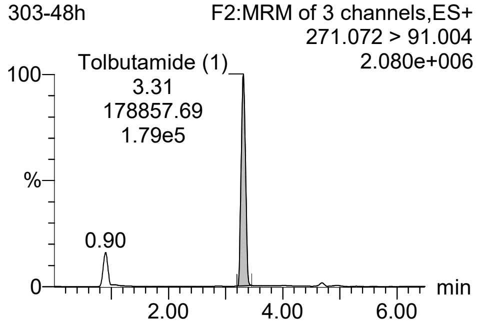

	# Name	Trace	RT	Area	IS Area	Response	Primar...	Conc.	%Dev
1	1 WBPD081_044 (2)	851.808 > 1008....	3.00	33.458	178857.688	0.000	bb	0.5	
2	2 Tolbutamide (1)	271.072 > 91.004	3.31	178857.688		178857.688	bb	1.0	-0.9

Dataset: D:\Data\27013-24001-NG.PRO\20241214_WBPD081_044_SA-Tu.qld

Last Altered: Tuesday, July 15, 2025 15:34:14 China Standard Time
Printed: Tuesday, July 15, 2025 15:38:44 China Standard Time

Name: 20241213_1_054, ID: 303-72h, Description:

WBPD081_044 (2)

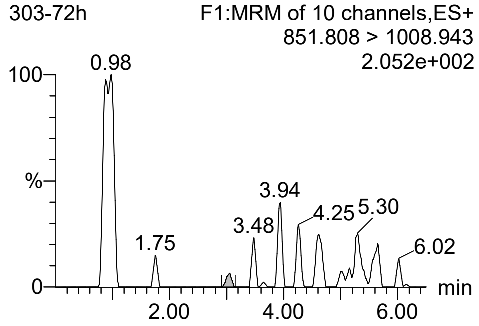

Tolbutamide (1)

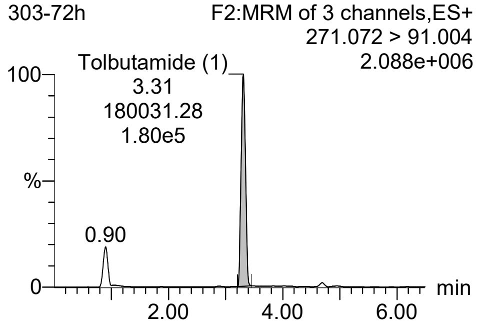

	#	Name	Trace	RT	Area	IS Area	Response	Primar...	Conc.	%Dev
1	1	WBPD081_044 (2)	851.808 > 1008....	3.05	1.415	180031.281	0.000	bbI		
2	2	Tolbutamide (1)	271.072 > 91.004	3.31	180031.281		180031.281	bb	1.0	-0.2

Name: 20241213_1_055, ID: 303-96h, Description:

WBPD081_044 (2)

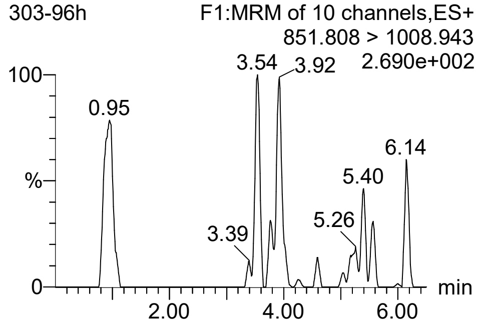

Tolbutamide (1)

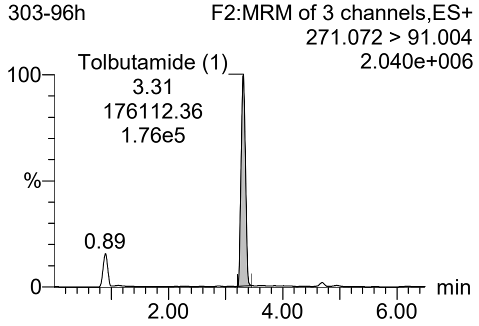

	#	Name	Trace	RT	Area	IS Area	Response	Primar...	Conc.	%Dev
1	1	WBPD081_044 (2)	851.808 > 1008....			176112.359				
2	2	Tolbutamide (1)	271.072 > 91.004	3.31	176112.359		176112.359	bb	1.0	-2.4

Name: 20241213_1_056, ID: 303-168h, Description:

WBPD081_044 (2)

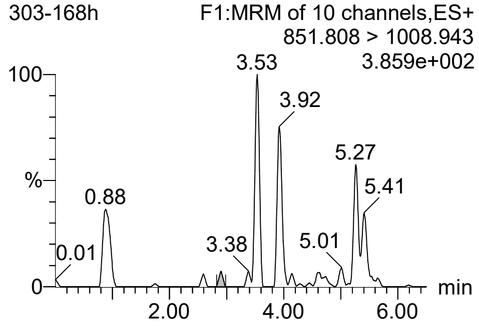

Tolbutamide (1)

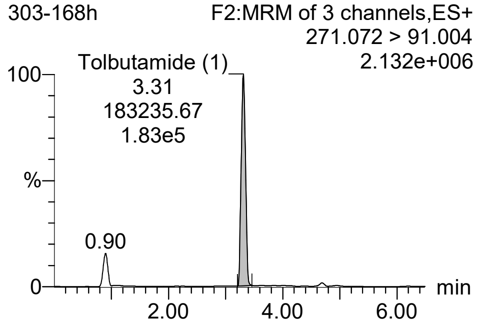

	#	Name	Trace	RT	Area	IS Area	Response	Primar...	Conc.	%Dev
1	1	WBPD081_044 (2)	851.808 > 1008....	2.90	2.180	183235.672	0.000	bbI		
2	2	Tolbutamide (1)	271.072 > 91.004	3.31	183235.672		183235.672	bb	1.0	1.5

Dataset: D:\Data\27013-24001-NG.PRO\20241214_WBPD081_044_SA-Tu.qld

Last Altered: Tuesday, July 15, 2025 15:34:14 China Standard Time
Printed: Tuesday, July 15, 2025 15:38:44 China Standard Time

Name: 20241213_1_057, ID: Solvent, Description:

WBPD081_044 (2)

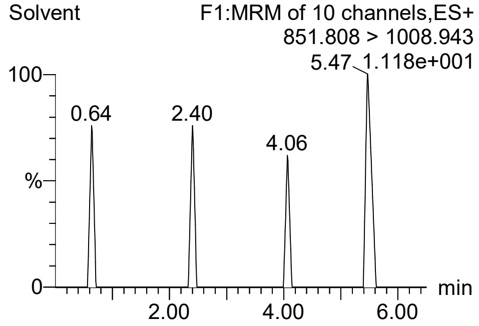

Tolbutamide (1)

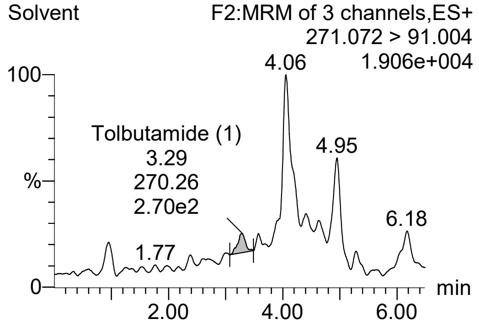

	#	Name	Trace	RT	Area	IS Area	Response	Primar...	Conc.	%Dev
1	1	WBPD081_044 (2)	851.808 > 1008....			270.256				
2	2	Tolbutamide (1)	271.072 > 91.004	3.29	270.256		270.256	bb	0.0	-99.9

Name: 20241213_1_058, ID: Solvent, Description:

WBPD081_044 (2)

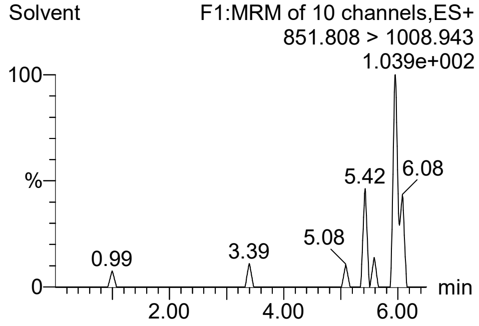

Tolbutamide (1)

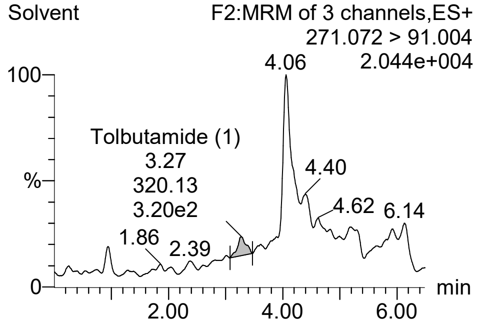

	#	Name	Trace	RT	Area	IS Area	Response	Primar...	Conc.	%Dev
1	1	WBPD081_044 (2)	851.808 > 1008....			320.126				
2	2	Tolbutamide (1)	271.072 > 91.004	3.27	320.126		320.126	bb	0.0	-99.8

Name: 20241213_1_059, ID: B, Description:

WBPD081_044 (2)

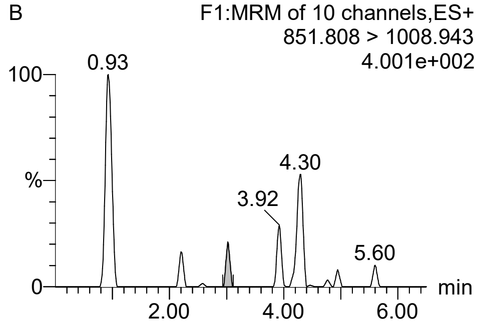

Tolbutamide (1)

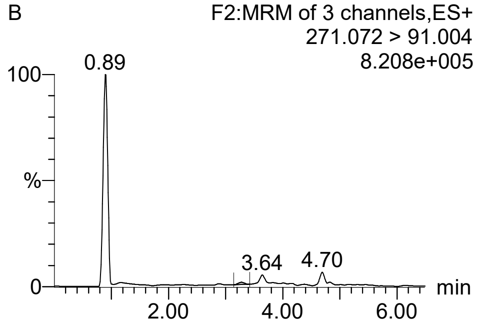

	#	Name	Trace	RT	Area	IS Area	Response	Primar...	Conc.	%Dev
1	1	WBPD081_044 (2)	851.808 > 1008....	3.02	6.577	1105.917	0.006	bb	22.8	
2	2	Tolbutamide (1)	271.072 > 91.004	3.28	1105.917		1105.917	bb	0.0	-99.4

Dataset: D:\Data\27013-24001-NG.PRO\20241214_WBPD081_044_SA-Tu.qld

Last Altered: Tuesday, July 15, 2025 15:34:14 China Standard Time
Printed: Tuesday, July 15, 2025 15:38:44 China Standard Time

Name: 20241213_1_060, ID: O, Description:

WBPD081_044 (2)

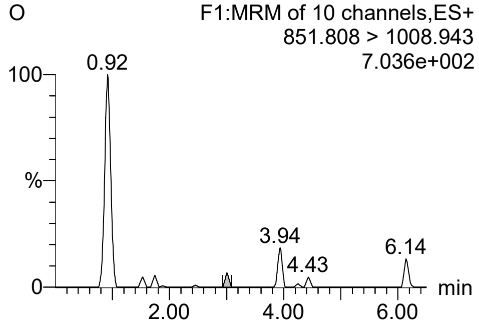

Tolbutamide (1)

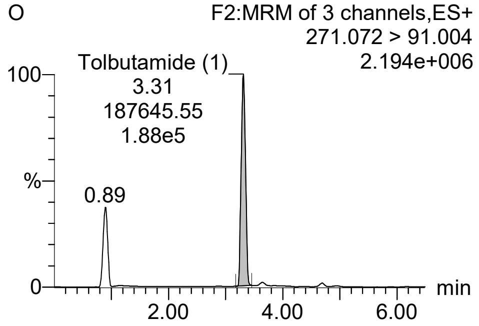

	#	Name	Trace	RT	Area	IS Area	Response	Primar...	Conc.	%Dev
1	1	WBPD081_044 (2)	851.808 > 1008....	3.00	3.643	187645.547	0.000	bbI		
2	2	Tolbutamide (1)	271.072 > 91.004	3.31	187645.547		187645.547	bb	1.0	4.0

Name: 20241213_1_061, ID: Q1, Description:

WBPD081_044 (2)

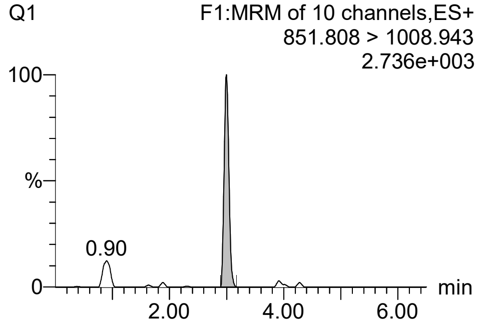

Tolbutamide (1)

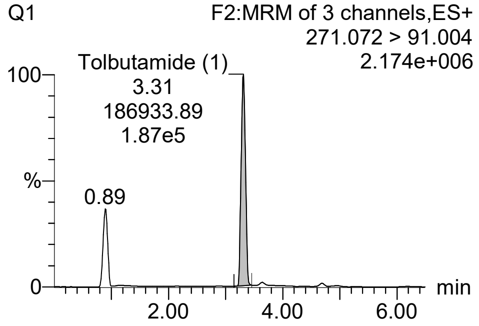

	#	Name	Trace	RT	Area	IS Area	Response	Primar...	Conc.	%Dev
1	1	WBPD081_044 (2)	851.808 > 1008....	3.00	263.514	186933.891	0.001	bb	5.2	-13.4
2	2	Tolbutamide (1)	271.072 > 91.004	3.31	186933.891		186933.891	bb	1.0	3.6

Name: 20241213_1_062, ID: Q2, Description:

WBPD081_044 (2)

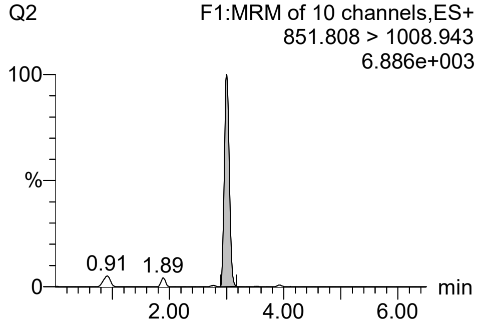

Tolbutamide (1)

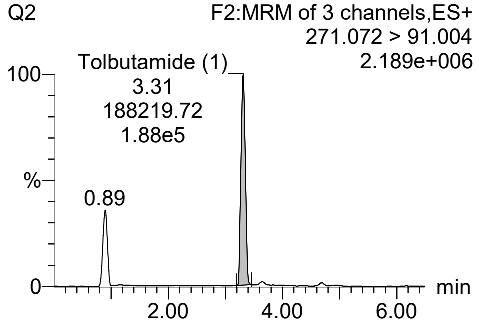

	#	Name	Trace	RT	Area	IS Area	Response	Primar...	Conc.	%Dev
1	1	WBPD081_044 (2)	851.808 > 1008....	3.00	698.430	188219.719	0.004	bb	14.1	17.7
2	2	Tolbutamide (1)	271.072 > 91.004	3.31	188219.719		188219.719	bb	1.0	4.3

Dataset: D:\Data\27013-24001-NG.PRO\20241214_WBPD081_044_SA-Tu.qld

Last Altered: Tuesday, July 15, 2025 15:34:14 China Standard Time
Printed: Tuesday, July 15, 2025 15:38:44 China Standard Time

Name: 20241213_1_063, ID: Q3, Description:

WBPD081_044 (2)

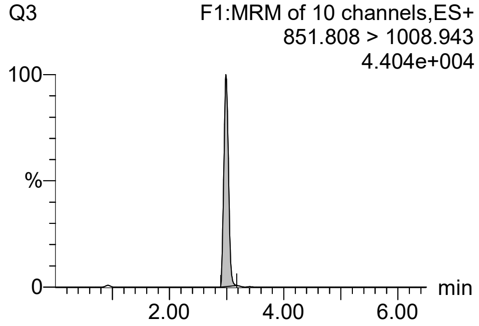

Tolbutamide (1)

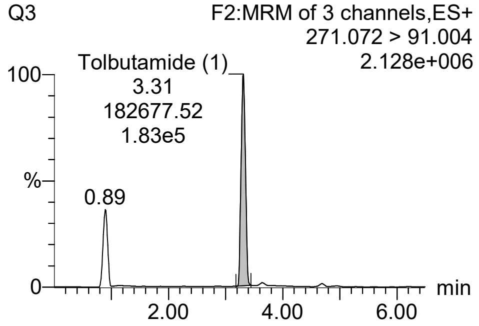

	# Name	Trace	RT	Area	IS Area	Response	Primar...	Conc.	%Dev
1	1 WBPD081_044 (2)	851.808 > 1008....	2.99	4134.252	182677.516	0.023	bb	87.5	9.4
2	2 Tolbutamide (1)	271.072 > 91.004	3.31	182677.516		182677.516	bb	1.0	1.2

Name: 20241213_1_064, ID: Q4, Description:

WBPD081_044 (2)

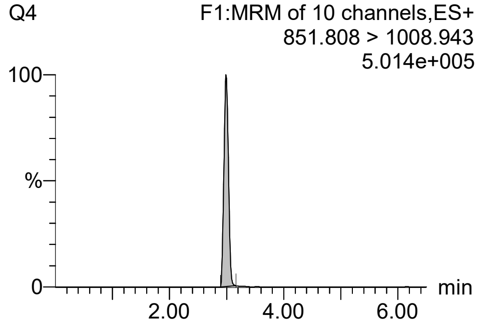

Tolbutamide (1)

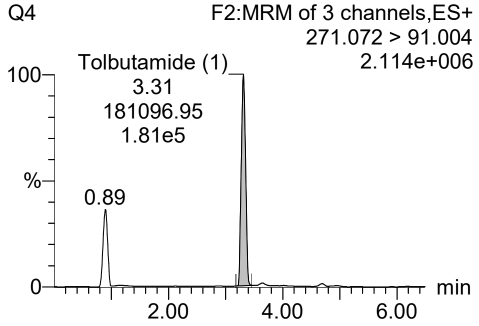

	# Name	Trace	RT	Area	IS Area	Response	Primar...	Conc.	%Dev
1	1 WBPD081_044 (2)	851.808 > 1008....	2.99	46000.078	181096.953	0.254	bb	985.0	23.1
2	2 Tolbutamide (1)	271.072 > 91.004	3.31	181096.953		181096.953	bb	1.0	0.4

Name: 20241213_1_065, ID: Solvent, Description:

WBPD081_044 (2)

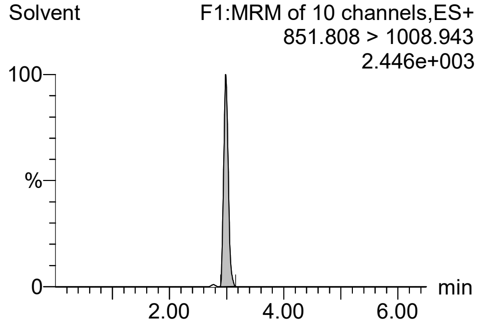

Tolbutamide (1)

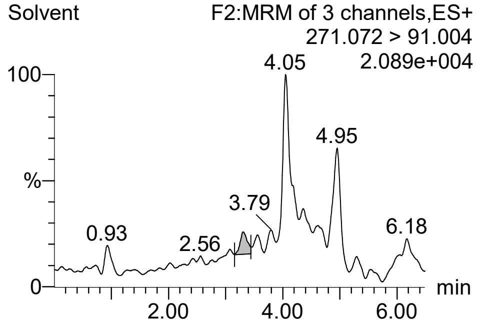

	# Name	Trace	RT	Area	IS Area	Response	Primar...	Conc.	%Dev
1	1 WBPD081_044 (2)	851.808 > 1008....	2.98	228.355	321.493	0.710	bb	2754.9	
2	2 Tolbutamide (1)	271.072 > 91.004	3.30	321.493		321.493	bd	0.0	-99.8

Dataset: D:\Data\27013-24001-NG.PRO\20241214_WBPD081_044_SA-Tu.qld

Last Altered: Tuesday, July 15, 2025 15:34:14 China Standard Time
Printed: Tuesday, July 15, 2025 15:38:44 China Standard Time

Name: 20241213_1_066, ID: Solvent, Description:

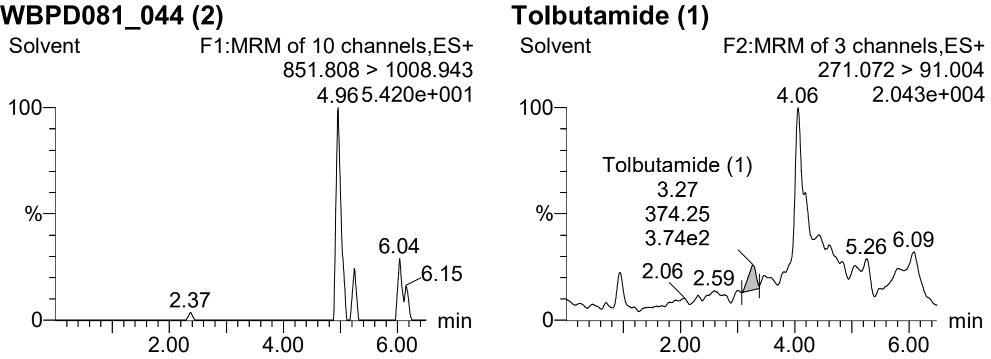

	#	Name	Trace	RT	Area	IS Area	Response	Primar...	Conc.	%Dev
1	1	WBPD081_044 (2)	851.808 > 1008....			374.246				
2	2	Tolbutamide (1)	271.072 > 91.004	3.27	374.246		374.246	bd	0.0	-99.8
