# Supplementary material for: AI‐Driven De Novo Design of Ultra Long‐Acting GLP‐1 Receptor Agonists
Source: Adv Sci (Weinh). 2025 Aug 11;12(40):e07044. doi: 10.1002/advs.202507044 (PMC12561408; doi:10.1002/advs.202507044)
Supplement: Supplementary file 2 — Supporting Information [file ADVS-12-e07044-s001.zip › SI_The raw data of PK for Semaglutide.pdf]

Dataset: D:\Data\27013-24001-NG.PRO\20241213_Soma-Tu.qld

Last Altered: Tuesday, July 15, 2025 15:41:54 China Standard Time
Printed: Tuesday, July 15, 2025 15:43:23 China Standard Time

Method: D:\Data\27013-24001-NG.PRO\MethDB\20241216-Soma.mdb 16 Dec 2024 08:57:51
Calibration: 15 Jul 2025 15:41:54

Compound name: Somaglutide (2)
Correlation coefficient: r = 0.995914, r^2 = 0.991845
Calibration curve: 0.000147982 * x + 7.01497e-006
Response type: Internal Std (Ref 2), Area * (IS Conc. / IS Area)
Curve type: Linear, Origin: Exclude, Weighting: 1/x^2, Axis trans: None

	Name	ID	Type	Std. Conc	RT	Area	IS Area	Response	Conc.	%Dev	Primar...
1	20241213_2_001	Solvent			3.43	4.842	245.998	0.020	133.0		bb
2	20241213_2_002	Solvent					215.745				
3	20241213_2_003	B	Blank		3.35	1.526	602.131	0.003	17.1		bb
4	20241213_2_004	O	Blank		3.26	0.882	213372.203	0.000			bbI
5	20241213_2_005	STD1	Standard	2.000	3.25	70.694	219103.422	0.000	2.1	6.6	bb
6	20241213_2_006	STD2	Standard	5.000	3.26	139.887	221778.391	0.001	4.2	-15.7	bb
7	20241213_2_007	STD3	Standard	10.000	3.26	314.208	213805.500	0.001	9.9	-1.2	bb
8	20241213_2_008	STD4	Standard	50.000	3.26	1544.120	212871.563	0.007	49.0	-2.1	bb
9	20241213_2_009	STD5	Standard	100.000	3.26	3071.410	217039.016	0.014	95.6	-4.4	bb
10	20241213_2_010	STD6	Standard	500.000	3.26	16505.467	219818.813	0.075	507.4	1.5	bb
11	20241213_2_011	STD7	Standard	900.000	3.26	29569.861	214467.016	0.138	931.7	3.5	bb
12	20241213_2_012	STD8	Standard	1000.000	3.26	34869.469	210931.000	0.165	1117.1	11.7	bb
13	20241213_2_013	Solvent			3.26	234.916					bb
14	20241213_2_014	Solvent			3.26	56.410	130.536	0.432	2920.2		bb
15	20241213_2_015	B	Blank		3.25	33.942	891.352	0.038	257.3		bb
16	20241213_2_016	O	Blank		3.26	9.666	209539.516	0.000	0.3		bd
17	20241213_2_017	Q1	QC	6.000	3.26	171.802	212707.750	0.001	5.4	-9.8	bb
18	20241213_2_018	Q2	QC	12.000	3.25	410.303	215531.500	0.002	12.8	6.8	bb
19	20241213_2_019	Q3	QC	80.000	3.26	2772.335	215322.938	0.013	87.0	8.7	bb
20	20241213_2_020	Q4	QC	800.000	3.26	28799.145	215691.734	0.134	902.2	12.8	bb
21	20241213_2_021	Solvent			3.26	160.725	83.575	1.923	12995.6		bb
22	20241213_2_022	Solvent			3.26	58.304	138.354	0.421	2847.7		bb
23	20241213_2_023	401-Predose			3.25	19.332	213885.203	0.000	0.6		bd
24	20241213_2_024	401-2h			3.26	7679.094	215594.703	0.036	240.6		bb
25	20241213_2_025	401-4h			3.26	9442.895	206833.328	0.046	308.5		bb
26	20241213_2_026	401-8h			3.26	9718.216	204647.344	0.047	320.9		bb
27	20241213_2_027	401-12h			3.26	8806.253	210374.672	0.042	282.8		bb
28	20241213_2_028	401-24h			3.26	4108.188	208016.281	0.020	133.4		bb
29	20241213_2_029	401-48h			3.26	400.978	208551.531	0.002	12.9		bb
30	20241213_2_030	401-72h			3.25	46.710	207820.859	0.000	1.5		bb
31	20241213_2_031	401-96h			3.26	31.951	208177.547	0.000	1.0		bd
32	20241213_2_032	401-168h			3.40	14.092	210191.922	0.000	0.4		bb
33	20241213_2_033	Solvent			3.29	28.358					bb
34	20241213_2_034	Solvent					211.996				
35	20241213_2_035	402-Predose			3.27	30.950	209077.188	0.000	1.0		bb
36	20241213_2_036	402-2h			3.26	8216.287	212135.266	0.039	261.7		bb
37	20241213_2_037	402-4h			3.26	8735.833	214604.219	0.041	275.0		bb
38	20241213_2_038	402-8h			3.26	10134.659	209200.563	0.048	327.3		bb
39	20241213_2_039	402-12h			3.26	8681.223	210963.609	0.041	278.0		bb
40	20241213_2_040	402-24h			3.26	4088.476	203923.297	0.020	135.4		bb
41	20241213_2_041	402-48h			3.25	412.613	206753.422	0.002	13.4		bb
42	20241213_2_042	402-72h			3.25	56.528	209410.313	0.000	1.8		bd
43	20241213_2_043	402-96h			3.37	9.397	209881.563	0.000	0.3		db
44	20241213_2_044	402-168h			3.17	7.822	213802.656	0.000	0.2		bb

Dataset: D:\Data\27013-24001-NG.PRO\20241213_Soma-Tu.qld

Last Altered: Tuesday, July 15, 2025 15:41:54 China Standard Time
Printed: Tuesday, July 15, 2025 15:43:23 China Standard Time

Compound name: Somaglutide (2)

	Name	ID	Type	Std. Conc	RT	Area	IS Area	Response	Conc.	%Dev	Primar...
45	20241213_2_045	Solvent			3.27	17.643	230.597	0.077	517.0		bb
46	20241213_2_046	Solvent			3.24	12.137	153.181	0.079	535.4		bd
47	20241213_2_047	403-Predose			3.27	20.738	212836.797	0.000	0.6		dd
48	20241213_2_048	403-2h			3.26	8814.810	218793.188	0.040	272.2		bb
49	20241213_2_049	403-4h			3.25	10591.197	211187.172	0.050	338.9		bb
50	20241213_2_050	403-8h			3.26	11047.925	218719.859	0.051	341.3		bb
51	20241213_2_051	403-12h			3.26	8740.216	211249.984	0.041	279.5		bb
52	20241213_2_052	403-24h			3.26	4053.415	216447.984	0.019	126.5		bb
53	20241213_2_053	403-48h			3.26	489.261	214407.297	0.002	15.4		bb
54	20241213_2_054	403-72h			3.26	53.471	209918.844	0.000	1.7		bb
55	20241213_2_055	403-96h			3.28	16.917	206962.828	0.000	0.5		bb
56	20241213_2_056	403-168h			3.25	2.436	219810.922	0.000	0.0		bb
57	20241213_2_057	Solvent					231.137				
58	20241213_2_058	Solvent			3.21	4.026	247.934	0.016	109.7		bb
59	20241213_2_059	B	Blank		3.32	2.601	712.111	0.004	24.6		dd
60	20241213_2_060	O	Blank		3.33	8.757	210278.641	0.000	0.2		dd
61	20241213_2_061	Q1	QC	6.000	3.26	214.952	207371.516	0.001	7.0	16.0	bd
62	20241213_2_062	Q2	QC	12.000	3.25	439.237	211725.781	0.002	14.0	16.4	bb
63	20241213_2_063	Q3	QC	80.000	3.26	2887.114	211134.203	0.014	92.4	15.4	bb
64	20241213_2_064	Q4	QC	800.000	3.26	30339.646	218614.734	0.139	937.8	17.2	bb
65	20241213_2_065	Solvent			3.26	241.294	189.941	1.270	8584.5		bb
66	20241213_2_066	Solvent			3.26	50.891	169.135	0.301	2033.2		bd

Dataset: D:\Data\27013-24001-NG.PRO\20241213_Soma-Tu.qld

Last Altered: Tuesday, July 15, 2025 15:41:54 China Standard Time
Printed: Tuesday, July 15, 2025 15:43:23 China Standard Time

Compound name: Somaglutide (2)

	Inj. Vol	Factor1	Vial
1	10.000	0.0	3:H,12
2	10.000	0.0	3:H,12
3	10.000	0.0	3:E,1
4	10.000	0.0	3:E,2
5	10.000	1.0	3:E,3
6	10.000	1.0	3:E,4
7	10.000	1.0	3:E,5
8	10.000	1.0	3:E,6
9	10.000	1.0	3:E,7
10	10.000	1.0	3:E,8
11	10.000	1.0	3:E,9
12	10.000	1.0	3:E,10
13	10.000	0.0	3:H,12
14	10.000	0.0	3:H,12
15	10.000	0.0	3:E,1
16	10.000	0.0	3:E,2
17	10.000	0.0	3:E,11
18	10.000	0.0	3:E,12
19	10.000	0.0	3:F,1
20	10.000	0.0	3:F,2
21	10.000	0.0	3:H,12
22	10.000	0.0	3:H,12
23	10.000	0.0	3:F,7
24	10.000	0.0	3:F,8
25	10.000	0.0	3:F,9
26	10.000	0.0	3:F,10
27	10.000	0.0	3:F,11
28	10.000	0.0	3:F,12
29	10.000	0.0	3:G,1
30	10.000	0.0	3:G,2
31	10.000	0.0	3:G,3
32	10.000	0.0	3:G,4
33	10.000	0.0	3:H,12
34	10.000	0.0	3:H,12
35	10.000	0.0	3:G,5
36	10.000	0.0	3:G,6
37	10.000	0.0	3:G,7
38	10.000	0.0	3:G,8
39	10.000	0.0	3:G,9
40	10.000	0.0	3:G,10
41	10.000	0.0	3:G,11
42	10.000	0.0	3:G,12
43	10.000	0.0	3:H,1
44	10.000	0.0	3:H,2
45	10.000	0.0	3:H,12
46	10.000	0.0	3:H,12
47	10.000	0.0	3:H,3
48	10.000	0.0	3:H,4
49	10.000	0.0	3:H,5
50	10.000	0.0	3:H,6
51	10.000	0.0	3:H,7

Dataset: D:\Data\27013-24001-NG.PRO\20241213_Soma-Tu.qld

Last Altered: Tuesday, July 15, 2025 15:41:54 China Standard Time
Printed: Tuesday, July 15, 2025 15:43:23 China Standard Time

Compound name: Somaglutide (2)

	Inj. Vol	Factor1	Vial
52	10.000	0.0	3:H,8
53	10.000	0.0	3:H,9
54	10.000	0.0	3:H,10
55	10.000	0.0	3:H,11
56	10.000	0.0	5:H,12
57	10.000	0.0	3:H,12
58	10.000	0.0	3:H,12
59	10.000	0.0	3:E,1
60	10.000	0.0	3:E,2
61	10.000	0.0	3:F,3
62	10.000	0.0	3:F,4
63	10.000	0.0	3:F,5
64	10.000	0.0	3:F,6
65	10.000	0.0	3:H,12
66	10.000	0.0	3:H,12

Dataset: D:\Data\27013-24001-NG.PRO\20241213_Soma-Tu.qld

Last Altered: Tuesday, July 15, 2025 15:41:54 China Standard Time
Printed: Tuesday, July 15, 2025 15:43:23 China Standard Time

Compound name: Tolbutamide (2)
Response Factor: 216227
RRF SD: 3796.99, Relative SD: 1.75602
Response type: External Std, Area
Curve type: RF

	Name	ID	Type	Std. Conc	RT	Area	IS Area	Response	Conc.	%Dev	Primar...
1	20241213_2_001	Solvent		1.000	3.30	245.998		245.998	0.0	-99.9	bb
2	20241213_2_002	Solvent		1.000	3.30	215.745		215.745	0.0	-99.9	bb
3	20241213_2_003	B	Blank	1.000	3.29	602.131		602.131	0.0	-99.7	bb
4	20241213_2_004	O	Blank	1.000	3.32	213372.203		213372.203	1.0	-1.3	bb
5	20241213_2_005	STD1	Standard	1.000	3.31	219103.422		219103.422	1.0	1.3	bb
6	20241213_2_006	STD2	Standard	1.000	3.31	221778.391		221778.391	1.0	2.6	bb
7	20241213_2_007	STD3	Standard	1.000	3.31	213805.500		213805.500	1.0	-1.1	bb
8	20241213_2_008	STD4	Standard	1.000	3.32	212871.563		212871.563	1.0	-1.6	bb
9	20241213_2_009	STD5	Standard	1.000	3.32	217039.016		217039.016	1.0	0.4	bb
10	20241213_2_010	STD6	Standard	1.000	3.32	219818.813		219818.813	1.0	1.7	bb
11	20241213_2_011	STD7	Standard	1.000	3.32	214467.016		214467.016	1.0	-0.8	bb
12	20241213_2_012	STD8	Standard	1.000	3.32	210931.000		210931.000	1.0	-2.4	bb
13	20241213_2_013	Solvent		1.000							
14	20241213_2_014	Solvent		1.000	3.29	130.536		130.536	0.0	-99.9	bb
15	20241213_2_015	B	Blank	1.000	3.28	891.352		891.352	0.0	-99.6	bb
16	20241213_2_016	O	Blank	1.000	3.32	209539.516		209539.516	1.0	-3.1	bb
17	20241213_2_017	Q1	QC	1.000	3.32	212707.750		212707.750	1.0	-1.6	bb
18	20241213_2_018	Q2	QC	1.000	3.31	215531.500		215531.500	1.0	-0.3	bb
19	20241213_2_019	Q3	QC	1.000	3.31	215322.938		215322.938	1.0	-0.4	bb
20	20241213_2_020	Q4	QC	1.000	3.32	215691.734		215691.734	1.0	-0.2	bb
21	20241213_2_021	Solvent		1.000	3.29	83.575		83.575	0.0	-100.0	bb
22	20241213_2_022	Solvent		1.000	3.30	138.354		138.354	0.0	-99.9	bb
23	20241213_2_023	401-Predose		1.000	3.32	213885.203		213885.203	1.0	-1.1	bb
24	20241213_2_024	401-2h		1.000	3.32	215594.703		215594.703	1.0	-0.3	bb
25	20241213_2_025	401-4h		1.000	3.31	206833.328		206833.328	1.0	-4.3	bb
26	20241213_2_026	401-8h		1.000	3.32	204647.344		204647.344	0.9	-5.4	bb
27	20241213_2_027	401-12h		1.000	3.32	210374.672		210374.672	1.0	-2.7	bb
28	20241213_2_028	401-24h		1.000	3.32	208016.281		208016.281	1.0	-3.8	bb
29	20241213_2_029	401-48h		1.000	3.32	208551.531		208551.531	1.0	-3.5	bb
30	20241213_2_030	401-72h		1.000	3.32	207820.859		207820.859	1.0	-3.9	bb
31	20241213_2_031	401-96h		1.000	3.32	208177.547		208177.547	1.0	-3.7	bb
32	20241213_2_032	401-168h		1.000	3.32	210191.922		210191.922	1.0	-2.8	bb
33	20241213_2_033	Solvent		1.000							
34	20241213_2_034	Solvent		1.000	3.29	211.996		211.996	0.0	-99.9	bb
35	20241213_2_035	402-Predose		1.000	3.32	209077.188		209077.188	1.0	-3.3	bb
36	20241213_2_036	402-2h		1.000	3.32	212135.266		212135.266	1.0	-1.9	bb
37	20241213_2_037	402-4h		1.000	3.31	214604.219		214604.219	1.0	-0.8	bb
38	20241213_2_038	402-8h		1.000	3.32	209200.563		209200.563	1.0	-3.2	bb
39	20241213_2_039	402-12h		1.000	3.32	210963.609		210963.609	1.0	-2.4	bb
40	20241213_2_040	402-24h		1.000	3.32	203923.297		203923.297	0.9	-5.7	bb
41	20241213_2_041	402-48h		1.000	3.31	206753.422		206753.422	1.0	-4.4	bb
42	20241213_2_042	402-72h		1.000	3.31	209410.313		209410.313	1.0	-3.2	bb
43	20241213_2_043	402-96h		1.000	3.31	209881.563		209881.563	1.0	-2.9	bb
44	20241213_2_044	402-168h		1.000	3.31	213802.656		213802.656	1.0	-1.1	bb
45	20241213_2_045	Solvent		1.000	3.30	230.597		230.597	0.0	-99.9	bb
46	20241213_2_046	Solvent		1.000	3.33	153.181		153.181	0.0	-99.9	bb
47	20241213_2_047	403-Predose		1.000	3.31	212836.797		212836.797	1.0	-1.6	bb
48	20241213_2_048	403-2h		1.000	3.32	218793.188		218793.188	1.0	1.2	bb

Dataset: D:\Data\27013-24001-NG.PRO\20241213_Soma-Tu.qld

Last Altered: Tuesday, July 15, 2025 15:41:54 China Standard Time
Printed: Tuesday, July 15, 2025 15:43:23 China Standard Time

Compound name: Tolbutamide (2)

	Name	ID	Type	Std. Conc	RT	Area	IS Area	Response	Conc.	%Dev	Primar...
49	20241213_2_049	403-4h		1.000	3.32	211187.172		211187.172	1.0	-2.3	bb
50	20241213_2_050	403-8h		1.000	3.32	218719.859		218719.859	1.0	1.2	bb
51	20241213_2_051	403-12h		1.000	3.32	211249.984		211249.984	1.0	-2.3	bb
52	20241213_2_052	403-24h		1.000	3.32	216447.984		216447.984	1.0	0.1	bb
53	20241213_2_053	403-48h		1.000	3.32	214407.297		214407.297	1.0	-0.8	bb
54	20241213_2_054	403-72h		1.000	3.32	209918.844		209918.844	1.0	-2.9	bb
55	20241213_2_055	403-96h		1.000	3.32	206962.828		206962.828	1.0	-4.3	bb
56	20241213_2_056	403-168h		1.000	3.32	219810.922		219810.922	1.0	1.7	bb
57	20241213_2_057	Solvent		1.000	3.30	231.137		231.137	0.0	-99.9	bb
58	20241213_2_058	Solvent		1.000	3.28	247.934		247.934	0.0	-99.9	bb
59	20241213_2_059	B	Blank	1.000	3.29	712.111		712.111	0.0	-99.7	bb
60	20241213_2_060	O	Blank	1.000	3.32	210278.641		210278.641	1.0	-2.8	bb
61	20241213_2_061	Q1	QC	1.000	3.31	207371.516		207371.516	1.0	-4.1	bb
62	20241213_2_062	Q2	QC	1.000	3.32	211725.781		211725.781	1.0	-2.1	bb
63	20241213_2_063	Q3	QC	1.000	3.32	211134.203		211134.203	1.0	-2.4	bb
64	20241213_2_064	Q4	QC	1.000	3.31	218614.734		218614.734	1.0	1.1	bb
65	20241213_2_065	Solvent		1.000	3.30	189.941		189.941	0.0	-99.9	bb
66	20241213_2_066	Solvent		1.000	3.30	169.135		169.135	0.0	-99.9	bb

Dataset: D:\Data\27013-24001-NG.PRO\20241213_Soma-Tu.qld

Last Altered: Tuesday, July 15, 2025 15:41:54 China Standard Time
Printed: Tuesday, July 15, 2025 15:43:23 China Standard Time

Compound name: Tolbutamide (2)

	Inj. Vol	Factor1	Vial
1	10.000	1.0	3:H,12
2	10.000	1.0	3:H,12
3	10.000	1.0	3:E,1
4	10.000	1.0	3:E,2
5	10.000	1.0	3:E,3
6	10.000	1.0	3:E,4
7	10.000	1.0	3:E,5
8	10.000	1.0	3:E,6
9	10.000	1.0	3:E,7
10	10.000	1.0	3:E,8
11	10.000	1.0	3:E,9
12	10.000	1.0	3:E,10
13	10.000	1.0	3:H,12
14	10.000	1.0	3:H,12
15	10.000	1.0	3:E,1
16	10.000	1.0	3:E,2
17	10.000	1.0	3:E,11
18	10.000	1.0	3:E,12
19	10.000	1.0	3:F,1
20	10.000	1.0	3:F,2
21	10.000	1.0	3:H,12
22	10.000	1.0	3:H,12
23	10.000	1.0	3:F,7
24	10.000	1.0	3:F,8
25	10.000	1.0	3:F,9
26	10.000	1.0	3:F,10
27	10.000	1.0	3:F,11
28	10.000	1.0	3:F,12
29	10.000	1.0	3:G,1
30	10.000	1.0	3:G,2
31	10.000	1.0	3:G,3
32	10.000	1.0	3:G,4
33	10.000	1.0	3:H,12
34	10.000	1.0	3:H,12
35	10.000	1.0	3:G,5
36	10.000	1.0	3:G,6
37	10.000	1.0	3:G,7
38	10.000	1.0	3:G,8
39	10.000	1.0	3:G,9
40	10.000	1.0	3:G,10
41	10.000	1.0	3:G,11
42	10.000	1.0	3:G,12
43	10.000	1.0	3:H,1
44	10.000	1.0	3:H,2
45	10.000	1.0	3:H,12
46	10.000	1.0	3:H,12
47	10.000	1.0	3:H,3
48	10.000	1.0	3:H,4
49	10.000	1.0	3:H,5
50	10.000	1.0	3:H,6
51	10.000	1.0	3:H,7

Dataset: D:\Data\27013-24001-NG.PRO\20241213_Soma-Tu.qld

Last Altered: Tuesday, July 15, 2025 15:41:54 China Standard Time
Printed: Tuesday, July 15, 2025 15:43:23 China Standard Time

Compound name: Tolbutamide (2)

	Inj. Vol	Factor1	Vial
52	10.000	1.0	3:H,8
53	10.000	1.0	3:H,9
54	10.000	1.0	3:H,10
55	10.000	1.0	3:H,11
56	10.000	1.0	5:H,12
57	10.000	1.0	3:H,12
58	10.000	1.0	3:H,12
59	10.000	1.0	3:E,1
60	10.000	1.0	3:E,2
61	10.000	1.0	3:F,3
62	10.000	1.0	3:F,4
63	10.000	1.0	3:F,5
64	10.000	1.0	3:F,6
65	10.000	1.0	3:H,12
66	10.000	1.0	3:H,12

Dataset: D:\Data\27013-24001-NG.PRO\20241213_Soma-Tu.qld

Last Altered: Tuesday, July 15, 2025 15:41:54 China Standard Time
Printed: Tuesday, July 15, 2025 15:43:23 China Standard Time

Method: D:\Data\27013-24001-NG.PRO\MethDB\20241216-Soma.mdb 16 Dec 2024 08:57:51
Calibration: 15 Jul 2025 15:41:54

Compound name: Somaglutide (2)
Correlation coefficient: r = 0.995914, r^2 = 0.991845
Calibration curve: 0.000147982 * x + 7.01497e-006
Response type: Internal Std (Ref 2), Area * (IS Conc. / IS Area)
Curve type: Linear, Origin: Exclude, Weighting: 1/x^2, Axis trans: None

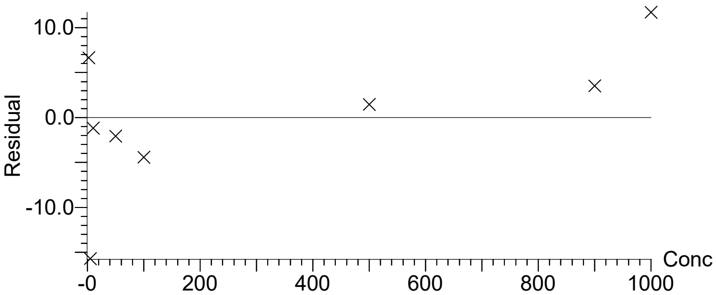

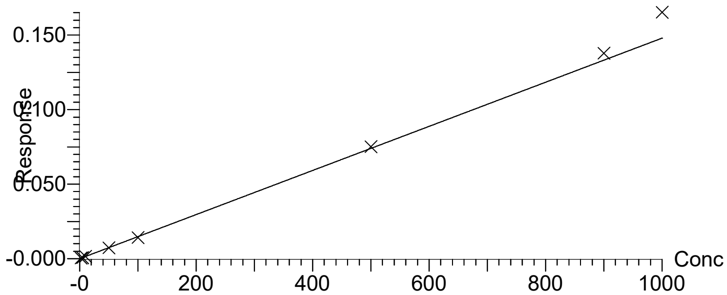

Compound name: Tolbutamide (2)
Response Factor: 216227
RRF SD: 3796.99, % Relative SD: 1.75602
Response type: External Std, Area
Curve type: RF

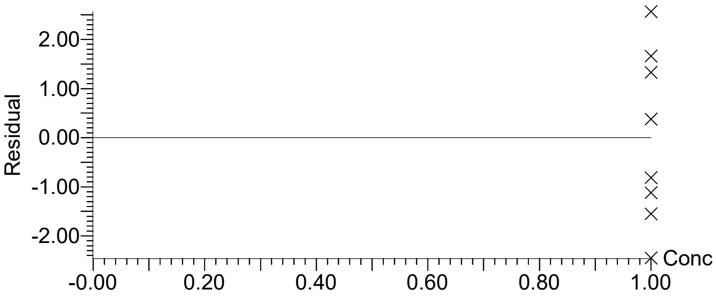

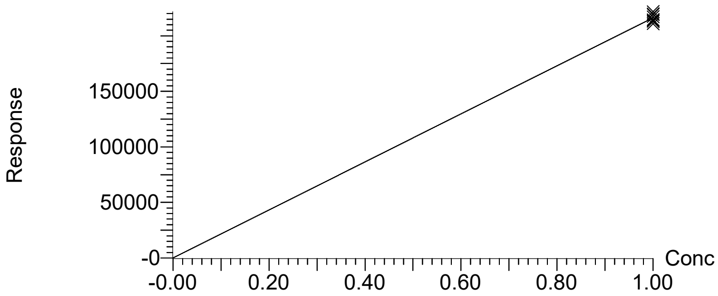

Dataset: D:\Data\27013-24001-NG.PRO\20241213_Soma-Tu.qld

Last Altered: Tuesday, July 15, 2025 15:41:54 China Standard Time
Printed: Tuesday, July 15, 2025 15:43:23 China Standard Time

Method: D:\Data\27013-24001-NG.PRO\MethDB\20241216-Soma.mdb 16 Dec 2024 08:57:51
Calibration: 15 Jul 2025 15:41:54

Name: 20241213_2_001, ID: Solvent, Description:

Somaglutide (2)

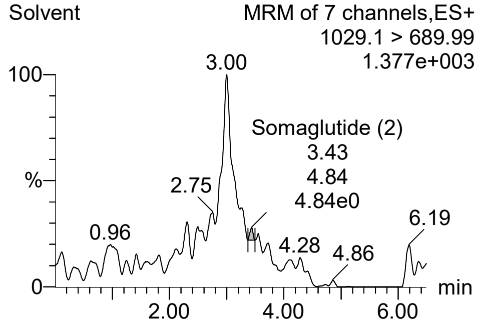

Tolbutamide (2)

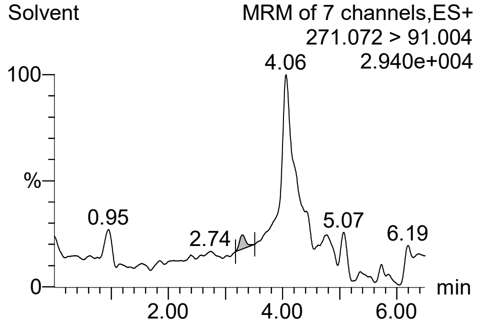

	# Name	Trace	RT	Area	IS Area	Response	Primar...	Conc.	%Dev
1	1 Somaglutide (2)	1029.1 > 689.99	3.43	4.842	245.998	0.020	bb	133.0	
2	2 Tolbutamide (2)	271.072 > 91.004	3.30	245.998		245.998	bb	0.0	-99.9

Name: 20241213_2_002, ID: Solvent, Description:

Somaglutide (2)

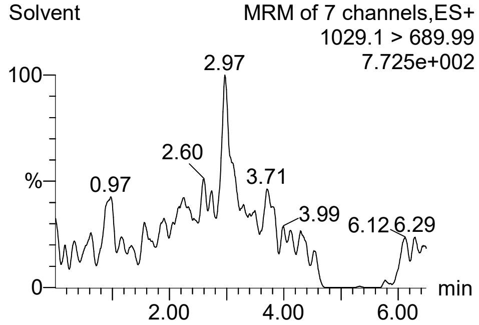

Tolbutamide (2)

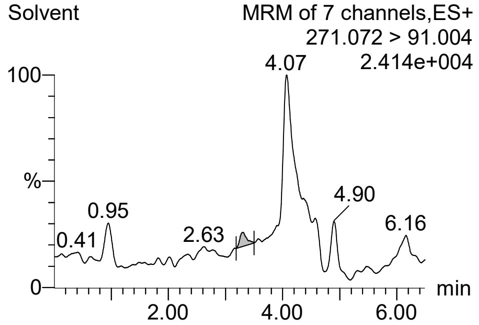

	# Name	Trace	RT	Area	IS Area	Response	Primar...	Conc.	%Dev
1	1 Somaglutide (2)	1029.1 > 689.99			215.745				
2	2 Tolbutamide (2)	271.072 > 91.004	3.30	215.745		215.745	bb	0.0	-99.9

Name: 20241213_2_003, ID: B, Description:

Somaglutide (2)

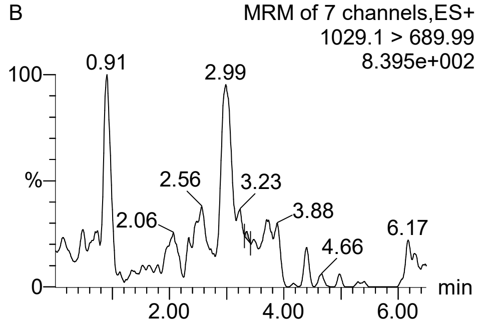

Tolbutamide (2)

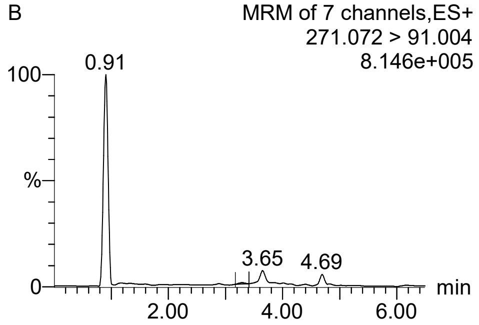

Dataset: D:\Data\27013-24001-NG.PRO\20241213_Soma-Tu.qld

Last Altered: Tuesday, July 15, 2025 15:41:54 China Standard Time
Printed: Tuesday, July 15, 2025 15:43:23 China Standard Time

Name: 20241213_2_003, ID: B, Description:

	# Name	Trace	RT	Area	IS Area	Response	Primar...	Conc.	%Dev
1	1 Somaglutide (2)	1029.1 > 689.99	3.35	1.526	602.131	0.003	bb	17.1	
2	2 Tolbutamide (2)	271.072 > 91.004	3.29	602.131		602.131	bb	0.0	-99.7

Name: 20241213_2_004, ID: O, Description:

Somaglutide (2)

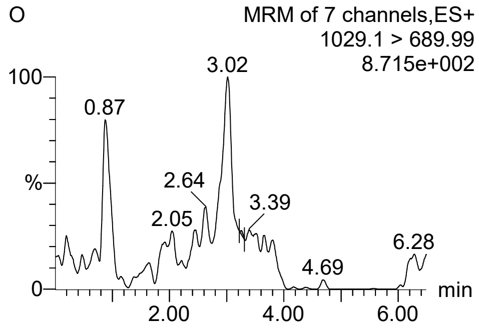

Tolbutamide (2)

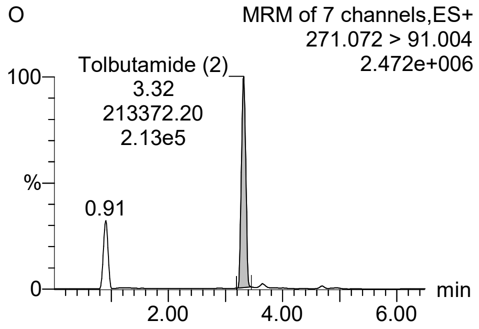

	# Name	Trace	RT	Area	IS Area	Response	Primar...	Conc.	%Dev
1	1 Somaglutide (2)	1029.1 > 689.99	3.26	0.882	213372.203	0.000	bbI		
2	2 Tolbutamide (2)	271.072 > 91.004	3.32	213372.203		213372.203	bb	1.0	-1.3

Name: 20241213_2_005, ID: STD1, Description: SEMA

Somaglutide (2)

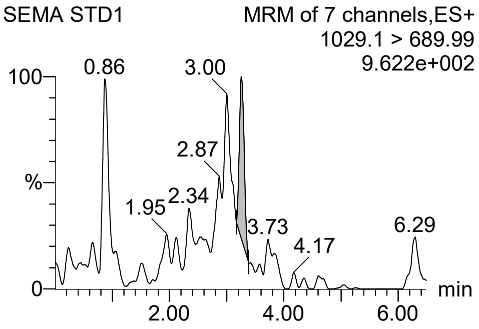

Tolbutamide (2)

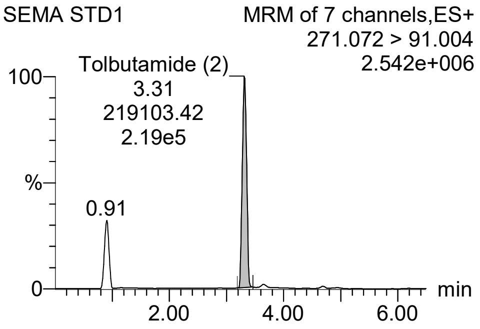

	# Name	Trace	RT	Area	IS Area	Response	Primar...	Conc.	%Dev
1	1 Somaglutide (2)	1029.1 > 689.99	3.25	70.694	219103.422	0.000	bb	2.1	6.6
2	2 Tolbutamide (2)	271.072 > 91.004	3.31	219103.422		219103.422	bb	1.0	1.3

Name: 20241213_2_006, ID: STD2, Description: SEMA

Dataset: D:\Data\27013-24001-NG.PRO\20241213_Soma-Tu.qld

Last Altered: Tuesday, July 15, 2025 15:41:54 China Standard Time
Printed: Tuesday, July 15, 2025 15:43:23 China Standard Time

Name: 20241213_2_006, ID: STD2, Description: SEMA

Somaglutide (2)

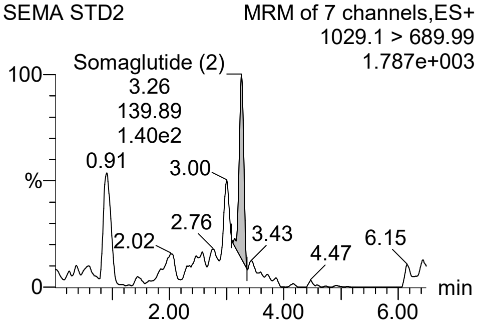

Tolbutamide (2)

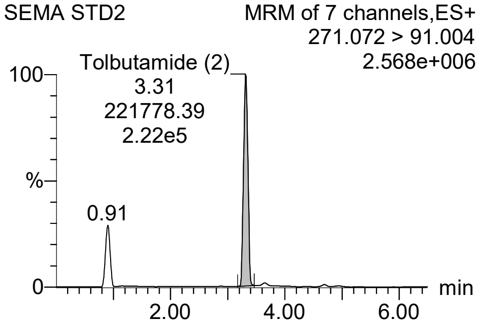

	#	Name	Trace	RT	Area	IS Area	Response	Primar...	Conc.	%Dev
1	1	Somaglutide (2)	1029.1 > 689.99	3.26	139.887	221778.391	0.001	bb	4.2	-15.7
2	2	Tolbutamide (2)	271.072 > 91.004	3.31	221778.391		221778.391	bb	1.0	2.6

Name: 20241213_2_007, ID: STD3, Description: SEMA

Somaglutide (2)

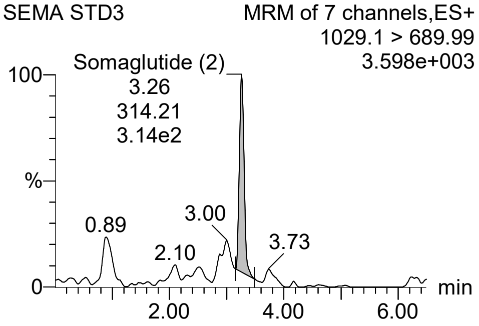

Tolbutamide (2)

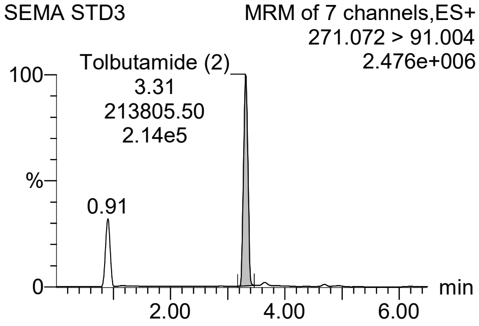

	#	Name	Trace	RT	Area	IS Area	Response	Primar...	Conc.	%Dev
1	1	Somaglutide (2)	1029.1 > 689.99	3.26	314.208	213805.500	0.001	bb	9.9	-1.2
2	2	Tolbutamide (2)	271.072 > 91.004	3.31	213805.500		213805.500	bb	1.0	-1.1

Name: 20241213_2_008, ID: STD4, Description: SEMA

Somaglutide (2)

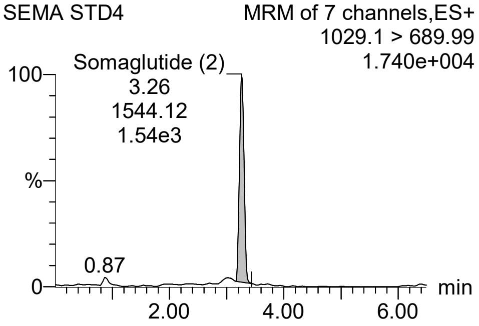

Tolbutamide (2)

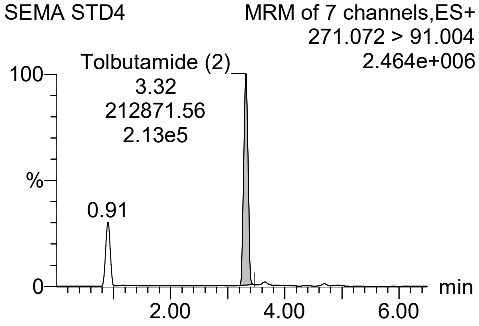

	#	Name	Trace	RT	Area	IS Area	Response	Primar...	Conc.	%Dev
1	1	Somaglutide (2)	1029.1 > 689.99	3.26	1544.120	212871.563	0.007	bb	49.0	-2.1
2	2	Tolbutamide (2)	271.072 > 91.004	3.32	212871.563		212871.563	bb	1.0	-1.6

Dataset: D:\Data\27013-24001-NG.PRO\20241213_Soma-Tu.qld

Last Altered: Tuesday, July 15, 2025 15:41:54 China Standard Time
Printed: Tuesday, July 15, 2025 15:43:23 China Standard Time

Name: 20241213_2_009, ID: STD5, Description: SEMA

Somaglutide (2)

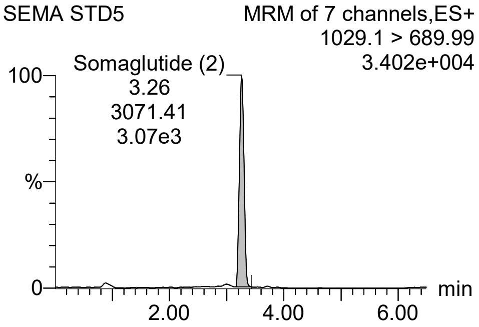

Tolbutamide (2)

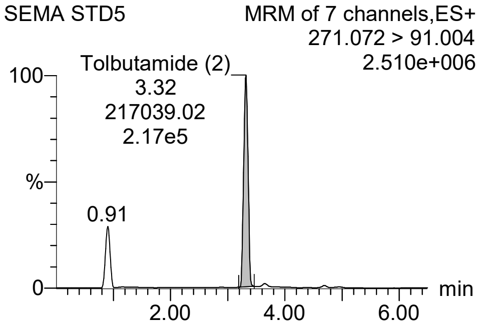

	#	Name	Trace	RT	Area	IS Area	Response	Primar...	Conc.	%Dev
1	1	Somaglutide (2)	1029.1 > 689.99	3.26	3071.410	217039.016	0.014	bb	95.6	-4.4
2	2	Tolbutamide (2)	271.072 > 91.004	3.32	217039.016		217039.016	bb	1.0	0.4

Name: 20241213_2_010, ID: STD6, Description: SEMA

Somaglutide (2)

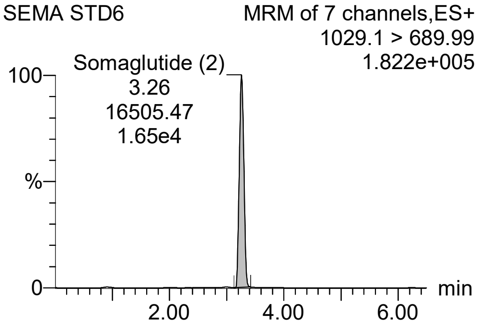

Tolbutamide (2)

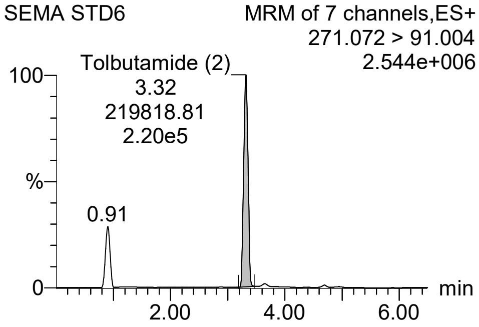

	#	Name	Trace	RT	Area	IS Area	Response	Primar...	Conc.	%Dev
1	1	Somaglutide (2)	1029.1 > 689.99	3.26	16505.467	219818.813	0.075	bb	507.4	1.5
2	2	Tolbutamide (2)	271.072 > 91.004	3.32	219818.813		219818.813	bb	1.0	1.7

Name: 20241213_2_011, ID: STD7, Description: SEMA

Somaglutide (2)

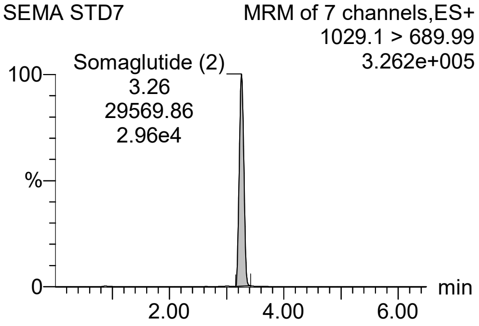

Tolbutamide (2)

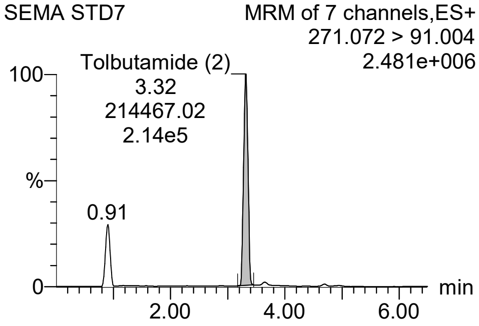

	#	Name	Trace	RT	Area	IS Area	Response	Primar...	Conc.	%Dev
1	1	Somaglutide (2)	1029.1 > 689.99	3.26	29569.861	214467.016	0.138	bb	931.7	3.5
2	2	Tolbutamide (2)	271.072 > 91.004	3.32	214467.016		214467.016	bb	1.0	-0.8

Dataset: D:\Data\27013-24001-NG.PRO\20241213_Soma-Tu.qld

Last Altered: Tuesday, July 15, 2025 15:41:54 China Standard Time
Printed: Tuesday, July 15, 2025 15:43:23 China Standard Time

Name: 20241213_2_012, ID: STD8, Description: SEMA

Somaglutide (2)

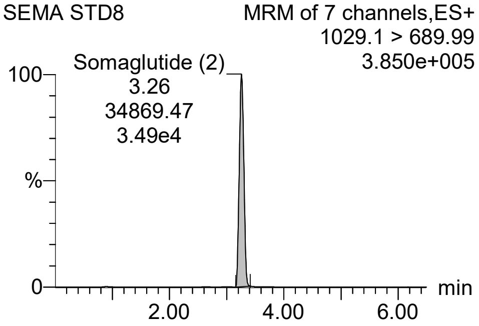

Tolbutamide (2)

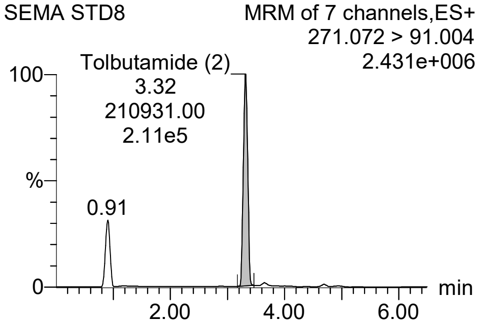

	#	Name	Trace	RT	Area	IS Area	Response	Primar...	Conc.	%Dev
1	1	Somaglutide (2)	1029.1 > 689.99	3.26	34869.469	210931.000	0.165	bb	1117.1	11.7
2	2	Tolbutamide (2)	271.072 > 91.004	3.32	210931.000		210931.000	bb	1.0	-2.4

Name: 20241213_2_013, ID: Solvent, Description:

Somaglutide (2)

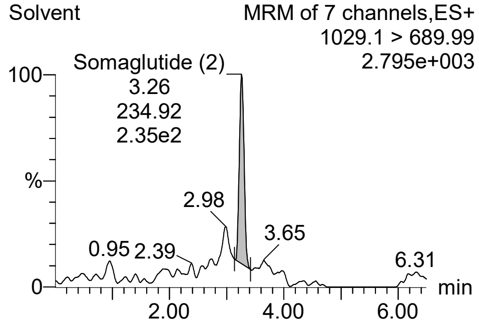

Tolbutamide (2)

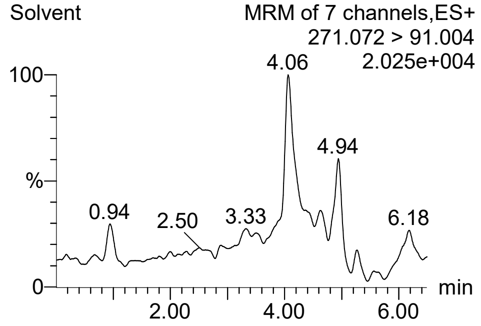

	#	Name	Trace	RT	Area	IS Area	Response	Primar...	Conc.	%Dev
1	1	Somaglutide (2)	1029.1 > 689.99	3.26	234.916			bb		
2	2	Tolbutamide (2)	271.072 > 91.004							

Name: 20241213_2_014, ID: Solvent, Description:

Somaglutide (2)

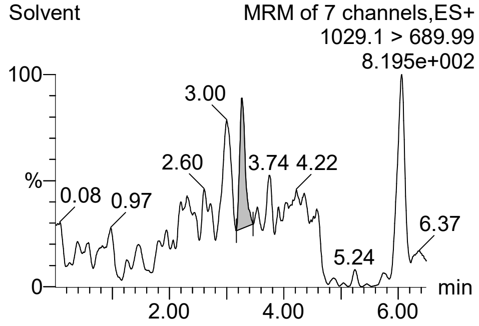

Tolbutamide (2)

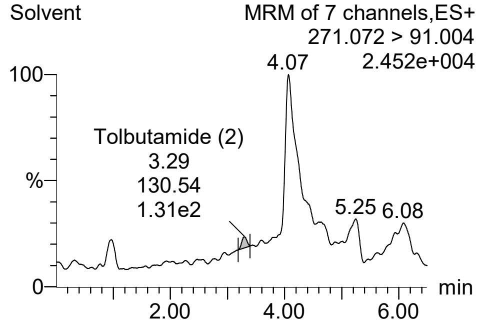

	#	Name	Trace	RT	Area	IS Area	Response	Primar...	Conc.	%Dev
1	1	Somaglutide (2)	1029.1 > 689.99	3.26	56.410	130.536	0.432	bb	2920.2	
2	2	Tolbutamide (2)	271.072 > 91.004	3.29	130.536		130.536	bb	0.0	-99.9

Dataset: D:\Data\27013-24001-NG.PRO\20241213_Soma-Tu.qld

Last Altered: Tuesday, July 15, 2025 15:41:54 China Standard Time
Printed: Tuesday, July 15, 2025 15:43:23 China Standard Time

Name: 20241213_2_015, ID: B, Description:

Somaglutide (2)

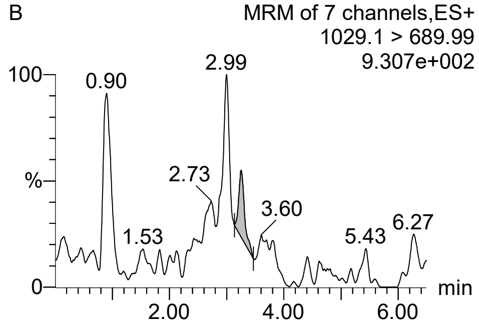

Tolbutamide (2)

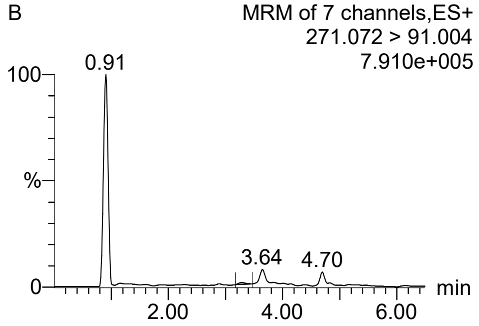

	# Name	Trace	RT	Area	IS Area	Response	Primar...	Conc.	%Dev
1	1 Somaglutide (2)	1029.1 > 689.99	3.25	33.942	891.352	0.038	bb	257.3	
2	2 Tolbutamide (2)	271.072 > 91.004	3.28	891.352		891.352	bb	0.0	-99.6

Name: 20241213_2_016, ID: O, Description:

Somaglutide (2)

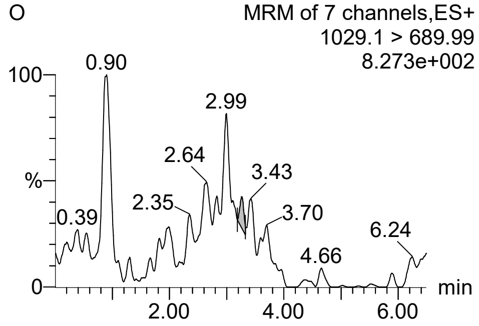

Tolbutamide (2)

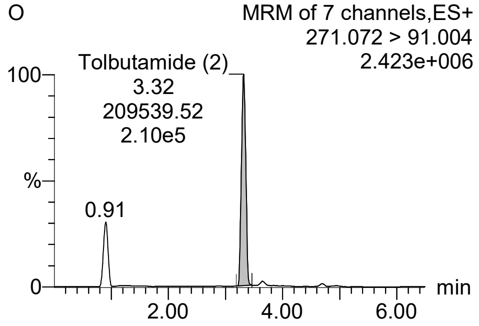

	# Name	Trace	RT	Area	IS Area	Response	Primar...	Conc.	%Dev
1	1 Somaglutide (2)	1029.1 > 689.99	3.26	9.666	209539.516	0.000	bd	0.3	
2	2 Tolbutamide (2)	271.072 > 91.004	3.32	209539.516		209539.516	bb	1.0	-3.1

Name: 20241213_2_017, ID: Q1, Description:

Somaglutide (2)

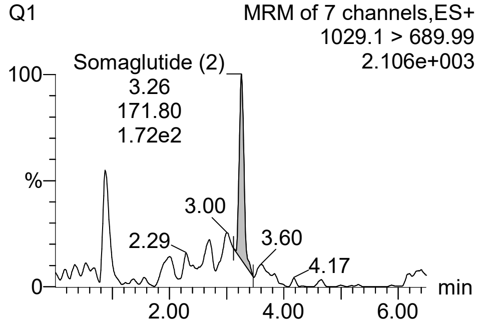

Tolbutamide (2)

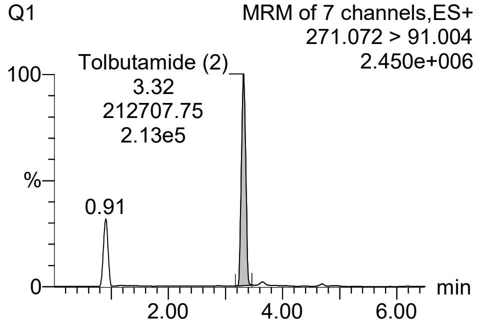

	# Name	Trace	RT	Area	IS Area	Response	Primar...	Conc.	%Dev
1	1 Somaglutide (2)	1029.1 > 689.99	3.26	171.802	212707.750	0.001	bb	5.4	-9.8
2	2 Tolbutamide (2)	271.072 > 91.004	3.32	212707.750		212707.750	bb	1.0	-1.6

Dataset: D:\Data\27013-24001-NG.PRO\20241213_Soma-Tu.qld

Last Altered: Tuesday, July 15, 2025 15:41:54 China Standard Time
Printed: Tuesday, July 15, 2025 15:43:23 China Standard Time

Name: 20241213_2_018, ID: Q2, Description:

Somaglutide (2)

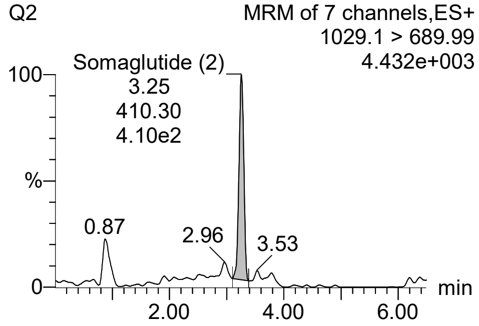

Tolbutamide (2)

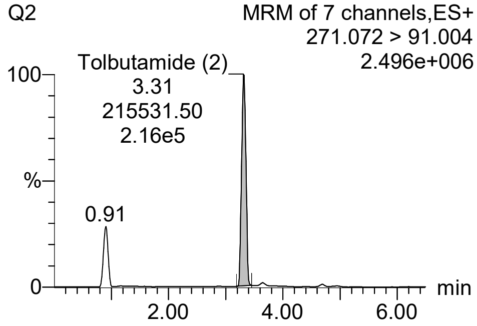

	#	Name	Trace	RT	Area	IS Area	Response	Primar...	Conc.	%Dev
1	1	Somaglutide (2)	1029.1 > 689.99	3.25	410.303	215531.500	0.002	bb	12.8	6.8
2	2	Tolbutamide (2)	271.072 > 91.004	3.31	215531.500		215531.500	bb	1.0	-0.3

Name: 20241213_2_019, ID: Q3, Description:

Somaglutide (2)

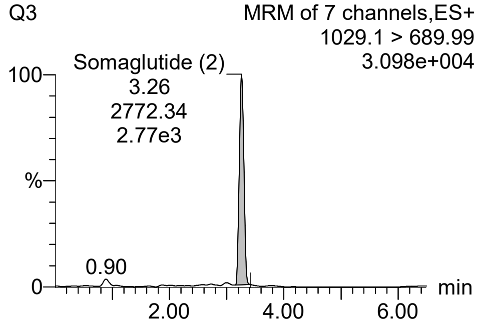

Tolbutamide (2)

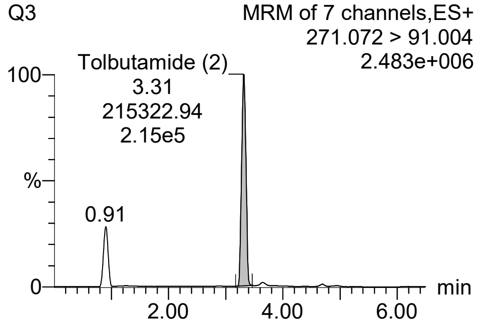

	#	Name	Trace	RT	Area	IS Area	Response	Primar...	Conc.	%Dev
1	1	Somaglutide (2)	1029.1 > 689.99	3.26	2772.335	215322.938	0.013	bb	87.0	8.7
2	2	Tolbutamide (2)	271.072 > 91.004	3.31	215322.938		215322.938	bb	1.0	-0.4

Name: 20241213_2_020, ID: Q4, Description:

Somaglutide (2)

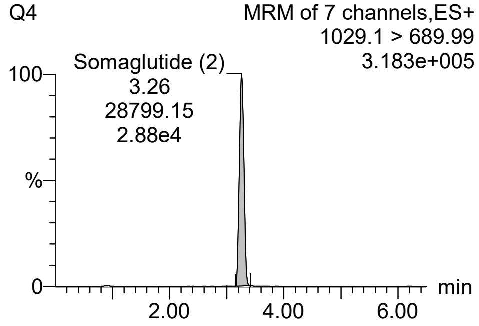

Tolbutamide (2)

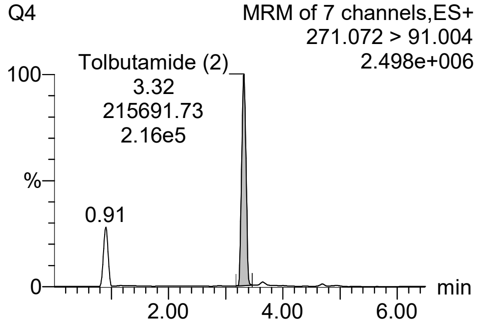

	#	Name	Trace	RT	Area	IS Area	Response	Primar...	Conc.	%Dev
1	1	Somaglutide (2)	1029.1 > 689.99	3.26	28799.145	215691.734	0.134	bb	902.2	12.8
2	2	Tolbutamide (2)	271.072 > 91.004	3.32	215691.734		215691.734	bb	1.0	-0.2

Dataset: D:\Data\27013-24001-NG.PRO\20241213_Soma-Tu.qld

Last Altered: Tuesday, July 15, 2025 15:41:54 China Standard Time
Printed: Tuesday, July 15, 2025 15:43:23 China Standard Time

Name: 20241213_2_021, ID: Solvent, Description:

Somaglutide (2)

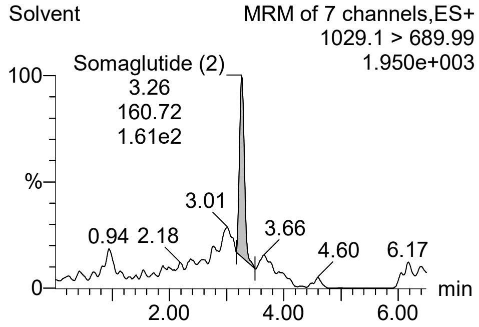

Tolbutamide (2)

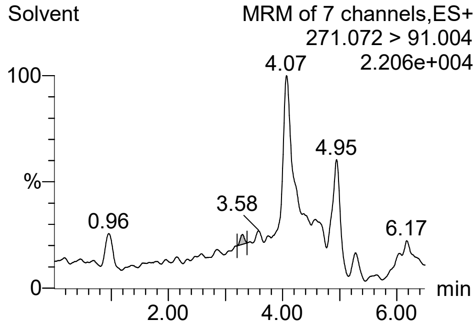

	#	Name	Trace	RT	Area	IS Area	Response	Primar...	Conc.	%Dev
1	1	Somaglutide (2)	1029.1 > 689.99	3.26	160.725	83.575	1.923	bb	12995.6	
2	2	Tolbutamide (2)	271.072 > 91.004	3.29	83.575		83.575	bb	0.0	-100.0

Name: 20241213_2_022, ID: Solvent, Description:

Somaglutide (2)

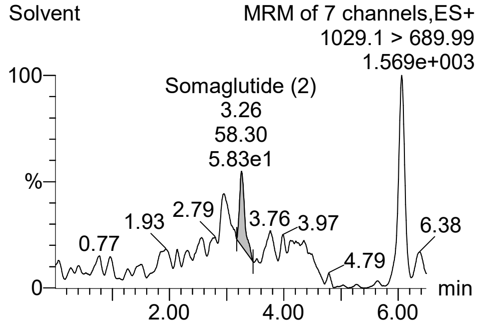

Tolbutamide (2)

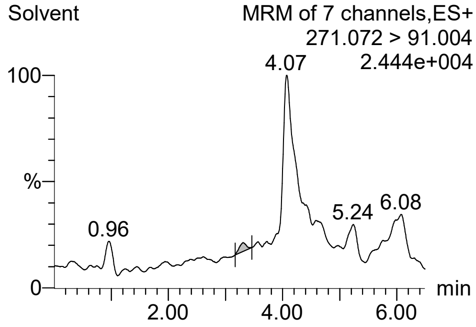

	#	Name	Trace	RT	Area	IS Area	Response	Primar...	Conc.	%Dev
1	1	Somaglutide (2)	1029.1 > 689.99	3.26	58.304	138.354	0.421	bb	2847.7	
2	2	Tolbutamide (2)	271.072 > 91.004	3.30	138.354		138.354	bb	0.0	-99.9

Name: 20241213_2_023, ID: 401-Predose, Description:

Somaglutide (2)

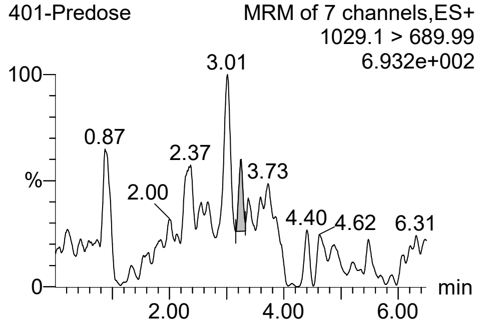

Tolbutamide (2)

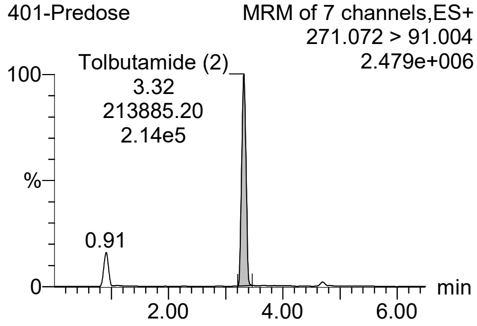

	#	Name	Trace	RT	Area	IS Area	Response	Primar...	Conc.	%Dev
1	1	Somaglutide (2)	1029.1 > 689.99	3.25	19.332	213885.203	0.000	bd	0.6	
2	2	Tolbutamide (2)	271.072 > 91.004	3.32	213885.203		213885.203	bb	1.0	-1.1

Dataset: D:\Data\27013-24001-NG.PRO\20241213_Soma-Tu.qld

Last Altered: Tuesday, July 15, 2025 15:41:54 China Standard Time
Printed: Tuesday, July 15, 2025 15:43:23 China Standard Time

Name: 20241213_2_024, ID: 401-2h, Description:

Somaglutide (2)

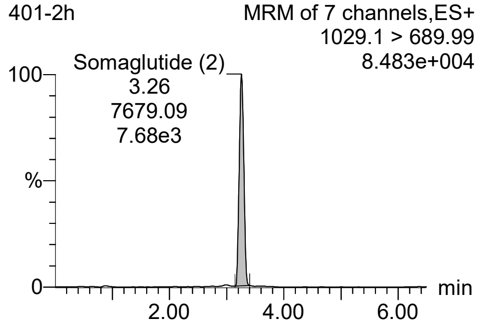

Tolbutamide (2)

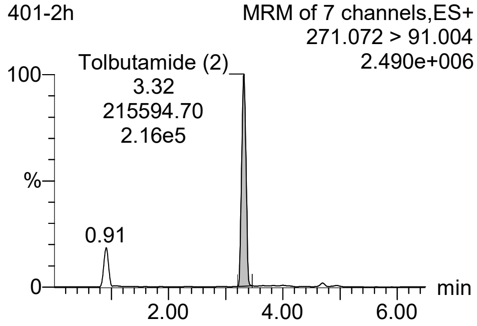

	#	Name	Trace	RT	Area	IS Area	Response	Primar...	Conc.	%Dev
1	1	Somaglutide (2)	1029.1 > 689.99	3.26	7679.094	215594.703	0.036	bb	240.6	
2	2	Tolbutamide (2)	271.072 > 91.004	3.32	215594.703		215594.703	bb	1.0	-0.3

Name: 20241213_2_025, ID: 401-4h, Description:

Somaglutide (2)

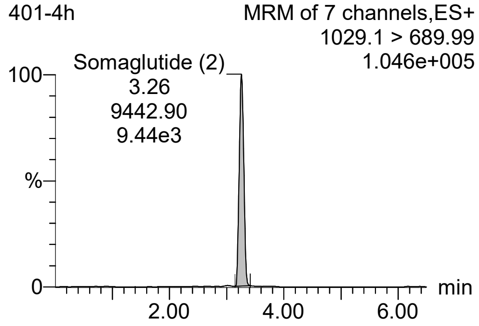

Tolbutamide (2)

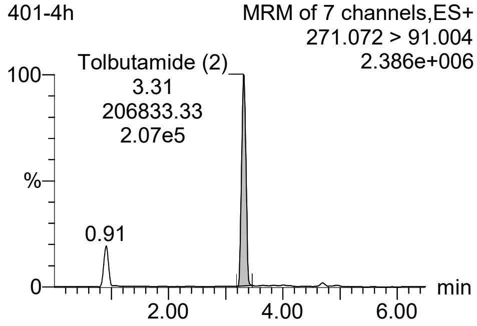

	#	Name	Trace	RT	Area	IS Area	Response	Primar...	Conc.	%Dev
1	1	Somaglutide (2)	1029.1 > 689.99	3.26	9442.895	206833.328	0.046	bb	308.5	
2	2	Tolbutamide (2)	271.072 > 91.004	3.31	206833.328		206833.328	bb	1.0	-4.3

Name: 20241213_2_026, ID: 401-8h, Description:

Somaglutide (2)

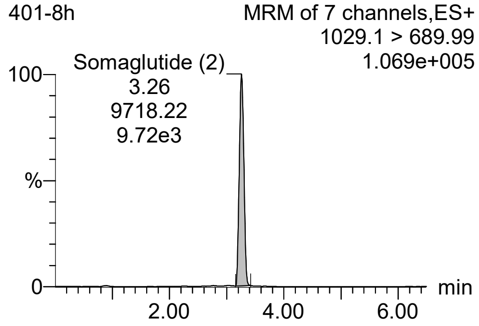

Tolbutamide (2)

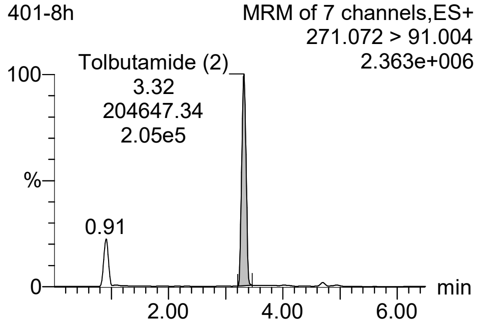

	#	Name	Trace	RT	Area	IS Area	Response	Primar...	Conc.	%Dev
1	1	Somaglutide (2)	1029.1 > 689.99	3.26	9718.216	204647.344	0.047	bb	320.9	
2	2	Tolbutamide (2)	271.072 > 91.004	3.32	204647.344		204647.344	bb	0.9	-5.4

Dataset: D:\Data\27013-24001-NG.PRO\20241213_Soma-Tu.qld

Last Altered: Tuesday, July 15, 2025 15:41:54 China Standard Time
Printed: Tuesday, July 15, 2025 15:43:23 China Standard Time

Name: 20241213_2_027, ID: 401-12h, Description:

Somaglutide (2)

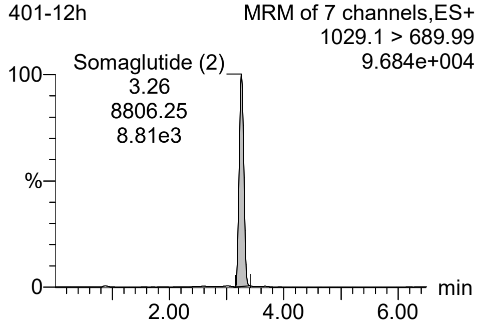

Tolbutamide (2)

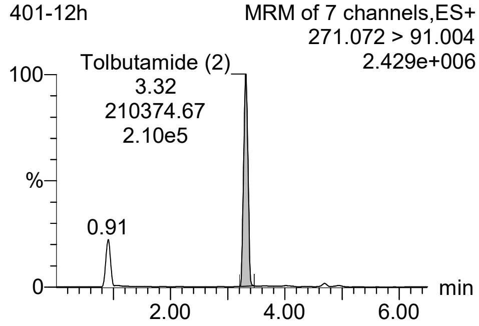

	#	Name	Trace	RT	Area	IS Area	Response	Primar...	Conc.	%Dev
1	1	Somaglutide (2)	1029.1 > 689.99	3.26	8806.253	210374.672	0.042	bb	282.8	
2	2	Tolbutamide (2)	271.072 > 91.004	3.32	210374.672		210374.672	bb	1.0	-2.7

Name: 20241213_2_028, ID: 401-24h, Description:

Somaglutide (2)

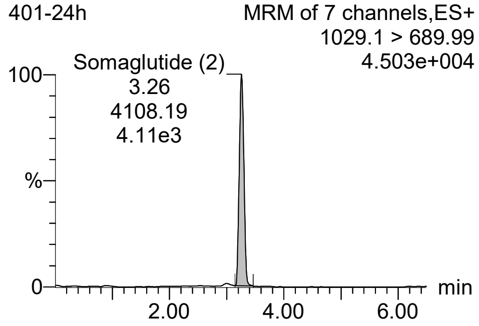

Tolbutamide (2)

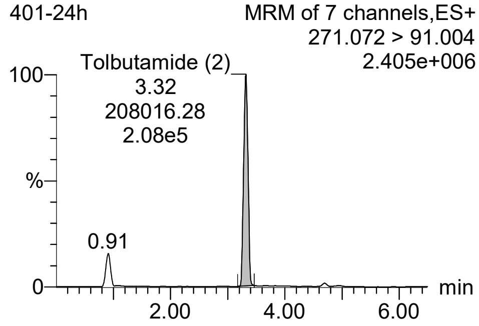

	#	Name	Trace	RT	Area	IS Area	Response	Primar...	Conc.	%Dev
1	1	Somaglutide (2)	1029.1 > 689.99	3.26	4108.188	208016.281	0.020	bb	133.4	
2	2	Tolbutamide (2)	271.072 > 91.004	3.32	208016.281		208016.281	bb	1.0	-3.8

Name: 20241213_2_029, ID: 401-48h, Description:

Somaglutide (2)

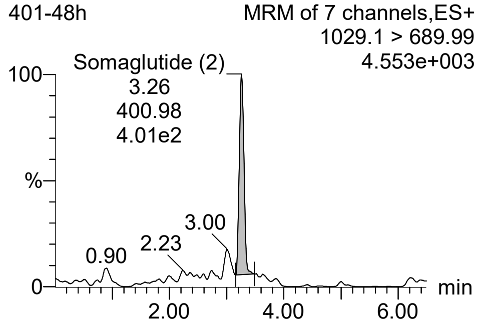

Tolbutamide (2)

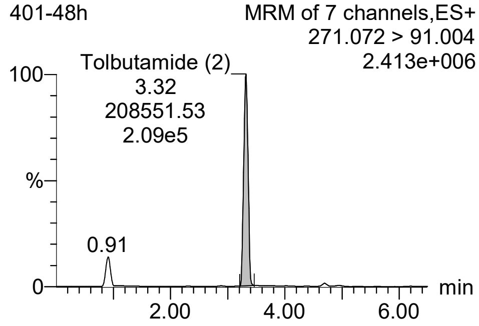

	#	Name	Trace	RT	Area	IS Area	Response	Primar...	Conc.	%Dev
1	1	Somaglutide (2)	1029.1 > 689.99	3.26	400.978	208551.531	0.002	bb	12.9	
2	2	Tolbutamide (2)	271.072 > 91.004	3.32	208551.531		208551.531	bb	1.0	-3.5

Dataset: D:\Data\27013-24001-NG.PRO\20241213_Soma-Tu.qld

Last Altered: Tuesday, July 15, 2025 15:41:54 China Standard Time
Printed: Tuesday, July 15, 2025 15:43:23 China Standard Time

Name: 20241213_2_030, ID: 401-72h, Description:

Somaglutide (2)

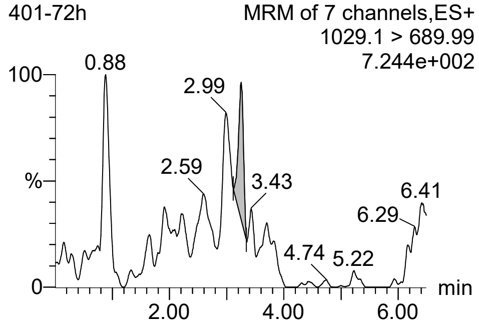

Tolbutamide (2)

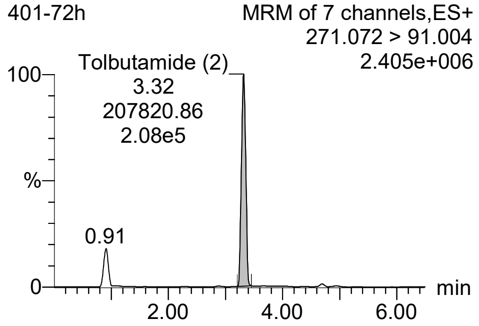

	# Name	Trace	RT	Area	IS Area	Response	Primar...	Conc.	%Dev
1	1 Somaglutide (2)	1029.1 > 689.99	3.25	46.710	207820.859	0.000	bb	1.5	
2	2 Tolbutamide (2)	271.072 > 91.004	3.32	207820.859		207820.859	bb	1.0	-3.9

Name: 20241213_2_031, ID: 401-96h, Description:

Somaglutide (2)

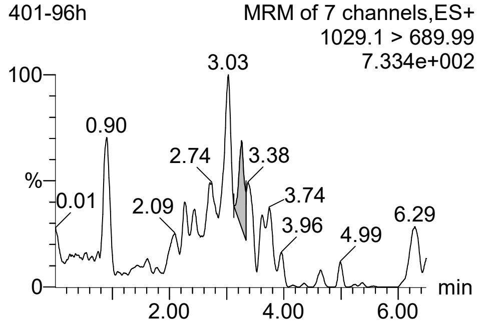

Tolbutamide (2)

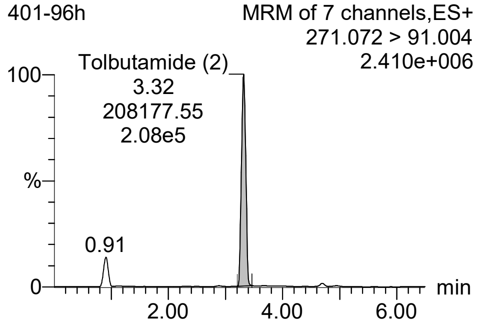

	# Name	Trace	RT	Area	IS Area	Response	Primar...	Conc.	%Dev
1	1 Somaglutide (2)	1029.1 > 689.99	3.26	31.951	208177.547	0.000	bd	1.0	
2	2 Tolbutamide (2)	271.072 > 91.004	3.32	208177.547		208177.547	bb	1.0	-3.7

Name: 20241213_2_032, ID: 401-168h, Description:

Somaglutide (2)

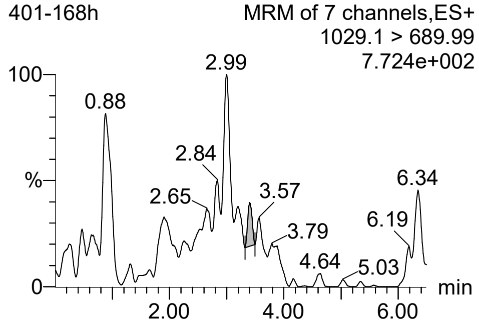

Tolbutamide (2)

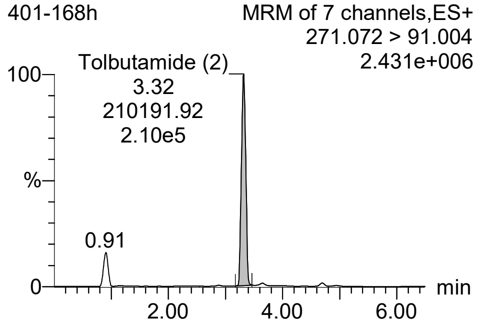

	# Name	Trace	RT	Area	IS Area	Response	Primar...	Conc.	%Dev
1	1 Somaglutide (2)	1029.1 > 689.99	3.40	14.092	210191.922	0.000	bb	0.4	
2	2 Tolbutamide (2)	271.072 > 91.004	3.32	210191.922		210191.922	bb	1.0	-2.8

Dataset: D:\Data\27013-24001-NG.PRO\20241213_Soma-Tu.qld

Last Altered: Tuesday, July 15, 2025 15:41:54 China Standard Time
Printed: Tuesday, July 15, 2025 15:43:23 China Standard Time

Name: 20241213_2_033, ID: Solvent, Description:

Somaglutide (2)

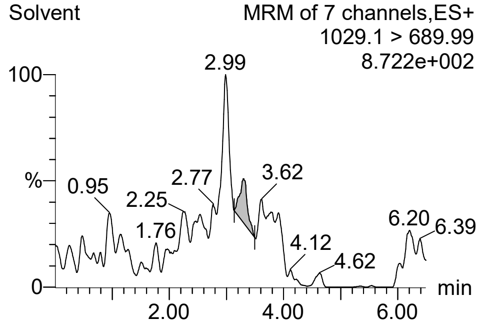

Tolbutamide (2)

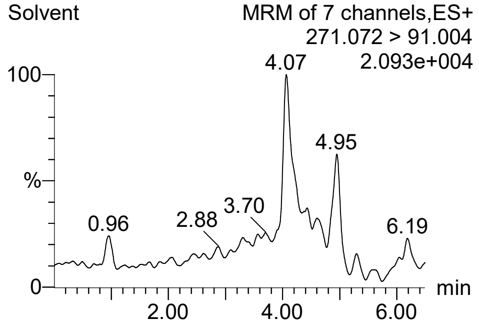

	#	Name	Trace	RT	Area	IS Area	Response	Primar...	Conc.	%Dev
1	1	Somaglutide (2)	1029.1 > 689.99	3.29	28.358			bb		
2	2	Tolbutamide (2)	271.072 > 91.004							

Name: 20241213_2_034, ID: Solvent, Description:

Somaglutide (2)

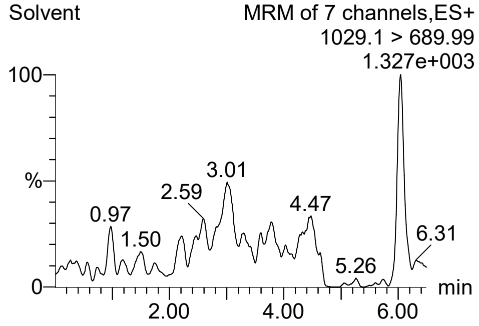

Tolbutamide (2)

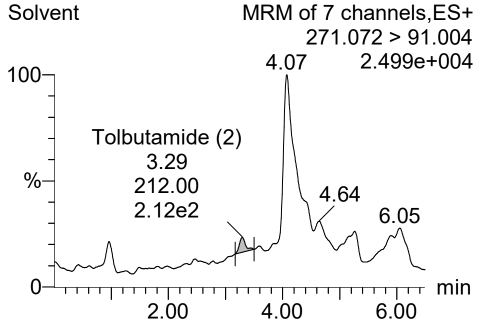

	#	Name	Trace	RT	Area	IS Area	Response	Primar...	Conc.	%Dev
1	1	Somaglutide (2)	1029.1 > 689.99			211.996				
2	2	Tolbutamide (2)	271.072 > 91.004	3.29	211.996		211.996	bb	0.0	-99.9

Name: 20241213_2_035, ID: 402-Predose, Description:

Somaglutide (2)

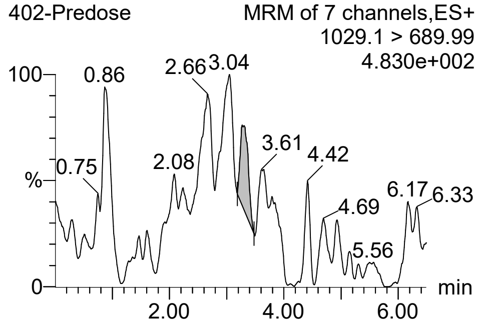

Tolbutamide (2)

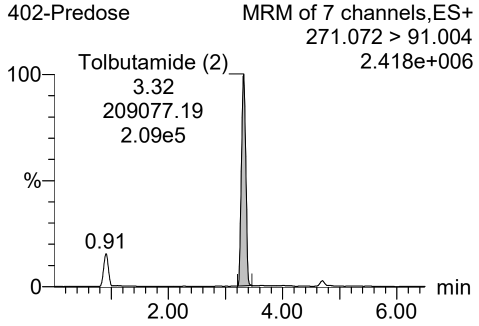

	#	Name	Trace	RT	Area	IS Area	Response	Primar...	Conc.	%Dev
1	1	Somaglutide (2)	1029.1 > 689.99	3.27	30.950	209077.188	0.000	bb	1.0	
2	2	Tolbutamide (2)	271.072 > 91.004	3.32	209077.188		209077.188	bb	1.0	-3.3

Dataset: D:\Data\27013-24001-NG.PRO\20241213_Soma-Tu.qld

Last Altered: Tuesday, July 15, 2025 15:41:54 China Standard Time
Printed: Tuesday, July 15, 2025 15:43:23 China Standard Time

Name: 20241213_2_036, ID: 402-2h, Description:

Somaglutide (2)

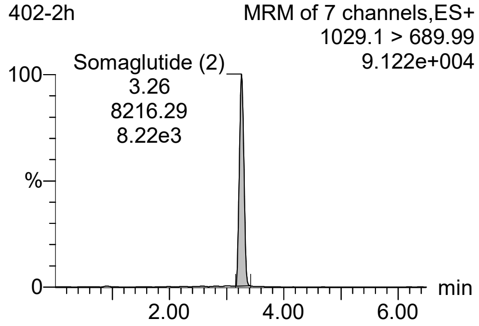

Tolbutamide (2)

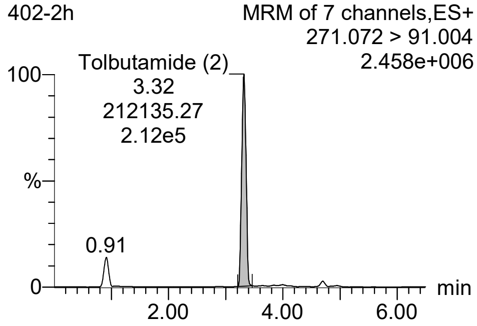

	#	Name	Trace	RT	Area	IS Area	Response	Primar...	Conc.	%Dev
1	1	Somaglutide (2)	1029.1 > 689.99	3.26	8216.287	212135.266	0.039	bb	261.7	
2	2	Tolbutamide (2)	271.072 > 91.004	3.32	212135.266		212135.266	bb	1.0	-1.9

Name: 20241213_2_037, ID: 402-4h, Description:

Somaglutide (2)

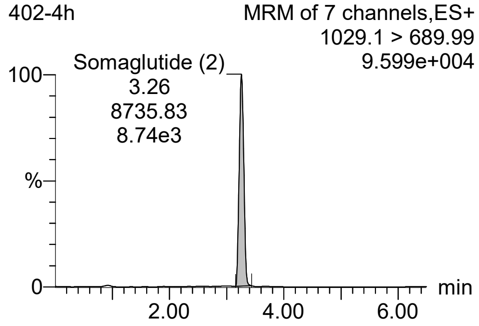

Tolbutamide (2)

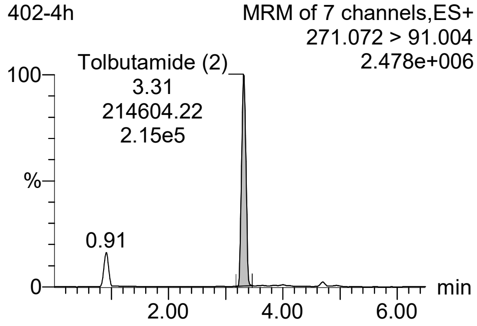

	#	Name	Trace	RT	Area	IS Area	Response	Primar...	Conc.	%Dev
1	1	Somaglutide (2)	1029.1 > 689.99	3.26	8735.833	214604.219	0.041	bb	275.0	
2	2	Tolbutamide (2)	271.072 > 91.004	3.31	214604.219		214604.219	bb	1.0	-0.8

Name: 20241213_2_038, ID: 402-8h, Description:

Somaglutide (2)

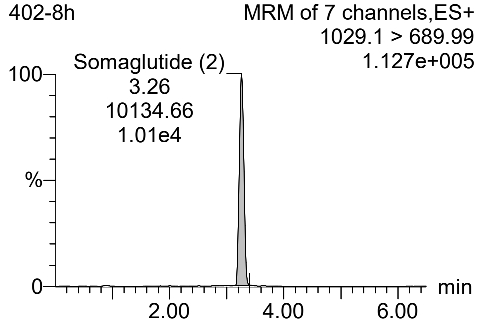

Tolbutamide (2)

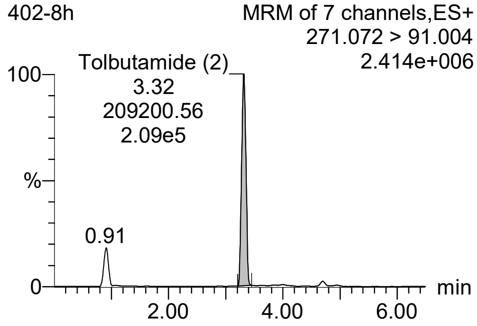

	#	Name	Trace	RT	Area	IS Area	Response	Primar...	Conc.	%Dev
1	1	Somaglutide (2)	1029.1 > 689.99	3.26	10134.659	209200.563	0.048	bb	327.3	
2	2	Tolbutamide (2)	271.072 > 91.004	3.32	209200.563		209200.563	bb	1.0	-3.2

Name: 20241213_2_039, ID: 402-12h, Description:

Somaglutide (2)

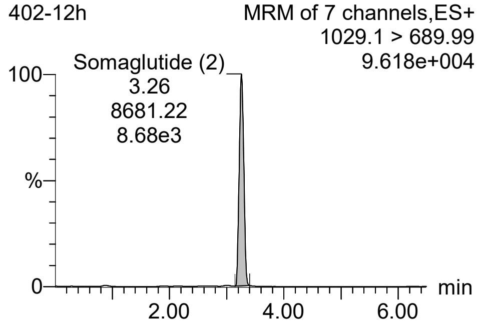

Tolbutamide (2)

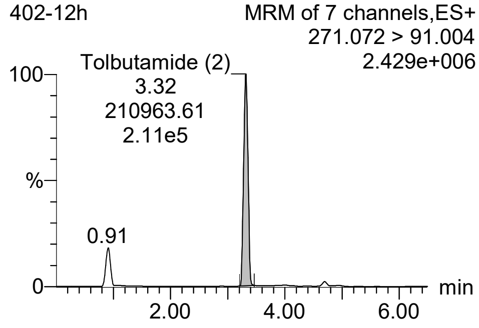

	#	Name	Trace	RT	Area	IS Area	Response	Primar...	Conc.	%Dev
1	1	Somaglutide (2)	1029.1 > 689.99	3.26	8681.223	210963.609	0.041	bb	278.0	
2	2	Tolbutamide (2)	271.072 > 91.004	3.32	210963.609		210963.609	bb	1.0	-2.4

Name: 20241213_2_040, ID: 402-24h, Description:

Somaglutide (2)

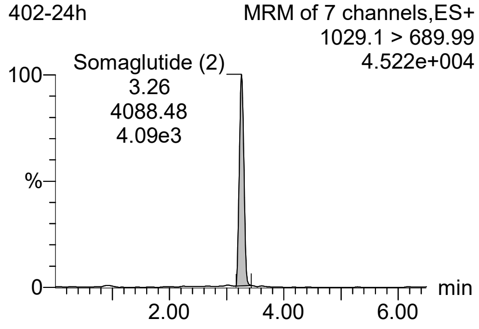

Tolbutamide (2)

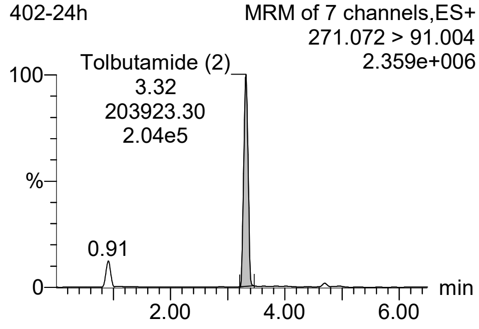

	#	Name	Trace	RT	Area	IS Area	Response	Primar...	Conc.	%Dev
1	1	Somaglutide (2)	1029.1 > 689.99	3.26	4088.476	203923.297	0.020	bb	135.4	
2	2	Tolbutamide (2)	271.072 > 91.004	3.32	203923.297		203923.297	bb	0.9	-5.7

Name: 20241213_2_041, ID: 402-48h, Description:

Somaglutide (2)

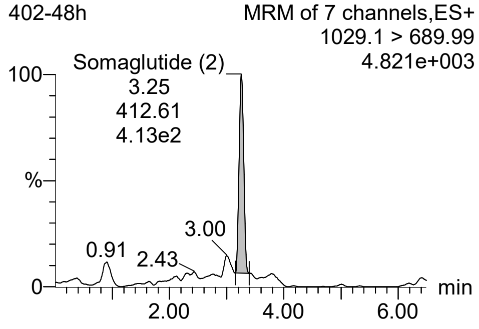

Tolbutamide (2)

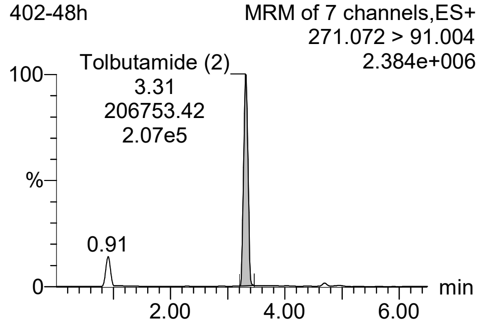

	#	Name	Trace	RT	Area	IS Area	Response	Primar...	Conc.	%Dev
1	1	Somaglutide (2)	1029.1 > 689.99	3.25	412.613	206753.422	0.002	bb	13.4	
2	2	Tolbutamide (2)	271.072 > 91.004	3.31	206753.422		206753.422	bb	1.0	-4.4

Dataset: D:\Data\27013-24001-NG.PRO\20241213_Soma-Tu.qld

Last Altered: Tuesday, July 15, 2025 15:41:54 China Standard Time
Printed: Tuesday, July 15, 2025 15:43:23 China Standard Time

Name: 20241213_2_042, ID: 402-72h, Description:

Somaglutide (2)

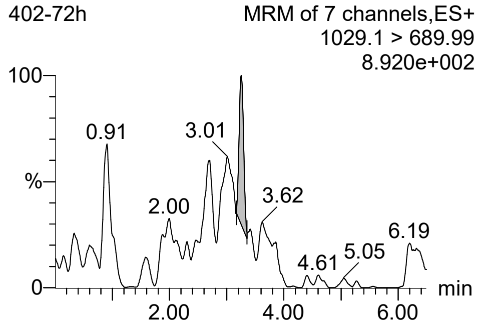

Tolbutamide (2)

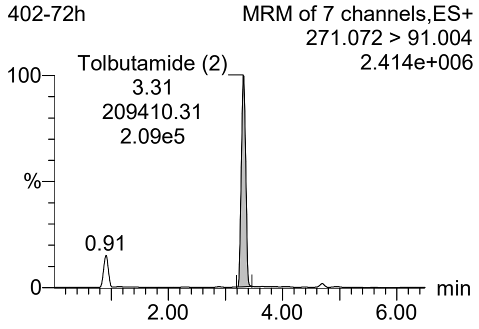

	#	Name	Trace	RT	Area	IS Area	Response	Primar...	Conc.	%Dev
1	1	Somaglutide (2)	1029.1 > 689.99	3.25	56.528	209410.313	0.000	bd	1.8	
2	2	Tolbutamide (2)	271.072 > 91.004	3.31	209410.313		209410.313	bb	1.0	-3.2

Name: 20241213_2_043, ID: 402-96h, Description:

Somaglutide (2)

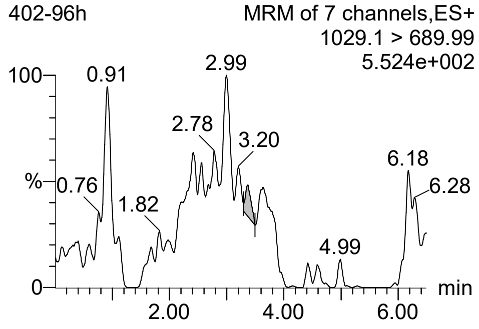

Tolbutamide (2)

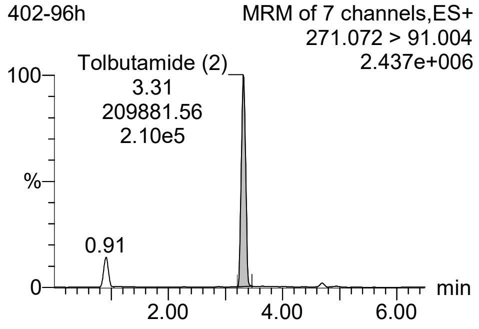

	#	Name	Trace	RT	Area	IS Area	Response	Primar...	Conc.	%Dev
1	1	Somaglutide (2)	1029.1 > 689.99	3.37	9.397	209881.563	0.000	db	0.3	
2	2	Tolbutamide (2)	271.072 > 91.004	3.31	209881.563		209881.563	bb	1.0	-2.9

Name: 20241213_2_044, ID: 402-168h, Description:

Somaglutide (2)

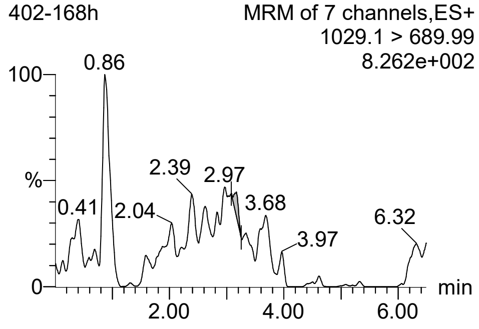

Tolbutamide (2)

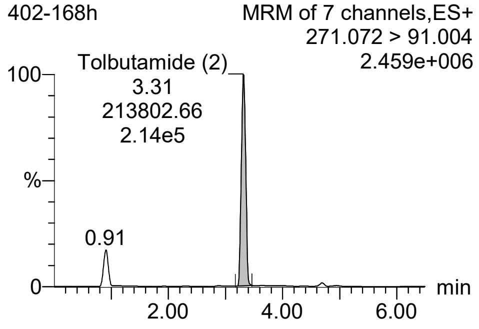

	#	Name	Trace	RT	Area	IS Area	Response	Primar...	Conc.	%Dev
1	1	Somaglutide (2)	1029.1 > 689.99	3.17	7.822	213802.656	0.000	bb	0.2	
2	2	Tolbutamide (2)	271.072 > 91.004	3.31	213802.656		213802.656	bb	1.0	-1.1

Dataset: D:\Data\27013-24001-NG.PRO\20241213_Soma-Tu.qld

Last Altered: Tuesday, July 15, 2025 15:41:54 China Standard Time
Printed: Tuesday, July 15, 2025 15:43:23 China Standard Time

Name: 20241213_2_045, ID: Solvent, Description:

Somaglutide (2)

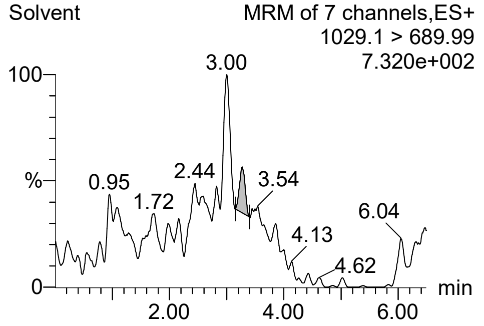

Tolbutamide (2)

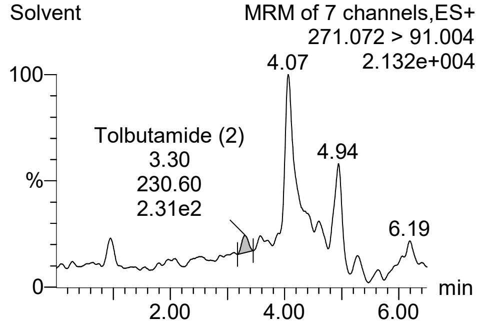

	# Name	Trace	RT	Area	IS Area	Response	Primar...	Conc.	%Dev
1	1 Somaglutide (2)	1029.1 > 689.99	3.27	17.643	230.597	0.077	bb	517.0	
2	2 Tolbutamide (2)	271.072 > 91.004	3.30	230.597		230.597	bb	0.0	-99.9

Name: 20241213_2_046, ID: Solvent, Description:

Somaglutide (2)

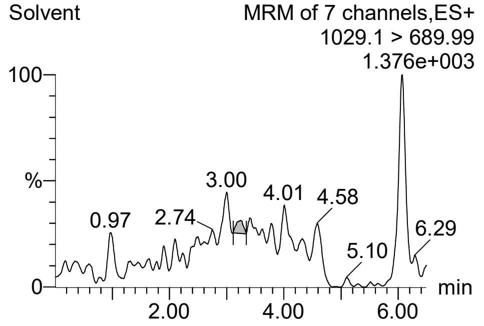

Tolbutamide (2)

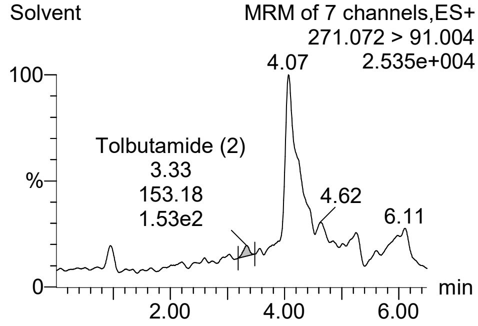

	# Name	Trace	RT	Area	IS Area	Response	Primar...	Conc.	%Dev
1	1 Somaglutide (2)	1029.1 > 689.99	3.24	12.137	153.181	0.079	bd	535.4	
2	2 Tolbutamide (2)	271.072 > 91.004	3.33	153.181		153.181	bb	0.0	-99.9

Name: 20241213_2_047, ID: 403-Predose, Description:

Somaglutide (2)

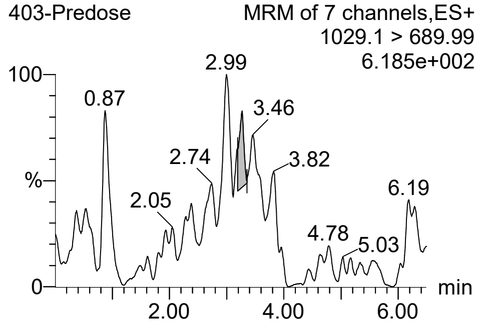

Tolbutamide (2)

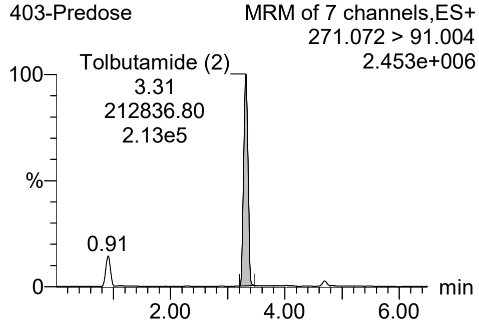

	# Name	Trace	RT	Area	IS Area	Response	Primar...	Conc.	%Dev
1	1 Somaglutide (2)	1029.1 > 689.99	3.27	20.738	212836.797	0.000	dd	0.6	
2	2 Tolbutamide (2)	271.072 > 91.004	3.31	212836.797		212836.797	bb	1.0	-1.6

Dataset: D:\Data\27013-24001-NG.PRO\20241213_Soma-Tu.qld

Last Altered: Tuesday, July 15, 2025 15:41:54 China Standard Time
Printed: Tuesday, July 15, 2025 15:43:23 China Standard Time

Name: 20241213_2_048, ID: 403-2h, Description:

Somaglutide (2)

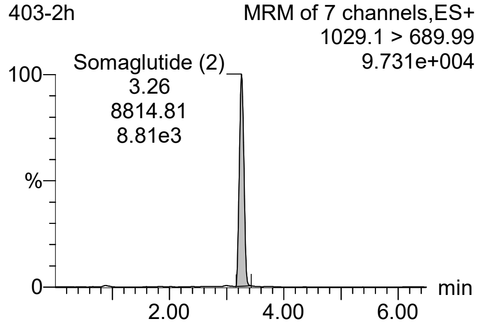

Tolbutamide (2)

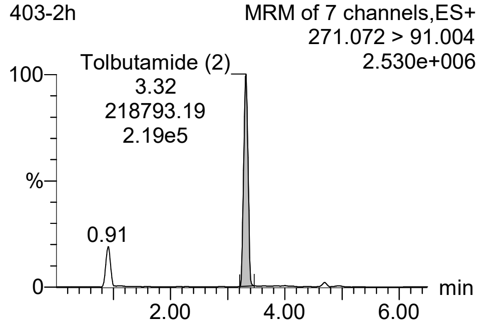

	#	Name	Trace	RT	Area	IS Area	Response	Primar...	Conc.	%Dev
1	1	Somaglutide (2)	1029.1 > 689.99	3.26	8814.810	218793.188	0.040	bb	272.2	
2	2	Tolbutamide (2)	271.072 > 91.004	3.32	218793.188		218793.188	bb	1.0	1.2

Name: 20241213_2_049, ID: 403-4h, Description:

Somaglutide (2)

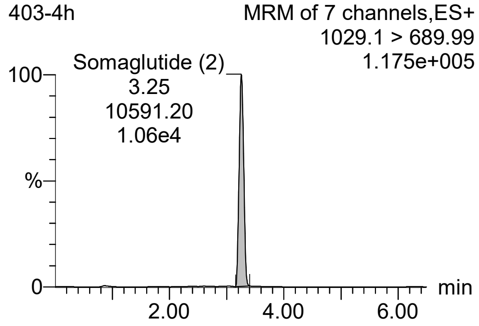

Tolbutamide (2)

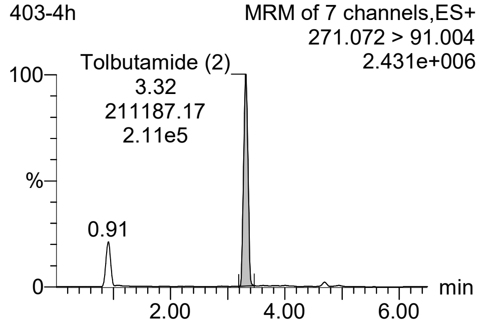

	#	Name	Trace	RT	Area	IS Area	Response	Primar...	Conc.	%Dev
1	1	Somaglutide (2)	1029.1 > 689.99	3.25	10591.197	211187.172	0.050	bb	338.9	
2	2	Tolbutamide (2)	271.072 > 91.004	3.32	211187.172		211187.172	bb	1.0	-2.3

Name: 20241213_2_050, ID: 403-8h, Description:

Somaglutide (2)

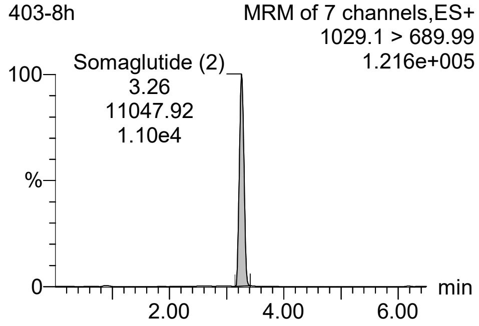

Tolbutamide (2)

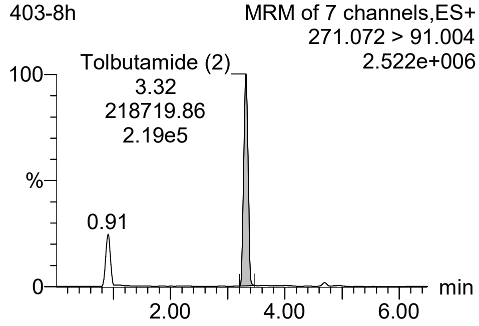

	#	Name	Trace	RT	Area	IS Area	Response	Primar...	Conc.	%Dev
1	1	Somaglutide (2)	1029.1 > 689.99	3.26	11047.925	218719.859	0.051	bb	341.3	
2	2	Tolbutamide (2)	271.072 > 91.004	3.32	218719.859		218719.859	bb	1.0	1.2

Dataset: D:\Data\27013-24001-NG.PRO\20241213_Soma-Tu.qld

Last Altered: Tuesday, July 15, 2025 15:41:54 China Standard Time
Printed: Tuesday, July 15, 2025 15:43:23 China Standard Time

Name: 20241213_2_051, ID: 403-12h, Description:

Somaglutide (2)

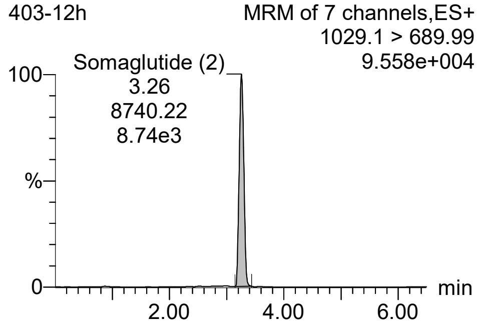

Tolbutamide (2)

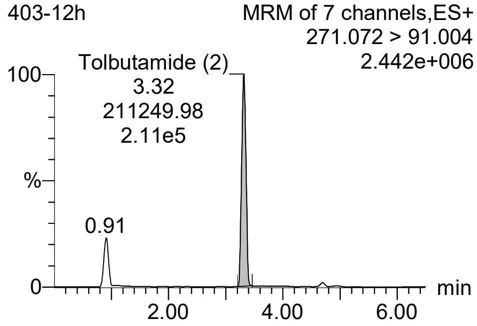

	#	Name	Trace	RT	Area	IS Area	Response	Primar...	Conc.	%Dev
1	1	Somaglutide (2)	1029.1 > 689.99	3.26	8740.216	211249.984	0.041	bb	279.5	
2	2	Tolbutamide (2)	271.072 > 91.004	3.32	211249.984		211249.984	bb	1.0	-2.3

Name: 20241213_2_052, ID: 403-24h, Description:

Somaglutide (2)

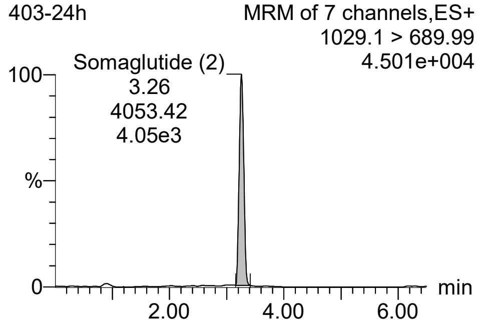

Tolbutamide (2)

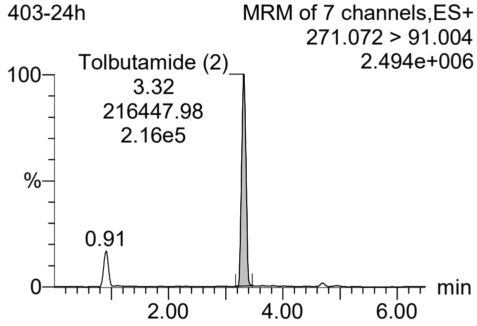

	#	Name	Trace	RT	Area	IS Area	Response	Primar...	Conc.	%Dev
1	1	Somaglutide (2)	1029.1 > 689.99	3.26	4053.415	216447.984	0.019	bb	126.5	
2	2	Tolbutamide (2)	271.072 > 91.004	3.32	216447.984		216447.984	bb	1.0	0.1

Name: 20241213_2_053, ID: 403-48h, Description:

Somaglutide (2)

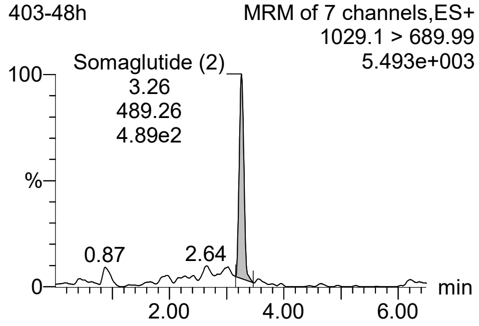

Tolbutamide (2)

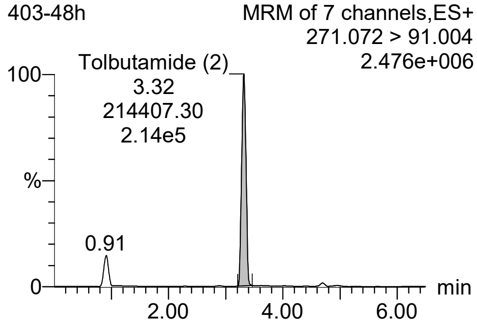

	#	Name	Trace	RT	Area	IS Area	Response	Primar...	Conc.	%Dev
1	1	Somaglutide (2)	1029.1 > 689.99	3.26	489.261	214407.297	0.002	bb	15.4	
2	2	Tolbutamide (2)	271.072 > 91.004	3.32	214407.297		214407.297	bb	1.0	-0.8

Dataset: D:\Data\27013-24001-NG.PRO\20241213_Soma-Tu.qld

Last Altered: Tuesday, July 15, 2025 15:41:54 China Standard Time
Printed: Tuesday, July 15, 2025 15:43:23 China Standard Time

Name: 20241213_2_054, ID: 403-72h, Description:

Somaglutide (2)

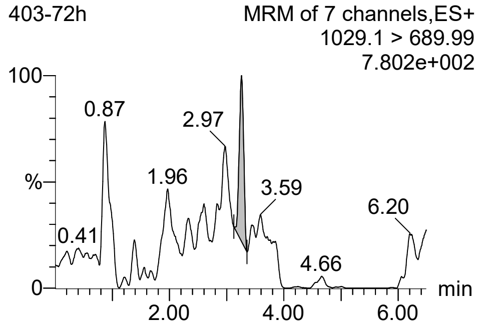

Tolbutamide (2)

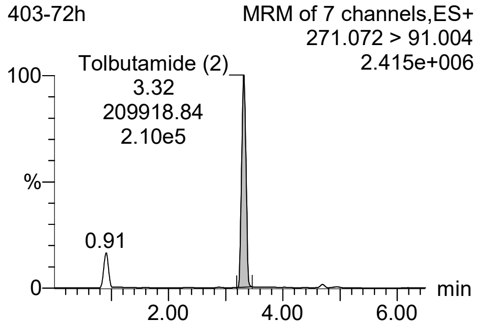

	#	Name	Trace	RT	Area	IS Area	Response	Primar...	Conc.	%Dev
1	1	Somaglutide (2)	1029.1 > 689.99	3.26	53.471	209918.844	0.000	bb	1.7	
2	2	Tolbutamide (2)	271.072 > 91.004	3.32	209918.844		209918.844	bb	1.0	-2.9

Name: 20241213_2_055, ID: 403-96h, Description:

Somaglutide (2)

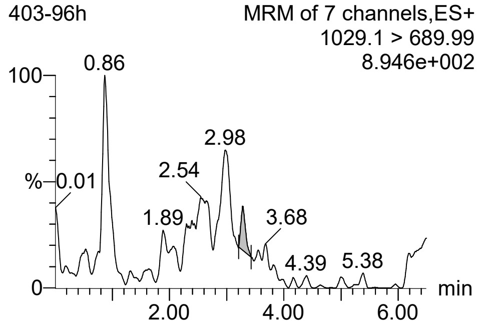

Tolbutamide (2)

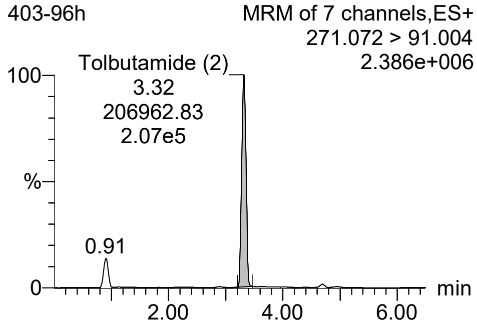

	#	Name	Trace	RT	Area	IS Area	Response	Primar...	Conc.	%Dev
1	1	Somaglutide (2)	1029.1 > 689.99	3.28	16.917	206962.828	0.000	bb	0.5	
2	2	Tolbutamide (2)	271.072 > 91.004	3.32	206962.828		206962.828	bb	1.0	-4.3

Name: 20241213_2_056, ID: 403-168h, Description:

Somaglutide (2)

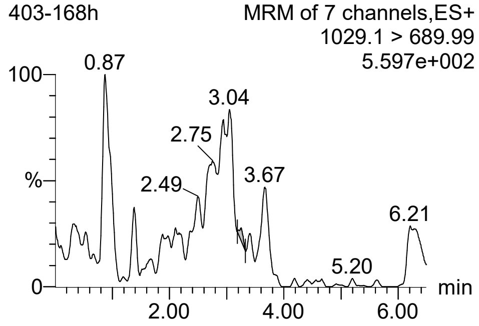

Tolbutamide (2)

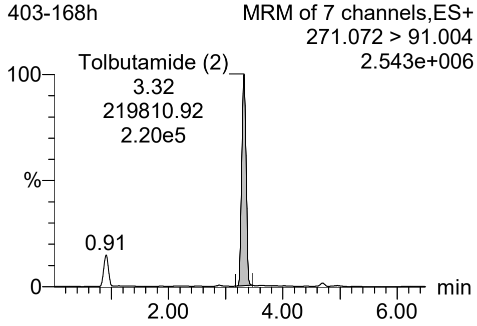

	#	Name	Trace	RT	Area	IS Area	Response	Primar...	Conc.	%Dev
1	1	Somaglutide (2)	1029.1 > 689.99	3.25	2.436	219810.922	0.000	bb	0.0	
2	2	Tolbutamide (2)	271.072 > 91.004	3.32	219810.922		219810.922	bb	1.0	1.7

Dataset: D:\Data\27013-24001-NG.PRO\20241213_Soma-Tu.qld

Last Altered: Tuesday, July 15, 2025 15:41:54 China Standard Time
Printed: Tuesday, July 15, 2025 15:43:23 China Standard Time

Name: 20241213_2_057, ID: Solvent, Description:

Somaglutide (2)

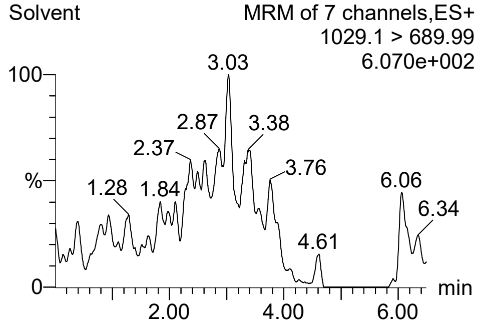

Tolbutamide (2)

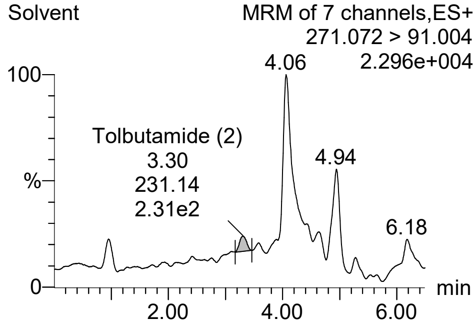

	# Name	Trace	RT	Area	IS Area	Response	Primar...	Conc.	%Dev
1	1 Somaglutide (2)	1029.1 > 689.99			231.137				
2	2 Tolbutamide (2)	271.072 > 91.004	3.30	231.137		231.137	bb	0.0	-99.9

Name: 20241213_2_058, ID: Solvent, Description:

Somaglutide (2)

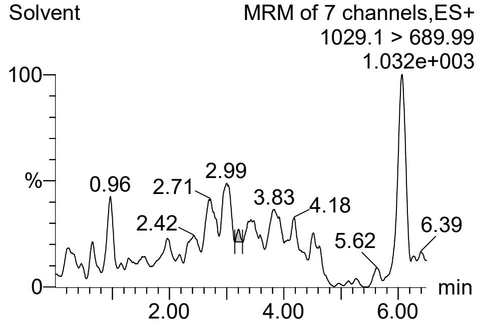

Tolbutamide (2)

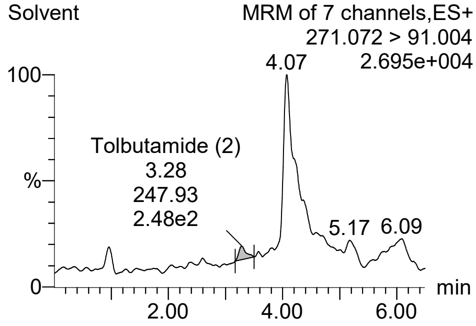

	# Name	Trace	RT	Area	IS Area	Response	Primar...	Conc.	%Dev
1	1 Somaglutide (2)	1029.1 > 689.99	3.21	4.026	247.934	0.016	bb	109.7	
2	2 Tolbutamide (2)	271.072 > 91.004	3.28	247.934		247.934	bb	0.0	-99.9

Name: 20241213_2_059, ID: B, Description:

Somaglutide (2)

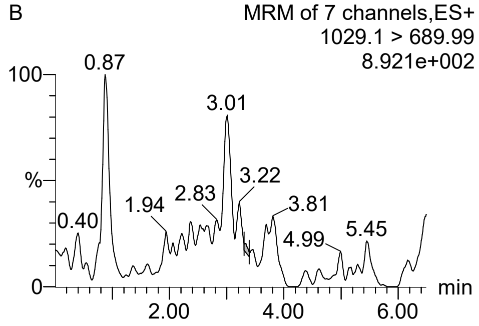

Tolbutamide (2)

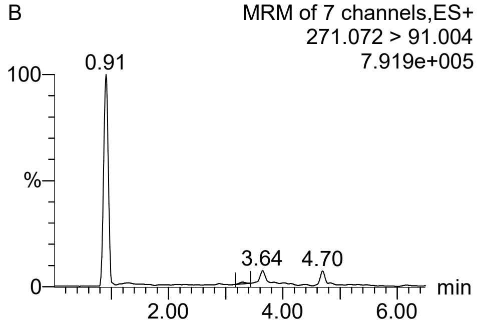

	# Name	Trace	RT	Area	IS Area	Response	Primar...	Conc.	%Dev
1	1 Somaglutide (2)	1029.1 > 689.99	3.32	2.601	712.111	0.004	dd	24.6	
2	2 Tolbutamide (2)	271.072 > 91.004	3.29	712.111		712.111	bb	0.0	-99.7

Dataset: D:\Data\27013-24001-NG.PRO\20241213_Soma-Tu.qld

Last Altered: Tuesday, July 15, 2025 15:41:54 China Standard Time
Printed: Tuesday, July 15, 2025 15:43:23 China Standard Time

Name: 20241213_2_060, ID: O, Description:

Somaglutide (2)

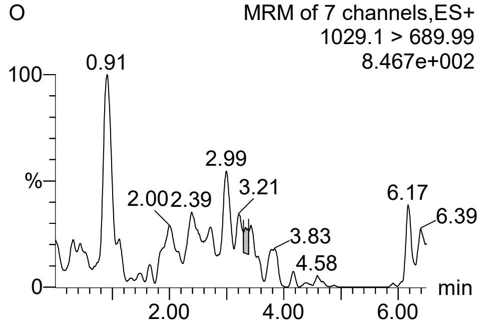

Tolbutamide (2)

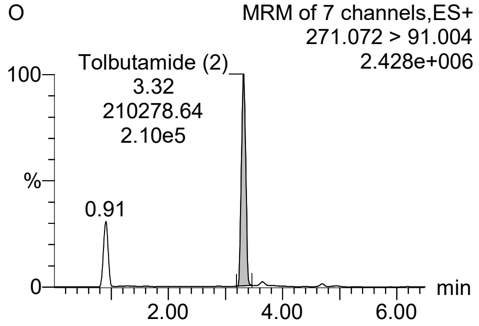

	#	Name	Trace	RT	Area	IS Area	Response	Primar...	Conc.	%Dev
1	1	Somaglutide (2)	1029.1 > 689.99	3.33	8.757	210278.641	0.000	dd	0.2	
2	2	Tolbutamide (2)	271.072 > 91.004	3.32	210278.641		210278.641	bb	1.0	-2.8

Name: 20241213_2_061, ID: Q1, Description:

Somaglutide (2)

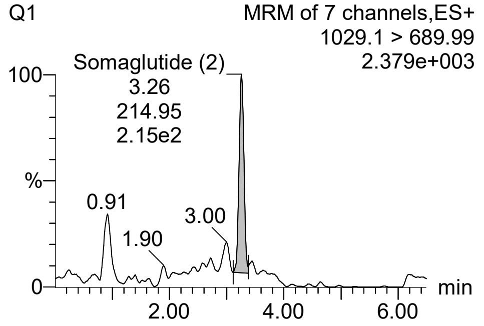

Tolbutamide (2)

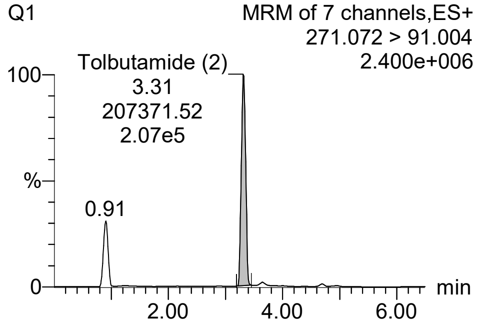

	#	Name	Trace	RT	Area	IS Area	Response	Primar...	Conc.	%Dev
1	1	Somaglutide (2)	1029.1 > 689.99	3.26	214.952	207371.516	0.001	bd	7.0	16.0
2	2	Tolbutamide (2)	271.072 > 91.004	3.31	207371.516		207371.516	bb	1.0	-4.1

Name: 20241213_2_062, ID: Q2, Description:

Somaglutide (2)

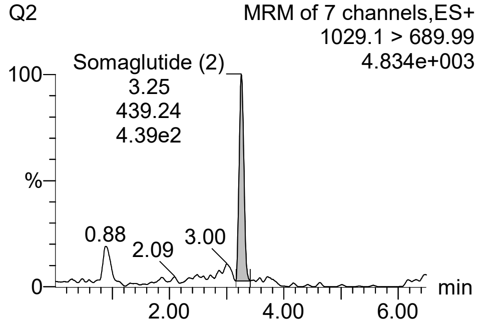

Tolbutamide (2)

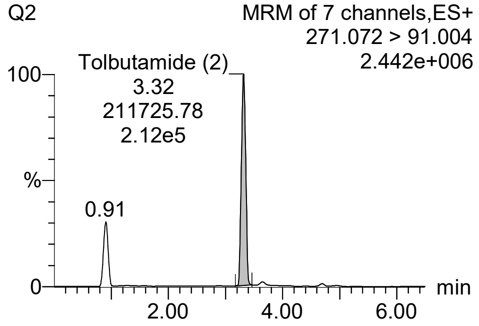

	#	Name	Trace	RT	Area	IS Area	Response	Primar...	Conc.	%Dev
1	1	Somaglutide (2)	1029.1 > 689.99	3.25	439.237	211725.781	0.002	bb	14.0	16.4
2	2	Tolbutamide (2)	271.072 > 91.004	3.32	211725.781		211725.781	bb	1.0	-2.1

Dataset: D:\Data\27013-24001-NG.PRO\20241213_Soma-Tu.qld

Last Altered: Tuesday, July 15, 2025 15:41:54 China Standard Time
Printed: Tuesday, July 15, 2025 15:43:23 China Standard Time

Name: 20241213_2_063, ID: Q3, Description:

Somaglutide (2)

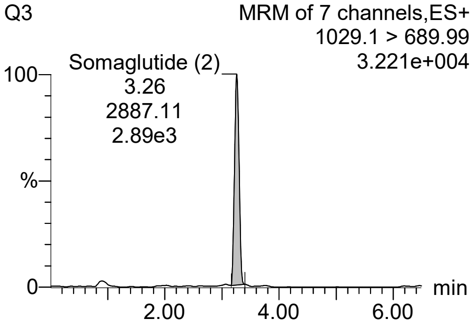

Tolbutamide (2)

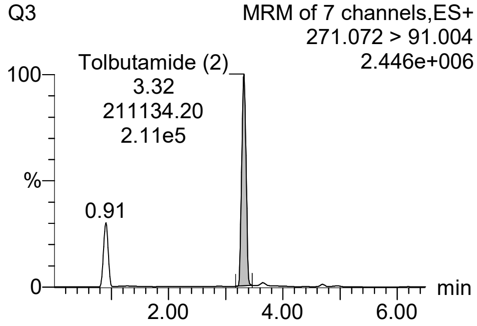

	#	Name	Trace	RT	Area	IS Area	Response	Primar...	Conc.	%Dev
1	1	Somaglutide (2)	1029.1 > 689.99	3.26	2887.114	211134.203	0.014	bb	92.4	15.4
2	2	Tolbutamide (2)	271.072 > 91.004	3.32	211134.203		211134.203	bb	1.0	-2.4

Name: 20241213_2_064, ID: Q4, Description:

Somaglutide (2)

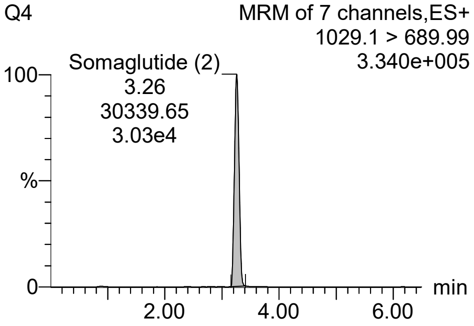

Tolbutamide (2)

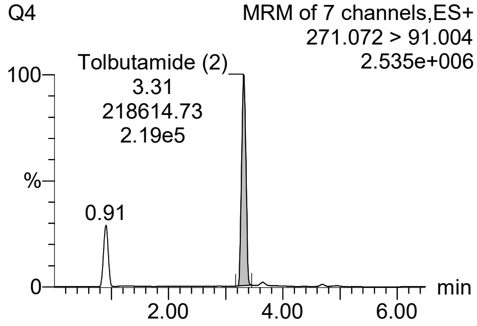

	#	Name	Trace	RT	Area	IS Area	Response	Primar...	Conc.	%Dev
1	1	Somaglutide (2)	1029.1 > 689.99	3.26	30339.646	218614.734	0.139	bb	937.8	17.2
2	2	Tolbutamide (2)	271.072 > 91.004	3.31	218614.734		218614.734	bb	1.0	1.1

Name: 20241213_2_065, ID: Solvent, Description:

Somaglutide (2)

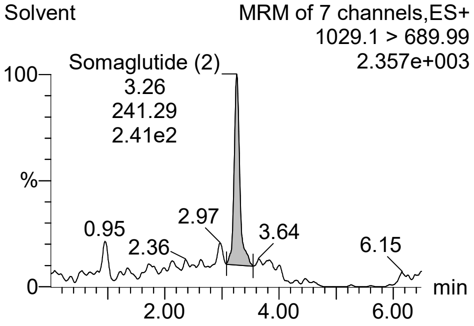

Tolbutamide (2)

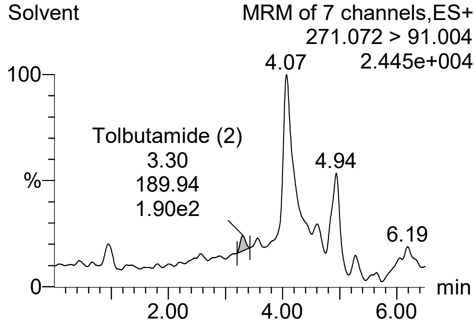

	#	Name	Trace	RT	Area	IS Area	Response	Primar...	Conc.	%Dev
1	1	Somaglutide (2)	1029.1 > 689.99	3.26	241.294	189.941	1.270	bb	8584.5	
2	2	Tolbutamide (2)	271.072 > 91.004	3.30	189.941		189.941	bb	0.0	-99.9

Dataset: D:\Data\27013-24001-NG.PRO\20241213_Soma-Tu.qld

Last Altered: Tuesday, July 15, 2025 15:41:54 China Standard Time
Printed: Tuesday, July 15, 2025 15:43:23 China Standard Time

Name: 20241213_2_066, ID: Solvent, Description:

Somaglutide (2)

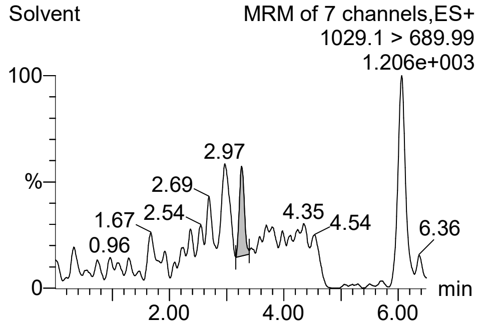

Tolbutamide (2)

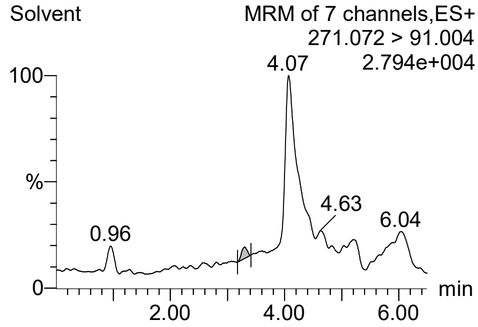

	#	Name	Trace	RT	Area	IS Area	Response	Primar...	Conc.	%Dev
1	1	Somaglutide (2)	1029.1 > 689.99	3.26	50.891	169.135	0.301	bd	2033.2	
2	2	Tolbutamide (2)	271.072 > 91.004	3.30	169.135		169.135	bb	0.0	-99.9
